# Pd-Catalyzed Cascade Reactions of Aziridines: One-Step Access to Complex Tetracyclic Amines

**DOI:** 10.1021/acs.orglett.1c01403

**Published:** 2021-06-16

**Authors:** Jonathan P. Knowles, Hannah G. Steeds, Maria Schwarz, Francesca Latter, Kevin I. Booker-Milburn

**Affiliations:** † School of Chemistry, University of Bristol, Cantock’s Close, Bristol BS8 1TS, U.K.; ‡ Department of Applied Sciences, Northumbria University, Newcastle upon Tyne NE1 8ST, U.K.

## Abstract

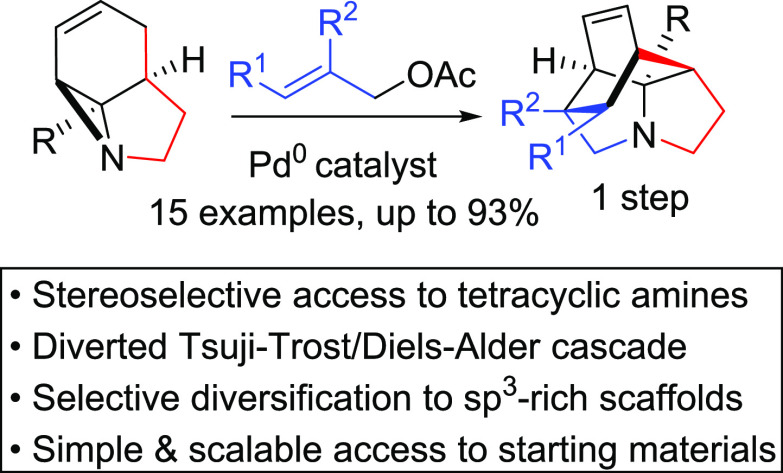

The combination of palladium catalysis and thermal cycloaddition is shown to transform tricyclic aziridines into complex, stereodefined tetracyclic products in a single step. This highly unusual cascade process involves a diverted Tsuji–Trost sequence leading to a surprisingly facile intramolecular Diels–Alder reaction. The starting materials are accessible on multigram scales from the photochemical rearrangement of simple pyrroles. The tetracyclic amine products can be further elaborated through routine transformations, highlighting their potential as scaffolds for medicinal chemistry.

Nitrogen-containing heterocycles are among the most prominent structural motifs within bioactive molecules, showing a wide range of activity, including anticancer, antibacterial, and antiviral activity, and some acting on the central nervous system (CNS).^1,2^ Compounds rich in sp^3^ character are known to perform favorably within the clinic, where their enhanced three-dimensionality leads to improved selectivity.^3^ Methodologies for accessing N-containing, complex three-dimensional scaffolds are therefore a key objective for synthetic chemists, potentially allowing rapid access to high-value lead compounds.^4^ Cascade reactions represent an ideal route to such compounds, necessarily adding significant complexity in a single transformation.^5^

Synthetic photochemistry has a long history of creating highly complex molecules.^6^ These products are frequently reactive, thus proving to be versatile intermediates in synthesis.^6,7^ Catalytic modification of such products continues to harbor interest, forming conformationally constrained, saturated heterocycles. We have previously shown tricyclic aziridines **2**, formed directly from pyrroles **1**,^8^ are particularly versatile intermediates in this respect (Scheme 1a).^9,10^ Herein, we report an efficient single-step approach to the hitherto unreported ring system **6** via a novel three-part cascade process.

**Scheme 1:**
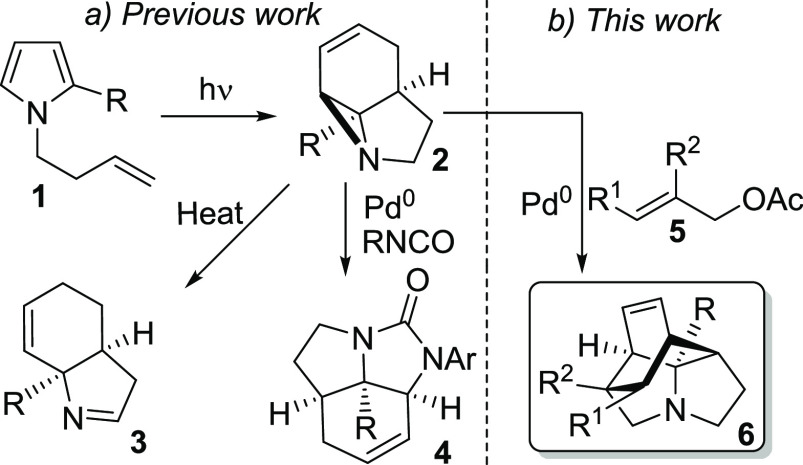
Previous and Current Photochemical/Catalytic Sequences to Form Complex Structures^9,10^

Previous Pd^0^-mediated ring expansion/cycloaddition of **2** with dipolarophiles gave access to five-membered rings such as **4**,^10^ and we were interested in determining whether extension to six-membered rings was possible. We therefore considered whether bifunctional reagent **8** could function as both a mild nucleophile and an electrophile, enabling formation of **10** (Scheme 2). Surprisingly, however, reaction of **2** (R = CO_2_^*t*^Bu) gave N-alkylated product **11**, where diene formation and desilylation had occurred. As dienes are key synthetic building blocks,^11^ we decided to investigate the scope of this reaction.

**Scheme 2:**
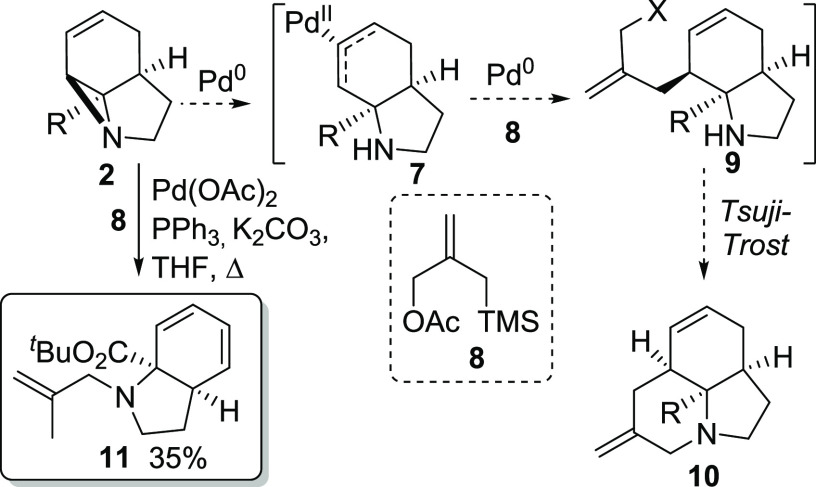
Planned Tsuji–Trost Pathway

Replacing **8** with allyl acetate converted **2** (R = CO_2_^*t*^Bu) to allylated product **12a** in a much-improved 87% yield (Table 1). These conditions also proved to be applicable to aziridines **2b** (R = COMe) and **2c** (R = CONHEt). Nitrile **2d** proved to be unsuccessful, possibly due to a decreased level of steric crowding of the aziridine ring.^12^ Use of allylic bromides rather than allylic acetates also proved to be possible but gave reduced yields and did not remove the requirement for Pd catalysis.

**Table 1:**
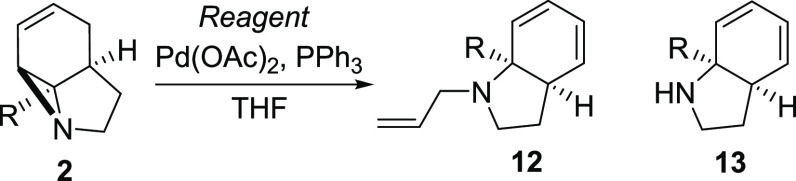
Effect of the Variation of the Aziridine and Allyl Reagent

entry	R	reagent	product	yield (%)
1^a^,^b^	CO_2_^*t*^Bu	allyl acetate	**12a**	87
2^b^,^c^	COMe	allyl acetate	**12b**	56^d^
3^b^,^c^	CONHEt	allyl acetate	**12c**	60^d^
4^a^,^b^	CN	allyl acetate	**12d**	0^e^
5^d^	CO_2_^*t*^Bu	none	**13a**	83
6^a^	COMe	none	**13b**	82
7^a^	CONHEt	none	**13c**	44
8^a^	CN	none	**13d**	0^e^
9^a^,^f^	CO_2_^*t*^Bu	none	**13a**	0
10^a^,^g^	CO_2_^*t*^Bu	none	**13a**	0

a Reaction performed at 70 °C.

b Performed in the presence of 1.3 equiv of K_2_CO_3_.

c Reaction performed at 30 °C.

d Yield determined by ^1^H NMR using 1,3,5-trimethoxybenzene as the internal standard.

e Slow conversion to retro-ene product **3** was observed.^9^

f Performed using Pd(PPh_3_)_4_.

g Performed using Pd_2_(dba)_3_/PPh_3_.

Reaction in the absence of an allylating reagent also proved to be successful, forming secondary amino-dienes **13a–c** in good yield (entries 5–7, respectively). This was found to proceed most efficiently in the absence of K_2_CO_3_, and again nitrile **2d** proved to be unreactive. Interestingly, these reactions proved to be unsuccessful when other Pd(0)/PPh_3_-based systems were employed (entries 9 and 10), suggesting a byproduct of catalyst activation might play a key role in aziridine N activation. Consistent with this, the presence of a mild Lewis or Brønsted acid was found to be essential for the reaction to occur (see the Supporting Information for full details).

We then turned our attention to exploiting the dienyl component of these cyclic dienes **12**. Diels–Alder reaction of N-allyl derivative **12a** with maleimide formed the expected adduct **14** (Scheme 3). However, we were intrigued to isolate trace amounts of the intramolecular Diels–Alder (IMDA) reaction product **15**, which was unexpected given the unactivated nature of the dienophile. Simply heating **12a** led to formation of **15** in an excellent 93% yield, demonstrating rapid access to a complex unreported, ring system (three steps from pyrrole **1a**(13)).

**Scheme 3:**
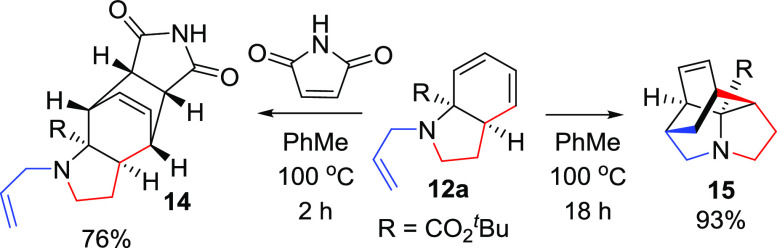
Inter- and Intramolecular Diels–Alder Reactions of **12a**

To explore this further, we expanded the range of allylating reagents and moved to performing the ring-opening/cycloaddition sequence in a single step. This proved to be highly successful, with use of an electron-withdrawing functionality at position 2 of component **5** being well tolerated and accelerating the Diels–Alder reaction (Scheme 4). One-pot reaction of **2a** required refluxing in dioxane to effect full conversion in the Tsuji–Trost reaction; however, the less sterically hindered aziridines **2b** and **2c** were found to react fully in THF. Importantly, scale-up of these reactions proved to be facile, with **6aa**, **6ab**, and **6bc** being formed in equal or increased yield on a 3 mmol scale.

**Scheme 4:**
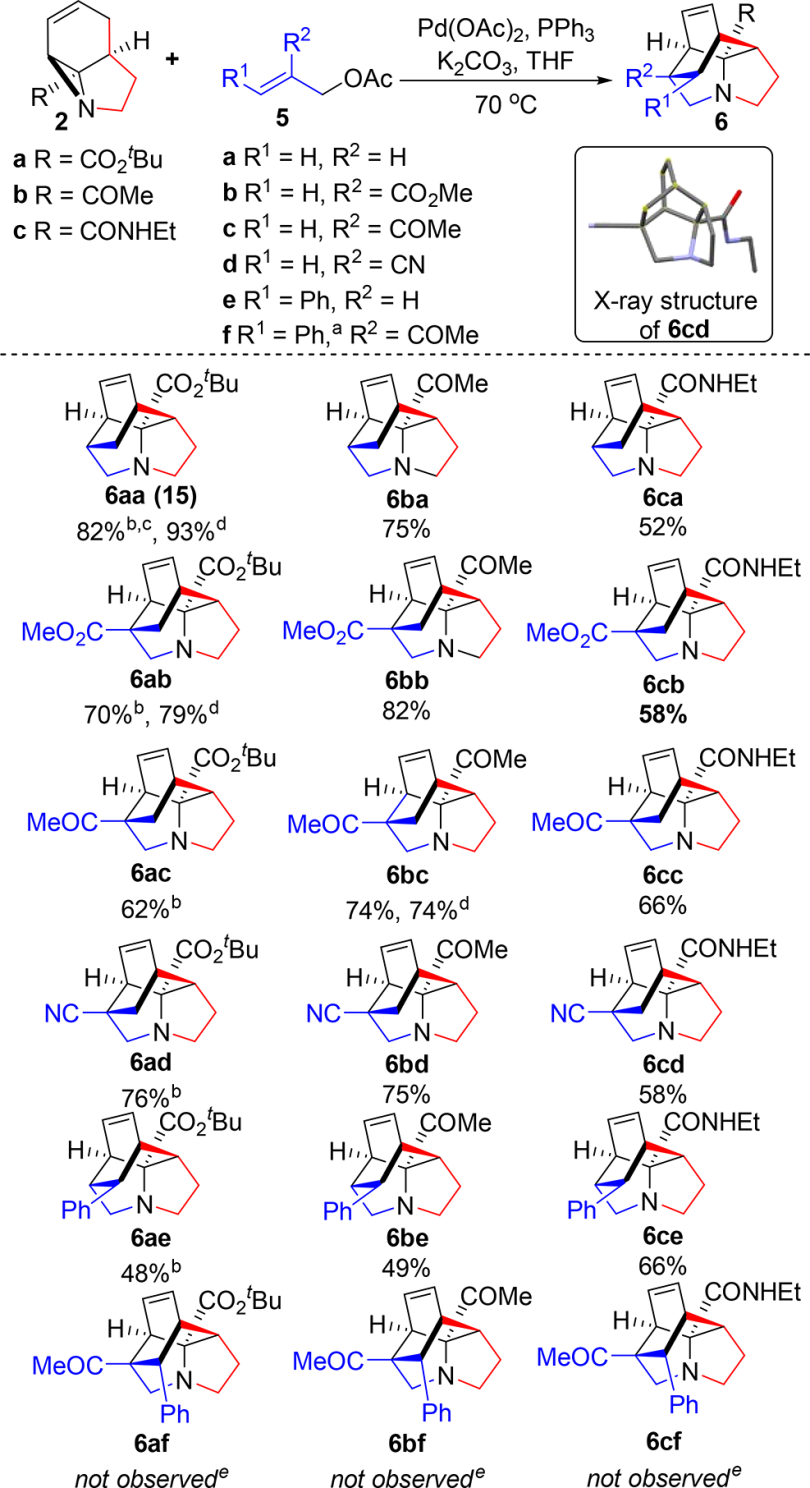
Scope and Limitations of the Tandem Ring-Opening/Diels–Alder Process Substituted with R^1^ at the methylene rather than the alkenyl position. Performed in dioxane at 100 °C. With 3 equiv of allyl acetate. On a 3 mmol scale. Intermediates **12af–cf** were isolated in 45%, 46%, and 29% yields, respectively.

Reactions of 3-substituted tether **5e** also proved to be successful, with high regiocontrol for the linear allylated intermediate combining with high *E* selectivity to yield a single stereoisomer. However, attempted reactions of disubstituted allyl acetate **5f** were less successful, with only the allylated diene intermediate being obtained. This likely reflects increased steric demand, where the phenyl substituent of the *E*-alkene would need to adopt an unfavorable *endo*-cyclic position in the transition state.

The cycloaddition step was seen to occur under conditions substantially milder than those of similar IMDA reactions.^14^ Indeed, substrates lacking an activated dienophile (i.e., **12a–c**) reacted at 70 °C, and we chose to investigate this further. As observed above, ^*t*^Bu system **12a** proved to be less reactive than amide **12c** (*k* = 6.8 × 10^–6^ s^–1^ vs *k* = 5.5 × 10^–5^ s^–1^ at 75 °C). An Eyring study (Figure 1) demonstrated this variation to be largely controlled by the enthalpy of activation, with a 20 kJ mol^–1^ difference between **12a** and **12c**. While it is unclear whether this increase is due entirely to electronic factors or includes an additional conformational element, both values appear to be low when compared with those known for other IMDA reactions.^15^ Further attempts to explore the impact of the dienophile activation proved not to be possible due to appreciable formation of **6ab** even at 20 °C, again emphasizing the facile nature of this IMDA process.

**Figure 1:**
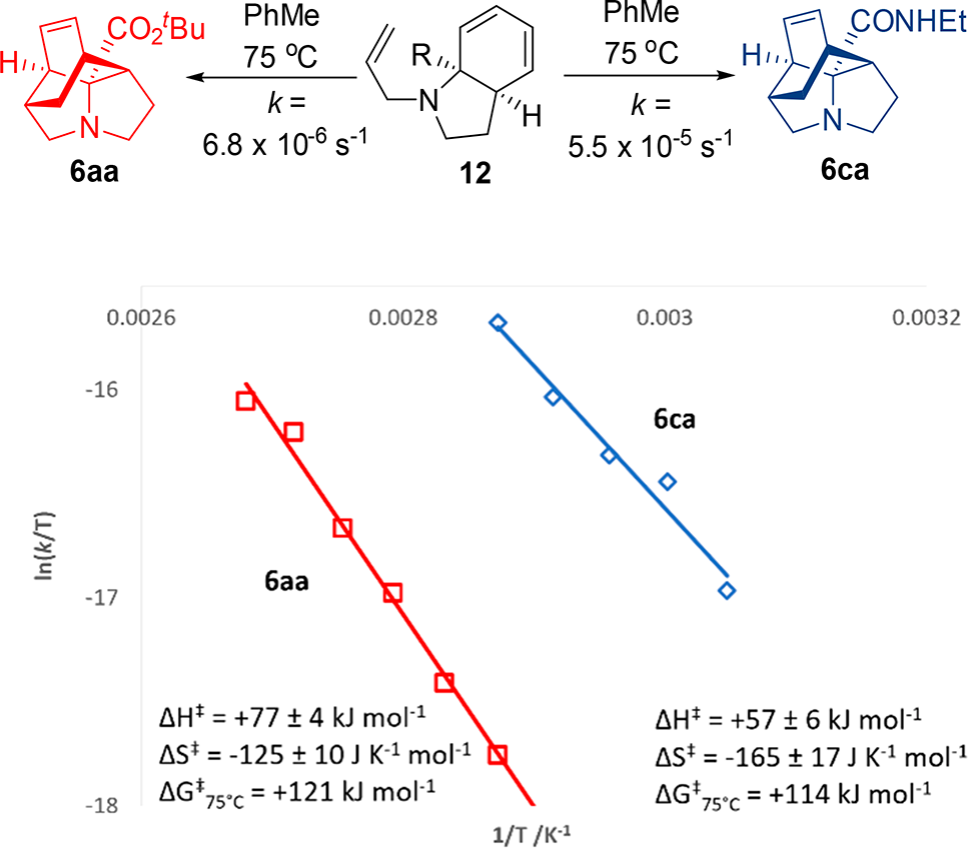
Eyring plots and thermodynamic parameters for the Diels–Alder cyclization to form **6aa** and **6ca**.

To explore the role of acetate observed in Table 1 [entries 9 and 10 (see also the Supporting Information)], compound **16** was prepared and subjected to the reaction conditions;^13^ however, diene **13a** was not observed, ruling this out as a potential intermediate (Scheme 5). Deuterated substrate **17** was also subjected to the reaction conditions, leading to the formation of **18** by cleavage of a single C–D bond. The kinetic isotope effect associated with this process was investigated through a competition reaction with **17** and **2a**, which showed essentially no difference in reaction rate (see the Supporting Information for details).

**Scheme 5:**
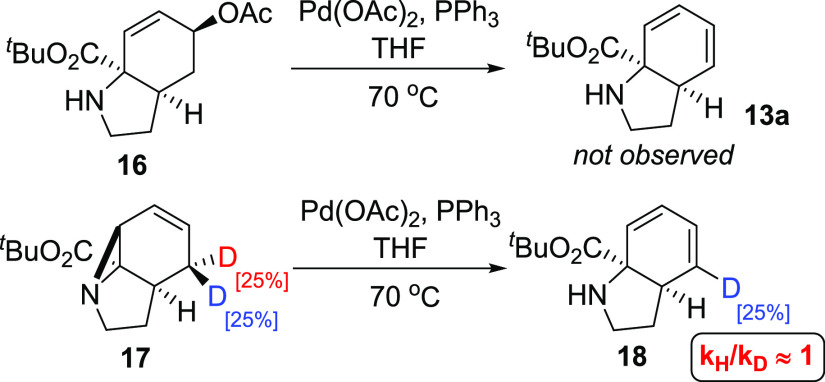
Mechanistic and Isotopic Labeling Studies

On the basis of this and the preceding results, the mechanism can be proposed (Scheme 6). Initial additive-assisted, Pd-catalyzed C–N cleavage of **2** leads to the formation of a π-allyl Pd intermediate **7**. This species then undergoes direct β-hydride elimination, even in the absence of additional base, to form intermediate diene **20**. What follows is likely to be a standard Tsuji–Trost mechanism between **20** and allyl acetate **5**, with the added base present serving to ensure sufficient levels of reactive free amine **20**. The lack of a significant KIE associated with this process, as determined by competition (i.e., between **17** and **2a**), is consistent with the first step (C–N cleavage) being turnover-limiting. This low KIE value necessarily means that a reversible β-hydride elimination cannot be ruled out.^16^ The resulting N-allylated product **12** then undergoes cycloaddition to form product **6**, the rate of which is controlled by the aziridine and allyl substituents. Although a Pd-catalyzed elimination/intermolecular DA process has been reported previously,^17^ to the best of our knowledge, this is the first example of a sequential Tsuji–Trost/IMDA cascade.^18,19^

**Scheme 6:**
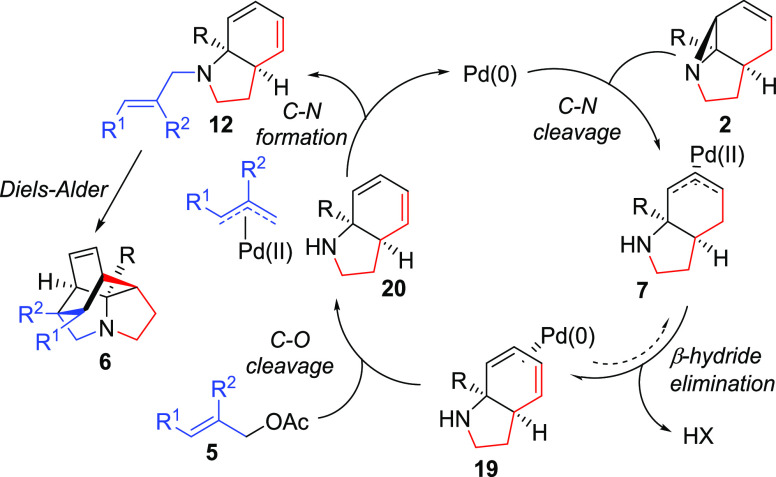
Proposed Mechanism

Given our previous discussion of the importance of a high sp^3^ content within drug discovery programs,^3^ we undertook a short study to diversify products **6** using routine transformations (Scheme 7). For example, in a telescoped oxidative cleavage/reductive amination sequence, compound **6aa** was efficiently transformed into tetracyclic amino ester **21**, possessing orthogonal protection for further functionalization. Alternatively, selective and sequential ester hydrolysis/amide formation gave **22** in a 47% yield overall, demonstrating potential for efficient two-dimensional amide library formation.

**Scheme 7:**
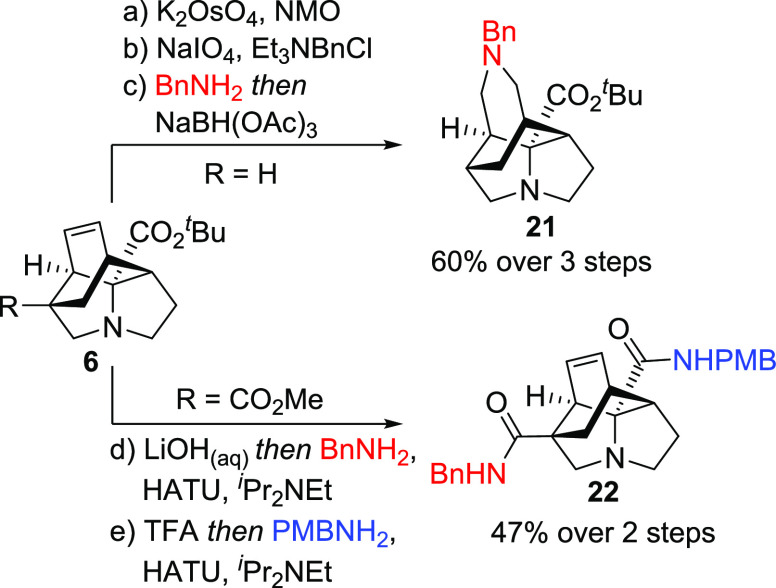
Functionalization of Tetracyclic Scaffolds

In conclusion, we have shown that stereodefined tetracycles **6** can be formed in only two steps from simple pyrroles, through initial photochemical conversion to aziridines **2**. These undergo a one-pot diverted Tsuji–Trost reaction, followed by a standard Tsuji–Trost reaction affording the allylated diene, which itself undergoes a direct IMDA reaction. The mechanism of diene formation likely involves rate-limiting acid-assisted C–N cleavage, followed by direct β-hydride elimination. These results underline the power of photochemical/catalytic sequences in preparing complex ring systems. Finally, we have shown that the tetracyclic amines formed from this cascade process undergo further functionalization reactions, highlighting their potential as sp^3^-rich scaffolds in drug discovery.

## Supporting Information Available

Experimental procedures, spectral and analytical data, copies of ^1^H and ^13^C NMR spectra for new compounds, and crystallographic data of **6cd** (PDF)

## Accession Codes

CCDC 2047771 contains the supplementary crystallographic data for this paper. These data can be obtained free of charge via www.ccdc.cam.ac.uk/data_request/cif, or by emailing data_request@ccdc.cam.ac.uk, or by contacting The Cambridge Crystallographic Data Centre, 12 Union Road, Cambridge CB2 1EZ, UK; fax: +44 1223 336033.
