# Supplementary material for: Pd-Catalyzed Cascade Reactions of Aziridines: One-Step Access to Complex Tetracyclic Amines
Source: Org Lett. 2021 Jun 16;23(13):4986–90. doi: 10.1021/acs.orglett.1c01403 (PMC8289308; doi:10.1021/acs.orglett.1c01403)
Supplement: Supplementary file 1 — ol1c01403_si_001.pdf [file ol1c01403_si_001.pdf]

Pd-Catalyzed Cascade Reactions of Aziridines: One-step Access to Complex Tetracyclic Amines

Jonathan P. Knowles,‡ Hannah G. Steeds,‡† Maria Schwarz,† Francesca Latter† and Kevin I. Booker-Milburn*†*

†School of Chemistry, University of Bristol, Cantock's Close, Bristol, UK, BS8 1TS
‡Department of Applied Sciences, Northumbria University, Newcastle upon Tyne, UK, NE1 8ST

Table of Contents

General Information	S3
Experimental Procedures	S4
General Procedures	S4
Additive Screen	S4
Eyring Data	S4
Synthesis of Dienes	S6
Synthesis of Tethers	S8
Synthesis of Diels-Alder Products	S10
Competition Experiment	S18
Synthesis of Deuterated Labelled Compounds & Mechanistic Probe	S19
Functionalization of Products	S21
Crystal Structure Data	S22
References	S23
NMR Spectra	S24

General Information

Unless otherwise stated chemicals were purchased from Sigma, Fluorochem or Alfa and used without further purification. Aziridines **2a-d** were prepared as described previously.[1,2] Dry solvents were obtained by passage through a column of anhydrous alumina using equipment from Anhydrous Engineering (University of Bristol) based on the Grubbs' design. Strauss flasks fitted with a J. Youngs valve were used to collect anhydrous solvent. Solvents used for the Pd-catalyzed reactions were degassed by bubbling with N_2. All other commercially available reagents were used as received. Reactions requiring anhydrous conditions were performed under N_2, glassware was flame dried immediately prior to use. Liquid reagents, solutions or solvents were added *via* syringe through rubber septa; solid reagents were added *via* Schlenk type adapters. Reaction mixtures were stirred magnetically. Unless otherwise stated reactions carried out at RT varied between 16-22 ºC depending on the season. The heating of reactions was performed using an oil bath.

Flash column chromatography was performed on Aldrich silica gel: 230-400 mesh (40-63 μm). Analytical thin layer chromatography was performed on aluminium backed 60 F_{254} silica plates. Visualisation was achieved by UV florescence (254 or 365 nm) and/or staining with $KMnO_4$ solution and heat. Extracts were concentrated *in vacuo* using both a Heidolph Hei-VAP Advantage rotary evaporator (bath temperatures up to 50 °C) at a pressure of 15 mmHg (diaphragm pump) and a high vacuum line at room temperature.

^{1}H-NMR and ^{13}C-NMR spectra were measured at 25 °C in the solvent specified with Varian, Jeol or Bruker spectrometers operating at field strengths listed. Chemical shifts (δ) are quoted in parts per million (ppm) with spectra referenced to the residual solvent peaks. Coupling constants (*J*) are reported in Hz and are reported as an average. Multiplicities are abbreviated as: s (singlet), d (doublet), t (triplet), q (quartet), qn (quintet), m (multiplet), br (broad), app (apparent) or combinations thereof. Assignments of ^{1}H-NMR and ^{13}C-NMR signals were made where possible, using COSY, HSQC and HMBC experiments. ^{1}H-NMR yields used 1,3,5-trimethoxybenzene as the internal standard.

Melting points were determined from a recrystallized material using Bibby Stuart SMP10 apparatus and are uncorrected.

Infra-red spectra were recorded in the range 4000-650 cm^{-1} on a Perkin Elmer Spectrum either as neat films or solids compressed onto a diamond window and are reported to the nearest 1 cm^{-1}.

Mass spectra were determined by the University of Bristol mass spectrometry service by electrospray ionisation (ESI) mode or electron ionisation (EI) mode. High resolution mass spectra (HRMS) were obtained on a Bruker Daltronics MicroTOF II (ESI) instrument. Molecular ion peaks are reported as $[M+H]^+$ or $[M+Na]^+$.

X-ray diffraction experiments on **6cd** were carried out at 100(2) K on a Bruker APEX II diffractometer using Mo-K_α radiation ($\lambda = 0.71073$ Å). Intensities were integrated in SAINT[3] and absorption corrections based on equivalent reflections were applied using SADABS.[4] The structure was solved using ShelXT[5] and refined by full matrix least squares against F^2 in ShelXL[6,7] using Olex2.[8] All of the non-hydrogen atoms were refined anisotropically. While all of the hydrogen atoms were located geometrically and refined using a riding model, apart from the N-H protons which were located in the difference map and refined freely. Crystal structure and refinement data are given in Table S3. Crystallographic data for compounds **6cd** have been deposited with the Cambridge Crystallographic Data Centre as supplementary publication CCDC 2047771. Copies of the data can be obtained free of charge on application to CCDC, 12 Union Road, Cambridge CB2 1EZ, UK [fax(+44) 1223 336033, e-mail: deposit@ccdc.cam.ac.uk].

Experimental Procedures

General Procedures

The ketone and amide aziridine systems **2b** and **2c** underwent the cascade in refluxing THF. The ^{t}Bu ester aziridine systems **2a** required refluxing dioxane for the reaction to reach completion. The unactivated allyl acetate system, product **6aa**, required this for the IMDA, the others, **6ab-6ae**, for the Tsuji-Trost reaction.

General Procedure A: Ring-opening/Tsuji-Trost/Diels Alder Sequence in THF

$Pd(OAc)_2$ (0.1 equiv.), PPh_3 (0.42 equiv.) and K_2CO_3 (1.3 equiv.) were stirred in degassed THF (0.03 M) for 10 min. The appropriate aziridine (1 equiv.) in THF (0.01 M) followed by the desired allyl acetate (1.2 equiv.) in THF (0.01 M) were added to the stirred solution and the reaction was heated under reflux for 16 h. The reaction mixture was cooled to RT, filtered, concentrated *in vacuo* and loaded directly on a prepacked SiO_2 column for purification.

General Procedure B: Ring-opening/Tsuji-Trost/Diels Alder Sequence in Dioxane

$Pd(OAc)_2$ (0.1 equiv.), PPh_3 (0.42 equiv.) and K_2CO_3 (1.3 equiv.) were stirred in degassed dioxane, (0.03 M) for 10 min. The appropriate aziridine (1 equiv.) in dioxane (0.01 M) was added and the reaction was heated under reflux for 1.5 h. The appropriate allyl acetate (1.2 equiv.) in dioxane (0.01 M) was added and the reaction was heated under reflux for a further 16 h. The reaction mixture was cooled to RT, filtered, concentrated *in vacuo* and loaded directly on a prepacked SiO_2 column for purification.

The photochemically derived, tricyclic aziridine substrates **2a-c** were synthesised as previously described.[1,2]

Additive Screen

Table S1. Effect of catalyst and additives on the ring opening/elimination sequence

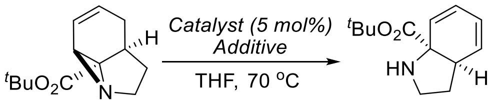

2a → **13**

Entry	Catalyst	Additive[a]	Conversion (%)[b]
1	$Pd(OAc)_2/PPh_3$	None	100
2	$Pd(OAc)_2/PPh_3$	K_2CO_3	100
3	$Pd(PPh_3)_4$	None	0
4	$Pd(PPh_3)_4$	AcOH	100
5	$Pd(PPh_3)_4$	$PPh_3(O)$	0
6	$Pd(PPh_3)_4$	Ac_2O	100[c]
7	$Pd(PPh_3)_4$	CsOAc	29
8	$Pd(PPh_3)_4$	Bu_4NOAc	0
9	$Pd_2(dba)_3/PPh_3$	None	0
10	$Pd_2(dba)_3/PPh_3$	$PPh_3(O)$	<5
11	$Pd_2(dba)_3/PPh_3$	AcOH	100

[a]Added in an equimolar quantity to substrate; [b]Determined by ^{1}H NMR after 45 min at 70 °C; [c]A mixture of **13** and the corresponding acetamide was formed.

Preliminary experiments were undertaken to gain some insight into the mechanism of the key diene formation stage, using aziridine **2a**. With the original catalyst, diene formation is rapid, both in the presence or absence of potassium carbonate (Entries 1 & 2). Use of $Pd(PPh_3)_4$ led to no detectable product within the same time period (Entry 3). Simple addition of AcOH led to rapid reaction (Entry 4), whereas the presence of $Ph_3P(O)$ did not (Entry 5). To explore this further, additional additives were investigated using $Pd(PPh_3)_4$. Ac_2O reaction was complete after 45 min (Entry 6), CsOAc led to a slower conversion (29%, Entry 7), and acetate (Bu_4NOAc) proved ineffective (Entry 8). Similar results were obtained using the $Pd_2(dba)_3/PPh_3$ catalytic system, where using alone led to no observed reaction (Entry 9), addition of $Ph_3P(O)$ again failed to effect any reaction (Entry 10) and addition of AcOH led to rapid reaction. These results are consistent with *N*-activation being key to the observed reactivity, with this being achieved by protonation (AcOH), reaction with an electrophile (Ac_2O) or complexation to a coordinating metal.

Eyring Data

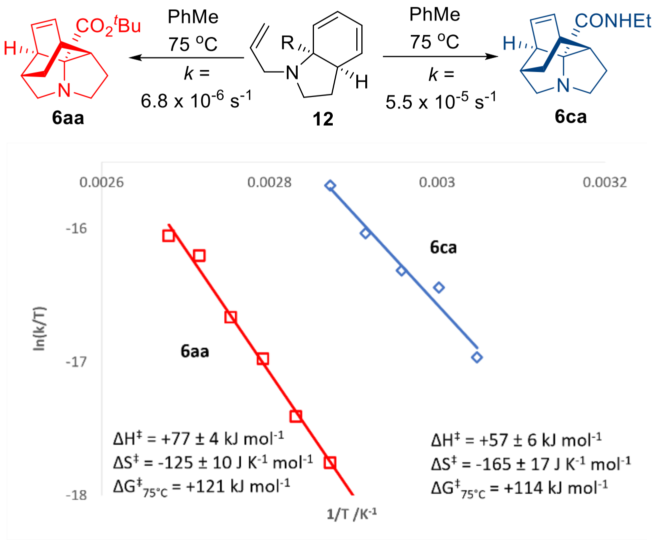

Figure 1. Eyring plots and thermodynamic parameters for the Diels-Alder cyclisation to form **6aa** and **6ca**.

Table S2. Rate constants at different temperatures for substrates **6aa** and **6ca**.

Entry	Temperature /°C	*k* /s^{-1} **6aa**	*k* /s^{-1} **6ca**
1	55	-	$1.41 \pm 0.01 \times 10^{-5}$
2	60	-	$2.41 \pm 0.01 \times 10^{-5}$
3	65	-	$2.78 \pm 0.03 \times 10^{-5}$
4	70	-	$3.73 \pm 0.07 \times 10^{-5}$
5	75	$6.78 \pm 0.09 \times 10^{-6}$	$5.5 \pm 0.1 \times 10^{-5}$
6	80	$9.72 \pm 0.03 \times 10^{-6}$	-
7	85	$1.52 \pm 0.01 \times 10^{-5}$	-
8	90	$2.10 \pm 0.01 \times 10^{-5}$	-
9	95	$3.38 \pm 0.02 \times 10^{-5}$	-
10	100	$3.98 \pm 0.03 \times 10^{-5}$	-

General Considerations for Kinetic Experiments

Rate constants were obtained using ^{1}H-NMR spectroscopy, acquired on a 500 MHz Varian spectrometer. A solution of substrate and standard, 1,3,5-trimethoxybenzene, were dissolved in deuterated PhMe (0.7 mL) and a RT ^{1}H-NMR was obtained. The instrument was then heated to the required temperature, and another spectrum was obtained. Spectra were obtained every 20 minutes for between 12 to 20 hours and the ^{1}H-NMR array data was processed using MestReNova. Errors were calculated in Microsoft Excel via a least squares analysis.

Representative Kinetic Experiment

A standard solution of allylated diene **6aa** (10.4 mg, 0.04 mmol), 1,3,5-trimethoxybenzene (1 mg, 0.006 mmol) in PhMe (0.7 mL) was added into an NMR tube, which was manually loaded into the NMR spectrometer with the probe temperature set at 25 °C. A spectrum was obtained at this temperature, then the probe was heated to 85 °C with the sample loaded within the magnet. The time taken to reach this temperature was recorded (generally ~15 mins for the instrument to reach temperature), and then an array of experiments was started, without further tuning or shimming, collecting spectra every 20 mins for 20 hours.

Comparison of Literature Values

Enthalpies of activation in general are significantly lower than others reported, for instance our systems **6aa** ($\Delta H^{\ddagger}$ =18.4 kcal mol^{-1}) and **6ca** ($\Delta H^{\ddagger}$ =13.6 kcal mol^{-1}) compared with the similarly unactivated dienophile, and cyclic diene component, with a of $\Delta H^{\ddagger}$ = 22.5 kcal mol^{-1} obtained from an Eyring plot.[9] Experimental values are also known for pentadienylacrylamides (i.e. activated dienophiles), where $\Delta H^{\ddagger}$ = 20.4 kcal mol^{-1},[10] a difference of 2 kcal mol^{-1} and 6.8 kcal mol^{-1} increase compared to our systems. Computed $\Delta H^{\ddagger}$ values for IMDA reactions involving activated dienophile cyclopentenone (19.7 kcal mol^{-1}) and cyclohexanone systems (21.5 kcal mol^{-1}) are also consistently larger, with only cyclobutene (15.6 kcal mol^{-1}) systems showing comparable reactivity.[11] Computed values for substituted vinylcyclohexadienes are also available, which are in the range of

21.6 kcal mol^{-1} to 31.5 kcal mol^{-1} at 25 °C for activated dienophiles, rising as high as 37.1 kcal mol^{-1} for those lacking an activating group.[1]

Synthesis of Dienes

(±)-*tert*-butyl (3a*S*,7a*R*)-1-(2-methylallyl)-1,2,3,3a-tetrahydro-7a*H*-indole-7a-carboxylate 9

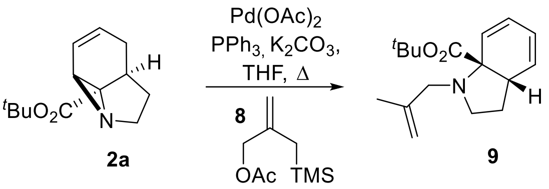

To a dried Schlenk tube under nitrogen were added palladium(II) acetate (6.4 mg, 0.029 mmol), triphenylphosphine (30 mg, 0.11 mmol), potassium carbonate (48 mg, 0.35 mmol) and anhydrous THF (3 mL), the mixture stirred for 5 min and a solution of substrate (60 mg, 0.27 mmol) and 2-(acetoxymethyl)allyltrimethylsilane (60 mg, 0.32 mmol) in anhydrous THF (1 mL) was added. The reaction was degassed by sparging with nitrogen and heated to 70 °C. After 14 h the reaction was cooled to rt and evaporated. Purification by silica gel chromatography (EtOAc/petrol, 2:98 to 1:4 as eluent) afforded the title compound (26 mg, 35%) as a clear oil. δ_H (400 MHz, $CDCl_3$) 1.44 (9H, s, *Me*3C), 1.60 (1H, dq, J 11.8, 7.4 Hz, NCH_2CH*H*), 1.66 (3H, s, Me), 2.28 (1H, dddd, J 11.4, 8.9, 6.8, 4.3 Hz, NCH_2C*H*H), 2.45 – 2.58 (2H, m, NC*H*2CH_2), 3.12 (1H, d, J 13.3, allylic CH*H*), 3.16 – 3.23 (2H, m, allylic C*H*H and CH), 4.73 (1H, ddd, J = 2.4, 1.6, 0.8 Hz, =CH*H*), 4.84 (1H, dd, J = 2.3, 1.2 Hz, =C*H*H), 5.68 (1H, ddt, J = 9.6, 4.1, 1.1 Hz, =C*H*-CH), 5.84 – 5.77 (2H, m, =C*H*-Cq and =C*H*) and 5.95 (1H, ddd, J = 9.9, 5.2, 0.8 Hz, =C*H*); δ_C (101 MHz, $CDCl_3$) 20.3 (Me), 28.2 (*Me*3C), 32.7 (NCH_2*C*H2), 42.0 (CH), 47.1 (NCH_2), 56.1 (allylic CH_2), 67.3 (*C*q-CO_2tBu), 81.0 (Me_3*C*O), 111.3 (=CH2), 119.6 (=CH), 122.1 (=CH), 123.7 (=CH), 129.8 (=*C*H-CH), 144.4 (*C*=CH_2) and 173.5 (CO_2tBu); HRMS (ESI$^+$) m/z: [M+H]$^+$ Calcd for $C_{17}H_{26}NO_2$ 276.1965; found 276.1958.

(±)-*tert*-Butyl (3a*S*,7a*R*)-1-allyl-1,2,3,3a-tetrahydro-7a*H*-indole-7a-carboxylate 12a

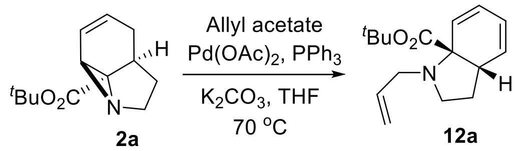

To a dried Schlenk tube under nitrogen were added palladium(II) acetate (6.4 mg, 0.029 mmol), triphenylphosphine (30 mg, 0.11 mmol), potassium carbonate (48 mg, 0.35 mmol) and anhydrous THF (3 mL), the mixture stirred for 5 min and a solution of aziridine **2a** (60 mg, 0.27 mmol) in anhydrous THF (1 mL) followed by allyl acetate (0.035 mL, 0.32 mmol) was added. The reaction was degassed by sparging with nitrogen and heated to 70 °C. After 14 h the reaction was cooled to rt and evaporated. Purification by silica gel chromatography (EtOAc/petrol, 2:98 to 1:9 as eluent) afforded the title compound (62 mg, 87%) as a clear oil. ν_{max} /cm^{-1} (film) 2974, 1718, 1543, 1367 and 1250; δ_H (400 MHz, $CDCl_3$) 1.44 (9H, s, *Me*3C), 1.61 (1H, dq, J 12.1, 7.7 Hz, NCH_2CH*H*), 2.31 (1H, dddd, J 11.9, 9.2, 6.9, 3.6 Hz, NCH_2C*H*H), 2.53 (1H, td, J = 8.3, 6.9 Hz, NCH*H*CH_2), 2.66 (1H, td, J 8.3, 3.6, NC*H*HCH_2), 3.13 – 3.22 (2H, m, allylic CH*H* and C*H*), 3.38 (1H, ddt, J 13.4, 5.6, 1.6 Hz, allylic C*H*H), 5.02 (1H, ddt, J 10.0, 2.2, 1.2 Hz, CH=CH*H*), 5.14 (1H, dq, J 17.1, 1.7 Hz, CH=C*H*H), 5.70 (1H, ddt, J 9.6, 4.2, 1.0 Hz, =C*H*-CH), 5.77 – 5.90 (3H, m, C*H*=CH_2, =C*H* and =C*H*) and 5.96 (1H, ddd, J 9.9, 5.4, 0.9 Hz, =CH); δ_C (101 MHz, $CDCl_3$) 28.2 (*Me*3C), 32.7 (NCH_2*C*H2), 41.9 (CH), 47.2 (NCH_2), 52.8 (allylic CH_2), 67.4 (*C*q-CO_2tBu), 81.3 (Me_3*C*O), 116.1 (=CH_2), 119.4 (=CH), 122.2 (=CH), 123.6 (=CH), 129.7 (=*C*H-CH), 137.1 (*C*H=CH_2) and 173.2 (CO_2tBu); HRMS (ESI$^+$) m/z: [M+H]$^+$ Calcd for $C_{16}H_{24}NO_2$, 262.1801; Found 262.1802.

(±)-1-((3a*R*,7a*S*)-1-allyl-1,2,3,3a-tetrahydro-7a*H*-indol-7a-yl)ethan-1-one 12b

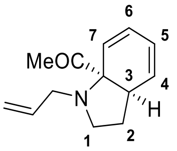

12b

Pd(OAc)2 (7 mg, 0.03 mmol), PPh_3 (29 mg, 0.11 mmol) and K_2CO_3 (48 mg, 0.35 mmol) were stirred in THF (2.5 mL) for 10 min. (±)-1-((3^1*R*,3a*S*,6a*S*)-1,3a,6,6a-tetrahydroazirino[2,3,1-*hi*]indol-3^1(2*H*)-yl)ethan-1-one **2b** (44 mg, 0.27 mmol) in THF (2.5 mL) and allyl acetate (0.034 mL, 0.33 mmol) were added to the stirred solution and the reaction was heated to 30 °C for 5 h, at which point the reaction mixture was filtered and concentrated *in vacuo*.. The crude material was purified by SiO_2 flash chromatography, eluting with 0-50% EtOAc/petrol, to give a yellow oil (17 mg, 56% NMR, 31% isolated). ^{1}H-NMR (400 MHz, $CDCl_3$) δ 6.17 (ddd, 1H, *J* = 10.1, 5.3, 0.9 Hz, 7-CH), 5.90-5.81 (m, 2H, C<u>H</u>CH_2 and 5-CH), 5.75 (ddt, 1H, *J* = 9.6, 4.9,

1.0 Hz, 4-CH), 5.65 (dt, 1H, *J* = 10.0, 0.8 Hz, 6-CH), 5.18 (dq, 1H, *J* = 16.9, 1.8 Hz, CHCHH), 5.07 (dq, 1H, *J* = 10.0, 1.4 Hz, CHCHH) 3.36 (ddt, 1H, *J* = 13.7, 5.6, 1.6 Hz, NCHH), 3.06 (ttt, 1H, *J* = 6.8, 1.2 Hz, NCHH), 3.03-2.96 (m, 2H, 1-CH and 3-CH), 2.41 (app q, 1H, 1-CH), 2.28-2.23 (m, 1H, 2-CH) superimposed on 2.22 (s, 3H, CH_3), 1.70-1.62 (m, 1H, 2-CH); ^{13}C-NMR (125 MHz, $CDCl_3$) δ 211.0 (CO), 136.6 (CHCH$_2$ or 5-CH), 129.8 (4-CH), 125.9 (7-CH), 122.1 (6-CH), 120.8 (CHCH$_2$ or 5-CH), 116.3 (CHCH$_2$), 73.1 (C), 53.0 (NCH$_2$), 50.4 (1-CH$_2$), 41.4 (3-CH), 33.3 (2-CH$_2$), 25.3 (CH_3); υ_{max}/cm^{-1} 2937.89, 2866.36, 1702.74, 1349.76, 1205.30, 1152.35, 1176.95, 1051.29, 713.52, 691.13, HRMS (ESI$^+$) m/z: [M+H]$^+$ Calcd for $C_{13}H_{17}NO$ 204.1388; Found 204.1382.

(±)-(3a*R*,7a*S*)-1-allyl-*N*-ethyl-1,2,3,3a-tetrahydro-7a*H*-indole-7a-carboxamide 12c

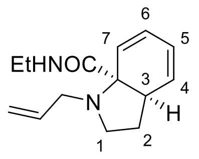

12c

$Pd(OAc)_2$ (7 mg, 0.03 mmol), PPh_3 (29 mg, 0.11 mmol) and K_2CO_3 (48 mg, 0.35 mmol) were stirred in THF (2.5 mL) for 10 min. (±)-(3^1*R*,3a*S*,6a*S*)-*N*-ethyl-1,3a,6,6a-tetrahydroazirino[2,3,1-*hi*]indol-3^1(2*H*)-carboxamide **2c** (52 mg, 0.27 mmol) in THF (2.5 mL) and allyl acetate (0.034 mL, 0.33 mmol) were added to the stirred solution and the reaction was heated to 70 °C or 2 h, at which point the reaction mixture was filtered and concentrated *in vacuo*. The crude material was purified by SiO_2 flash chromatography, eluting with 5-60% EtOAc/petrol to give a yellow oil (21 mg, 60% NMR, 33% isolated). ^{1}H-NMR (400 MHz, $CDCl_3$) δ 7.66 (br s, 1H, NH), 6.17 (dd, 1H, *J* = 9.6, 5.7 Hz, 7-CH), 5.90-5.80 (m, 2H, CHCH$_2$ and 5-CH), 5.59 (dd, 1H, *J* = 9.7, 3.9 Hz, 4-CH), 5.51 (d, 1H, *J* = 9.7 Hz, 6-CH), 5.18 (dq, 1H, *J* = 17.1, 1.7 Hz, CHCHH), 5.08 (br d, 1H, *J* = 10.3 Hz, CHCHH), 3.35 (ddt, 1H, *J* = 13.6, 5.5, 1.7 Hz, NCHH), 3.31-3.20 (m, 3H, 3-CH and CONHCH$_2$), 3.07-3.02 (m, 1H, 1-CH), 2.94 (br dd, 1H, *J* = 14.0, 7.2 Hz, NCHH), 2.46-2.39 (m, 1H, 1-CH), 2.18-2.08 (m, 1H, 2-CH), 1.70-1.67 (m, 1H, 2-CH), 1.12 (t, 3H, *J* = 7.2 Hz, CH_3); ^{13}C-NMR (125 MHz, $CDCl_3$) δ 175.7 (CO), 136.8 (CHCH$_2$ or 5-CH), 130.0 (4-CH), 126.2 (7-CH), 122.0 (6-CH), 121.4 (CHCH$_2$ or 5-CH), 116.4 (CHCH$_2$), 69.1 (C), 53.2 (NCH$_2$), 51.4 (1-CH$_2$), 43.4 (CONHCH$_2$), 34.0 (3-CH), 32.1 (2-CH$_2$), 14.9 (CH_3); υ_{max}/cm^{-1} 2932.65, 2869.95, 1657.80, 1504.88, 1449.25, 1150.03, 1106.86, 1050.81, 914.31, 691.11; HRMS (ESI$^+$) m/z: [M+H]$^+$ Calcd for $C_{14}H_{20}N_2O$ 233.1654; Found 263.1639.

(±)-*tert*-Butyl (3a*S*,7a*R*)-1,2,3,3a-tetrahydro-7a*H*-indole-7a-carboxylate 13a

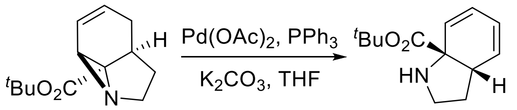

2a **13a**

To a dried Schlenk tube under nitrogen were added $Pd(OAc)_2$ (18 mg, 0.080 mmol), triphenylphosphine (84 mg, 0.32 mmol) and dry THF (2 mL), the mixture stirred for 1 min, a solution of aziridine **2a** (180 mg, 0.81 mmol) in dry THF (6 mL) was added, the mixture sparged with nitrogen and the mixture heated to 70 °C with stirring. After 14 h the reaction was cooled and evaporated. Purification by silica gel chromatography (EtOAc/petrol, 1:9 to 1:1 as eluent) afforded the title compound (149 mg, 83%) as a light-yellow oil. ν_{max} /cm^{-1} (film) 2974, 1719, 1454, 1393, 1367 and 1253; δ_H (500 MHz, $CDCl_3$) 1.47 (9H, s, *Me*$_3$C), 1.66 (1H, dtd, J 12.0, 8.2, 7.3, NCH$_2$CH*H*), 2.27 (1H, dddd, J 11.9, 8.6, 6.5, 4.2, NCH$_2$C*H*H), 2.73 (1H, ddd, J 9.7, 8.3, 6.5, NCH*H*), 2.79 (1H, dddd, J 9.7, 7.1, 4.1, 0.6, NC*H*H), 3.14 (1H, tdd, J 8.1, 4.2, 1.8, CH), 5.62 (1H, dt, J 9.6, 1.0, =C*H*-C$_q$), 5.79 (1H, ddt, J 9.6, 4.2, 1.1, CH), 5.87 (1H, dddd, J 9.6, 5.5, 1.9, 0.8, CH) and 5.95 – 6.00 (1H, dd, J 9.6, 5.4, CH); δ_C (126 MHz, $CDCl_3$) 27.9 (*Me*$_3$C), 35.1 (NCH$_2$*C*H$_2$), 40.9 (CH), 42.0 (NCH$_2$), 66.2 (*C*$_q$-CO$_2$^{t}Bu), 81.4 (Me$_3$*C*O), 119.6 (CH), 122.5 (CH), 126.2 (CH), 129.5 (CH) and 174.6 (*C*O$_2$^{t}Bu); HRMS (ESI$^+$) m/z: [M+H]$^+$ Calcd for $C_{13}H_{20}NO_2$ 222.1489; Found 222.1483.

(±)-1-((3a*S*,7a*R*)-1,2,3,3a-tetrahydro-7a*H*-indol-7a-yl)ethan-1-one 13b

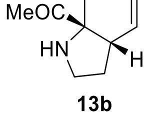

13b

To a dried Schlenk tube under nitrogen were added $Pd(OAc)_2$ (6.4 mg, 0.029 mmol), triphenylphosphine (30 mg, 0.11 mmol) and dry THF (2 mL), the mixture stirred for 5 min, a solution of aziridine **2b** (45 mg, 0.28 mmol) in dry THF (2 mL) was added, the mixture sparged with nitrogen and the mixture heated to 70 °C with stirring. After 19 h the reaction was cooled to rt and evaporated. Purification by silica gel chromatography (EtOAc/petrol, 9:1 to 1:0 as eluent) afforded the title compound (37 mg, 82%) as a yellow oil. ν_{max} /cm^{-1} (film) 2964, 1707, 1407, 1353 and 1170; δ_H (500 MHz, $CDCl_3$) 1.68 (1H, dtd, J 11.8,

9.2, 6.8, NCH_2CH*H*), 2.21 (1H, dddd, J 11.7, 7.9, 5.9, 3.5, NCH_2C*H*H), 2.27 (3H, s, Me), 2.61 (1H, td, J 9.5, 5.8, NCH*H*), 2.74 – 2.86 (2H, m, NC*H*H and NC*H*), 5.43 (1 H, dd, J 9.7, 1.0, =C*H*-C_q), 5.87 – 5.98 (2H, m, C*H*=C*H*-CH) and 6.03 (1H, ddd, J 9.6, 5.2, 1.0, C*H*=CH-C_q); δ_C (126 MHz, $CDCl_3$) 24.9 (CO*Me*), 36.2 (NCH_2*C*H_2), 40.6 (CH), 41.9 (N*C*H2), 71.3 (*C*q-COMe), 120.2 (CH=*C*H-), 123.1 (*C*H=CH-Cq), 126.0 (=*C*H-Cq), 129.0 (*C*H=CH) and 208.6 (*C*OMe); HRMS (ESI^+) m/z: $[M+H]^+$ Calcd for $C_{10}H_{14}NO$ 164.1070; Found 164.1075.

(±)-(3a*S*,7a*R*)-*N*-Ethyl-1,2,3,3a-tetrahydro-7a*H*-indole-7a-carboxamide 13c

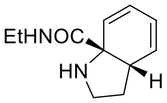

13c

To a dried Schlenk tube under nitrogen were added $Pd(OAc)_2$ (5.5 mg, 0.025 mmol), triphenylphosphine (26 mg, 0.10 mmol), potassium carbonate (42 mg, 0.35 mmol) and dry THF (2 mL), the mixture stirred for 5 min, and a solution of aziridine **2c** (45 mg, 0.28 mmol) in dry THF (2 mL) was added, the mixture sparged with nitrogen and the mixture heated to 70 °C with stirring. After 18 h the reaction was cooled and evaporated to give a yellow solid. Purification by silica gel chromatography (EtOH/aq. NH_3 in CH_2Cl_2, 1% to 5% as eluent) afforded the title compound (20 mg, 44%) as a yellow oil. ν_{max} /cm^{-1} (film) 3326 (br), 2968, 1733, 1659, 1510, 1449, 1374 and 1241; δ_H (500 MHz, $CDCl_3$) 1.15 (3H, t, J 7.3, *Me*CH_2N), 1.63 (1H, dq, J 12.2, 7.1, NCH_2CH*H*), 2.23 (1H, dddd, J 12.3, 8.1, 6.9, 5.5, NCH_2C*H*H), 2.61 (1H, dt, J 10.7, 7.1, NCH*H*CH_2), 2.81 (1H, dddd, J 10.7, 7.3, 5.5, 0.8, NC*H*HCH_2), 3.24 – 3.33 (3H, m, NC*H*$_2$Me and C*H*), 5.56 (1H, dd, J 9.5, 0.9, =C*H*-C_q), 5.79 (1H, dd, J 9.6, 4.0, =C*H*-CH), 5.90 (1H, dddd, J 9.6, 5.5, 2.1, 0.8, C*H*=CH-CH), 6.05 (1H, ddt, J 9.5, 5.5, 0.7, C*H*=CH-C_q) and 7.64 – 7.74 (1H, m, NH); δ_C (126 MHz, $CDCl_3$) 14.9 (Me), 34.2 (N*C*H_2Me), 35.6 (NCH_2*C*H_2), 41.7 (CH), 42.9 (N*C*H_2CH_2), 67.4 (*C*$_q$-CONHEt), 119.9 (*C*H=CH-CH), 124.2 (*C*H=CH-C_q), 125.8 (=*C*H-C_q), 130.6 (=*C*H-CH) and 175.3 (*C*ONHEt); HRMS (ESI^+) m/z: $[M+H]^+$ Calcd for $C_{11}H_{17}N_2O$ 193.1335; Found 193.1341.

Synthesis of Tethers

Methyl 2-(acetoxymethyl) acrylate 5b

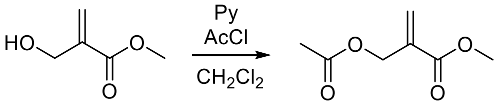

5b

To a stirred solution of methyl 2-(hydroxymethyl) acrylate (0.89 mL, 8.6 mmol), dry CH_2Cl_2 (18 mL) and pyridine at -10°C was added acetyl chloride (0.80 mL, 11 mmol) dropwise. The resulting solution was warmed to rt and stirred for 2 h. After this time the reaction was cooled to 0°C and water (30 mL) was charged. The aqueous layer was extracted with EtOAc (30 mL × 3) and the resulting organics washed with water (30 mL), sat. NH4Cl (30 mL) and brine (30 mL). The combined organics were dried over magnesium sulfate, filtered and concentrated *in vacuo*. The crude product was purified over silica gel (10 - 30% EtOAc in petrol) to afford **5b** (1.1 g, 81%) as a pale-yellow oil. vmax/cm^{-1} 2951 (br, m, C-H), 1740 (m, C=O), 1675 (m, C=O), 1368 (m), 730 (m, =C-H); 1H NMR (400 MHz, CDCl3) δH 6.35 (q, *J* = 1.0 Hz, 1H, CH), 5.83 (d, *J* = 1.4 Hz, 1H, CH), 4.79 (s, 2H, CH_2), 3.77 (s, 3H, CH_3), 2.08 (s, 3H, CH_3). ^{1}H-NMR data agrees with the literature.[13]

3-(Hydroxymethyl)but-3-en-2-one S1

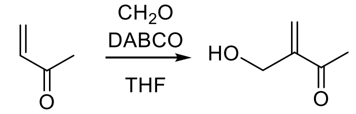

S1

To a stirred solution of methyl vinyl ketone (4.95 mL, 59 mmol), 37% aq. formaldehyde (4.5 mL, 71 mmol) and THF (30 mL) at 0 °C was added DABCO (0.68 g, 5.9 mmol). The resulting solution was allowed to warm to rt and stirred for 20 h. Brine (30 mL) was added, the layers separated, and the aqueous layer extracted with Et_2O (3 × 30 mL). The combined organics were dried over magnesium sulfate, filtered and concentrated *in vacuo*. The crude product was purified over silica gel (0 - 30% EtOAc in petrol) to afford **S1** (2.6 g, 44%) as a colourless oil. ν_{max}/cm^{-1} 3408 (br, m, O-H), 2926 (w, C-H), 1666 (s, C=O), 1367 (m), 1051 (m); ^{1}H NMR (400 MHz, CDCl3) δH 6.11 50 (s, 1H, CH), 6.03 (t, *J* = 1.4 Hz, 1H, CH), 4.30 (d, *J* = 5.9 Hz, 2H, CH_2), 2.36 (s, 3H, CH_3). ^{1}H-NMR data agrees with the literature.[14]

2-Methylene-3-oxobutyl acetate 5c

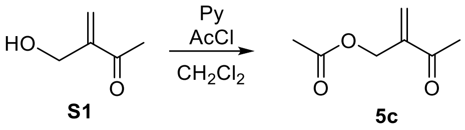

To a stirred solution of 3-(hydroxymethyl)but-3-en-2-one **S1** (2.6 g, 37 mmol), pyridine (3.9 mL, 48 mmol) and dry CH_2Cl_2 (65 mL) at 0°C was added acetyl chloride (3.4 mL, 48 mmol) dropwise. The resulting solution was allowed to warm to rt and stirred for 20 h. The reaction was quenched with 1M HCl (17 mL), the layers separated, and the aqueous layer extracted with CH_2Cl_2 (1 × 30 mL). The combined organics were washed with $NaHCO_3$ (13 mL), dried over magnesium sulfate, filtered and concentrated *in vacuo*. The crude product was purified over silica gel (0 - 30% EtOAc in petrol) to afford **5c** (1.8 g, 34%) as a colourless oil. ν_{max}/cm^{-1} 2970 (br, w, C-H), 1741 (s, C=O), 1675 (s, C=O), 1369 (m), 1227 (s); ^{1}H NMR (400 MHz, CDCl3) δH 6.18 (d, *J* = 1.0 Hz, 1H, CH), 6.00 (t, *J* = 1.5 Hz, 1H, CH), 4.78 (t, *J* = 1.5, 2H, CH_2), 2.35 (s, 3H, CH_3), 2.08 (s, 3H, CH_3). ^{1}H-NMR data agrees with the literature.[15]

2-(Hydroxymethyl)acrylonitrile S2

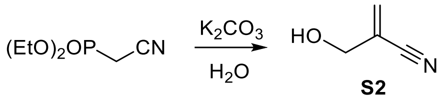

To a stirred solution of 37% aq. formaldehyde (5.5 mL, 74 mmol) and diethyl cyanomethylphosphonoacetate (3 mL, 19 mmol) was added saturated K_2CO_3 (4.0 mL, 32 mmol) dropwise over 30 min. The resulting solution was stirred at rt for 1.5 h. The reaction was quenched with NH_4Cl (6 mL), the layers separated, and the aqueous layer extracted with Et_2O (3 × 5 mL). The combined organics were dried over magnesium sulfate, filtered and concentrated *in vacuo*. The crude product was purified over silica gel (10 - 30% EtOAc in petrol) to afford **35** (0.59 g, 38%) as a colourless oil. ν_{max}/cm^{-1} 3415 (br, m, O-H), 2988 (m, C-H), 2229 (m, C≡N), 1408 (m), 1044 (s, C-O), 948 (m, =C-H); ^{1}H-NMR (400 MHz, $CDCl_3$) δH 6.04 (t, *J* = 1.8 Hz, 1H, CH), 6.01 (t, *J* = 1.4 Hz, 1H, CH), 4.23 (dt, *J* = 6.2, 1.6 Hz, 2H, CH_2). ^{1}H-NMR data agrees with the literature.[16]

2-Cyanoallyl acetate 5d

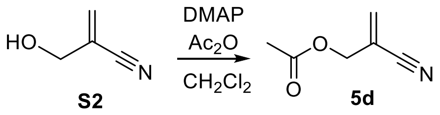

To a stirred solution of 2-(hydroxymethyl)acrylonitrile **S2** (0.59 g, 7.1 mmol) in dry CH_2Cl_2 (12 mL) was added acetic anhydride (2.0 mL, 22 mmol), triethylamine (1.2 mL, 8.5 mmol) and DMAP (26 mg, 0.23 mmol). The resulting solution was stirred at rt for 20 h. The reaction was quenched with MeOH (2 mL) and stirred for 10 mins, followed by sat. aq. $NaHCO_3$ (4 mL) and stirred for a further 10 min. The phases were allowed to settle and separated and the aqueous layer extracted with CH_2Cl_2 (2 × 6 mL). The combined organics were dried over magnesium sulfate, filtered and concentrated to almost dryness *in vacuo* with low temperature and vacuum used to avoid evaporation of the volatile product. The crude product was purified over silica gel (10 - 40% Et_2O in pentane) to afford **5d** (0.58 g, 66%) as a colourless oil. ν_{max}/cm^{-1} 2988 (br, m, C-H), 1744 (m, C=O), 1375 (m), 1215 (s), 1043 (s, C-O); ^{1}H NMR (400 MHz, $CDCl_3$) δ 6.07 (s, 1H, CH), 6.01 (t, *J* = 1.5 Hz, 1H, CH), 4.64 (s, 2H,CH_2), 2.10 (s, 3H, CH_3). ^{1}H-NMR data agrees with the literature.[17]

Cinnamyl acetate 5e

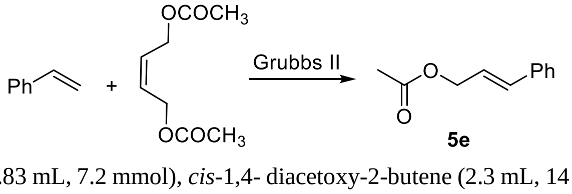

To a stirred solution of styrene (0.83 mL, 7.2 mmol), *cis*-1,4- diacetoxy-2-butene (2.3 mL, 14 mmol) and dry CH_2Cl_2 (20 mL), under a nitrogen atmosphere, was added Grubbs catalyst 2nd generation (75 mg, 0.18 mmol) as a solution in dry CH_2Cl_2 (20 mL). After stirring at rt for 16 h the resulting solution was concentrated *in vacuo* and purified over silica gel (10 - 30% Et_2O in petrol) to afford **5e** (0.80 g, 63%) as a yellow oil. ν_{max}/cm^{-1} 3027 (w, C-H), 1735 (s, C=O), 1226 (s), 1025 (m), 965 (m); ^{1}H-NMR (400 MHz, $CDCl_3$) δ 7.44 – 7.22 (m, 5H, Ph), 6.66 (m, *J* = 5.9 Hz, 1H, CH), 6.29 (dt, *J* = 15.9, 6.5 Hz, 1H, CH), 4.73 (dd, *J* = 6.5, 1.4 Hz, 2H, CH_2), 2.10 (s, 3H, C1-H). ^{1}H-NMR data agrees with the literature.[18]

3-(Hydroxy(phenyl)methyl)but-3-en-2-one S3

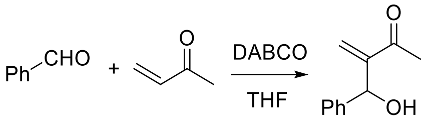

S3

To a stirred solution of methyl vinyl ketone (5.0 mL, 62 mmol) in THF (30 mL) at 0 °C under nitrogen was added benzaldehyde (7.3 mL, 72 mmol) followed by DABCO (675 mg, 6.0 mmol) portionwise. The reaction was allowed to warm to rt, stirred for 16 h and diluted with brine (30 mL). The mixture was extracted with Et_2O (2 × 30 mL) and the combined organic phase dried ($MgSO_4$) and evaporated to give a yellow oil. Purification by silica gel chromatography (EtOAc/petrol, 1:9 to 3:7 as eluent) afforded the title compound (1.48 g, 14%) as a clear oil. ^{1}H-NMR and ^{13}C-NMR data agrees with the literature.[19]

2-Methylene-3-oxo-1-phenylbutyl acetate 5f

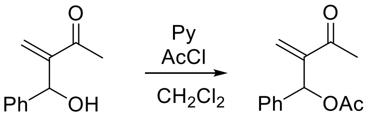

S3 **5f**

To a stirred solution of substrate **S3** (1.47 g, 8.4 mmol) and pyridine (1.6 mL, 20 mmol) in anhydrous CH_2Cl_2 (30 mL) at 0 °C under nitrogen was added acetyl chloride (1.35 mL, 19 mmol) dropwise. The reaction was allowed to warm to rt, stirred for 13 h, diluted with CH_2Cl_2 (20 mL) and washed with 1 M HCl (20 mL) and sat. aq. $NaHCO_3$ (20 mL). Drying ($MgSO_4$) and evaporation gave an orange oil. Purification by silica gel chromatography (EtOAc/petrol, 1:9 to 1:4 as eluent) afforded the title compound (363 mg, 20%) as a clear oil. ^{1}H-NMR data agrees with the literature.[20]

Synthesis of Diels-Alder Products

(±)-*tert*-Butyl (3a*R*,4*S*,4a*S*,7a*R*,8*S*,8a*R*)-1-allyl-5,7-dioxo-2,3,3a,4,4a,5,6,7,7a,8-decahydro-4,8-ethenopyrrolo[3,4-*f*]indole-8a(1*H*)-carboxyalte 14

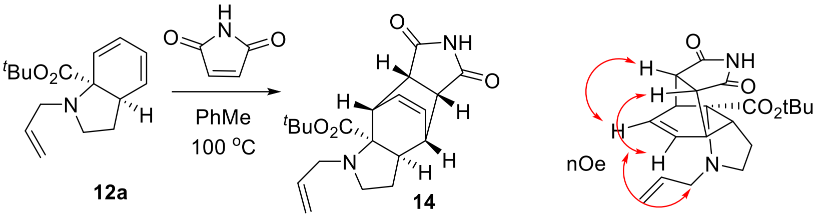

12a **14**

A stirred solution of diene **12a** (29 mg, 0.11 mmol) and maleimide (15 mg, 0.15 mmol) in toluene (5 mL) was heated to 100 °C. After 14 h the reaction was cooled and evaporated. Purification by silica gel chromatography (Et_2O/ CH_2Cl_2, 1:4 to 1:0 then neat EtOAc as eluent) afforded intermolecular Diels-Alder product **14** (34 mg, 77%) and intramolecular Diels-Alder product **15** (2 mg, 7%) as a clear oil. **14**: ν_{max} /cm^{-1} (film) 2974, 1705, 1367, 1353 and 1155; δ_H (500 MHz, $CDCl_3$) 1.37 (1H, ddt, J 12.7, 9.6, 7.6 NCH_2CH*H*), 1.51 (8H, s, *Me*$_3$C), 2.00 (1H, dddd, J 12.0, 9.2, 6.6, 2.4, NCH_2C*H*H), 2.55 (1H, td, J 9.0, 6.7, NCH*H*CH_2), 2.73 (1 H, ddd, J 9.5, 7.0, 2.6, CH), 2.78 – 2.87 (2H, m, NC*H*HCH_2 and NHC(O)C*H*), 2.95 – 3.04 (2H, m, NHC(O)C*H* and allylic CH*H*)), 3.12 (1 H, dtd, J 6.1, 2.8, 1.4, CH), 3.39 (1 H, ddt, J 14.0, 5.3, 1.7, allylic C*H*H), 3.60 (1 H, ddd, J 6.5, 3.1, 1.4, CH), 5.04 (1 H, dd, J 10.1, 1.6, =CH*H*), 5.14 (1 H, dq, J 17.1, 1.7, =C*H*H), 5.75 (1 H, dddd, J 16.9, 10.1, 6.8, 5.2, C*H*=CH_2), 6.12 (1 H, dd, J 8.9, 5.8, CH=C*H*), 6.17 (1 H, ddd, J 7.9, 6.3, 1.4, C*H*=CH) and 8.45 (1H, brs, NH); δ_C (126 MHz, $CDCl_3$) 28.3 (*Me*$_3$C), 28.9 (NCH_2*C*H_2), 36.9 (CH), 37.5 (CH), 41.9 (CH), 45.2 (CH), 47.0 (CH), 51.0 (N*C*H_2CH_2), 52.1 (allylic CH_2), 74.8 (C_q-CO_2tBu), 81.9 (Me_3*C*O), 116.0 (=CH_2), 129.5 (CH=*C*H), 132.9 (*C*H=CH), 136.4 fz(*C*H=CH_2), 171.4 (CO_2tBu), 178.6 (imide *C*=O), 178.8 (imide *C*=O); HRMS (ESI$^+$) m/z: [M+H]$^+$ Calcd for $C_{20}H_{27}N_2O_4$ 359.1965; Found 359.1961. Stereochemistry was proven by nOe interactions between the alkenyl bridge hydrogens (6.12 and 6.17 ppm) and the maleimide CH (2.8 ppm) and NCHH (3.39 ppm) peaks as depicted above. See S32 and S33 for spectra.

(±)-*tert*-Butyl (1*S*, 3^1*R*,5a*R*,6*S*,8a*R*,9*R*)-1,4,5,5a,6,8a-hexahydro-1,6-methanopyrrolo[3,2,1-*hi*]indole-3^1(2*H*)-carboxylate 6aa (15)

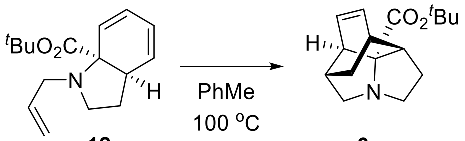

12a **6aa**

A stirred solution of diene (40 mg, 0.11 mmol) in toluene (5 mL) was heated to 100 °C. After 18 h the reaction was cooled and evaporated. Purification by silica gel chromatography (EtOH/NH_3 in CH_2Cl_2, 1% to 8% as eluent) afforded intramolecular Diels-Alder product **15** (37 mg, 93%) as a clear oil. ν_{max} /cm^{-1} (film) 2934, 1721, 1546, 1367 and 1159; ^{1}H-NMR (400 MHz, $CDCl_3$) δ_H (500 MHz, $CDCl_3$) 1.38 – 1.46 (11H, m, C*Me*$_3$ and CHC*H*$_2$CH), 1.56 (1H, dtd, J 12.9, 9.0, 3.9, NCH$_2$CH*H*), 1.87 (1H, dq, J 8.0, 4.1, NCH$_2$C*H*), 2.01 (1H, dtd, J 13.2, 9.4, 2.8, NCH$_2$C*H*H), 2.24 (1H, d, J 11.3, NCH*H*CH), 2.28 – 2.36 (2H, m, NCH$_2$CH$_2$C*H* and NCH$_2$CH$_2$CHC*H*), 3.04 (1H, ddd, J 12.2, 9.1, 2.8, NCH*H*CH$_2$), 3.24 (1H, ddd, J 5.9, 4.3, 1.2 Hz, NCHCHC*H*), 3.39 (1H, dt, J 12.5, 9.1 Hz, NC*H*HCH$_2$), 3.51 (1H, dd, J 11., 4.3, NC*H*HCH), 6.16 (1H, ddd, J 7.9, 6.5, 1.2 Hz, =C*H*-CH-Cq) and 6.45 (1H, ddd, J 8.0, 6.5, 1.3 Hz, =C*H*-CH-CH-Cq); δ_C (126 MHz, $CDCl_3$) 28.0 (*C*Me$_3$), 28.1 (NCH$_2$*C*H$_2$), 31.4 (CH*C*H$_2$CH), 34.4 (NCH$_2$CH$_2$CH*C*H), 34.8 (NCH$_2$*C*H), 44.9 (*C*HCqCO$_2$^{t}Bu), 49.7 (NCH$_2$CH$_2$*C*H), 58.5 (N*C*H$_2$CH$_2$), 64.0 (N*C*H$_2$CH), 80.4 (Me$_3$*C*O), 80.9 (C$_q$-CO$_2$^{t}Bu), 129.2 (=*C*H-CH-Cq)), 137.5 (=*C*H-CHCHCq) and 174.0 (*C*O$_2$^{t}Bu); HRMS (ESI$^+$) 262.1802 ($C_{16}H_{24}NO_2$, [M+H]$^+$, requires 262.1802).; HRMS (ESI$^+$) m/z: [M+H]$^+$ Calcd for $C_{16}H_{24}NO_2$ 262.1802; Found 262.1802.

(±)-*tert*-Butyl (1*S*, 3^1*R*,5a*R*,6*S*,8a*R*,9*R*)-1,4,5,5a,6,8a-hexahydro-1,6-methanopyrrolo[3,2,1-*hi*]indole-3^1(2*H*)-carboxylate 6aa

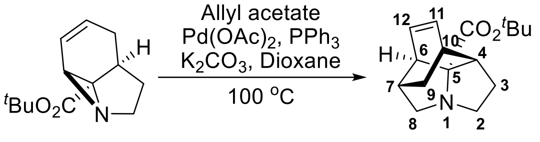

2a **6aa**

According to general procedure B, Pd(OAc)$_2$ (6.1 mg, 0.03 mmol), PPh$_3$ (30 mg, 0.11 mmol) and K_2CO_3 (49 mg, 0.35 mmol) were stirred in dioxane (2.5 mL) for 10 min. (±)-*tert*-Butyl (3^1*R*,3a*S*,6a*S*)-1,3a,6,6a-tetrahydroazirino[2,3,1-*hi*]indol-3^1(2*H*)-carboxylate **2a** (60 mg, 0.27 mmol) in dioxane (1.25 mL) was added to the stirred mixture and the reaction was heated to 100 °C. After 1.5 h allylacetate (81 mg, 0.81 mmol) in dioxane (1.25 mL) was added and the reaction mixture stirred for a further 16 h at the same temperature. The crude material was purified by SiO_2 flash chromatography (80 - 100% Et_2O in petrol+ 0.1% NH_3) to give a pale-yellow oil (58 mg, 82%).

3 mmol scale

According to general procedure B, Pd(OAc)$_2$ (67 mg, 0.3 mmol), PPh$_3$ (330 mg, 1.26 mmol) and K_2CO_3 (539 mg, 3.9 mmol) were stirred in dioxane (50 mL) for 10 min. (±)-*tert*-Butyl (3^1*R*,3a*S*,6a*S*)-1,3a,6,6a-tetrahydroazirino[2,3,1-*hi*]indol-3^1(2*H*)-carboxylate **2a** (664 mg, 3 mmol) in dioxane (2.5 mL) was added to the stirred mixture and the reaction was heated to 100 °C. After 1.5 h allylacetate (360 mg, 3.6 mmol) in dioxane (2.5 mL) was added and the reaction mixture stirred for a further 16 h at the same temperature. The crude material was purified by SiO_2 flash chromatography (80 - 100% Et_2O in petrol+ 0.1% NH_3) to give a pale-yellow oil (733 mg, 93%).

(±)-3^1-(*tert*-Butyl) 1-methyl (1*R*, 3^1*R*,5a*R*,6*S*,8a*R*)-5,5a,6,8a-tetrahydro-1,6-methanopyrrolo [3,2,1-*hi*]indole-1,3^1(2*H*,4*H*)-dicarboxylate 6ab

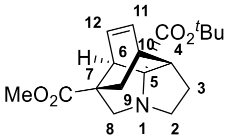

6ab

According to general procedure B, Pd(OAc)$_2$ (6.1 mg, 0.03 mmol), PPh$_3$ (30 mg, 0.11 mmol) and K_2CO_3 (49 mg, 0.35 mmol) were stirred in dioxane (2.5 mL) for 10 min. (±)-*tert*-Butyl (3^1*R*,3a*S*,6a*S*)-1,3a,6,6a-tetrahydroazirino[2,3,1-*hi*]indol-3^1(2*H*)-carboxylate **2a** (60 mg, 0.27 mmol) in dioxane (1.25 mL) was added to the stirred mixture and the reaction was heated to 100 °C. After 1.5 h methyl 2-(acetoxymethyl) acrylate (51 mg, 0.32 mmol) in dioxane (1.25 mL) was added and the reaction mixture stirred for a further 16 h at the same temperature. The crude material was purified by SiO_2 flash chromatography (60 Et_2O in petrol) to give a pale-yellow oil (60 mg, 70%). ν_{max}/cm^{-1} 2972 (br, s, C-H), 1725 (s, C=O), 1236 (m), 1162 (m), 1056 (m); ^{1}H-NMR (400 MHz, $CDCl_3$) δ 6.47 (ddd, *J* = 7.9, 6.6, 1.2 Hz, 1H, 11-CH), 6.15 (ddd, *J* = 7.9, 6.4, 1.2 Hz, 1H, 12-CH), 3.67 (dd, 1H, *J* = 11.2, 1.4 Hz, 8-CH) superimposed on 3.64 (s, 3H, CH_3), 3.59 (dd, 1H, *J* = 6.5, 1.3 Hz, 6-CH), 3.42 (ddd, *J* = 12.6, 9.5, 8.1 Hz, 1H, 2-CH), 3.03 (ddd, *J* = 12.5, 9.2, 3.3 Hz, 1H, 2-CH), 2.47 (d, *J* = 11.2 Hz, 1H, 8-CH) superimposed on 2.45-2.39 (m, 1H, 10-CH), 2.29-2.22 (m, 1H, 4-CH), 2.11-2.01 (m, 1H, 3-CH), 1.80 (dtd, *J* = 13.7, 2.3, 1.5 Hz, 1H, 9-CH), 1.69 (dd, *J* = 13.7, 3.1 Hz, 1H, 9-CH), 1.64-1.53 (m, 1H, 3-CH), 1.42 (s, 9H, C(CH_3)$_3$); ^{13}C-NMR (125 MHz, $CDCl_3$) δ 174.7 (CO), 173.3 (CO), 137.5 (11-CH), 128.5 (12-CH), 81.1 (C), 80.9 (C), 66.8 (8-CH$_2$), 58.7 (2-CH$_2$), 52.1 (CH_3), 50.9 (7-C), 49.6 (4-CH), 47.3 (6-CH), 35.5 (10-CH), 34.1 (9-CH$_2$), 28.5 (3-CH$_2$), 28.1 (C($\underline{C}H_3$)$_3$); HRMS (ESI$^+$) m/z: [M+H]$^+$ Calcd for $C_{18}H_{25}NO_4$ 320.1856; Found 320.1836.

3 mmol scale

According to general procedure B, Pd(OAc)$_2$ (67 mg, 0.3 mmol), PPh$_3$ (330 mg, 1.26 mmol) and K$_2$CO$_3$ (539 mg, 3.9 mmol) were stirred in dioxane (50 mL) for 10 min. (±)-*tert*-Butyl (3^1*R*,3a*S*,6a*S*)-1,3a,6,6a-tetrahydroazirino[2,3,1-*hi*]indol-3^1(2*H*)-carboxylate **2a** (664 mg, 3 mmol) in dioxane (2.5 mL) was added to the stirred mixture and the reaction was heated to 100 °C. After 1.5 h a methyl 2-(acetoxymethyl) acrylate (569 mg, 3.6 mmol) in dioxane (2.5 mL) was added and the reaction mixture stirred for a further 16 h at the same temperature. The crude material was purified by SiO$_2$ flash chromatography (60 Et$_2$O in petrol) to give a pale-yellow oil (762 mg, 79%).

(±)-*tert*-Butyl (1*S*, 3^1*R*,5a*R*,6*S*,8a*R*,9*R*)-1-acetyl-1,4,5,5a,6,8a-hexahydro-1,6-methanopyrrolo [3,2,1-*hi*]indole-3^1(2*H*)-carboxylate 6ac

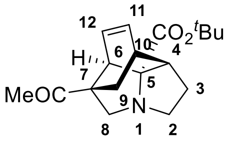

6ac

According to general procedure B, Pd(OAc)$_2$ (6.1 mg, 0.03 mmol), PPh$_3$ (30 mg, 0.11 mmol) and K$_2$CO$_3$ (49 mg, 0.35 mmol) were stirred in dioxane (2.5 mL) for 10 min. (±)-*tert*-Butyl (3^1*R*,3a*S*,6a*S*)-1,3a,6,6a-tetrahydroazirino[2,3,1-*hi*]indol-3^1(2*H*)-carboxylate **2a** (60 mg, 0.27 mmol) in dioxane (1.25 mL) was added to the stirred mixture and the reaction was heated to 100 °C. After 1.5 h 2-methylene-3-oxobutyl acetate (46 mg, 0.32 mmol) in dioxane (1.25 mL) was added and the reaction mixture stirred for a further 16 h at the same temperature. The crude material was purified by SiO$_2$ flash chromatography (80 - 100% Et$_2$O in petrol+ 0.1% NH$_3$) to give a pale-yellow solid (51 mg, 62%). m.p.: 63-66 ˚C; ν_{max}/cm^{-1} 2935 (br, m, C-H), 1722 (s, C=O), 1699 (s, C=O), 1228 (m), 1157 (s); ^{1}H NMR (400 MHz, CDCl$_3$) δ_H 6.45 (ddd, *J* = 7.9, 6.7, 1.2 Hz, 1H, 11-CH), 6.16 (ddd, *J* = 7.9, 6.4, 1.2 Hz, 1H, 12-CH), 3.62 (d, *J* = 11.3 Hz, 1H, 8-CH), 3.54 (dd, *J* = 6.4, 0.8 Hz, 1H, 6-CH), 3.41 (dt, *J* = 12.6, 8.9 Hz, 1H, 2-CH), 3.04 (ddd, *J* = 12.5, 9.2, 3.1 Hz, 1H, 2-CH), 2.47 (ddd, *J* = 6.7, 2.8, 1.2 Hz, 1H, 10-CH), 2.42 (d, *J* = 11.3 Hz, 1H, 8-CH), 2.33 – 2.27 (m, 1H, 4-CH), 2.12 – 2.02 (m, 1H, 3-CH), superimposed on 2.08 (s, 3H, CH$_3$), 1.74 – 1.55 (m, 3H, 3-CH and 9-CH$_2$), 1.43 (s, 9H, C(CH$_3$)$_3$); ^{13}C NMR (101 MHz, CDCl$_3$) δ_C 208.7 (CO), 173.3 (CO), 137.4 (11-CH), 128.6 (12-CH), 81.3 (C), 81.0 (C), 66.1 (8-CH$_2$), 58.6 (2-CH$_2$), 57.8 (7-C), 49.7 (4-CH), 46.6 (6-CH), 35.5 (10-CH), 33.5 (9-CH$_2$), 28.4 (3-CH$_2$), 28.1 (C($\underline{C}$H$_3$)$_3$), 26.8 (CH$_3$); HRMS (ESI$^+$) m/z: [M+H]$^+$ Calcd for C$_{18}$H$_{25}$NO$_3$ 304.1907; Found 304.1897

(±)-*tert*-Butyl (1*R*,3^1*R*,5a*R*,6*S*,8a*R*)-1-cyano-1,4,5,5a,6,8a-hexahydro-1,6-methanopyrrolo[3,2,1-hi]indole-3^1(2*H*)-carboxylate 6ad

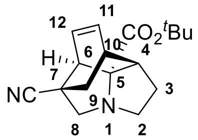

6ad

According to general procedure B, Pd(OAc)$_2$ (6.1 mg, 0.03 mmol), PPh$_3$ (30 mg, 0.11 mmol) and K$_2$CO$_3$ (49 mg, 0.35 mmol) were stirred in dioxane (2.5 mL) for 10 min. (±)-*tert*-Butyl (3^1*R*,3a*S*,6a*S*)-1,3a,6,6a-tetrahydroazirino[2,3,1-*hi*]indol-3^1(2*H*)-carboxylate **2a** (60 mg, 0.27 mmol) in dioxane (1.25 mL) was added to the stirred mixture and the reaction was heated to 100 °C. After 1.5 h 2-cyanoallyl acetate (41 mg, 0.32 mmol) in dioxane (1.25 mL) was added and the reaction mixture stirred for a further 16 h at the same temperature. The crude material was purified by SiO$_2$ flash chromatography (20% 3:1 EtOAc:EtOH + 0.1% NH$_3$ in pentane) to give a pale-yellow solid (59 mg, 76%). m.p.: 74-76 ˚C; ν_{max}/cm^{-1} 2972 (br, s, C-H), 2884 (m, C-H), 2230 (w, C≡N), 1724 (s, C=O), 1252 (m), 1125 (s); ^{1}H NMR (400 MHz, CDCl$_3$) δ_H 6.60 (ddd, *J* = 8.0, 6.7, 1.2 Hz, 1H, 11-CH), 6.22 (ddd, *J* = 7.9, 6.4, 1.2 Hz, 1H, 12-CH), 3.68 (dd, *J* = 11.2, 1.4 Hz, 1H, 8-CH), 3.56 (dd, *J* = 6.4, 1.4 Hz, 1H, 6-CH), 3.42 (ddd, , *J* = 12.7, 9.6, 7.9 Hz, 1H, 2-CH), 3.02 (ddd, *J* = 12.8, 9.3, 3.5 Hz, 1H, 2-CH), 2.62 (d, *J* = 11.4 Hz, 1H, 8-CH), 2.49 – 2.42 (m, 1H, 10-CH), 2.29 – 2.22 (m, 1H, 4-CH), 2.07 (dtd, *J* = 13.1, 9.5, 3.5 Hz, 1H, 3-CH), 1.81 (dd, *J* = 13.7, 3.0 Hz, 1H, 9-CH), 1.72 (dtd, *J* = 13.8, 2.3, 1.3 Hz, 1H, 9-CH), 1.58 – 1.48 (m, 1H, 3-CH), 1.41 (s, 9H, C(CH$_3$)$_3$). ^{13}C NMR (101 MHz, CDCl$_3$) δ_C 172.2 (CO), 138.9 (11-CH), 127.0 (12-CH), 122.3 (CN), 81.5 (C), 80.3 (C), 66.5 (8-CH$_2$), 58.7 (2-CH$_2$), 49.1 (4-CH), 48.6 (6-CH), 37.3 (7-C), 35.8 (9-CH$_2$), 34.7 (10-CH), 28.2 (3-CH$_2$), 28.0 (C($\underline{C}$H$_3$)$_3$). HRMS (ESI$^+$) m/z: [M+H]$^+$ Calcd for C$_{17}$H$_{22}$N$_2$O$_2$ 287.1754; Found 287.1757.

(±)-*tert*-Butyl (1*S*,3[1]*R*,5a*R*,6*R*,8a*R*,9*R*)-9-phenyl-1,4,5,5a,6,8a-hexahydro-1,6-methanopyrrolo[3,2,1-hi]indole-3[1](2*H*)-carboxylate 6ae

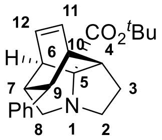

6ae

According to general procedure B, Pd(OAc)$_2$ (6.1 mg, 0.03 mmol), PPh$_3$ (30 mg, 0.11 mmol) and K$_2$CO$_3$ (49 mg, 0.35 mmol) were stirred in dioxane (2.5 mL) for 10 min. (±)-*tert*-Butyl (3[1]*R*,3a*S*,6a*S*)-1,3a,6,6a-tetrahydroazirino[2,3,1-*hi*]indol-3[1](2*H*)-carboxylate **2a** (60 mg, 0.27 mmol) in dioxane (1.25 mL) was added to the stirred mixture and the reaction was heated to 100 °C. After 1.5 h cinnamyl acetate (57 mg, 0.32 mmol) in dioxane (1.25 mL) was added and the reaction mixture stirred for a further 16 h at the same temperature. The crude material was purified by SiO$_2$ flash chromatography (Et$_2$O) to give a pale-yellow oil (44 mg, 48%). ν_{max}/cm^{-1} 2929 (br, s, C-H), 2872 (m, C-H), 1724 (s, C=O), 1165 (s), 700 (m); ^{1}H NMR (400 MHz, CDCl$_3$) δ_H 7.25 – 7.18 (m, 2H, ArCH), 7.18 – 7.11 (m, 1H, ArCH), 7.11 – 7.01 (m, 2H, ArCH), 6.35 (ddd, *J* = 7.9, 6.6, 1.2 Hz, 1H, 12-CH), 6.14 (ddd, *J* = 7.9, 6.5, 1.2 Hz, 1H, 11-CH), 3.62 – 3.47 (m, 2H, 2-CH, 8-CH), 3.38 (ddd, *J* = 6.6, 4.2, 1.2 Hz, 1H, 6-CH), 3.16 (ddd, *J* = 12.7, 9.3, 3.4 Hz, 1H, 2-CH), 2.77 (t, *J* = 2.5 Hz, 1H, 6-CH), 2.52 (dt, *J* = 6.4, 3.1 Hz, 1H, 10-CH), 2.40 (d, *J* = 11.4 Hz, 1H, 8-CH), 2.35 – 2.24 (m, 2H, 4-CH, 7-CH), 2.14 (dtd, *J* = 13.1, 9.6, 3.3 Hz, 1H, 3-CH), 1.87 – 1.76 (m, 1H, 3-CH), 1.45 (s, 9H, C14-H, C(CH$_3$)$_3$). ^{13}C NMR (101 MHz, CDCl$_3$) δ_C 174.1 (CO), 145.5 (11-CH), 134.7 (12-CH), 130.5 (ArC), 128.6 (ArCH), 128.0 (ArCH), 125.9 (ArCH), 80.7 (C), 80.7 (C), 64.7 (8-CH$_2$), 59.3 (2-CH$_2$), 50.3 (4-CH), 47.3 (9-CH$_2$), 45.6 (6-CH), 44.0 (7-CH), 43.1 (10-CH), 28.4 (3-CH$_2$), 28.1 (C($\underline{C}$H$_3$)$_3$). HRMS (ESI$^+$) m/z: [M+H]$^+$ Calcd for C$_{22}$H$_{27}$NO$_2$ 338.2115; Found 338.2108.

(±)-((1*S*,3[1]*R*,5a*R*,6*S*,8a*R*)-1,4,5,5a,6,8a-Hexahydro-1,6-methanopyrrolo[3,2,1-*hi*]indol-3[1](2*H*)-yl)ethan-1-one 6ba

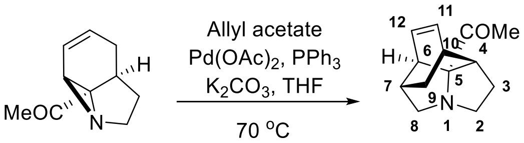

According to general procedure A, Pd(OAc)$_2$ (7 mg, 0.03 mmol), PPh$_3$ (29 mg, 0.11 mmol) and K$_2$CO$_3$ (48 mg, 0.35 mmol) were stirred in THF (2.5 mL) for 5 min. (±)-1-((3[1]*R*,3a*S*,6a*S*)-1,3a,6,6a-tetrahydroazirino[2,3,1-*hi*]indol-3[1](2*H*)-yl)ethan-1-one **2b** (44 mg, 0.27 mmol) in THF (2.5 mL) and allyl acetate (0.03 mL, 0.33 mmol) were added to the stirred solution and the reaction was heated to 70 °C. The crude material was purified by SiO$_2$ flash chromatography, eluting with 0-8% 1:8 NH$_3$:EtOH/CH$_2$Cl$_2$ to give a brown oil (41 mg, 75%). ^{1}H-NMR (400 MHz, CDCl$_3$) δ 6.56-6.52 (m, 1H, 11-CH), 6.02-5.98 (m, 1H, 12-CH), 3.64 (dd, 1H, *J* = 11.7, 4.5 Hz, 8-CH), 3.13-3.10 (m, 1H, 6-CH), 3.04-2.90 (m, 2H, 2-CH$_2$), 2.62-2.57 (m, 1H, 4-CH), 2.43-2.39 (m, 1H, 10-CH), 2.32 (d, 1H, *J* = 11.7 Hz, 8-CH), 2.24 (s, 3H, CH$_3$), 2.02-1.97 (m, 1H, 7-CH), 1.93-1.85 (m, 1H, 3-CH), 1.58-1.48 (m, 1H, 3-CH), 1.45-1.37 (m, 2H, 9-CH$_2$); ^{13}C-NMR (125 MHz, CDCl$_3$) δ 211.2 (CO), 139.2 (11-CH), 127.2 (12-CH), 85.7 (C), 63.6 (8-CH$_2$), 57.3 (2-CH$_2$), 46.2 (6-CH), 45.2 (4-CH), 35.4 (7-CH), 33.9 (10-CH), 31.2 (9-CH$_2$), 27.7, (3-CH$_2$), 25.0 (CH$_3$); ν_{max}/cm^{-1} 2938 (m, C-H), 2865 (m, C-H), 1702 (s, C=O), 1350 (w), 1051 (w); HRMS (ESI$^+$) m/z: [M+H]$^+$ Calcd for C$_{13}$H$_{17}$NO 204.1383; Found 204.1389.

(±)-Methyl (1*R*,3[1]*R*,5a*R*,6*S*,8a*R*)-3[1]-acetyl-3[1],4,5,5a,6,8a-hexahydro-1,6-methanopyrrolo[3,2,1-*hi*]indole-1(2*H*)-carboxylate 6bb

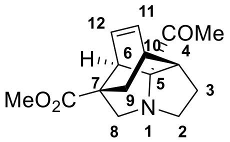

6bb

According to general procedure A, Pd(OAc)$_2$ (6.1 mg, 0.03 mmol), PPh$_3$ (30 mg, 0.11 mmol) and K$_2$CO$_3$ (49 mg, 0.35 mmol) were stirred in THF (2.5 mL) for 10 min. (±)-1-((3[1]*R*,3a*S*,6a*S*)-1,3a,6,6a-Tetrahydroazirino[2,3,1-*hi*]indol-3[1](2*H*)-yl)ethan-1-one **2b** (44 mg, 0.27 mmol) in THF (1.25 mL) and methyl 2-(acetoxymethyl) acrylate (51 mg, 0.32 mmol in THF (1.25 mL) were added and the reaction was heated to 70 °C and stirred for 16 h. The crude material was purified by SiO$_2$ flash chromatography (5 - 20% 3:1 EtOAc:EtOH + 0.1% NH$_3$ in pentane) to give a colourless solid (58 mg, 82%). m.p.: 69 – 71 ˚C; ν_{max}/cm^{-1} 2951 (m, C-H), 2874 (w, C-H), 1729 (s, C=O), 1705 (s, C=O), 731 (m); ^{1}H NMR (500 MHz, CDCl$_3$) δ_H 6.57 (td, *J* = 7.3, 6.8, 1.3 Hz, 1H, 11-CH), 6.01 (tt, *J* = 7.6, 1.0 Hz, 1H, 12-CH), 3.76 (d, *J* = 11.2 Hz, 1H, 8-CH), 3.68 (s, 3H, OCH$_3$), 3.45 (d, *J* = 6.4 Hz, 1H, 6-CH), 3.02 (dd, *J* = 9.0, 6.2 Hz, 2H, 2-CH$_2$), 2.62 – 2.58 (m, 1H, 4-CH), 2.57 (d, *J* = 11.3 Hz, 1H, 8-CH), 2.51 (dd, *J* = 6.6, 3.1 Hz, 1H, 10-CH), 2.27 (s, 3H, CH$_3$), 1.94 (ddt, *J* = 12.8, 9.3, 6.2 Hz, 1H, 3-CH), 1.86 – 1.79 (m, 1H,

9-CH), 1.76 (dd, J = 13.7, 3.1 Hz, 1H, 9-CH), 1.63 – 1.52 (m, 1H, 3-CH). ^{13}C NMR (126 MHz, $CDCl_3$) δ_C 211.0 (CO), 174.5 (CO), 139.1 (11-CH), 126.4 (12-CH), 85.7 (5-C), 66.7 (8-CH_2), 57.4 (2-CH_2), 52.1 (7-CH), 51.5 (OCH_3), 48.4 (6-CH), 45.0 (4-CH), 35.1 (10-CH), 34.1 (9-CH_2), 28.1 (3-CH_2), 25.0 (CH_3). HRMS (ESI$^+$) m/z: $[M+H]^+$ Calcd for $C_{15}H_{19}NO_3$ 262.1438; Found 262.1417.

(±)-1,1'-((1*R*,31*R*,5a*R*,6*S*,8a*R*)-5,5a,6,8a-tetrahydro-1,6-methanopyrrolo[3,2,1-*hi*]indole-1,3^1(2*H*,4*H*)-diyl)bis(ethan-1-one) 6bc

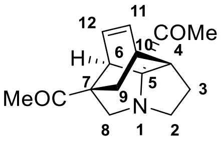

6bc

According to general procedure A, $Pd(OAc)_2$ (6.1 mg, 0.03 mmol), PPh_3 (30 mg, 0.11 mmol) and K_2CO_3 (49 mg, 0.35 mmol) were stirred in THF (2.5 mL) for 10 min. (±)-1-((3^1*R*,3a*S*,6a*S*)-1,3a,6,6a-Tetrahydroazirino[2,3,1-*hi*]indol-3^1(2*H*)-yl)ethan-1-one **2b** (44 mg, 0.27 mmol) in THF (1.25 mL) and 2-methylene-3-oxobutyl acetate (46 mg, 0.32 mmol) in THF (1.25 mL) were added and the reaction was heated to 70 °C and stirred for 16 h. The crude material was purified by SiO_2 flash chromatography (10 - 30% 3:1 EtOAc:EtOH + 0.1% NH_3 in pentane) and any product coeluting with triphenylphosphine oxide was subjected to another column with identical conditions to give a yellow semi-solid (49 mg, 74%). ν_{max}/cm^{-1} 2930 (br, w, C-H), 2802 (w, C-H), 1702 (w, C=O), 904 (s), 726 (s); ^{1}H NMR (500 MHz, $CDCl_3$) δ_H 6.51 (ddd, J = 8.0, 6.6, 1.2 Hz, 1H, 11-CH), 6.01 (ddd, J = 7.9, 6.5, 1.2 Hz, 1H, 12-CH), 3.65 (dd, J = 11.3, 1.4 Hz, 1H, 8-CH), 3.36 (d, J = 6.6, 1H, 6-CH), 3.07 – 2.91 (m, 2H, 2-CH_2), 2.60 (ddt, J = 9.3, 4.4, 2.3 Hz, 1H, 4-CH), 2.57 – 2.50 (m, 1H, 10-CH), superimposed on 2.51 (d, J = 11.2 Hz, 1H, 8-CH), 2.23 (s, 3H, CH_3), 2.07 (s, 3H, CH_3), 1.93 (dddd, J = 13.4, 9.4, 7.9, 4.0 Hz, 1H, 3-CH), 1.77 (dd, J = 13.4, 3.2 Hz, 1H, 9-CH), 1.67 – 1.49 (m, 2H, 9-CH, 3-CH). ^{13}C NMR (126 MHz, $CDCl_3$) δ_C 210.9 (CO), 208.4 (CO), 138.7 (11-CH), 127.1 (12-CH), 85.9 (5-C), 65.9 (8-CH_2), 58.6 (7-C), 57.4 (2-CH_2), 47.3 (6-CH), 45.3 (4-CH), 35.3 (10-CH), 34.1 (9-CH_2), 28.1 (3-CH_2), 27.0 (CH_3), 25.1 (CH_3). HRMS (ESI$^+$) m/z: $[M+H]^+$ Calcd for $C_{15}H_{19}NO_2$ 246.1489; Found 246.1471.

3mmol scale

According to general procedure A, $Pd(OAc)_2$ (67 mg, 0.3 mmol), PPh_3 (330 mg, 1.26 mmol) and K_2CO_3 (539 mg, 3.9 mmol)were stirred in THF (50 mL) for 10 min. (±)-1-((3^1*R*,3a*S*,6a*S*)-1,3a,6,6a-Tetrahydroazirino[2,3,1-*hi*]indol-3^1(2*H*)-yl)ethan-1-one **2b** (489 mg, 3 mmol) in THF (2.5 mL) and 2-methylene-3-oxobutyl acetate (512 mg, 3.6 mmol) in THF (2.5 mL) were added and the reaction was heated to 70 °C and stirred for 16 h. The crude material was purified by SiO_2 flash chromatography (30 - 40% 3:1 EtOAc:EtOH + 0.1% NH_3 in pentane) to give a yellow semi-solid (542 mg, 74%).

(±)-1,1'-((1*R*,3^1*R*,5a*R*,6*S*,8a*R*)-5,5a,6,8a-tetrahydro-1,6-methanopyrrolo[3,2,1-*hi*]indole-1,3^1(2*H*,4*H*)-diyl)bis(ethan-1-one) 6bd

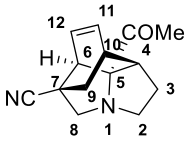

6bd

According to general procedure A, $Pd(OAc)_2$ (6.1 mg, 0.03 mmol), PPh_3 (30 mg, 0.11 mmol) and K_2CO_3 (49 mg, 0.35 mmol) were stirred in THF (2.5 mL) for 10 min. (±)-1-((3^1*R*,3a*S*,6a*S*)-1,3a,6,6a-Tetrahydroazirino[2,3,1-*hi*]indol-3^1(2*H*)-yl)ethan-1-one **2b** (44 mg, 0.27 mmol) in THF (1.25 mL) and 2- cyanoallyl acetate (41 mg, 0.32 mmol) in THF (1.25 mL) were added and the reaction was heated to 70 °C and stirred for 16 h. The crude material was purified by SiO_2 flash chromatography (5 - 10% 3:1 EtOAc:EtOH + 0.1% NH_3 in pentane) to give a yellow semi-solid (46 mg, 75%). ν_{max}/cm^{-1} 2953 (br, m, C-H), 2875 (m, C-H), 2236 (w, C≡N), 1702 (s, C=O), 728 (m), 726 (s); ^{1}H NMR (400 MHz, $CDCl_3$) δ_H 6.68 (ddd, J = 8.0, 6.5, 1.2 Hz, 1H, 11-CH), 6.08 (ddd, J = 8.1, 6.4, 1.1 Hz, 1H, 12-CH), 3.77 (dd, J = 11.4, 1.3 Hz, 1H, 8-CH), 3.37 (dt, J = 6.4, 0.8 Hz, 1H, 6-CH), 3.12 – 2.97 (m, 2H, 2-CH_2), 2.71 (d, J = 11.4 Hz, 1H, 8-CH), 2.60 – 2.49 (m, 2H, 4-CH, 10-CH), 2.23 (s, 3H, CH_3), 1.99 – 1.82 (m, 2H, 3-CH, 9-CH), 1.78 – 1.71 (m, 1H, 9-CH), 1.56 – 1.45 (m, 1H, 3-CH). ^{13}C NMR (101 MHz, $CDCl_3$) δ_C 209.6 (CO), 140.4 (CO), 125.3 (11-CH), 122.2 (12-CH), 84.9 (5-C), 66.5 (8-CH_2), 57.6 (2-CH_2), 49.7 (6-CH), 45.0 (4-CH), 37.9 (7-C), 35.9 (9-CH_2), 34.6 (10-CH), 27.9 (3-CH_2), 25.0 (CH_3). HRMS (ESI$^+$) m/z: $[M+H]^+$ Calcd for $C_{14}H_{16}N_2O$ 229.1335; Found 229.1333.

(±)-1-((1*S*,3[1]*R*,5a*R*,6*R*,8a*R*,9*R*)-9-phenyl-1,4,5,5a,6,8a-hexahydro-1,6-methanopyrrolo[3,2,1-*hi*]indol-3[1](2*H*)-yl)ethan-1-one 6be

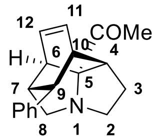

6be

According to general procedure A, Pd(OAc)$_2$ (6.1 mg, 0.03 mmol), PPh$_3$ (30 mg, 0.11 mmol) and K$_2$CO$_3$ (49 mg, 0.35 mmol) were stirred in THF (2.5 mL) for 10 min. (±)-1-((3[1]*R*,3a*S*,6a*S*)-1,3a,6,6a-Tetrahydroazirino[2,3,1-*hi*]indol-3[1](2*H*)-yl)ethan-1-one **2b** (44 mg, 0.27 mmol) in THF (1.25 mL) and 2- cinnamyl acetate (57 mg, 0.32 mmol) in THF (1.25 mL) were added and the reaction was heated to 70 °C and stirred for 16 h. The crude material was purified by SiO$_2$ flash chromatography (5 - 20% 3:1 EtOAc:EtOH + 0.1% NH$_3$ in pentane) to give a pale yellow oil (37 mg, 49%). ν_{max}/cm^{-1} 2942 (br, m, C-H), 2870 (m, C-H), 1702 (s, C=O), 1351 (w), 700 (s); ^{1}H NMR (500 MHz, CDCl$_3$) δ_H 7.27 – 7.20 (m, 2H, ArCH), 7.19 – 7.12 (m, 1H, ArCH), 7.09 – 7.04 (m, 2H, ArCH), 6.27 – 6.14 (m, 2H, 11-CH, 12-CH), 3.65 (dd, *J* = 11.5, 4.2 Hz, 1H, 8-CH), 3.21 (ddd, *J* = 6.1, 4.3, 1.5 Hz, 1H, 6-CH), 3.13 (dd, *J* = 9.0, 6.3 Hz, 2H, 2-CH$_2$), 2.81 (t, *J* = 2.5 Hz, 1H, 9-CH), 2.63 (dt, *J* = 9.4, 3.3 Hz, 1H, 4-CH), superimposed on 2.61 – 2.56 (m, 1H, 10-CH), 2.48 (d, *J* = 11.5 Hz, 1H, 8-CH), 2.38 (td, *J* = 4.2, 1.8 Hz, 1H, 7-CH), 2.27 (s, 3H, CH$_3$), 1.99 (ddt, *J* = 13.5, 9.4, 6.2 Hz, 1H, 3-CH), 1.78 (ddt, *J* = 13.9, 9.3, 4.5 Hz, 1H, 3-CH). ^{13}C NMR (126 MHz, CDCl$_3$) δ_C 212.1 (CO), 145.3 (ArC), 136.3 (11-CH), 128.6 (ArCH), 128.6 (12-CH), 128.0 (ArCH), 126.0 (ArCH), 85.5 (5-CH), 64.5 (8-CH$_2$), 58.0 (2-CH$_2$), 47.4 (9-CH), 47.1 (6-CH), 45.9 (4-CH), 44.7 (7-CH), 42.9 (10-CH), 28.0 (3-CH$_2$), 25.3 (CH$_3$). HRMS (ESI$^+$) m/z: [M+H]$^+$ Calcd for $C_{19}H_{22}NO$ 280.1696; Found 280.1696. NOE enhancement between alkenic CHs and ortho Hs on phenyl ring confirmed the equatorial stereochemistry conformation. See S43 and S44 for spectra.

(±)-(1*S*,3[1]*R*,5a*R*,6*S*,8a*R*)-*N*-ethyl-1,4,5,5a,6,8a-hexahydro-1,6-methanopyrrolo[3,2,1-*hi*]indole-3[1](2*H*)-carboxamide 6ca

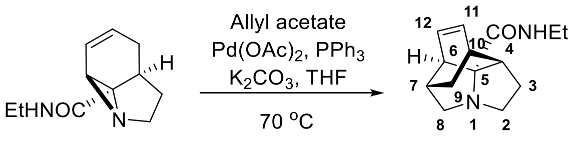

2c **6ca**

According to general procedure A, Pd(OAc)$_2$ (7 mg, 0.03 mmol), PPh$_3$ (29 mg, 0.11 mmol) and K$_2$CO$_3$ (48 mg, 0.35 mmol) were stirred in THF (2.5 mL) for 5 min. (±)-(3[1]*R*,3a*S*,6a*S*)-*N*-ethyl-1,3a,6,6a-tetrahydroazirino[2,3,1-*hi*]indol-3[1](2*H*)-carboxamide **2c** (52 mg, 0.27 mmol) in THF (2.5 mL) and allyl acetate (0.03 mL, 0.33 mmol) were added to the stirred solution and the reaction was heated to 70 °C. The crude material was purified by SiO$_2$ flash chromatography, eluting with 0-1% 1:8 NH$_3$:EtOH/Et$_2$O to give a colourless oil (33 mg, 52%). ^{1}H-NMR (400 MHz, CDCl$_3$) δ 7.78 (br s, 1H, NH), 6.55-6.51 (m, 1H, 11-CH), 6.14-6.10 (m, 1H, 12-CH), 3.46 (dd, 1H, *J* = 11.4, 4.4 Hz, 8-CH), 3.24-3.17 (m, 2H, C$\underline{H_2}$CH$_3$), 3.02-2.94 (m, 2H, 2-CH$_2$), 2.86-2.83 (app t, 1H, 6-CH), 2.61 (dqn, 1H, *J* = 9.3, 2.0 Hz, 4-CH), 2.41-2.38 (m, 1H, 10-CH), 2.26 (d, 1H, *J* = 11.4 Hz, 8-CH), 1.92-1.83 (m, 2H, 3-CH and 7-CH), 1.57-1.48 (m, 1H, 3-CH), 1.46-1.35 (m, 2H, 9-CH$_2$), 1.10 (t, 3H, *J* = 7.4 Hz, CH$_3$); ^{13}C-NMR (125 MHz, CDCl$_3$) δ 174.8 (CO), 137.7 (11-CH), 128.0 (12-CH), 80.9 (C), 62.8 (8-CH$_2$), 56.9 (2-CH$_2$), 47.7 (6-CH), 47.0 (4-CH), 36.0 (7-CH), 34.2 (10-CH), 33.7 ($\underline{C}$H$_2$CH$_3$), 31.3 (9-CH$_2$), 27.9 (3-CH$_2$), 14.9 (CH$_3$); ν_{max}/cm^{-1} 3340 (br, w, N-H), 2933 (s, C-H), 2868 (s, C-H), 1660 (s, C=O), 1505 (s), 726 (m); HRMS (ESI$^+$) m/z: [M+H]$^+$ Calcd for $C_{14}H_{20}N_2O$ 233.1648; Found 233.1646.

(±)-Methyl (1*S*,3[1]*R*,5a*R*,6*S*,8a*R*)-3[1]-(ethylcarbamoyl)-3[1],4,5,5a,6,8a-hexahydro-1,6-methano pyrrolo[3,2,1-*hi*]indole-1(2*H*)-carboxylate 6cb

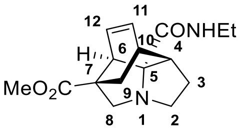

6cb

According to general procedure A, Pd(OAc)$_2$ (7 mg, 0.03 mmol), PPh$_3$ (29 mg, 0.11 mmol) and K$_2$CO$_3$ (48 mg, 0.35 mmol) were stirred in THF (2.5 mL) for 5 min. (±)-(3[1]*R*,3a*S*,6a*S*)-*N*-ethyl-1,3a,6,6a-tetrahydroazirino[2,3,1-*hi*]indol-3[1](2*H*)-carboxamide **2c** (52 mg, 0.27 mmol) in THF (2.5 mL) and methyl 2-(acetoxymethyl)acrylate (52 mg, 0.33 mmol) were added to the stirred solution and the reaction was heated to 70 °C. The crude material was purified by SiO$_2$ flash chromatography, eluting with 0-1% 1:8 NH$_3$:EtOH/Et$_2$O to give a colourless oil (45 mg, 58%). ^{1}H-NMR (400 MHz, CDCl$_3$) δ 7.70 (br s, 1H, NH), 6.55 (app t, 1H, 11-CH), 6.10 (app t, 1H, 12-CH), 3.70-3.66 (m, 1H, 8-CH), superimposed on 3.62 (s, 3H, OCH$_3$), 3.24-3.17 (m, 3H, 6-CH and C$\underline{H_2}$CH$_3$), 3.09-2.94 (m, 2H, 2-CH$_2$), 2.61 (br d, 1H, *J* = 7.8 Hz, 4-CH), 2.50 (d, 2H, *J* = 11.5 Hz, 8-CH), 1.97-1.89 (m, 1H, 3-CH), 1.83-1.69 (m, 2H, 9-CH$_2$), 1.60-1.51 (m, 1H, 3-CH), 1.10 (t, 3H, *J* = 7.2 Hz, CH$_3$); ^{13}C-NMR

(125 MHz, $CDCl_3$) δ 174.3 (CO), 173.3 (CO), 137.7 (11-CH), 127.0 (12-CH), 81.1 (C), 65.6 (8-CH_2), 56.9 (2-CH_2), 52.1 (OCH_3), 51.7 (C), 49.9 (6-CH), 46.6 (4-CH), 35.2 (10-CH) 34.0 (C̲H_2CH_3), 33.9 (9-CH_2), 27.9 (3-CH_2), 14.8 (CH_3); ν_{max}/cm^{-1} 3348 (br, w, N-H), 2950 (s, C-H), 2874 (m, C-H), 1727 (s, C=O), 1664 (s, C=O), 1238 (s), 1509 (s); HRMS (ESI^+) m/z: $[M+H]^+$ Calcd for $C_{16}H_{22}N_2O_3$ 291.1703; Found 291.1697.

(±)-(1*S*,3^1*R*,5a*R*,6*S*,8a*R*)-1-Acetyl-*N*-ethyl-1,4,5,5a,6,8a-hexahydro-1,6-methanopyrrolo[3,2,1-*hi*]indole-3^1(2*H*)-carboxamide 6cc

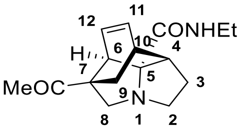

6cc

According to general procedure A, $Pd(OAc)_2$ (7 mg, 0.03 mmol), PPh_3 (29 mg, 0.11 mmol) and K_2CO_3 (48 mg, 0.35 mmol) were stirred in THF (2.5 mL) for 5 min. (±)-(3^1*R*,3a*S*,6a*S*)-*N*-ethyl-1,3a,6,6a-tetrahydroazirino[2,3,1-*hi*]indol-3^1(2*H*)-carboxamide **2c** (52 mg, 0.27 mmol) in THF (2.5 mL) and 2-methylene-3-oxo butyl acetate (47 mg, 0.33 mmol) were added to the stirred solution and the reaction was heated to 70 °C. The crude material was purified by SiO_2 flash chromatography, eluting with 0-1% 1:8 NH_3:EtOH/Et_2O to give a yellow oil (49 mg, 66%). ^{1}H-NMR (400 MHz, $CDCl_3$) δ 7.66 (br s, 1H, NH), 6.57-6.53 (m, 1H, 11-CH), 6.17-6.13 (m, 1H, 12-CH), 3.61 (d, 1H, J = 11.5 Hz, 8-CH), 3.25-3.18 (m, 2H, CH̲$_2CH_3$), 3.13 (d, 1H, J = 6.7 Hz, 6-CH), 3.05-2.95 (m, 2H, 2-CH_2), 2.64 (m, 1H, 4-CH), 2.56-2.53 (m, 1H, 10-CH), 2.47 (d, 1H, J = 11.5 Hz, 8-CH), 2.05 (s, 3H, CH_3), 1.99-1.91 (m, 1H, 3-CH), 1.76-1.62 (m, 2H, 9-CH_2), 1.64-1.53 (m, 1H, 3-CH), 1.11 (t, 3H, J = 7.4 Hz, CH_3); ^{13}C-NMR (125 MHz, $CDCl_3$) δ 208.5 (CO), 173.8 (CO), 137.4 (11-CH), 127.0 (12-CH), 81.1 (C), 64.7 (8-CH_2), 58.5 (C), 56.9 (2-CH_2), 49.0 (6-CH), 46.8 (4-CH), 35.3 (10-CH), 33.9 (C̲H_2CH_3), 28.0 (3-CH_2), 26.8 (CH_3), 14.8 (CH_3); ν_{max}/cm^{-1} 3320 (br, w, N-H), 2932 (br, w, C-H), 1733 (m, C=O), 1662 (s, C=O), 1239 (s); HRMS (ESI^+) m/z: $[M+H]^+$ Calcd for $C_{18}H_{26}N_2O_4$ 335.1965; Found 335.1963.

(±)-(1*R*, 3^1*R*,5a*R*,6*S*,8a*R*)-1-Cyano-*N*-ethyl-1,4,5,5a,6,8a-hexahydro-1,6-methanopyrrolo[3,2,1-*hi*]indole-3^1(2*H*)-carboxamide 6cd

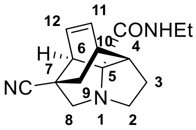

6cd

According to general procedure A, $Pd(OAc)_2$ (7 mg, 0.03 mmol), PPh_3 (29 mg, 0.11 mmol) and K_2CO_3 (48 mg, 0.35 mmol) were stirred in THF (2.5 mL) for 5 min. (±)-(3^1*R*,3a*S*,6a*S*)-*N*-ethyl-1,3a,6,6a-tetrahydroazirino[2,3,1-*hi*]indol-3^1(2*H*)-carboxamide **2c** (52 mg, 0.27 mmol) in THF (2.5 mL) and 2-cyanoallylacetate (41 mg, 0.33 mmol) were added to the stirred solution and the reaction was heated to 70 °C. The crude material was purified by SiO_2 flash chromatography, eluting with Et_2O to give a pale-yellow oil (40 mg, 58%). Recrystallisation through slow cooling of a saturated solution in refluxing Et_2O afforded material suitable for X-ray diffraction studies. Mp. 104 - 108 °C. ^{1}H-NMR (400 MHz, $CDCl_3$) δ 7.59 (br s, 1H, NH), 6.71-6.67 (m, 1H, 11-CH), 6.23-6.19 (m, 1H, 12-CH), 3.71 (d, 1H, J = 11.4 Hz, 8-CH), 3.26-3.15 (m, 3H, 6-CH and CH_2CH_3), 3.13-2.98 (m, 2H, 2-CH_2), 2.69 (d, 1H, J = 11.4 Hz, 8-CH), 2.62 (br d, 1H, J = 8.8 Hz, 4-CH), 2.56-2.53 (m, 1H, 10-CH), 2.00-1.91 (m, 1H, 3-CH), 1.88-1.84 (m, 1H, 9-CH), 1.77-1.73 (m, 1H, 9-CH), 1.57-1.48 (m, 1H, 3-CH), 1.11 (t, 3H, J = 7.6 Hz, CH_3); ^{13}C-NMR (125 MHz, $CDCl_3$) δ 172.5 (CO), 138.9 (11-CH), 125.8 (12-CH), 121.9 (CN), 80.3 (C), 65.4 (8-CH_2), 57.1 (2-CH_2), 50.9 (6-CH), 46.5 (4-CH), 38.0 (C), 35.8 (9-CH_2), 34.5 (10-CH), 34.0 (C̲H_2CH_3), 27.6 (3-CH_2), 14.8 (CH_3); ν_{max}/cm^{-1} 3351 (br, w, N-H), 2935 (m, C-H), 2875 (m, C-H), 2235 (w, C≡N), 1664 (s, C=O), 1510 (s); HRMS (ESI^+) m/z: $[M+H]^+$ Calcd for $C_{15}H_{19}N_3O$ 258.1603; Found 258.1602.

(±)-(1*S*, 3^1*R*,5a*R*,6*R*,8a*R*,9*R*)-*N*-ethyl-9-phenyl-1,4,5,5a,6,8a-hexahydro-1,6-methanopyrrolo [3,2,1-*hi*]indole-3^1(2*H*)-carboxamide 6ce

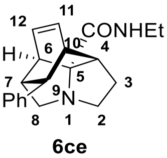

6ce

According to general procedure A, $Pd(OAc)_2$ (7 mg, 0.03 mmol), PPh_3 (29 mg, 0.11 mmol) and K_2CO_3 (48 mg, 0.35 mmol) were stirred in THF (2.5 mL) for 5 min. (±)-(3^1*R*,3a*S*,6a*S*)-*N*-ethyl-1,3a,6,6a-tetrahydroazirino[2,3,1-*hi*]indol-3^1(2*H*)-carboxamide **2c** (52 mg, 0.27 mmol) in THF (2.5 mL) and cinnamyl acetate (58 mg, 0.33 mmol) were added to the stirred solution and the reaction was heated to 70 °C. The crude material was purified by SiO_2 flash chromatography, eluting with Et_2O to give a colourless oil (55 mg, 66%). ^{1}H-NMR (400 MHz, $CDCl_3$) δ 7.81 (br s,1H, NH), 7.25-7.15 (m, 2H, ArCH), 7.17-

7.13 (m, 1H, ArCH), 7.08-7.06 (m, 2H, ArCH), 6.36-6.32 (m, 1H, 12-CH), 6.25-6.21 (m, 1H, 11-CH), 3.54 (dd, 1H, *J* = 11.6, 3.4 Hz, 8-CH), 3.29-3.23 (m, 2H, C<u>H</u>$_2$CH$_3$), 3.20-3.07 (m, 2H, 2-CH$_2$), 3.01-2.98 (m, 1H, 6- CH), 2.80 (br s, 1H, 9-CH), 2.66 (dt, 1H, *J* = 9.5, 3.1 Hz, 4-CH), 2.61-2.59 (m, 1H, 10-CH), 2.43 (d, 1H, *J* = 11.6 Hz, 8-CH), 2.33-2.31 (m, 1H, 7-CH), 2.06-1.97 (m, 1H, 3-CH), 1.84-1.75 (m, 1H, 3-CH), 1.13 (t, 3H, *J* = 7.3 Hz, CH$_3$); ^{13}C-NMR (125 MHz, CDCl$_3$) δ 174.7 (CO), 145.3 (ArC), 134.6 (11-CH), 129.3 (12-CH), 128.4 (ArCH), 127.8 (ArCH), 125.8 (ArCH), 80.6 (C), 63.4 (8-CH$_2$), 57.4 (2-CH$_2$), 48.4 (6-CH), 47.6 (4-CH), 47.0 (9-CH), 45.0 (7-CH), 43.0 (10-CH), 33.8 (<u>C</u>H$_2$CH$_3$), 28.0 (3-CH$_2$), 14.9 (CH$_3$); ν_{max}/cm^{-1} 3344 (br, w, N-H), 2930 (m, C-H), 2870 (m, C-H), 166s (s, C=O), 1504 (s), 700 (s); HRMS (ESI$^+$) m/z: [M+H]$^+$ Calcd for $C_{20}H_{24}N_2O$ 309.1961; Found 309.1959.

(±)-*tert*-Butyl (3a*R*,7a*S*)-1-(2-((*E*)-benzylidene)-3-oxobutyl)-1,2,3,3a-tetrahydro-7a*H*-indole-7a-carboxylate 12af

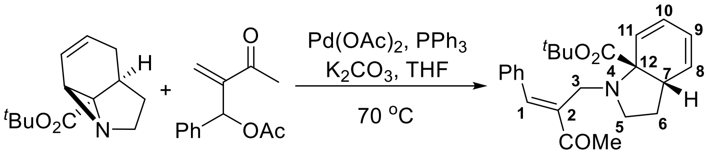

2a **5f** **12af**

According to general procedure A, to a dried Schlenk tube under nitrogen were added Pd(OAc)$_2$ (6.4 mg, 0.028 mmol), triphenylphosphine (30 mg, 0.11 mmol), potassium carbonate (48 mg, 0.35 mmol) and dry THF (3 mL), the mixture stirred for 5 min, a solution of azirdine **2a** (60 mg, 0.27 mmol) and tether (77 mg, 0.35 mmol) in dry THF (1 mL) was added, the mixture sparged with nitrogen and heated to 70 °C with stirring. After 13.5 h the reaction was cooled and evaporated. Purification by silica gel chromatography (EtOAc/petrol, 5:95 to 15:85 as eluent) afforded the title compound (46 mg, 45%) as a clear oil. ν_{max} /cm^{-1} (film) 2972, 1713, 1688, 1666, 1450, 1367 and 1233; ^{1}H NMR (500 MHz, CDCl$_3$) δ_H 7.53 (3H, dd, J 7.5, 1.5, 1-CH, ArCH) , 7.33 – 7.39 (3H, m, ArCH), 6.05 – 6.12 (2H, m, 10-CH, 11-CH) , 5.86 (1H, ddt, J 9.6, 4.2, 2.1, 9-CH), 5.64 – 5.71 (1H, m, 8-CH), 3.86 (1H, d, J 12.1, 3-CH), 3.71 (1H, dd, J 12.2, 1.2, 3-CH), 3.19 (1H, ddt, J 9.4, 6.4, 3.0, 7-CH), 2.53 (1H, td, J 8.1, 2.8, 5-CH), 2.44 (3H, s, CH$_3$) , 2.41 (1H, app q, J 8.1, 5-CH) , 2.23 – 2.33 (1H, m, 6-CH) , 1.57 – 1.65 (1H, m, 6-CH), 1.46 (9H, s, *Me*$_3$C); ^{13}C NMR (126 MHz, CDCl$_3$) δ_C 201.1 (CO), 172.6 (CO), 140.3 (2-*C*H), 139.8 (1-CH), 135.4 (ArC), 130.7 (8-CH), 130.0 (ArCH), 128.6 (ArCH), 128.3 (ArCH), 123.4 (CH), 123.0 (CH), 118.9 (9-CH), 81.5 (<u>C</u>(CH$_3$)$_3$), 67.7 (12-C), 45.5 (5-CH$_2$), 43.8 (3-*C*H$_2$), 41.5 (7-CH), 32.1 (6-CH$_2$), 28.2 (C(<u>C</u>H$_3$)$_3$), 27.1 (CH$_3$); HRMS (ESI$^+$) m/z: [M+H]$^+$ Calcd for $C_{24}H_{30}NO_3$ 380.2220; Found 380.2230.

(±)-(*E*)-3-(((3a*R*,7a*S*)-7a-acetyl-2,3,3a,7a-tetrahydro-1*H*-indol-1-yl)methyl)-4-phenylbut-3-en-2-one 12bf

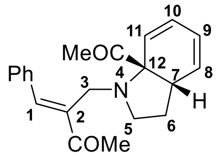

12bf

According to general procedure A, Pd(OAc)$_2$ (6.1 mg, 0.03 mmol), PPh$_3$ (30 mg, 0.11 mmol) and K$_2$CO$_3$ (49 mg, 0.35 mmol) were stirred in THF (2.5 mL) for 10 min. (±)-1-((3^1*R*,3a*S*,6a*S*)-1,3a,6,6a-Tetrahydroazirino[2,3,1-*hi*]indol-3^1(2*H*)-yl)ethan-1-one **2b** (44 mg, 0.27 mmol) in THF (1.25 mL) and 2-methylene-3-oxo-1-phenylbutyl acetate (71 mg, 0.32 mmol) in THF were added and the reaction was heated to 70 °C and stirred for 16 h. The crude material was purified by SiO$_2$ flash chromatography (50% Et$_2$O in petrol) to give a yellow oil (40 mg, 46%). ν_{max}/cm^{-1} 3035, 2961, 1704, 1667, 1350, 1231; ^{1}H NMR (400 MHz, CDCl$_3$) δ_H 7.49 (s, 1H, 1-CH), 7.47 – 7.42 (m, 2H, ArCH), 7.41 – 7.31 (m, 3H, ArCH), 6.07 (ddd, J = 9.8, 5.5, 1.0 Hz, 1H, 10-CH), 5.86 – 5.74 (m, 2H, 9-CH, 11-CH), 5.70 (ddt, J = 9.6, 4.4, 1.1 Hz, 1H, 8-CH), 3.85 (dd, J = 12.5, 0.7 Hz, 1H, 3-CH), 3.60 (dd, J = 12.5, 0.7 Hz, 1H, 3-CH), 3.06 (dddd, J = 8.7, 6.3, 4.3, 1.7 Hz, 1H, 7-CH), 2.63 (ddd, J = 9.1, 7.0, 6.3 Hz, 1H, 5-CH), 2.45 (s, 3H, CH$_3$), 2.40 – 2.32 (m, 1H, 5-CH), 2.15 (s, 3H, CH$_3$), 2.17 – 2.07 (m, 1H, 6-CH), 1.55 (ddt, J = 11.9, 7.3, 6.3 Hz, 1H, 6-CH)). ^{13}C NMR (101 MHz, CDCl$_3$) δ_C 210.8 (CO), 201.1 (CO), 140.7 (2-C), 140.2 (1-CH), 135.5 (ArC), 130.2 (8-CH), 129.8 (ArCH), 128.8 (ArCH), 128.6 (ArCH), 125.5 (10-CH), 122.3 (CH), 120.5 (CH), 73.2 (12C), 49.1 (5-CH$_2$), 43.8 (3-CH$_2$), 41.1 (7-CH), 32.9 (6-CH$_2$), 27.0 (CH$_3$), 26.0 (CH$_3$). HRMS (ESI$^+$) m/z: [M+H]$^+$ Calcd for $C_{21}H_{23}NO_2$ 322.1802; Found 322.1814.

(±)-(3a*R*,7a*S*)-1-(2-((*E*)-Benzylidene)-3-oxobutyl)-*N*-ethyl-1,2,3,3a-tetrahydro-7a*H*-indole-7a-carboxamide 12cf

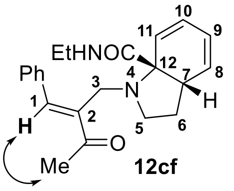

According to general procedure A, $Pd(OAc)_2$ (6.1 mg, 0.03 mmol), PPh_3 (30 mg, 0.11 mmol) and K_2CO_3 (49 mg, 0.35 mmol) were stirred in THF (2.5 mL) for 10 min. (±)-(3[1]*R*,3a*S*,6a*S*)-*N*-Ethyl-1,3a,6,6a-tetrahydroazirino[2,3,1-*hi*]indol-3[1](2*H*)-carboxamide **2c** (52 mg, 0.27 mmol) in THF (1.25 mL) and 2-methylene-3-oxo-1-phenylbutyl acetate (71 mg, 0.32 mmol) in THF were added and the reaction was heated to 70 °C and stirred for 16 h. The crude material was purified by SiO_2 flash chromatography (80% - 100% Et_2O in petrol) to give a pale-yellow oil (27 mg, 29%). ν_{max}/cm^{-1} 3300, 2967, 2873, 1656, 1514, 1233; ^1H NMR (400 MHz, $CDCl_3$) δ_H 8.27 (br s, 1H, NH), 7.62 (s, 1H, 1-CH), 7.43 – 7.29 (m, 5H, ArCH), 6.04 (ddt, *J* = 9.7, 5.4, 0.7 Hz, 1H, 10-CH), 5.78 (dddd, *J* = 9.7, 5.5, 2.3, 0.8 Hz, 1H, 9-CH), 5.48 (ddt, *J* = 9.6, 3.6, 1.0 Hz, 1H, 8-CH), 5.46 – 5.37 (m, 1H, 11-CH), 3.66 (dd, *J* = 12.4, 1.3 Hz, 1H, 3-CH), 3.57 (d, *J* = 12.4 Hz, 1H, 3-CH), 3.33 – 3.28 (m, *J* = 7.3, 5.7 Hz, 3H, $C\underline{H}_2CH_3$, 7-CH), 2.76 (ddd, *J* = 8.8, 6.7, 2.2 Hz, 1H, 5-CH), 2.50 (s, 3H, CH_3), 2.28 – 2.19 (m, 1H, 5-CH), 1.97 (dddd, *J* = 12.1, 10.5, 8.4, 6.7 Hz, 1H, 6-CH), 1.63 – 1.53 (m, 1H, 6-CH), 1.20 (t, *J* = 7.3 Hz, 3H, $CH_2C\underline{H}_3$). ^{13}C NMR (101 MHz, $CDCl_3$) δ_C 201.0 (CO), 176.0 (CO), 142.4 (1-CH), 140.6 (2-C), 135.1 (ArC), 130.5 (8-CH), 129.5 (ArCH), 129.0 (ArCH), 128.6 (ArCH), 126.2 (10-CH), 122.4 (11-CH), 121.6 (9-CH), 69.4 (12-C), 51.5 (5-CH_2), 44.8 (3-CH_2), 43.1 (7-CH), 34.4 ($\underline{C}H_2CH_3$), 31.8 (6-CH_2), 26.7 (CH_3), 14.9 ($CH_2\underline{C}H_3$). HRMS (ESI$^+$) m/z: $[M+H]^+$ Calcd for $C_{22}H_{27}N_2O_2$ 351.2067; Found 351.2082. Stereochemistry was proven by the nOe interactions depicted above. See S52 and S53 spectra.

Competition Experiment

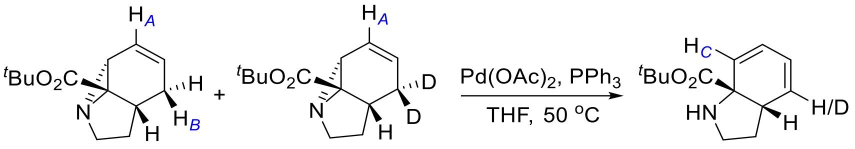

2a **17** **18**

To a dried Schlenk tube under a positive pressure of nitrogen were added $Pd(OAc)_2$ (7.3 mg, 0.033 mmol), triphenylphosphine (34 mg, 0.13 mmol) and anhydrous THF (2 mL). The mixture was stirred at rt for 5 min and a solution of substrates **2a** and **17** (68 mg, 0.31 mmol, 3:1 H/D by ^1H NMR) in anhydrous THF (3 mL) was added. The mixture was degassed by sparging with nitrogen and heated to 50 °C with stirring. The reaction was sampled by removal of a 150 µL aliquot after 15 min, 25 min, 35 min, 45 min and 56 min. The aliquot was passed through SiO_2 (EtOAc elution) and evaporated prior to ^1H NMR analysis in $CDCl_3$ containing 1,3,5-trimethoxybenzene (0.03 M) as internal standard. Total starting material concentration was assessed through the integration of ^1H environment **A** (5.79 ppm); total product concentration was assessed through the integration of ^1H environment **C** (5.61 ppm); and non-deuterated starting material concentration was measured by integration of ^1H environment **B**. Errors were calculated in Microsoft Excel via a least squares analysis. $k_H/k_D \sim 1$. δ_H (500 MHz, $CDCl_3$) 1.40 – 1.55 (9H, s, Me_3C), 1.65 (1H, dq, J 11.9, 7.8, NCH_2CHH), 2.18 – 2.32 (1H, m, NCH_2CHH), 2.72 (1H, ddd, J 9.7, 8.3, 6.5, NCH*H*), 2.78 (1H, ddd, J 10.8, 7.1, 4.2, NC*H*H), 3.14 (1H, tdd, J 8.0, 4.2, 1.9, C*H*), 5.61 (1H, d, J 9.6, =C*H*-C_q), 5.79 (0.75H, ddt, J 9.6, 4.1, 1.1, =C*H*/D=CH_2), 5.84 – 5.89 (1H, m, =CH) and 5.97 (1H, dd, J 9.4, 5.6, =CH); δ_C (126 MHz, $CDCl_3$) 27.9 (Me_3C), 35.1 (NCH_2CH_2), 40.8 (CH_{minor}), 40.9 (CH_{major}), 42.023 (NCH_2), 66.2 2 (C_q-CO_2^{t}Bu), 81.4 (Me_3*C*), 119.424 (=CH_{minor}), 119.565 (=CH_{major}), 122.515 (=CH), 126.0 (=CH), 129.1 (t, J 24.8, =CD), 129.5 (=CH) and 174.654 (CO_2^{t}Bu).

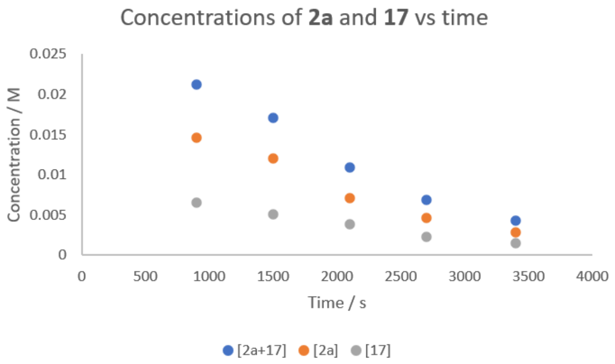

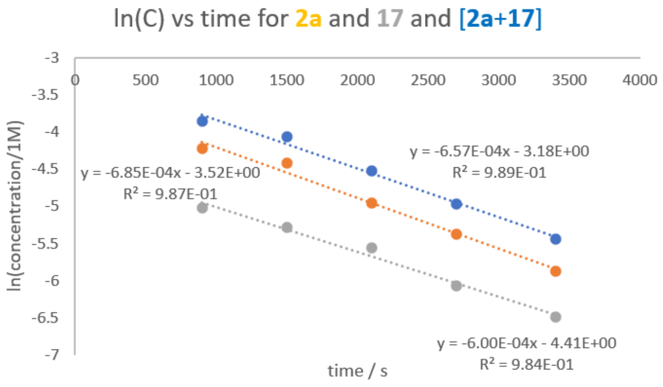

$k_{2a} = 6.8 \pm 0.5 \times 10^{-4}\ s^{-1}$

$k_{17} = 6.0 \pm 0.4 \times 10^{-4}\ s^{-1}$

$k_H/k_D = 1.1 \pm 0.1$

Synthesis of Deuterated Labelled Compounds & Mechanistic Investigations

(±)-*tert*-Butyl (3a*S*,7a*S*)-5-acetoxy-1,2,3,3a,4,5-hexahydro-7a*H*-indole-7a-carboxylate 16

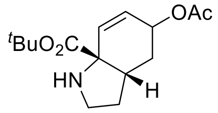

16

To a stirred solution of substrate **2a** (120 mg, 0.54 mmol), $Pd_2(dba)_3$ (15 mg, 0.016 mmol) and triphenylphosphite (0.036 mL, 0.14 mmol) in dry CH_2Cl_2 at rt under nitrogen was added AcOH (0.15 mL). After stirring for 16 h the reaction was poured onto sat. aq. $NaHCO_3$ (15 mL) and extracted with CH_2Cl_2 (2 × 10 mL). Drying ($MgSO_4$) and evaporation gave a grey oil. Purification by silica gel chromatography (EtOAc as eluent) afforded the title compound (86 mg, 57%) as a light-yellow oil. ^{1}H-NMR and ^{13}C-NMR data agrees with the literature.[8]

(Methyl-^{13}C-d_3)triphenylphosphonium iodide S4

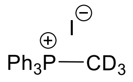

S4

To a stirred suspension of triphenylphosphine (11.8 g, 45 mmol) in anhydrous THF (55 mL) at rt were added d_3-iodomethane (5.5 g, 35.9 mmol) and d_3-^{13}C-iodomethane (1.06 g, 7.26 mmol) dropwise. The reaction was sealed and stirred at 60 °C for 3 h, cooled to rt, stirred for 14 h and filtered. The solid was washed with Et_2O (2 × 15 mL) and dried to afford the title compound (18.15 g, 99%) as a white solid. Spectral properties were identical to those reported.[21]

But-3-en-4,4-d_2-1-ol S6

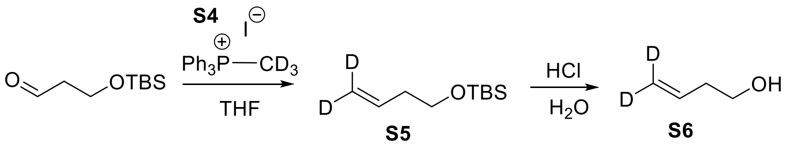

To a stirred suspension of phosphonium salt **S4** (13.8 g, 33.9 mmol) in anhydrous THF (120 mL) at 0 °C under nitrogen was added KOtBu (4.00 g, 35.7 mmol) portionwise over 5 min. The resulting yellow solution/suspension was warmed to rt, stirred for 90 min, cooled to -60 °C and a solution of 3-(tert-butyldimethylsiloxy)propionaldehyde (6.45 g, 34 mmol) in anhydrous THF (15 mL) was added dropwise over 10 min. The reaction was allowed to warm to rt, stirred for 14 h, diluted with petrol (250 mL) and cooled to -10 °C. The mixture was stirred for 15 min, filtered, and the solid washed with Et_2O/pentane (1:9, 100 mL) and the resulting solution evaporated to give an orange oil, **S5**. To a stirred solution of substrate **S5** (<34 mmol) in THF (8 mL) at rt was added 3 M aq. HCl (4 mL, 12 mmol) dropwise. The reaction was stirred for 2 h, diluted with brine (4 mL) and extracted with Et_2O (4 × 5 mL). Drying ($MgSO_4$) and careful concentration to ca. 7 mL gave a clear solution which was diluted with pentane (7 mL) and passed through a silica plug (Et_2O/pentane, 1:4 to 3:2 as eluent). Partial evaporation gave a clear oil (2.32 g). ^{1}H NMR suggested 1.75 mmol product, 5% over two steps, which was used directly in the next step.

1-(1-(But-3-en-1-yl-4,4-*d*$_2$)-1*H*-pyrrol-2-yl)-2,2,2-trichloroethan-1-one S7

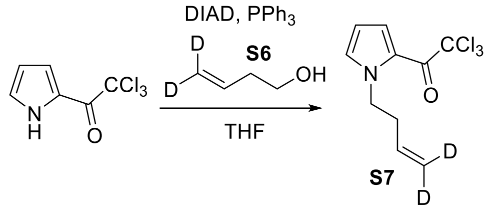

To a stirred solution of triphenylphosphine (524 mg, 2.00 mmol) in anhydrous THF (10 mL) at -78 °C under nitrogen was added DIAD (0.405 mL, 2.06 mmol) dropwise. The yellow reaction was stirred for 1 h and a solution of substrate **S6** (1.75 mmol) in anhydrous THF (2 mL) was added dropwise to the resulting suspension. After stirring for a further 1 h 2-(trichloroacetyl)pyrrole (425 mg, 2.00 mmol) was added in one portion. The reaction was stirred for 90 min and warmed to rt. After stirring for a further 21 h the mixture was evaporated and the resulting yellow oil triturated in Et_2O/petrol (2:3, 10 mL) at -10 °C. Filtration and evaporation gave a yellow oil which was purified by silica gel chromatography (Et_2O/petrol, 2:98 to 3:97 as eluent) to afford the title compound (240 mg, 51%) as a clear oil. δ_H (500 MHz, $CDCl_3$) 2.53 (2H, q, J 7.0, NCH_2CH_2), 4.41 (2H, t, J 7.1, NCH_2), 5.04 – 5.11 (0.4H, m, residual =C*H*D), 5.78 (1H, m, C*H*=CD_2), 6.24 (1 H, dd, J 4.4, 2.4, pyrrole CH), 7.02 (1 H, t, J 2.0, pyrrole CH) and 7.56 (1 H, dd, J 4.5, 1.6, pyrrole CH); δ_C (126 MHz, $CDCl_3$) 35.3 (q, J 6.2, NCH_2CH_2), 50.1 (NCH_2), 96.5 (CCl_3), 108.9 (pyrrole CH), 117.3 (pent, J 23.8, =CD_2), 117.5 (t, J 23.8, =CHD), 117.8 (s, =CH_2), 121.0 (Cq), 124.7 (pyrrole CH), 133.2 (pyrrole CH), 133.5 – 134.1 (m , *C*H=) and 172.6 (*C*OCCl_3).

(±)-*tert*-Butyl 1-(but-3-en-1-yl-4,4-*d*$_2$)-1*H*-pyrrole-2-carboxylate S8

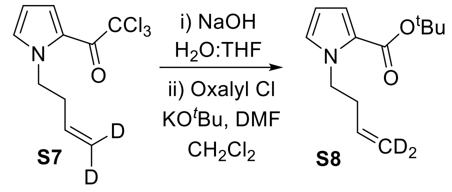

To a stirred solution of pyrrole **S7** (238 mg, 0.89 mmol) in THF (2 mL) at rt was added 2 M aq. NaOH (2 mL, 4 mmol) dropwise. The reaction was stirred for 14 h, cooled to 0 °C, diluted with CH_2Cl_2 (7 mL) and 3 M aq. HCl (2 mL) was added dropwise. The phases were separated, the aqueous phase extracted with CH_2Cl_2 (10 mL) and the combined organic phase dried ($MgSO_4$) and evaporated to give a pale-yellow oil which solidified on standing. This was dissolved in anhydrous CH_2Cl_2 (5 mL) at 0 °C under nitrogen and oxalyl chloride (100 µL, 1.16 mmol) was added dropwise over 3 min. The reaction was warmed to rt, stirred for 1 h and cooled to 0 °C. Potassium *tert*-butoxide (295 mg, 2.63 mmol) was added portionwise over 2 min, the reaction warmed to rt, stirred for 3.5 h and quenched with water (20 mL). The mixture was extracted with CH_2Cl_2 (2 × 15 mL) and the combined organic phase washed with brine (15 mL), dried ($MgSO_4$) and evaporated to give a brown oil. Purification by silica gel chromatography (Et_2O/petrol, 2% to 3% as eluent) afforded the title compound (128 mg, 65%) as a clear oil. δ_H (500 MHz, $CDCl_3$) 1.58 (9H, s, *Me*$_3$C), 2.44 – 2.59 (2H, m, NCH_2CH_2), 4.36 (2H, t, J 7.2, NCH_2), 4.97 – 5.09 (0.4H, m, residual =C*H*D), 5.77 (1H, m, C*H*=CD_2), 6.10 (1H, dd, J 3.9, 2.5, pyrrole CH), 6.79 (1H, dd, J 2.5, 1.9, pyrrole CH) and 6.90 (1H, dd, J 3.9, 1.9, pyrrole CH); δ_C (126 MHz, $CDCl_3$) 28.4 (Me_3C), 35.83 – 35.89 (m NCH_2CH_2), 48.7 (NCH_2), 80.2 (*C*Me_3), 107.4 (pyrrole CH), 116.5 (pent, J 23.9, =CD_2) 116.8 (t, J 23.8, =CHD), 117.1 (=CH_2), 117.9 (pyrrole CH), 123.2 (pyrrole C_q), 128.2 (pyrrole CH), 134.2 – 134.9 (m, *C*H=CD_2) and 160.6 (CO_2tBu).

(±)-*tert*-Butyl (3^1*S*,3a*R*,6a*R*)-1,3a,6,6a-tetrahydroazirino[2,3,1-*hi*]indole-3^1(2*H*)-carboxylate-6,6-*d*$_2$ 17

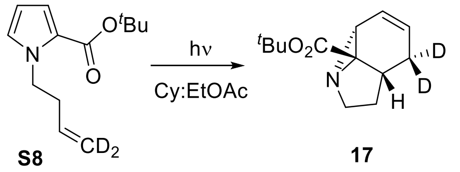

A stirred solution of substrate **S8** (120 mg, 0.54 mmol) in EtOAc (70 mL) and cyclohexane (400 mL) was degassed and transferred to a photochemical reactor under nitrogen. The solution was irradiated with a 36 W UVC lamp (approx. 50% immersion) for 70 min with stirring and evaporated to give a light-yellow oil. Purification by silica gel chromatography (EtOAc/petrol, 1:9 to 1:1 as eluent) afforded the title compound (39 mg, 33%) as a pale-yellow oil. δ_H (500 MHz, $CDCl_3$) 1.41 – 1.57 (10H, m, *Me*$_3$C and NCH_2CHH), 1.89 – 1.94 (0.2H, m, residual allylic C*H*D), 2.27 – 2.32 (0.2H, m, residual allylic CD*H*), 2.49 – 2.62 (2H, m, NCH_2C*H*H and NC*H*HCH_2), 2.86 (1H, dd, J 3.9, 1.2, NCH), 3.18 – 3.27 (2H, m, NC*H*HCH_2 and CH_2C*H*CH_2), 5.81 (1H, dt, J 10.0, 3.2, =C*H*-CH_2) and 6.21 – 6.26 (1H, m, C*H*=CH-CH_2); δ_C (126 MHz, $CDCl_3$) 28.1 (*Me*$_3$C), 28.802 (pent, J 19.5, =CD_2), 29.2 (t, J 19.5, =CHD), 29.6 (s, =CH_2), 33.14 – 33.56 (m, CH_2*C*HCH_2), 33.404, 41.07 – 41.27 (m, NCH_2CH_2), 43.5 (app t, J 3.2, NCH), 49.9 (N*C*H_2), 52.4 (*C*$_q$-CO_2tBu), 81.226 (Me_3*C*O), 120.68 – 120.84 (m, =*C*H-CD_2), 134.82 – 135.11 (*C*H=CH-CD_2) and 171.7 (*C*O_2^{t}Bu).

Functionalization of Products

(±)-*tert*-Butyl (4*R*,4a*R*,4a[1]*R*,9*S*,9a*S*)-2-benzyldecahydro-4a[1]*H*-4,9-methanoazepino[3,4,5-gh]pyrrolizine-4a[1]-carboxylate 21

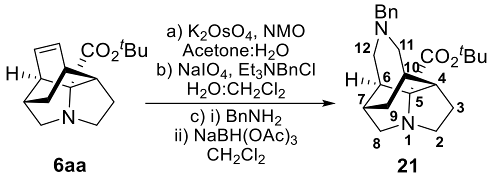

6aa 21

N-methyl morpholine N-oxide (0.070 g, 0.6 mmol, 1.5 eq.) and potassium osmate (VI) dihydrate (2.9 mg, 2 mol%) were added to a solution of tert-butyl 1,4,5,5a,8,8a-hexahydro-5,8-methanopyrrolo[3,2,1-*hi*]indole-3[1](2*H*)-carboxylate (0.104 g, 0.4 mmol, 1 eq.) in acetone (4 mL) and H_2O (0.4 mL). The mixture was stirred vigorously for 16 h. Sat. aq. sodium sulfite solution (5 mL) was added and acetone removed under reduced pressure. The mixture was extracted with CH_2Cl_2 (5 × 5 mL) and the combined organic layers were dried over $MgSO_4$ and concentrated under reduced pressure. The crude diol was dissolved in CH_2Cl_2 (0.8 mL) and H_2O (2 mL) under a nitrogen atmosphere. To this Et_3BnNCl (5 mg) and $NaIO_4$ (0.090 g, 0.42 mmol, 1.05 eq.) were added and the resulting mixture was stirred for 1 h. Then the layers were separated and the aqueous layer was extracted with CH_2Cl_2 (3 × 3 mL). The combined organic layers were dried over Na_2SO_4 and concentrated under reduced pressure to a volume of about 5 mL. To this solution of crude dialdehyde was added benzylamine (0.052 mL, 0.48 mmol, 1.2 eq.) under a nitrogen atmosphere. The mixture was stirred for 15 min, then $NaBH(OAc)_3$ (0.266 g, 1.2 mmol, 3 eq.) was added and the reaction was stirred for a further 1.5 h. The reaction was quenched by addition of sat. aq. Na_2CO_3 solution (ca 10 mL). The layers were separated and the aqueous layer was extracted with CH_2Cl_2 (5 × 10 mL). The combined organic layers were dried over $MgSO_4$ and concentrated under reduced pressure. The crude product was purified over silica gel (5% 1:8 NH_3:EtOH in CH_2Cl_2) to give a pale yellow oil (89 mg, 60% over 3 steps). ν_{max}/cm^{-1} 2923, 2865, 1720, 1454, 1365, 1254, 1160; ^1H NMR (400 MHz, $CDCl_3$) δ_H 7.28 (d, *J* = 4.4 Hz, 4H, ArCH), 7.25 – 7.19 (m, 1H, ArCH), 3.66 (d, *J* = 13.3 Hz, 1H, PhCH), 3.50 (ddd, *J* = 11.1, 5.4, 1.3 Hz, 1H, 8-CH), 3.32 (d, *J* = 13.3 Hz, 1H, PhCH), 3.08 – 2.96 (m, 3H, 2-CH, 4-CH, 12-CH), 2.91 – 2.75 (m, 3H, 2-CH, 6-CH, 11-CH), 2.51 (dd, *J* = 12.2, 1.6 Hz, 1H, 12-CH), 2.20 – 2.09 (m, 2H, 3-CH, 7-CH), 2.06 (d, *J* = 11.1 Hz, 1H, 8-CH), 2.03 – 1.95 (m, 1H, 9-CH), 1.91 (dd, *J* = 10.9, 1.3 Hz, 1H, 11-CH), 1.75 – 1.54 (m, 3H, 3-CH, 9-CH, 10-CH), 1.50 (s, 9H, $C(CH_3)_3$). ^{13}C NMR (101 MHz, $CDCl_3$) δ_C 173.7 (CO), 139.6 (ArC), 128.8 (ArCH), 128.3 (ArCH), 126.9 (ArCH), 80.3 ($\underline{C}(CH_3)_3$, 78.6 (5-C), 64.7 (8-CH_2), 63.9 (11-CH_2), 63.1 ($PhCH_2$), 54.5 (2-CH_2), 53.5 (12-CH_2), 46.1 (6-CH), 44.1 (4-CH), 36.9 (7-CH), 35.1 (9-CH_2), 34.4 (10-CH), 31.9 (3-CH_2), 28.3 ($C(\underline{C}H_3)_3$). HRMS (ESI^+) m/z: $[M+H]^+$ Calcd for $C_{23}H_{33}N_2O_2$ 369.2537; Found 369.2549.

(±)-(1*R*,3[1]*R*,5a*R*,6*S*,8a*R*)-*N*[1]-benzyl-*N*[3](4-methoxybenzyl)-5,5a,6,8a-tetrahydro-1,6-methanopyrrolo[3,2,1-*hi*]indole-1,3[1](2*H*,4*H*)-dicarboxamide 22

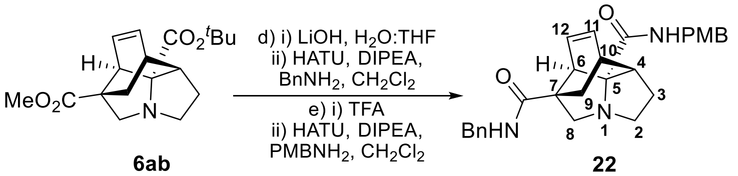

6ab 22

LiOH (0.029 g, 1.2 mmol, 2 eq.) was added to a solution of substrate (0.192 g, 0.6 mmol, 1eq.) in 1:1 THF/H_2O (6 mL). The reaction was stirred at RT for 16 h. The mixture was neutralized with 1M HCl (1.2 mL) and concentrated under reduced pressure. Any remaining H_2O was removed by repeated co-evaporation with toluene. CH_2Cl_2 was added to the residue and the mixture was filtered through Celite® and evaporated to dryness *in vacuo*. The crude acid was dissolved in dry CH_2Cl_2 (6 mL) under a nitrogen atmosphere and DIPEA (0.209 mL, 1.2 mmol, 2 eq.) and HATU (0.228 g, 0.6 mmol, 1 eq.) were added. The mixture was stirred for 5 min before adding benzylamine (0.072 mL, 0.66 mmol, 1.1 eq.) and stirring for a further 6 h. H_2O (10 mL) was added, the layers were separated and the aqueous layer was extracted with CH_2Cl_2 (4 × 10 mL). The combined organic layers were washed with 1N HCl (20 mL) and the aqueous layer back extracted with CH_2Cl_2 (2 × 10 mL). The combined organic layers were washed with brine (30 mL), dried over $MgSO_4$ and concentrated under reduced pressure. The residue was dissolved in $CHCl_3$ (2 mL) and the precipitate filtered off. The crude product was purified over silica gel (3 - 5% 1:8 NH_3:EtOH in CH_2Cl_2) and any remaining tetramethylurea removed by repeated co-evaporation with toluene, to give a colourless solid (0.152 g, 64%). m.p.: 131 – 133 ˚C; ν_{max}/cm^{-1} 3307, 2966, 2859, 1717, 1642, 1552, 1292, 1233, 1164; ^1H NMR (400 MHz, $CDCl_3$) δ_H 7.35 – 7.23 (m, 3H, ArCH), 7.23 – 7.15 (m, 2H, ArCH), 6.58 (ddd, *J* = 8.0, 6.6, 1.2 Hz, 1H, 11-CH), 6.23 (ddd, *J* = 7.8, 6.4, 1.2 Hz, 1H, 12-CH), 5.73 (br s, 1H, NH), 4.38 (d, *J* = 5.6 Hz, 2H, $PHCH_2$), 3.76 (dd, *J* = 11.5, 1.5 Hz, 1H, 8-CH), 3.48 (dd, *J* = 6.4, 1.2 Hz, 1H, 6-CH), 3.42 (dt, *J* = 12.6, 8.9 Hz, 1H, 2-CH), 3.06 (ddd, *J* = 12.5, 9.1, 3.1 Hz, 1H, 2-CH), 2.55 (d, *J* = 11.6 Hz, 1H, 8-CH), 2.52 – 2.45 (m, 1H, 10-CH), 2.31 – 2.23 (m, 1H, 4-CH), 2.07 (dtd, *J* = 12.7, 9.4, 3.2 Hz, 1H, 3-CH), 1.84 (dd, *J* = 13.7, 3.1 Hz, 1H, 9-CH), 1.70 – 1.53 (m, 2H, 3-CH, 9-CH), 1.42 (s, 9H, $C(CH_3)_3$). ^{13}C

NMR (101 MHz, $CDCl_3$) δ_C 174.3 (CO), 173.3 (CO), 139.2 (11-CH), 138.4 (ArC), 128.9 (ArCH), 128.4 (12-CH), 127.6 (ArCH), 81.7 (<u>C</u>(CH_3)$_3$), 81.1 (5-C), 66.7 (8-CH_2), 58.7 (2-CH_2), 51.2 (7-CH), 49.6 (4-CH), 46.7 (6-CH), 43.5 ($PhCH_2$), 36.0 (9-CH_2), 35.8 (10-CH), 28.3 (3-CH_2), 28.1 (C(<u>C</u>H_3)$_3$). HRMS (ESI^+) m/z: $[M+H]^+$ Calcd for $C_{24}H_{31}N_2O_3$ 395.2329; Found 395.2343.

The substrate (0.100 g, 0.25 mmol, 1eq.) was dissolved in TFA (1.5 mL), the solution stirred for 4 h and the reaction concentrated under reduced pressure. The crude acid was dissolved in dry CH_2Cl_2 (2.5 mL) under a nitrogen atmosphere and DIPEA (0.088 mL, 0.51 mmol, 2 eq.) and HATU (0.106 g, 0.28 mmol, 1.1 eq.) were added. The mixture was stirred for 5 min before adding 4-methoxybenzylamine (0.040 mL, 0.30 mmol, 1.2 eq.) and stirring for a further 14 h. H_2O (5 mL) was added, the layers were separated and the aqueous layer was extracted with CH_2Cl_2 (4 × 5 mL). The combined organic layers were washed with 1N HCl (10 mL) and the aqueous layer back extracted with CH_2Cl_2 (2 × 5 mL). The combined organic layers were washed with brine (20 mL), dried over $MgSO_4$ and concentrated under reduced pressure. The crude product was purified over silica gel (5% 1:8 NH_3:EtOH in CH_2Cl_2) and any remaining tetramethylurea removed by repeated co-evaporation with toluene, to give a colourless solid (0.085 g, 73%). m.p.: 191 – 193 ˚C; ν_{max}/cm^{-1} 3289, 2925, 2832, 1647, 1509, 1303, 1243; ^1H NMR (400 MHz, $CDCl_3$) δ_H 8.02 (br t, *J* = 5.8 Hz, 1H, NH), 7.37 – 7.23 (m, 3H, ArCH), 7.23 – 7.11 (m, 4H, ArCH), 6.91 – 6.81 (m, 2H, ArCH), 6.70 (ddd, *J* = 7.9, 6.6, 1.3 Hz, 1H, 11-CH), 6.25 (ddd, *J* = 7.8, 6.4, 1.2 Hz, 1H, 12-CH), 5.80 (br t, *J* = 5.8 Hz, 1H, NH), 4.42 – 4.23 (m, 4H, 2 × $PhCH_2$), 3.80 (s, 3H, OCH_3), 3.77 (d, *J* = 13.1 Hz, 1H, 8-CH), 3.14 (dd, *J* = 6.4, 1.2 Hz, 1H, 6-CH), 3.02 (ddt, *J* = 18.1, 12.9, 9.1 Hz, 2H, 2-CH_2), 2.70 – 2.49 (m, 3H, 4-CH, 8-CH, 10-CH), 2.04 – 1.83 (m, 2H, 3-CH, 9-CH), 1.69 – 1.50 (m, 2H, 3-CH, 9-CH) ^{13}C NMR (101 MHz, $CDCl_3$) δ_C 174.2 (CO), 174.0 (CO), 159.0 (ArC), 139.5 (11-CH), 138.4 (ArC), 130.8 (ArC), 129.0 (ArCH), 128.8 (ArCH), 127.5 (ArCH), 127.5 (12-CH), 127.5 (ArCH), 114.2 (ArCH), 81.7 (5-C), 65.3 (8-CH_2), 57.2 (2-CH_2), 55.4 (OCH_3), 52.1 (7-CH), 49.4 (6-CH), 47.1 (4-CH), 43.4 $PhCH_2$), 42.8 ($PhCH_2$), 36.3 (9-CH_2), 35.9 (10-CH), 28.1 (3-CH_2). HRMS (ESI^+) m/z: $[M+H]^+$ Calcd for $C_{28}H_{32}N_3O_3$ 458.2438; Found 458.2446.

Crystallographic Data

Table S3. Crystallographic Data of **6cd** (thermal ellipsoid is set at 50% probability)
Recrystallisation was achieved through slow cooling of a saturated solution in refluxing Et_2O.

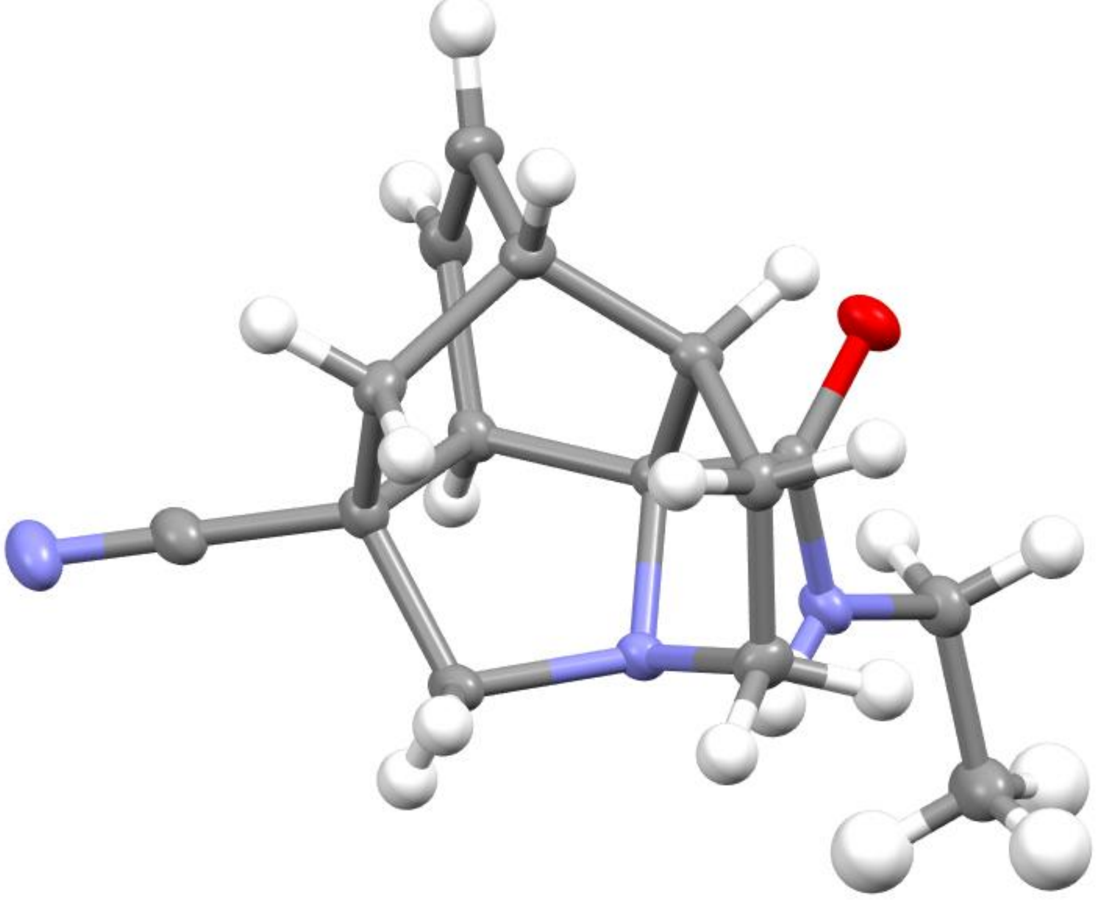

Empirical formula	$C_{15}H_{19}N_3O$
Formula weight	257.33
Temperature/ K	100(2)
Crystal system	monoclinic
Space Group	$P2_1/n$
a/Å	11.5420(4)
b/Å	9.8187(4)
c/ Å	12.5471(5)
α/°	90
β/°	111.549(2)
γ/°	90
Volume/Å^3	1322.54(9)
Z	4
ρ_{calc}g/cm^3	1.292
μ/mm^{-1}	0.083
F(000)	552.0
Crystal size/mm^3	0.444 × 0.378 × 0.332
Radiation	MoKα (λ = 0.71073)
2θ range for data collection/°	4.104 to 55.896
Index ranges	$-13 \leq h \leq 15$, $-12 \leq k \leq 12$, $-16 \leq l \leq 10$
Reflections collected	11836
Independent reflections	3160 [R_{int} = 0.0343, R_{sigma} = 0.0309]
Data/restraints/parameters	3160/0/177
Goodness-of-fit on F^2	1.044
Final R indexes [I>=2σ (I)]	R_1 = 0.0399, wR_2 = 0.0969
Final R indexes [all data]	R_1 = 0.0517, wR_2 = 0.1041
Largest diff. peak/hole/ e Å^{-3}	0.35/-0.22

References

1. K. G. Maskill, J. P. Knowles, L. D. Elliott, R. W. Alder, K. I. Booker-Milburn, *Angew. Chem. Int. Ed.* **2013**, *52*, 1499–1502.
2. E. E. Blackham, K. I. Booker-Milburn, *Angew. Chem. Int. Ed.* **2017**, *56*, 6613–6616.
3. Bruker, *SAINT+ v8.38A Integration Engine, Data Reduction Software, Bruker Analytical X-ray Instruments Inc., Madison, WI, USA*, 2015.
4. Bruker, *SADABS 2014/5, Bruker AXS area detector scaling and absorption correction, Bruker Analytical X-ray Instruments Inc., Madison, Wisconsin, USA*, 2014/5.
5. G. M. Sheldrick, *Acta Crystallogr. Sec. A Founda. Adv*. **2015**, *71*, 3-8.
6. G. M. Sheldrick, *Acta Crystallogr. Sect. A Found. Crystallogr.* **2008**, *64*, 112-122.
7. G. M. Sheldrick, *Acta Crystallogr. Sect. C. Struct. Chem.* **2015**, *71*, 3-8.
8. O. V. Dolomanov, L. J. Bourhis, R. J. Gildea, J. A. K. Howard, H. J. Puschmann, *Appl. Crystallogr*. **2009**, *42*, 339-341.
9. P. Magnus, T. Gallagher, P. Brown, J. C. Huffmann, *J. Am. Chem. Soc.* **1984**, *106*, 2105-2114.
10. W. Heinz, H. W. Gschwend, A. O. Lee, *J. Org. Chem.* **1973**, *38*, 2169-2175.
11. H. V. Pham, R. S. Paton, A. G. Ross, S. J. Danishefsky, K. N. Houk, *J. Am. Chem. Soc.* **2014**, *136*, 2397-2403.
12. K. S. Khuong, C. M. Beaudry, D. Trauner, K. N. Houk, *J. Am. Chem. Soc.* **2005**, *127*, 3688-3689.
13. W. J. Vloon, J. C. Van den Bos, G.-J. Koomen, U. K. Pandit, *Tetrahedron*, **1992**, *48*, 8317-8328.
14. E. M. István, T. S. Florian, *Beilstein J. Org. Chem.* **2013**, *9*, 1319-1325.
15. S. E. Denmark, Z. D. Matesich, *J. Org. Chem.* **2014**, *79*, 5970-5986.
16. R. Csuk, U. Höring, M. Schaade, *Tetrahedron* **1996**, *52*, 9759-9776.
17. D. Scharnagel, A. Müller, F. Prause, M. Eck, J. Goller, W. Milius, M. Breuning, *Chem. Eur. J.* **2015**, *21*, 12488-12500.
18. W. H. Henderson C. T. Check, N. Proust J. P. Stambuli, *Org. Lett.* **2010**, *12*, 824-827.
19. K. Asano, S. Matsubara, *Synthesis* **2009**, 3219-3226.
20. R. T. H. Linstadt, C. A. Peterson, C. I. Jette, Z. V. Boskovic, B. H. Lipshutz, *Org. Lett.* **2017**, *19*, 328-331.
21. J. J. Gajewski, K. B. Peterson, J. R. Kagel, Y. C. J. Huang, *J. Am. Chem. Soc*. **1989**, *111*, 9078-9081.

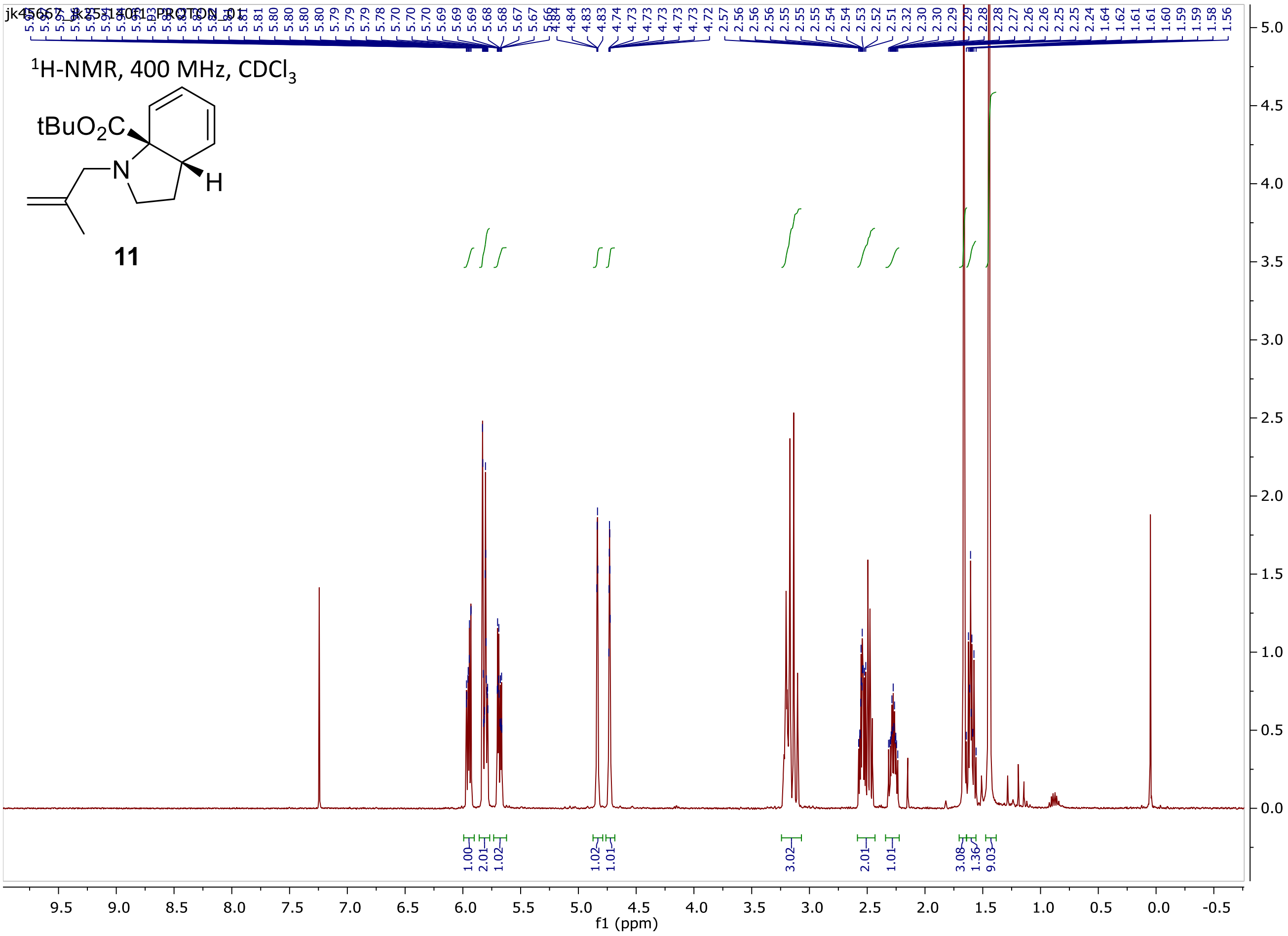

jk45667_jk25-140f1_PROTON_01
^{1}H-NMR, 400 MHz, $CDCl_3$
tBuO$_2$C
N
H
11
1.00
2.01
1.02
1.02
1.01
3.02
2.01
1.01
3.08
1.36
9.03
f1 (ppm)

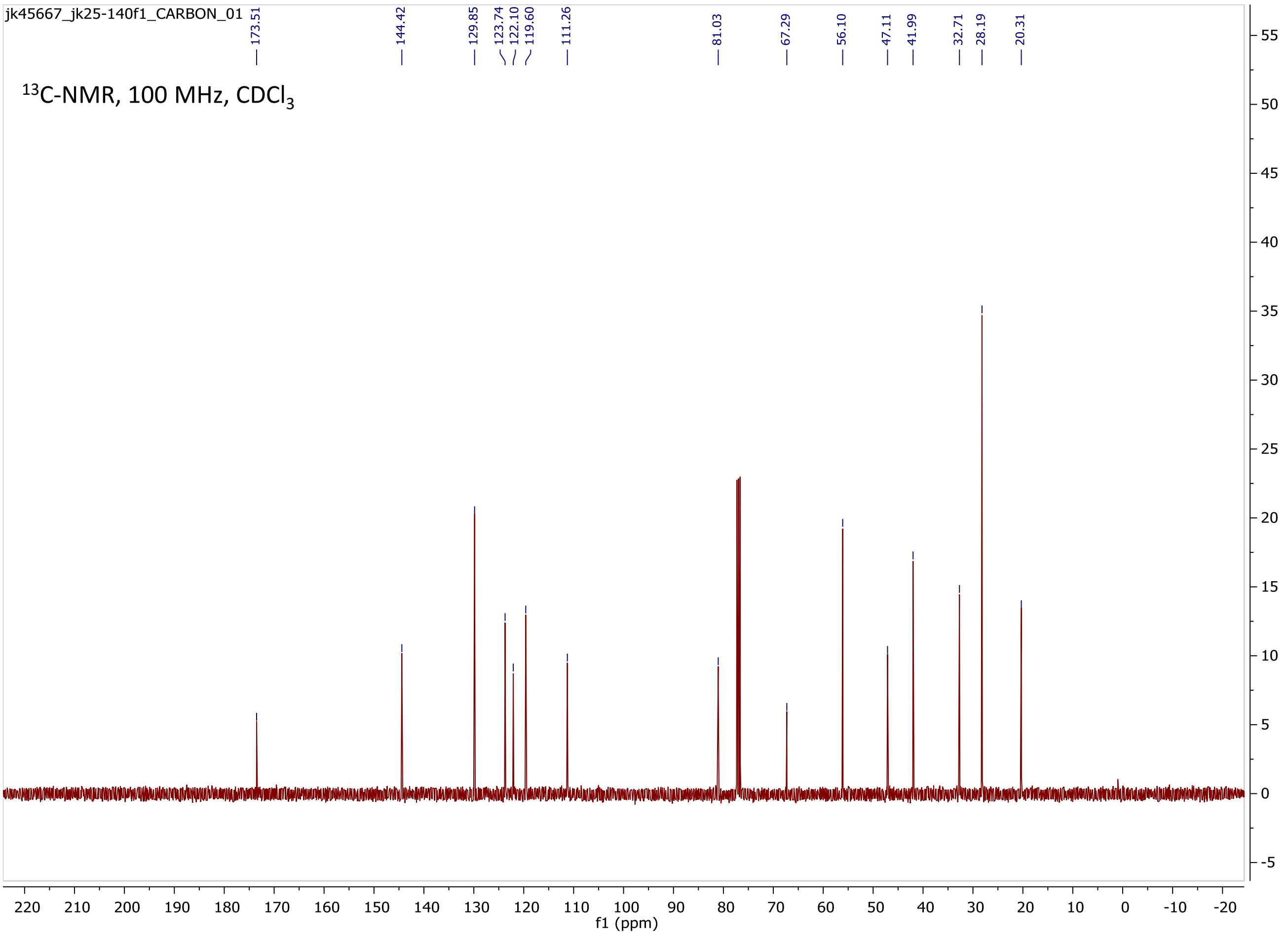

jk45667_jk25-140f1_CARBON_01
^{13}C-NMR, 100 MHz, $CDCl_3$
173.51
144.42
129.85
123.74
122.10
119.60
111.26
81.03
67.29
56.10
47.11
41.99
32.71
28.19
20.31
f1 (ppm)

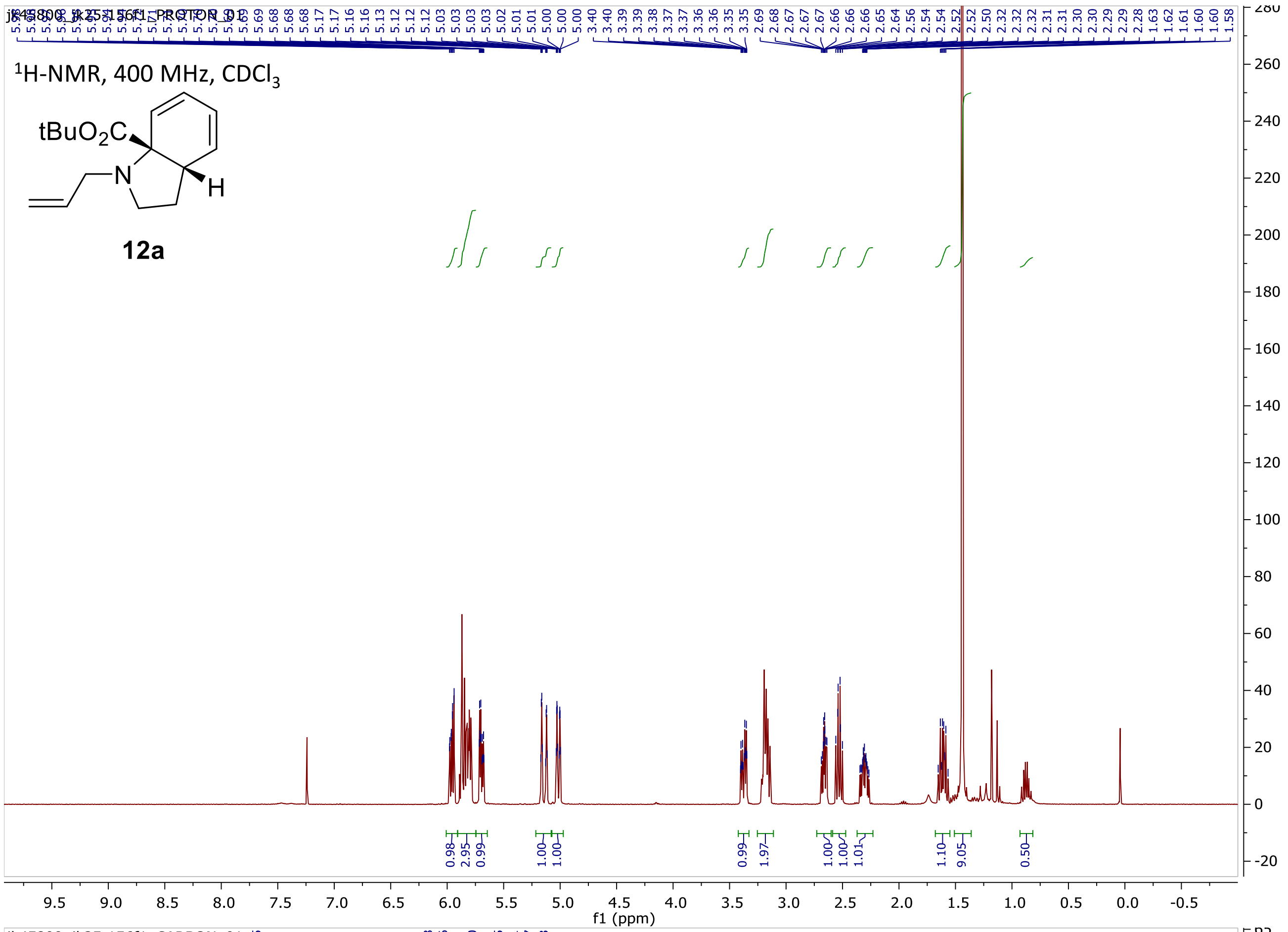
jk45800_jk25-156f1_PROTON_01
1H-NMR, 400 MHz, CDCl3
tBuO2C
N
H
12a
f1 (ppm)

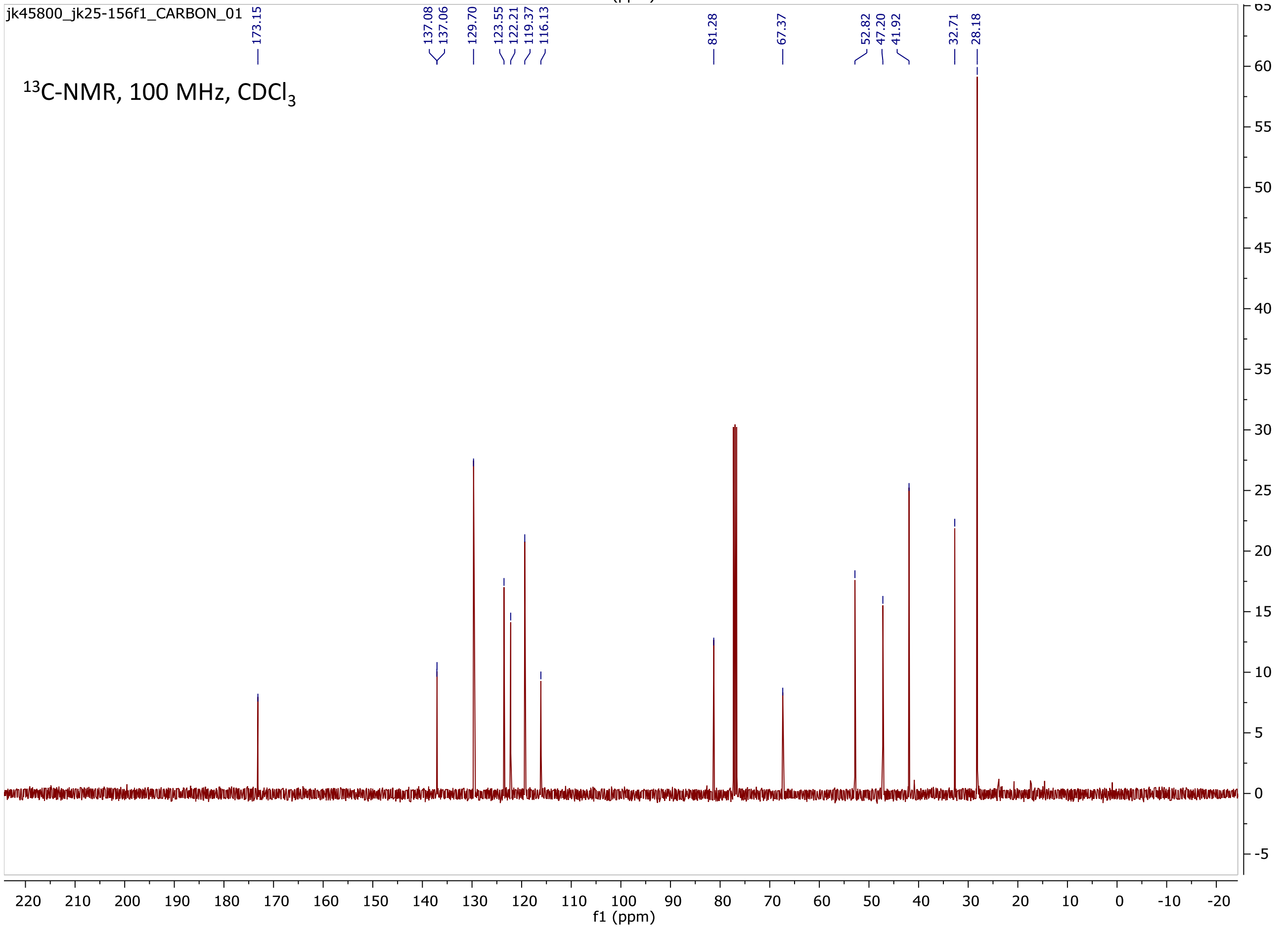
jk45800_jk25-156f1_CARBON_01
13C-NMR, 100 MHz, CDCl3
173.15
137.08
137.06
129.70
123.55
122.21
119.37
116.13
81.28
67.37
52.82
47.20
41.92
32.71
28.18
f1 (ppm)

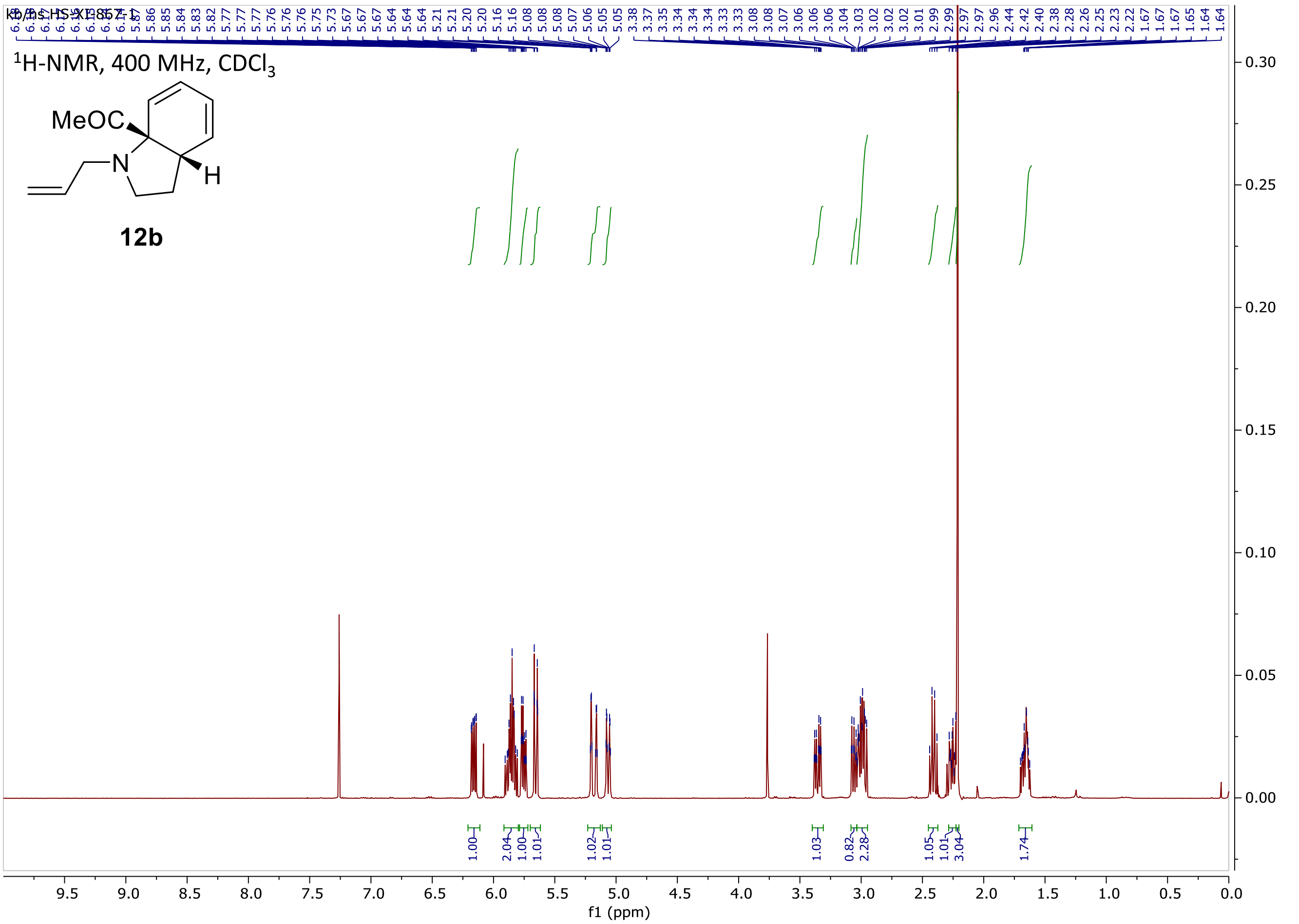
kb/hs HS-X1-867-1
^{1}H-NMR, 400 MHz, $CDCl_3$
MeOC
N
H
12b
f1 (ppm)

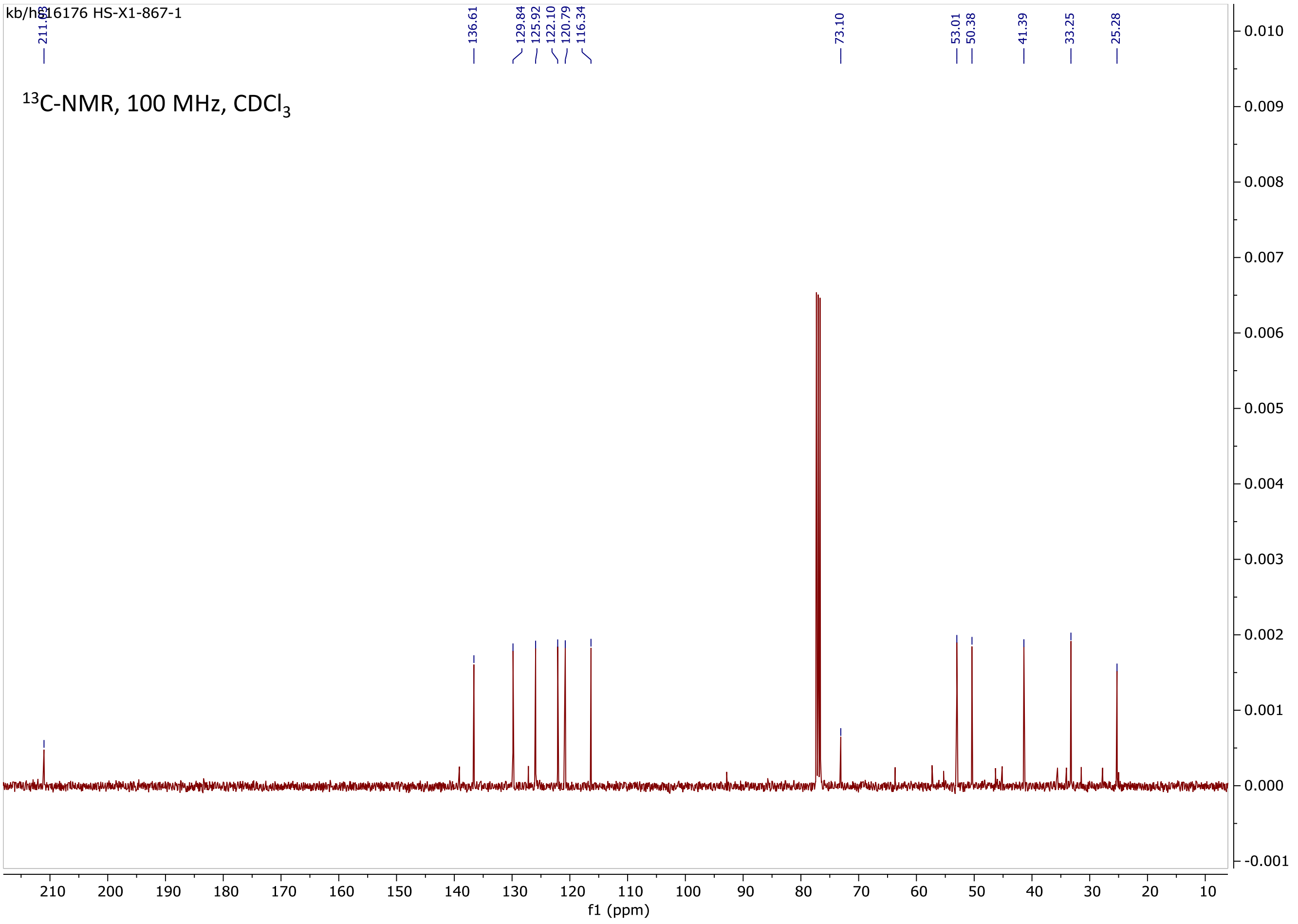
kb/hs16176 HS-X1-867-1
^{13}C-NMR, 100 MHz, $CDCl_3$
136.61
129.84
125.92
122.10
120.79
116.34
73.10
53.01
50.38
41.39
33.25
25.28
f1 (ppm)

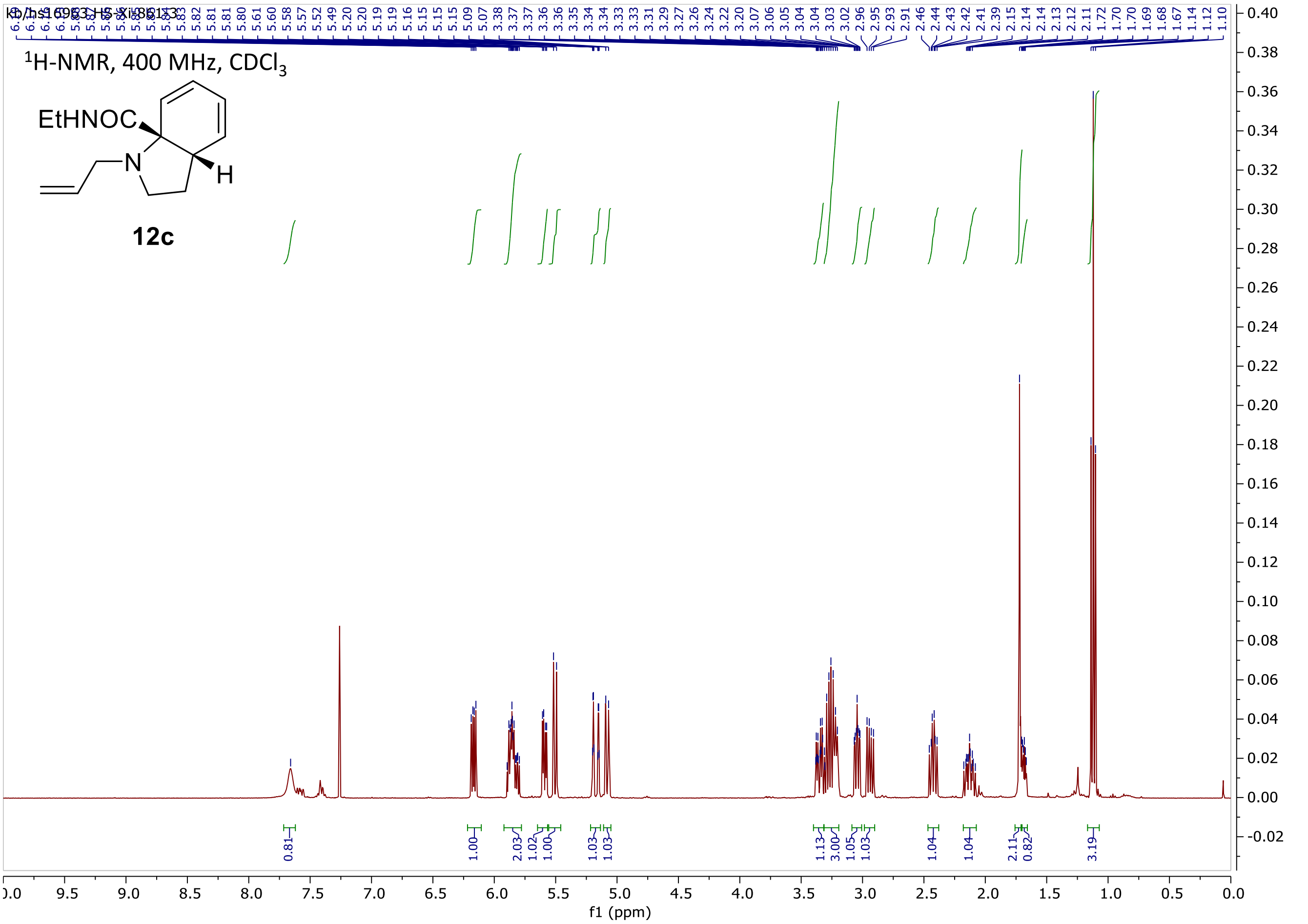

¹H-NMR, 400 MHz, CDCl3
EtHNOC
N
H
12c
f1 (ppm)

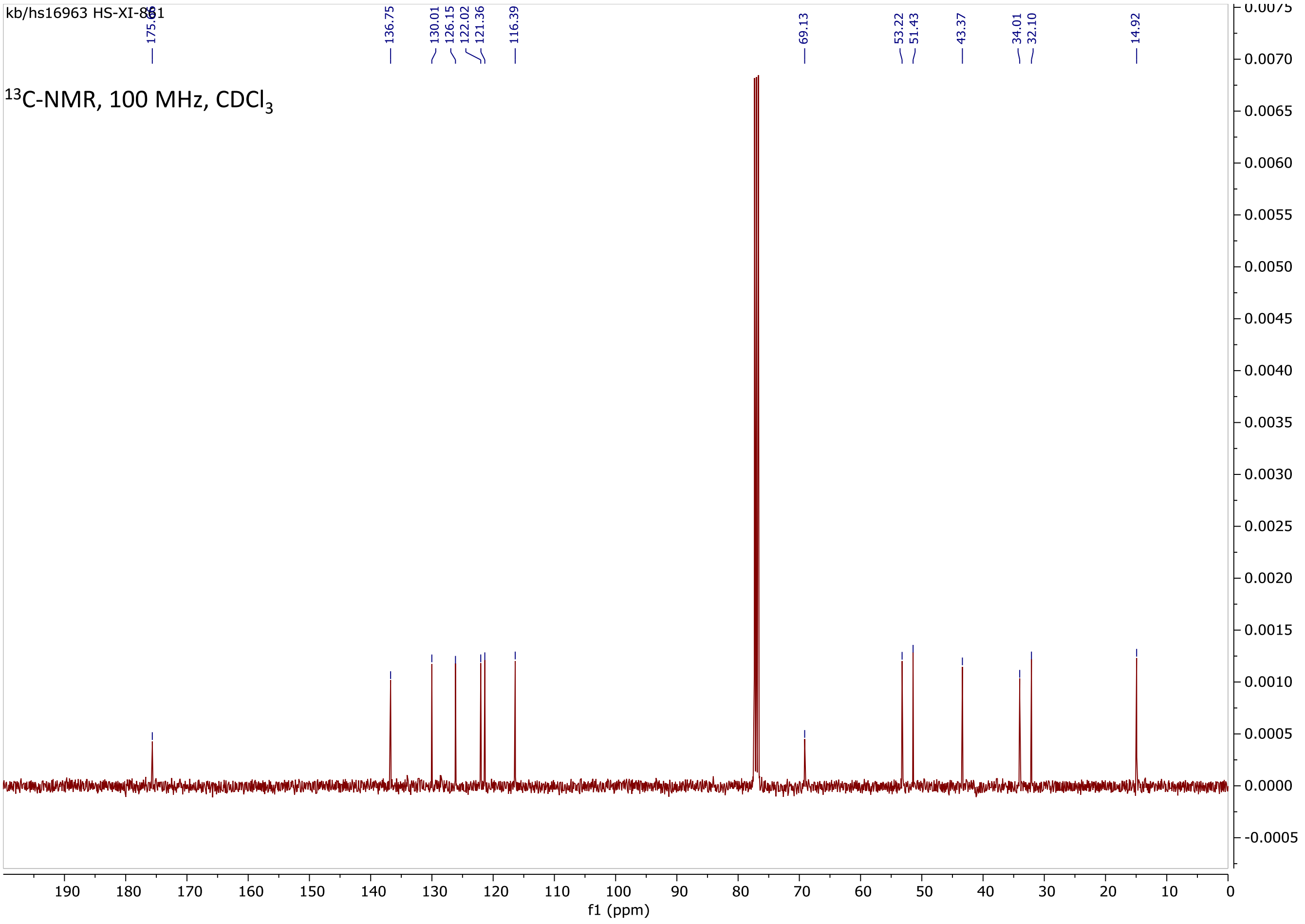

kb/hs16963 HS-XI-861
¹³C-NMR, 100 MHz, CDCl3
175.65
136.75
130.01
126.15
122.02
121.36
116.39
69.13
53.22
51.43
43.37
34.01
32.10
14.92
f1 (ppm)

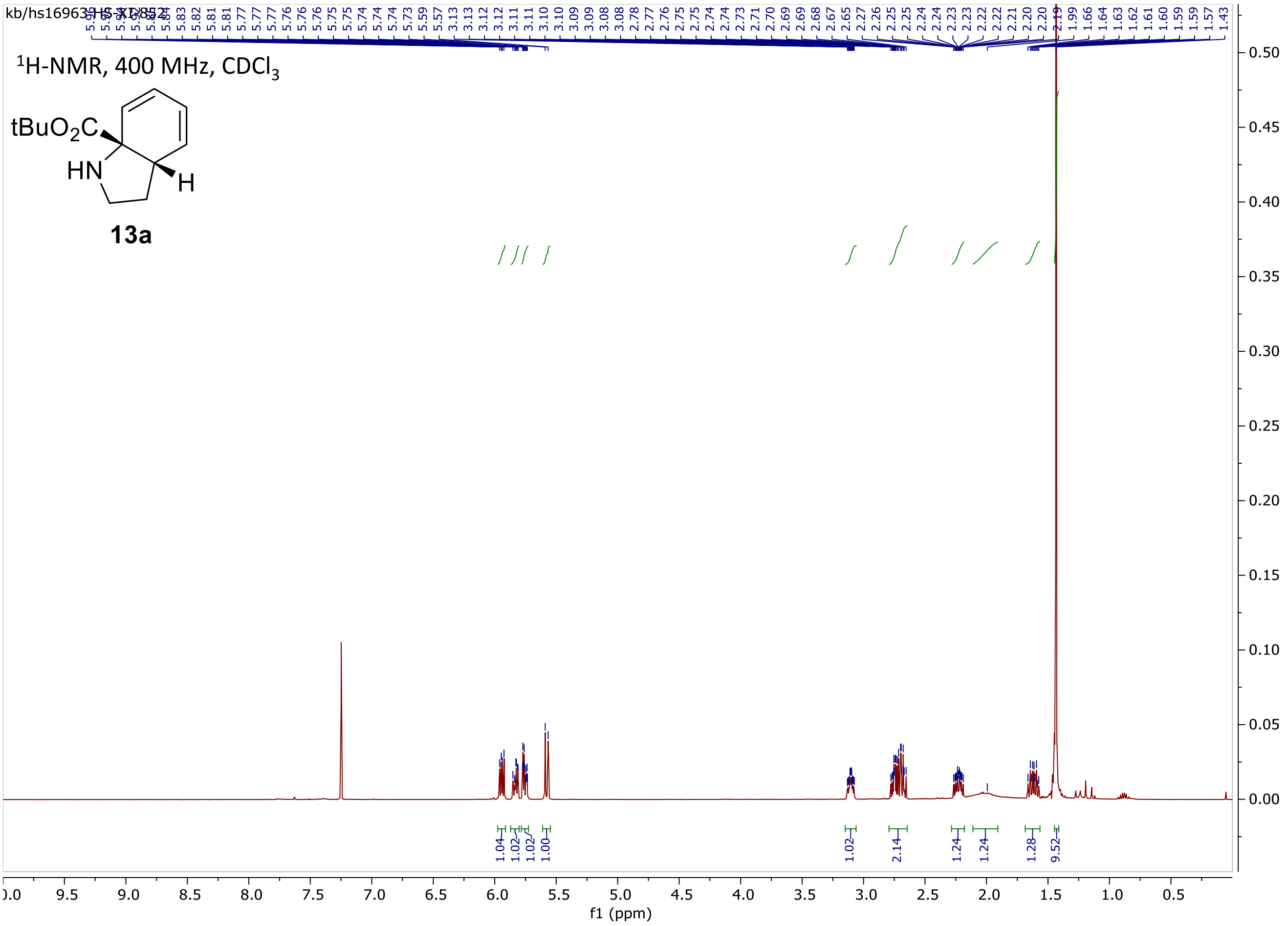
kb/hs16963HS-XI-852
^{1}H-NMR, 400 MHz, CDCl$_3$
tBuO$_2$C
HN
H
13a
1.04
1.02
1.02
1.00
1.02
2.14
1.24
1.24
1.28
9.52
f1 (ppm)

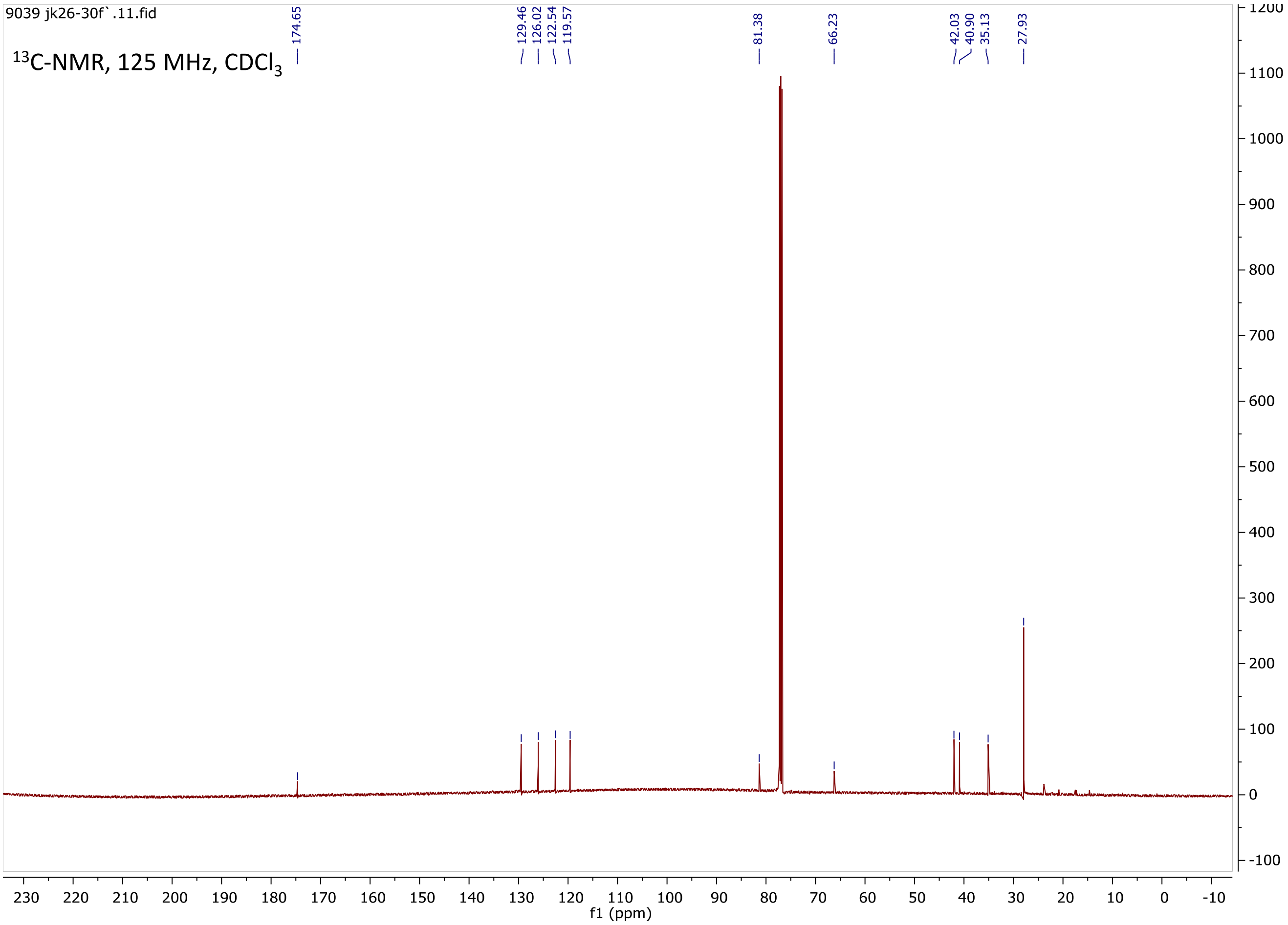
9039 jk26-30f`.11.fid
^{13}C-NMR, 125 MHz, CDCl$_3$
174.65
129.46
126.02
122.54
119.57
81.38
66.23
42.03
40.90
35.13
27.93
f1 (ppm)

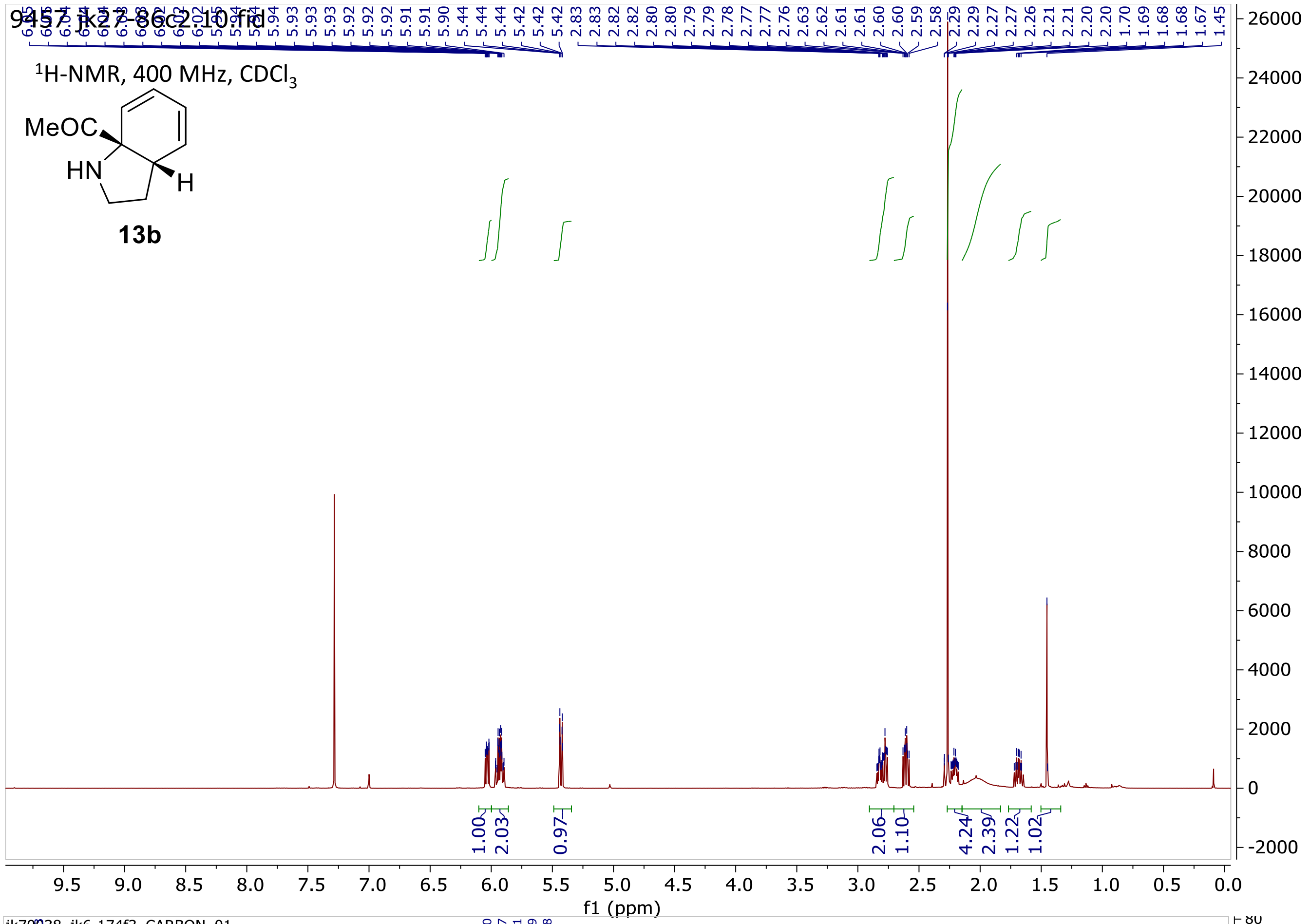
9457 jk27-86c2.10.fid
^{1}H-NMR, 400 MHz, $CDCl_3$
MeOC
HN
H
13b
f1 (ppm)

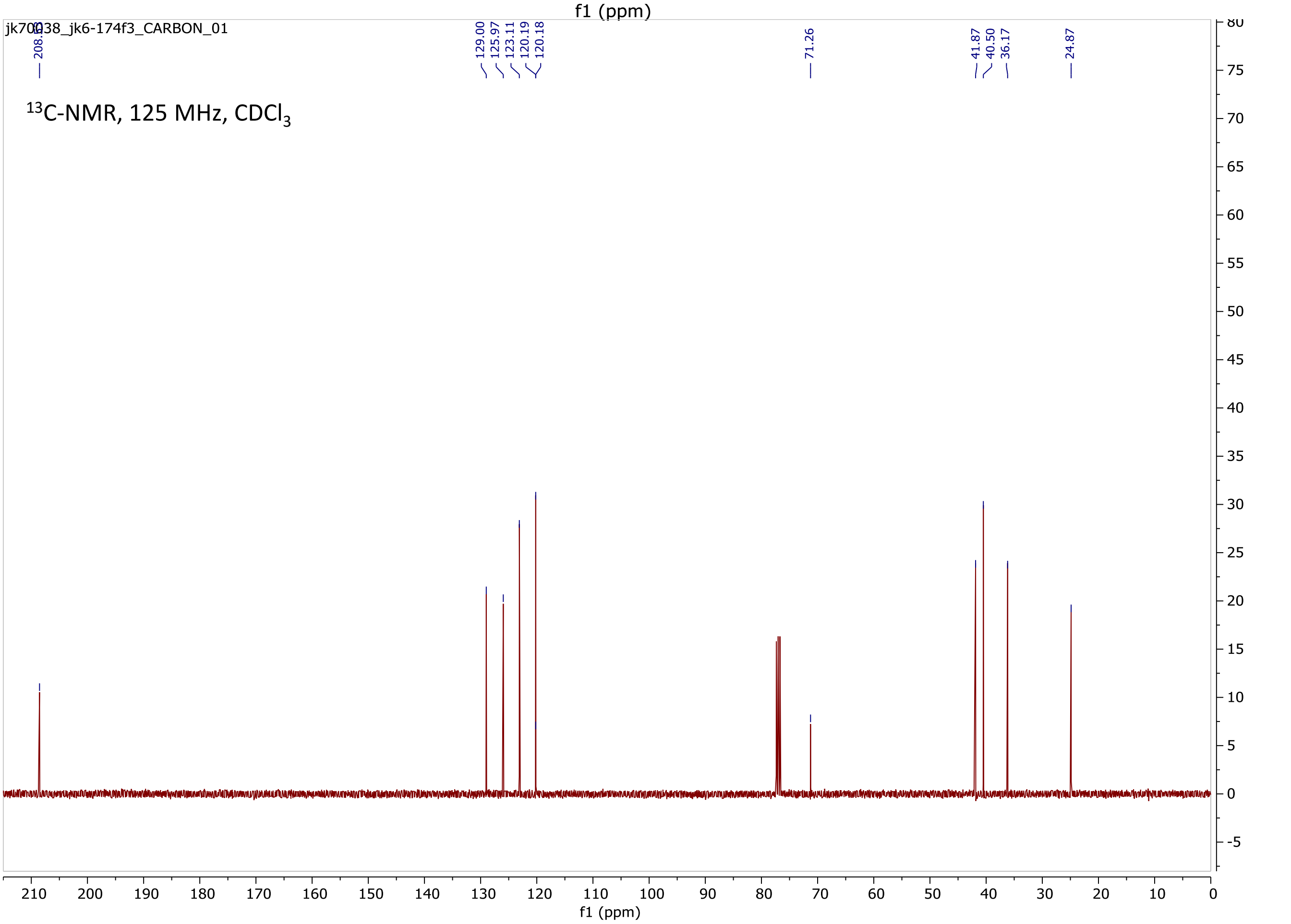
jk70038_jk6-174f3_CARBON_01
^{13}C-NMR, 125 MHz, $CDCl_3$
129.00
125.97
123.11
120.19
120.18
71.26
41.87
40.50
36.17
24.87
f1 (ppm)

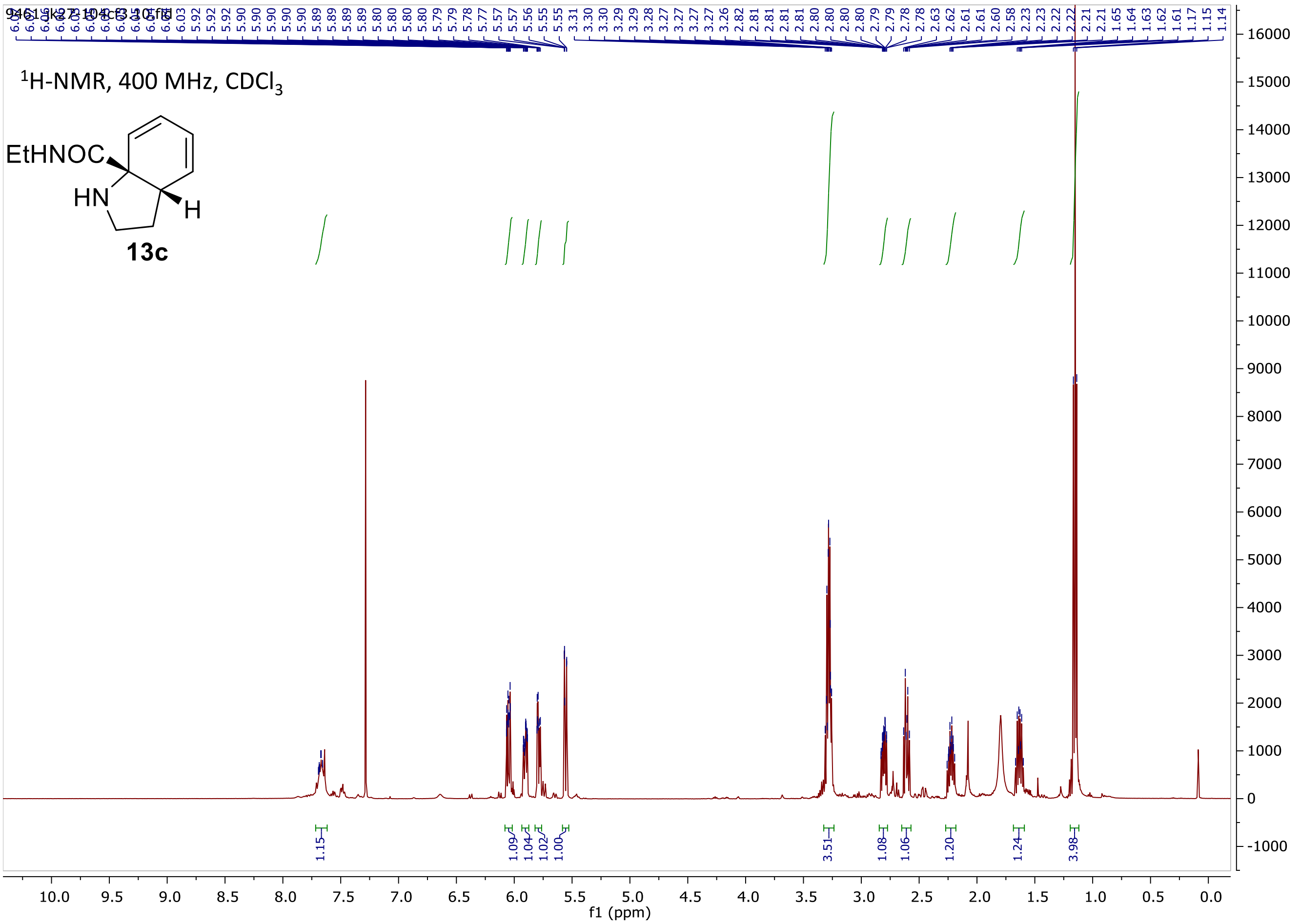
9461 jk27-104-cf3.10.[illegible]
1H-NMR, 400 MHz, CDCl3
EtHNOC
HN
H
13c
1.15
1.09
1.04
1.02
1.00
3.51
1.08
1.06
1.20
1.24
3.98
f1 (ppm)

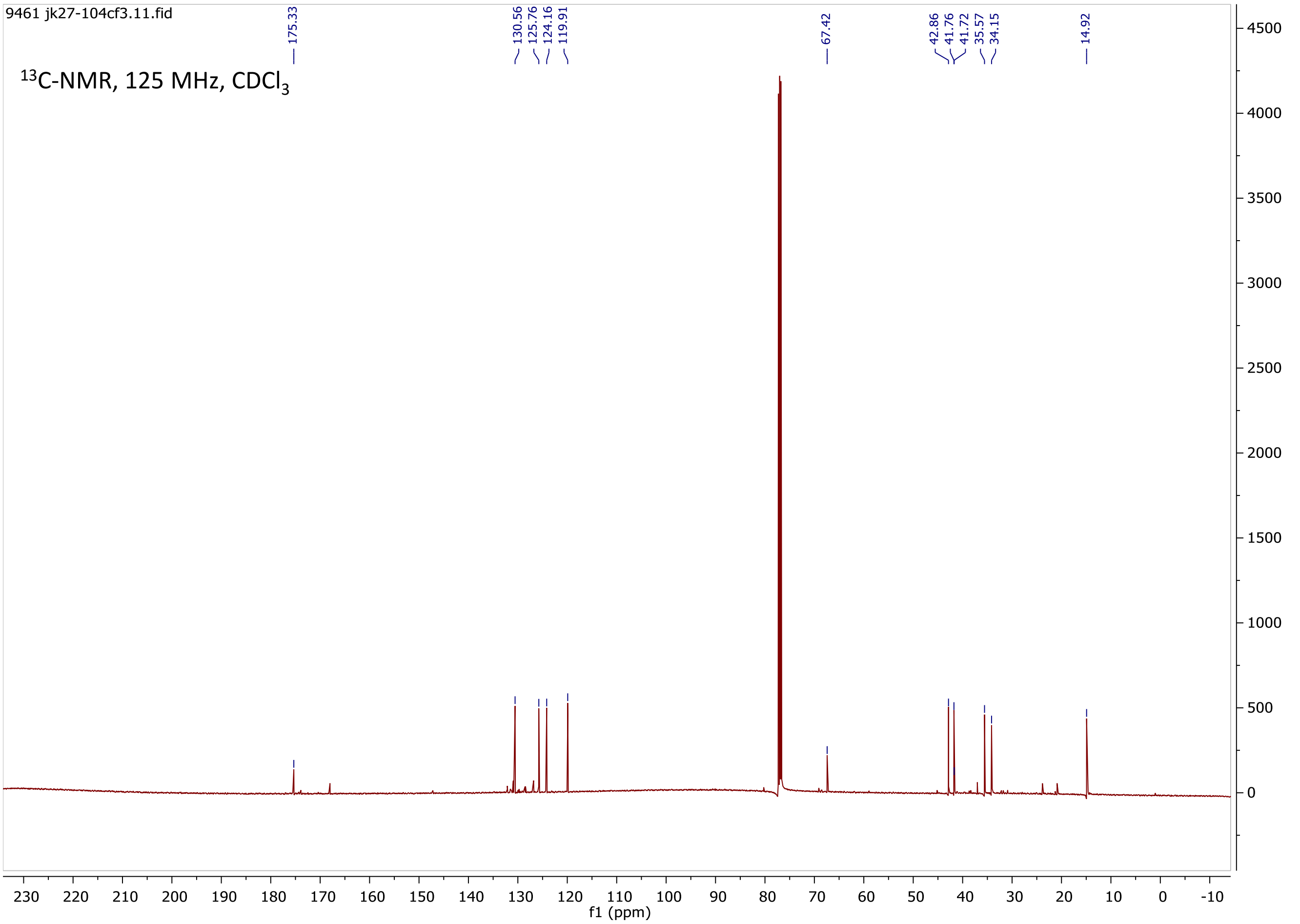
9461 jk27-104cf3.11.fid
175.33
130.56
125.76
124.16
119.91
67.42
42.86
41.76
41.72
35.57
34.15
14.92
13C-NMR, 125 MHz, CDCl3
f1 (ppm)

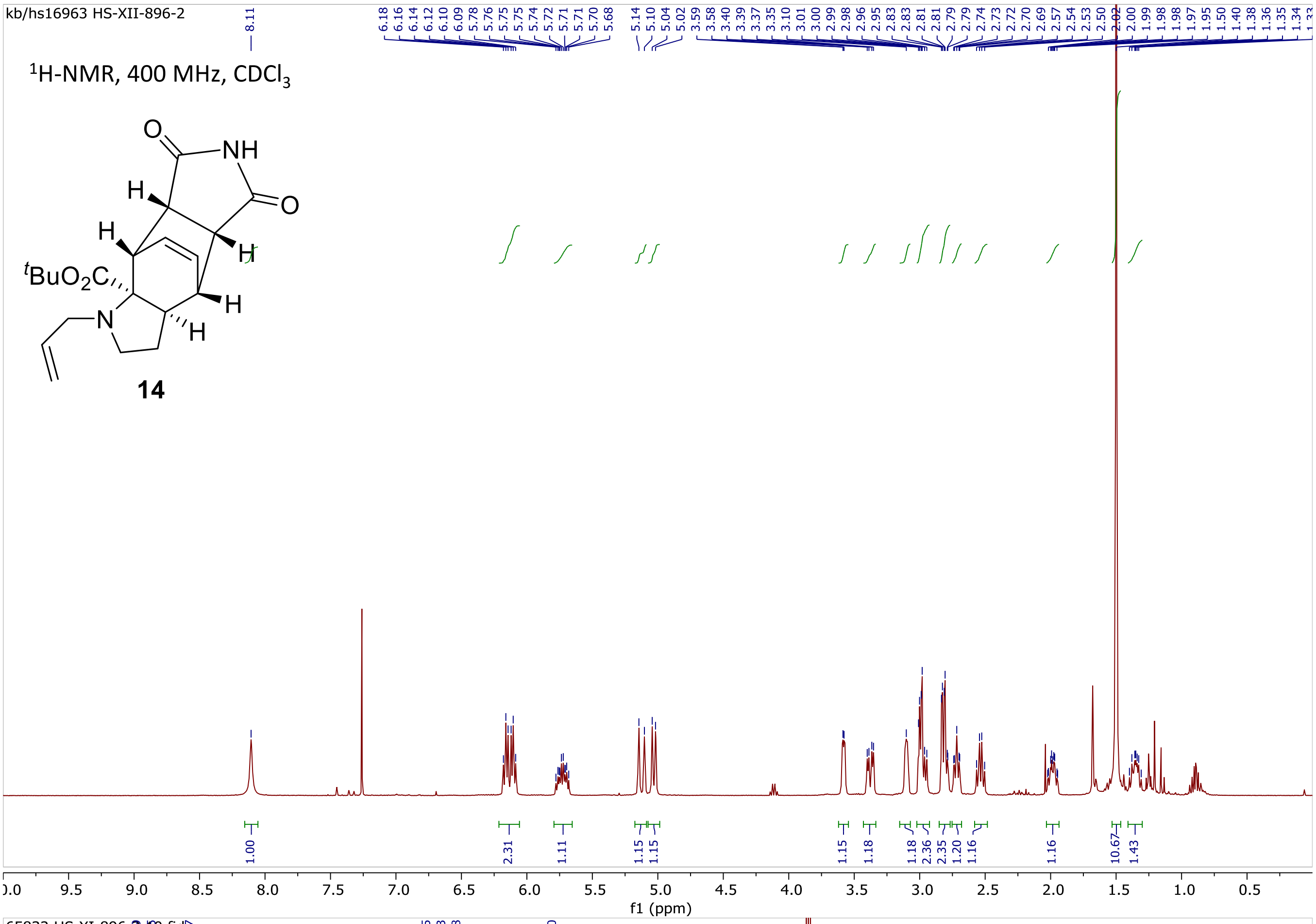
kb/hs16963 HS-XII-896-2
^{1}H-NMR, 400 MHz, $CDCl_3$
8.11
6.18 6.16 6.14 6.12 6.10 6.09 5.78 5.76 5.75 5.75 5.74 5.72 5.71 5.71 5.70 5.68
5.14 5.10 5.04 5.02
3.59 3.58 3.40 3.39 3.37 3.35 3.10 3.01 3.00 2.99 2.98 2.96 2.95 2.83 2.83 2.81 2.81 2.79 2.79 2.74 2.73 2.72 2.70 2.69 2.57 2.54 2.53 2.50 2.02 2.00 1.99 1.98 1.98 1.97 1.95 1.50 1.40 1.38 1.36 1.35 1.34 1.33
O NH O H H H H H
^{t}BuO$_2$C N
14
1.00 2.31 1.11 1.15 1.15 1.15 1.18 1.18 2.36 2.35 1.20 1.16 1.16 10.67 1.43
9.5 9.0 8.5 8.0 7.5 7.0 6.5 6.0 5.5 5.0 4.5 4.0 3.5 3.0 2.5 2.0 1.5 1.0 0.5
f1 (ppm)

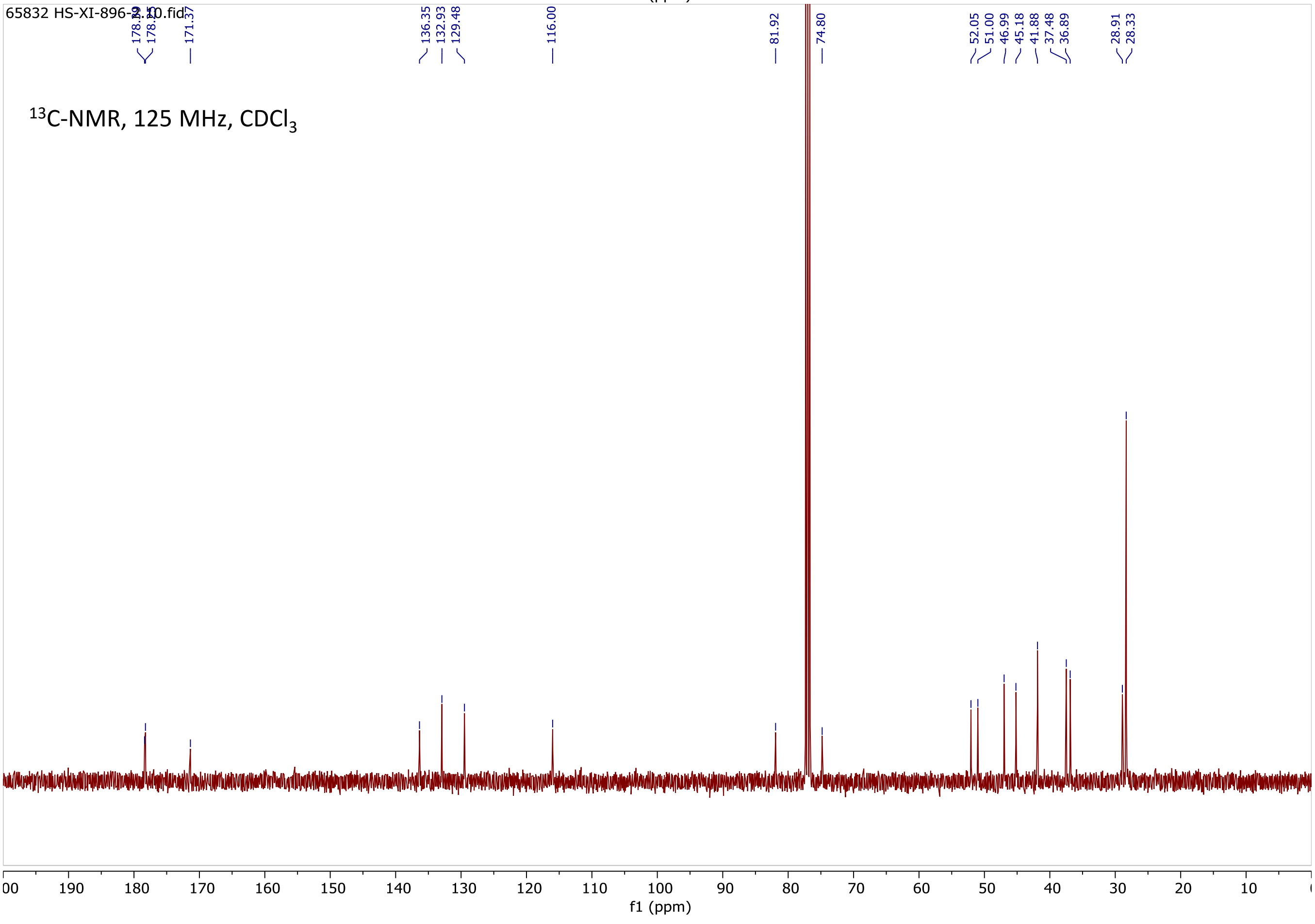
65832 HS-XI-896-2.10.fid
178.[illegible] 178.[illegible] 171.37
136.35 132.93 129.48
116.00
81.92
74.80
52.05 51.00 46.99 45.18 41.88 37.48 36.89
28.91 28.33
^{13}C-NMR, 125 MHz, $CDCl_3$
190 180 170 160 150 140 130 120 110 100 90 80 70 60 50 40 30 20 10
f1 (ppm)

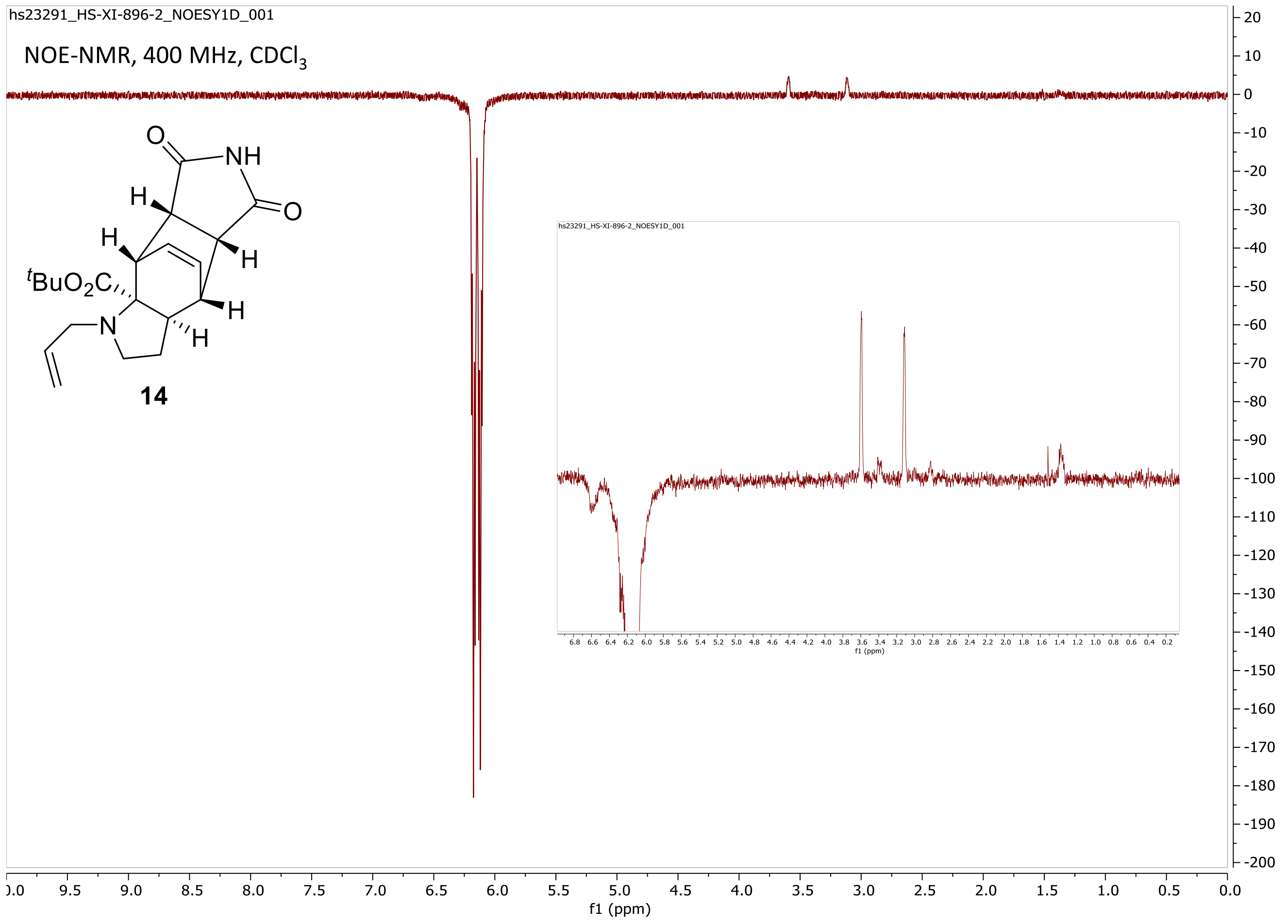
hs23291_HS-XI-896-2_NOESY1D_001
NOE-NMR, 400 MHz, CDCl3
O
NH
H
O
H
H
tBuO2C
H
N
H
14
f1 (ppm)

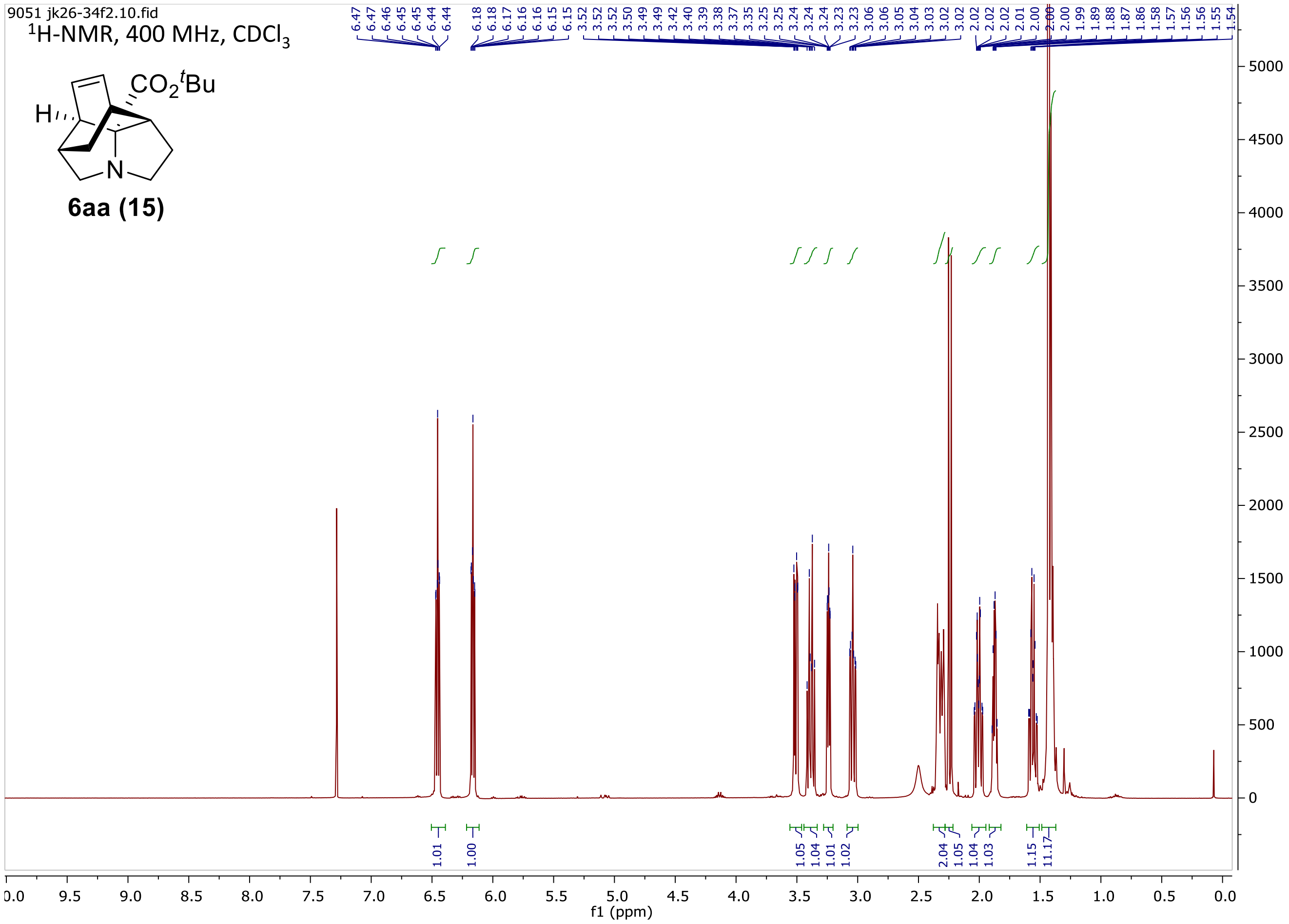
9051 jk26-34f2.10.fid
^{1}H-NMR, 400 MHz, $CDCl_3$
$CO_2{}^tBu$
H
N
6aa (15)
6.47 6.47 6.46 6.45 6.45 6.44 6.44 6.18 6.18 6.17 6.16 6.16 6.15 3.52 3.52 3.52 3.50 3.49 3.49 3.42 3.40 3.39 3.38 3.37 3.35 3.25 3.25 3.24 3.24 3.24 3.23 3.06 3.06 3.05 3.04 3.03 3.02 3.02 2.02 2.02 2.02 2.01 2.00 2.00 2.00 1.99 1.89 1.88 1.87 1.86 1.58 1.57 1.56 1.56 1.55 1.54
1.01 1.00 1.05 1.04 1.01 1.02 2.04 1.05 1.04 1.03 1.15 11.17
f1 (ppm)

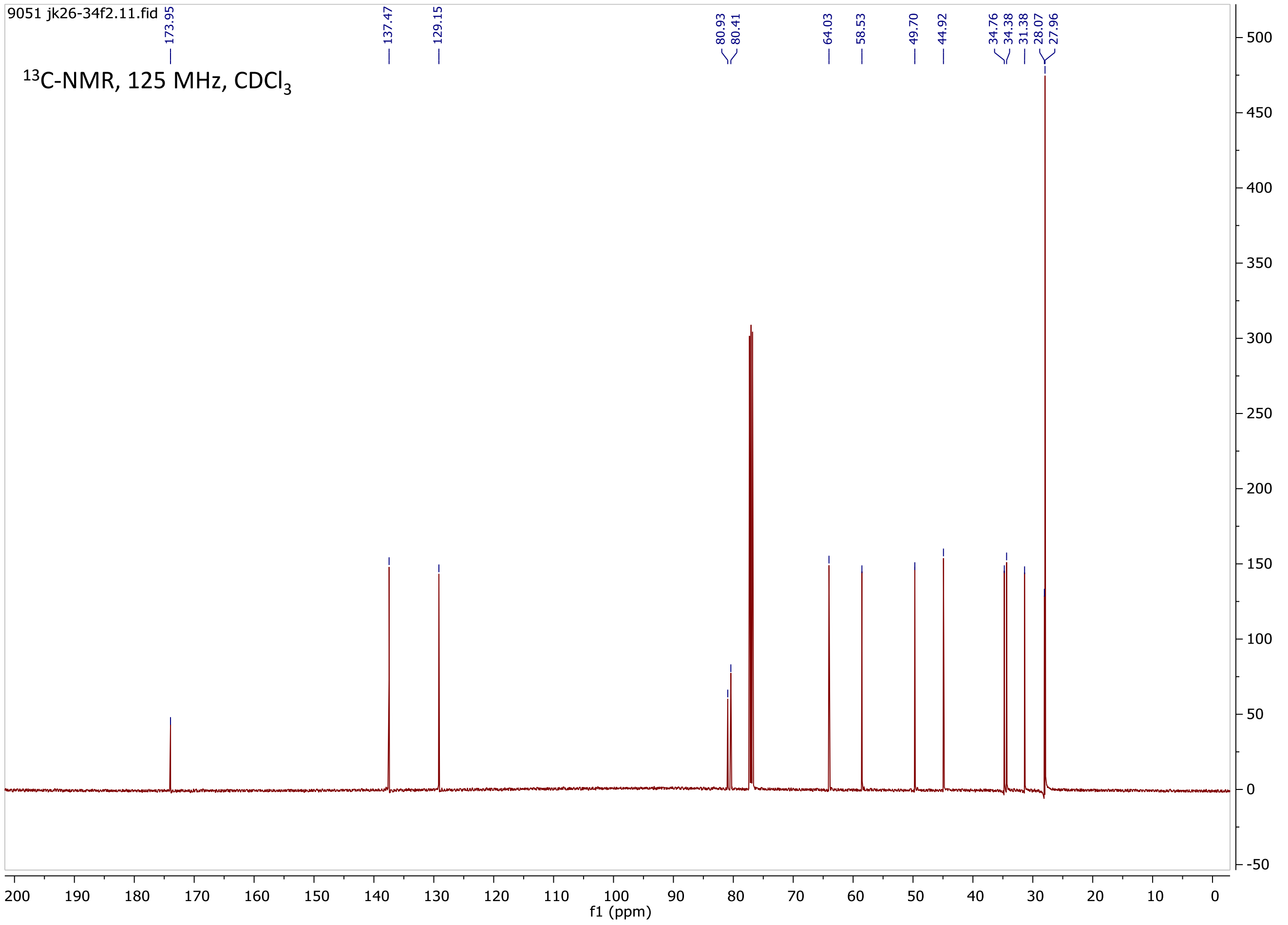
9051 jk26-34f2.11.fid
^{13}C-NMR, 125 MHz, $CDCl_3$
173.95 137.47 129.15 80.93 80.41 64.03 58.53 49.70 44.92 34.76 34.38 31.38 28.07 27.96
f1 (ppm)

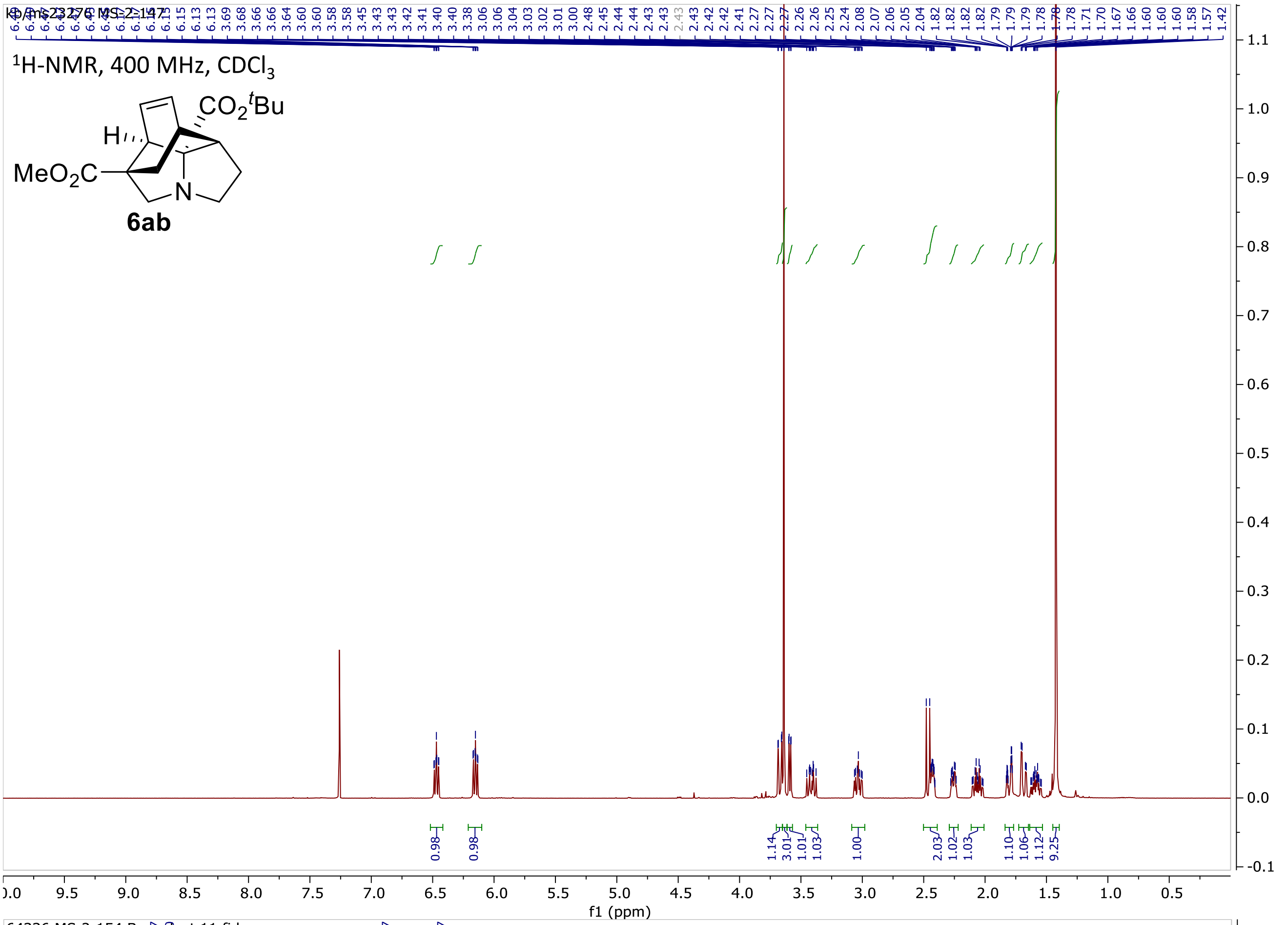

¹H-NMR, 400 MHz, CDCl₃
$CO_2{}^tBu$
H
MeO_2C
N
6ab
0.98
0.98
1.14
3.01
1.01
1.03
1.00
2.03
1.02
1.03
1.10
1.06
1.12
9.25
f1 (ppm)

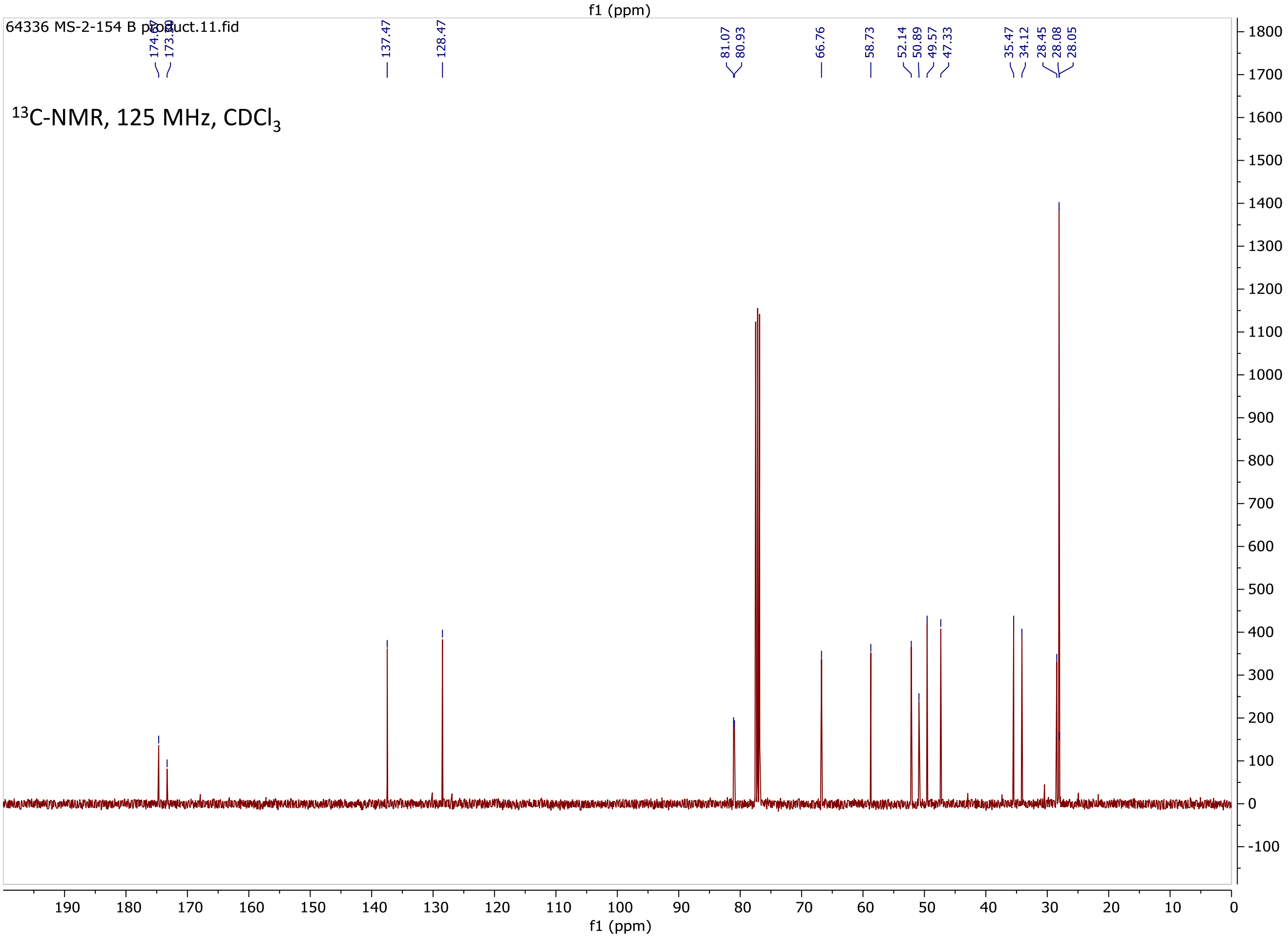

64336 MS-2-154 B product.11.fid
¹³C-NMR, 125 MHz, CDCl₃
174.0
173.
137.47
128.47
81.07
80.93
66.76
58.73
52.14
50.89
49.57
47.33
35.47
34.12
28.45
28.08
28.05
f1 (ppm)

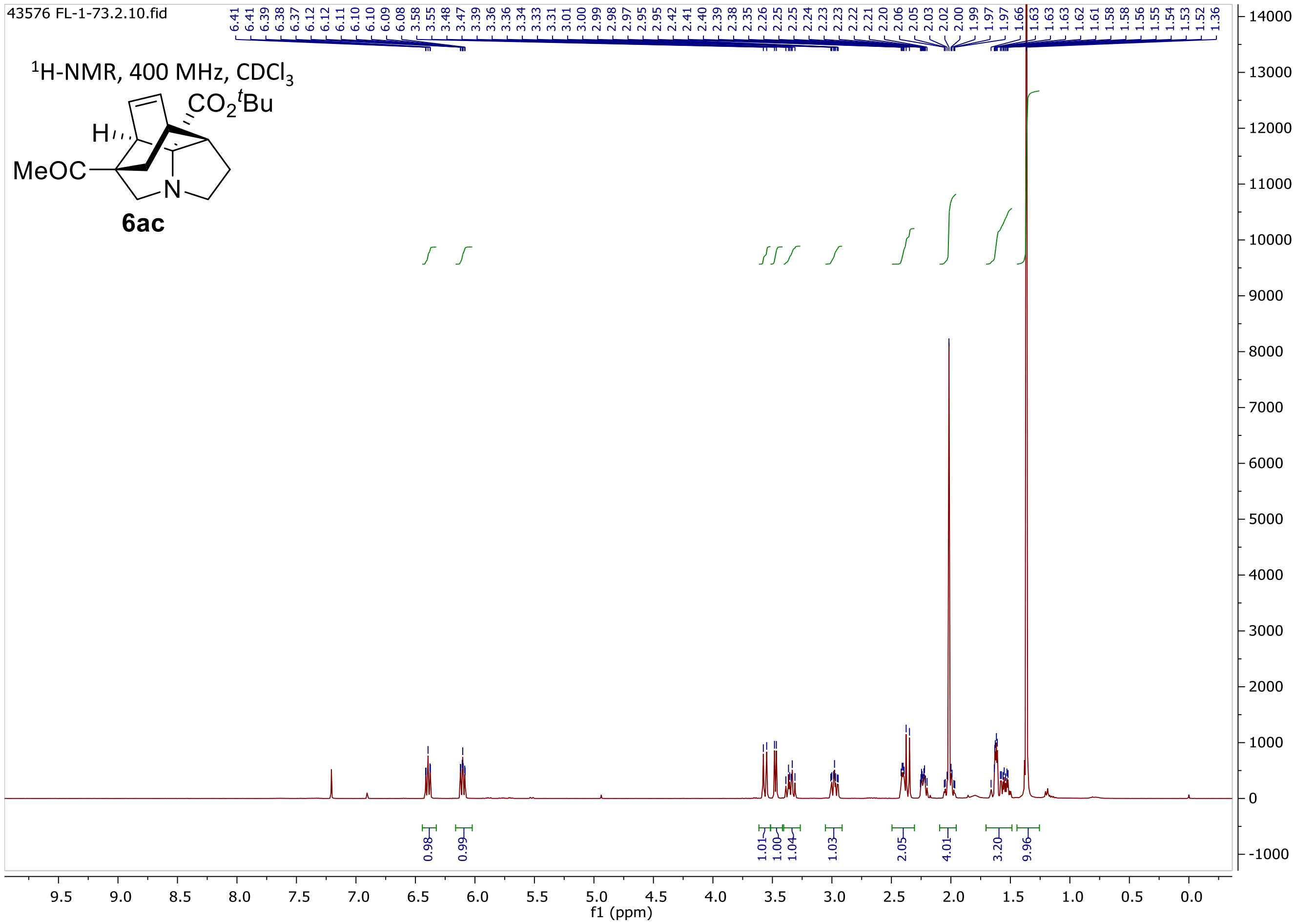
43576 FL-1-73.2.10.fid
1H-NMR, 400 MHz, CDCl3
6ac
6.41 6.41 6.39 6.38 6.37 6.12 6.12 6.11 6.10 6.09 6.08 3.58 3.55 3.48 3.47 3.39 3.36 3.36 3.34 3.33 3.31 3.01 3.00 2.99 2.98 2.97 2.95 2.95 2.42 2.41 2.40 2.39 2.38 2.35 2.26 2.25 2.25 2.24 2.23 2.23 2.22 2.21 2.20 2.06 2.05 2.03 2.02 2.00 1.99 1.97 1.97 1.66 1.63 1.63 1.62 1.61 1.58 1.58 1.56 1.55 1.54 1.53 1.52 1.36
0.98 0.99 1.01 1.00 1.04 1.03 2.05 4.01 3.20 9.96
f1 (ppm)

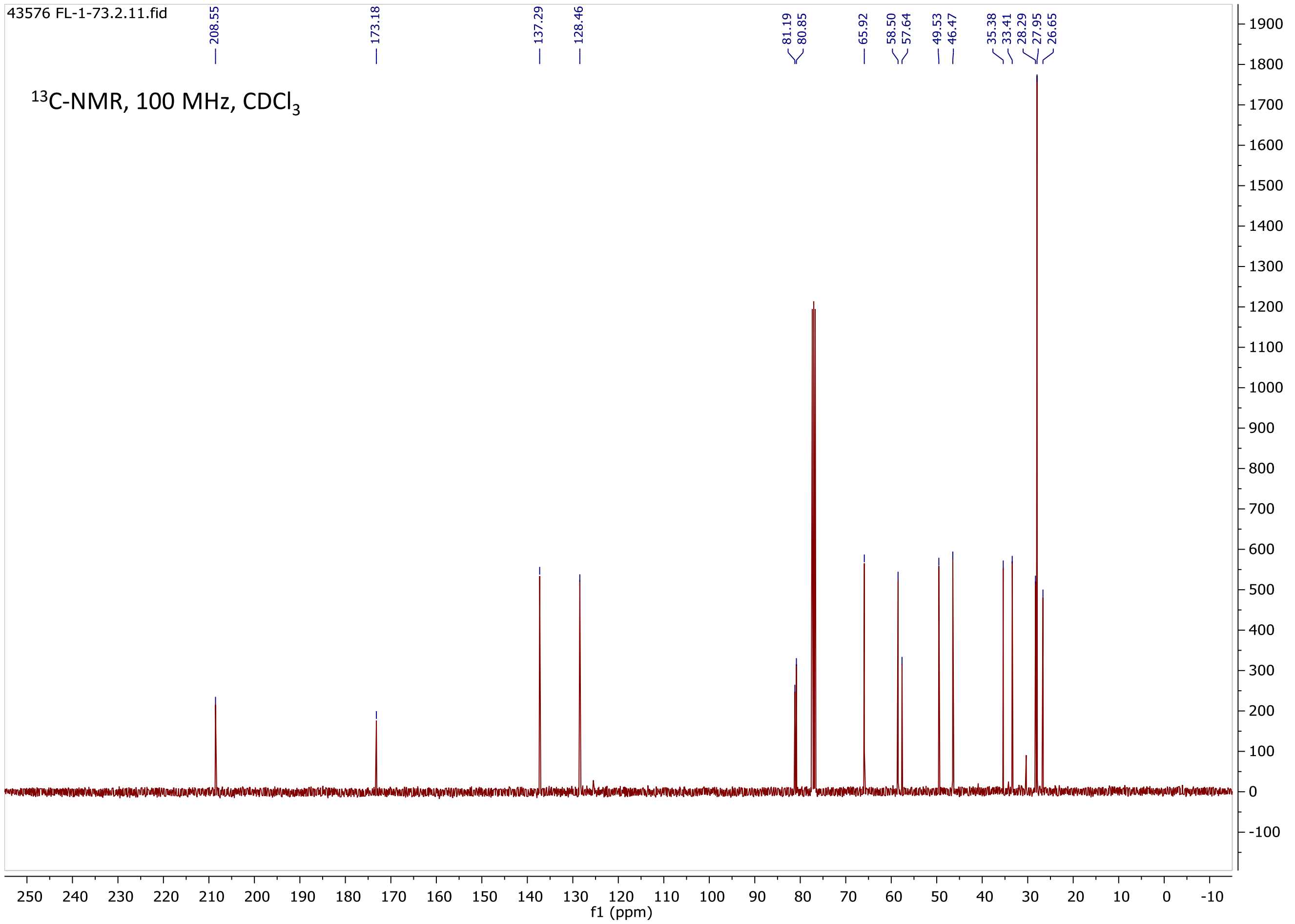
43576 FL-1-73.2.11.fid
13C-NMR, 100 MHz, CDCl3
208.55 173.18 137.29 128.46 81.19 80.85 65.92 58.50 57.64 49.53 46.47 35.38 33.41 28.29 27.95 26.65
f1 (ppm)

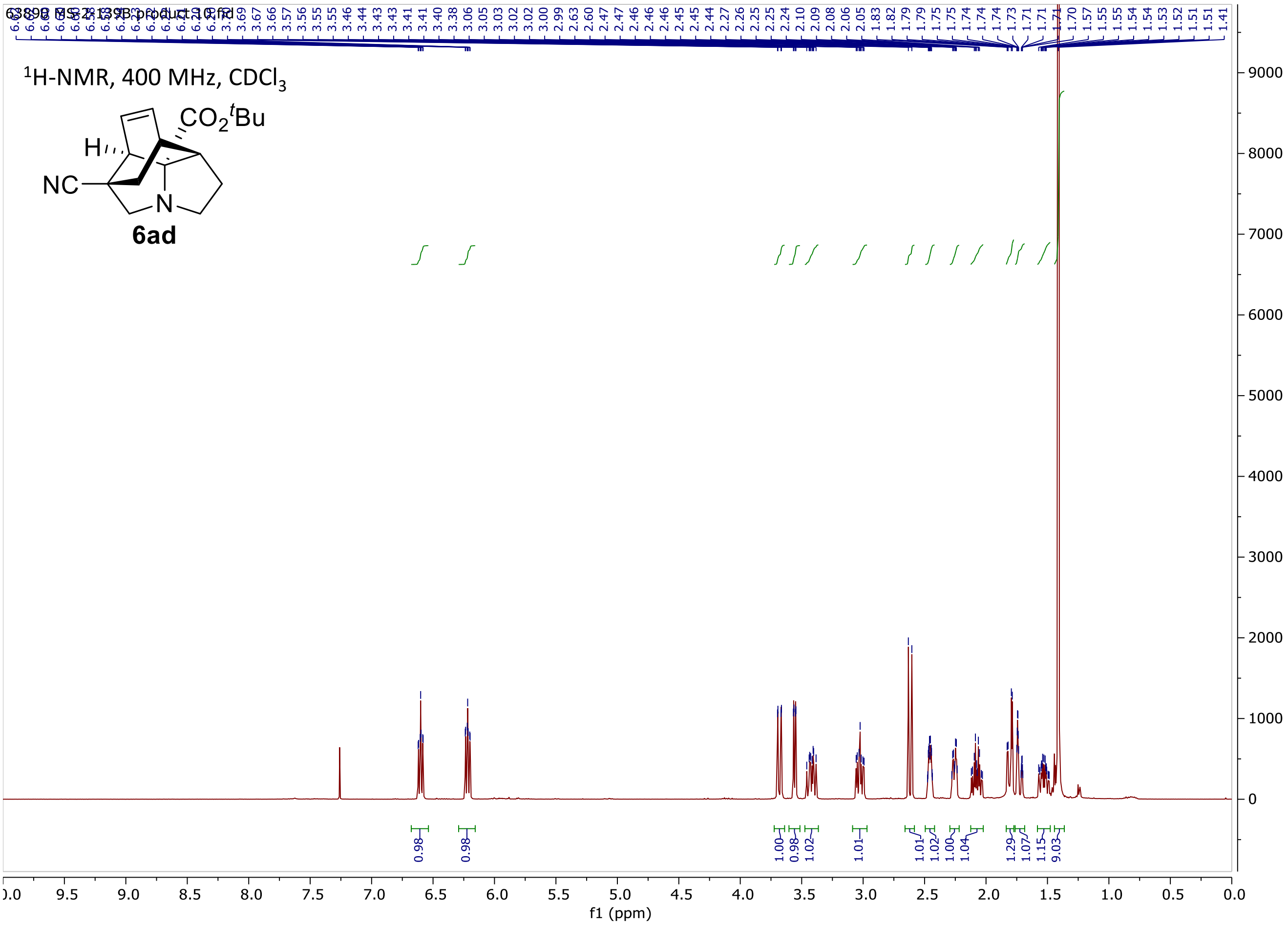

^{1}H-NMR, 400 MHz, $CDCl_3$
6ad
f1 (ppm)

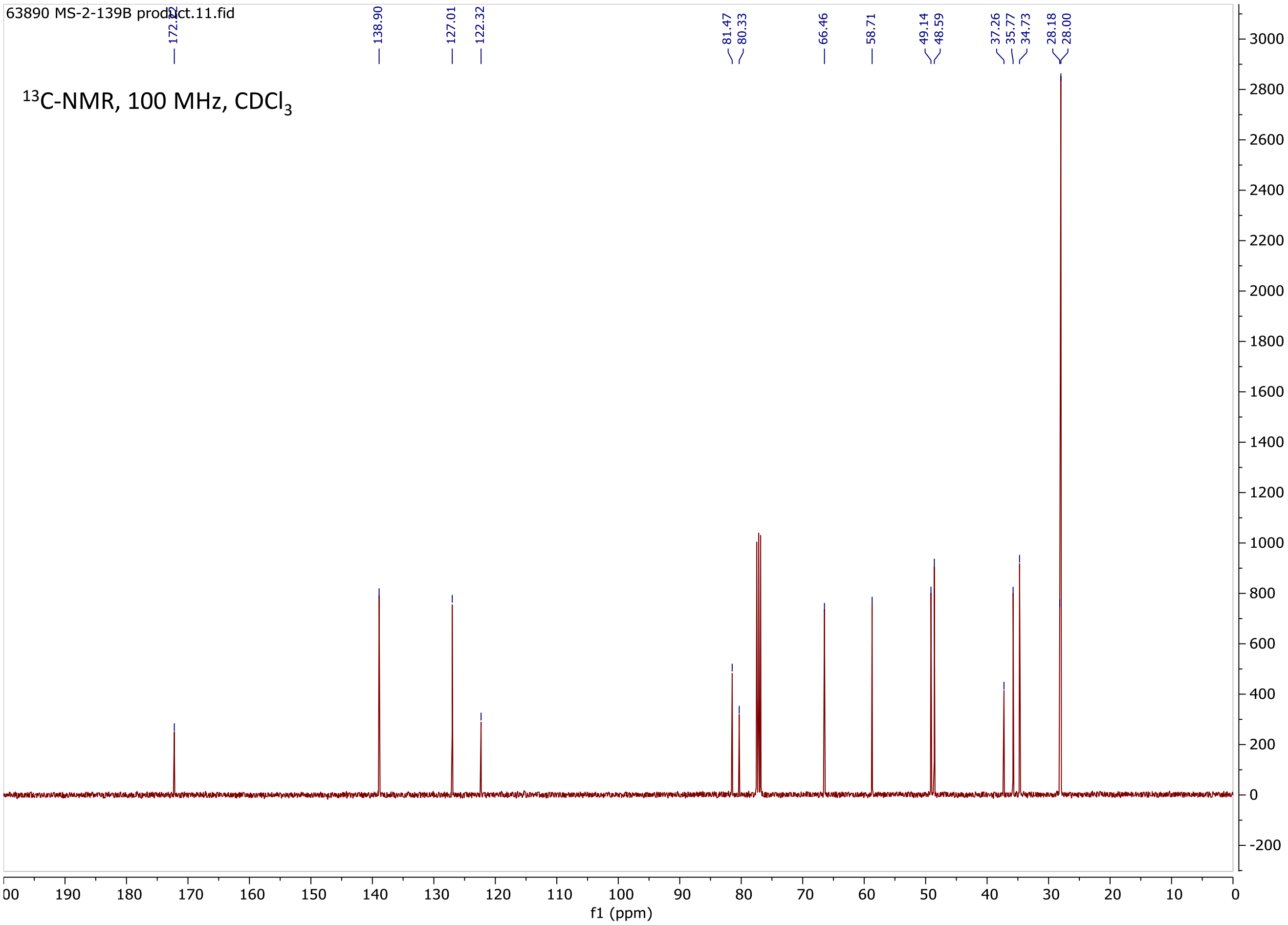

63890 MS-2-139B product.11.fid
^{13}C-NMR, 100 MHz, $CDCl_3$
172.12
138.90
127.01
122.32
81.47
80.33
66.46
58.71
49.14
48.59
37.26
35.77
34.73
28.18
28.00
f1 (ppm)

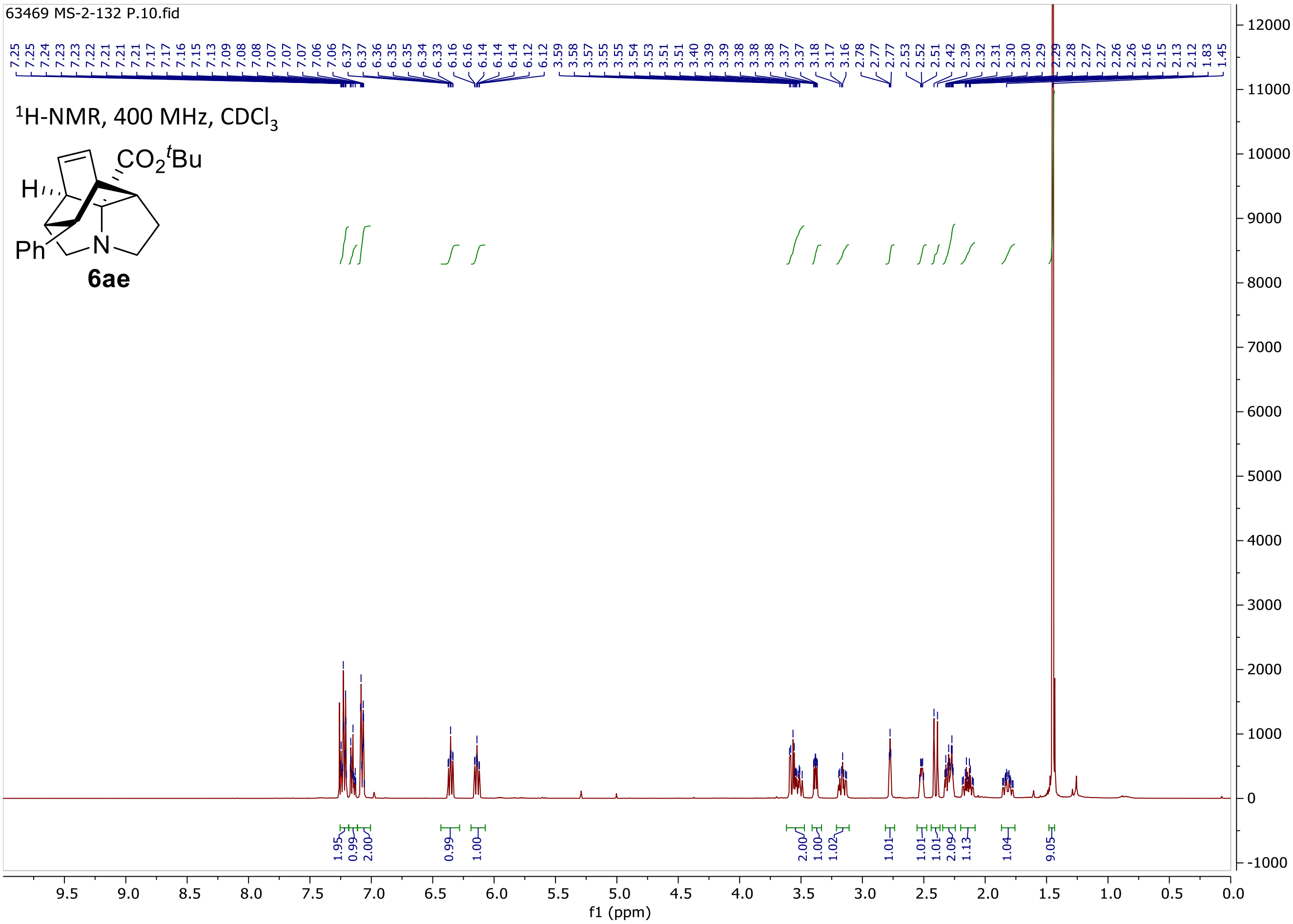
63469 MS-2-132 P.10.fid
7.25 7.25 7.24 7.23 7.23 7.22 7.21 7.21 7.21 7.17 7.17 7.16 7.15 7.13 7.09 7.08 7.08 7.07 7.07 7.06 7.06 6.37 6.37 6.36 6.35 6.35 6.34 6.33 6.16 6.16 6.14 6.14 6.12 6.12 3.59 3.58 3.57 3.55 3.55 3.54 3.53 3.51 3.51 3.40 3.39 3.39 3.38 3.38 3.38 3.37 3.37 3.18 3.17 3.16 2.78 2.77 2.77 2.53 2.52 2.51 2.42 2.39 2.32 2.31 2.30 2.29 2.29 2.28 2.27 2.27 2.26 2.26 2.16 2.15 2.13 2.12 1.83 1.45
^{1}H-NMR, 400 MHz, $CDCl_3$
$CO_2{}^tBu$
H
Ph
N
6ae
1.95 0.99 2.00 0.99 1.00 2.00 1.00 1.02 1.01 1.01 1.01 2.09 1.13 1.04 9.05
f1 (ppm)

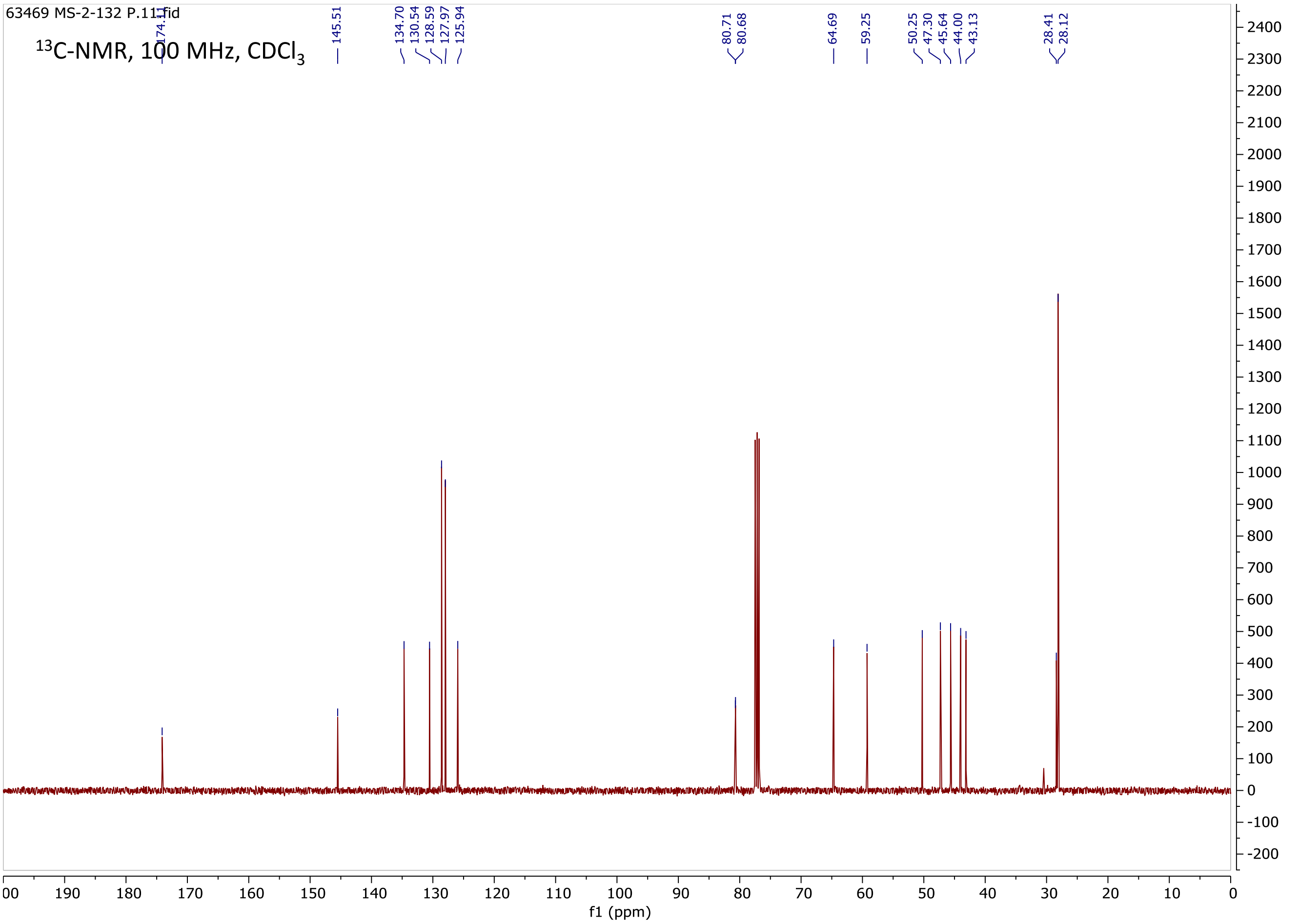
63469 MS-2-132 P.11.fid
^{13}C-NMR, 100 MHz, $CDCl_3$
145.51 134.70 130.54 128.59 127.97 125.94 80.71 80.68 64.69 59.25 50.25 47.30 45.64 44.00 43.13 28.41 28.12
f1 (ppm)

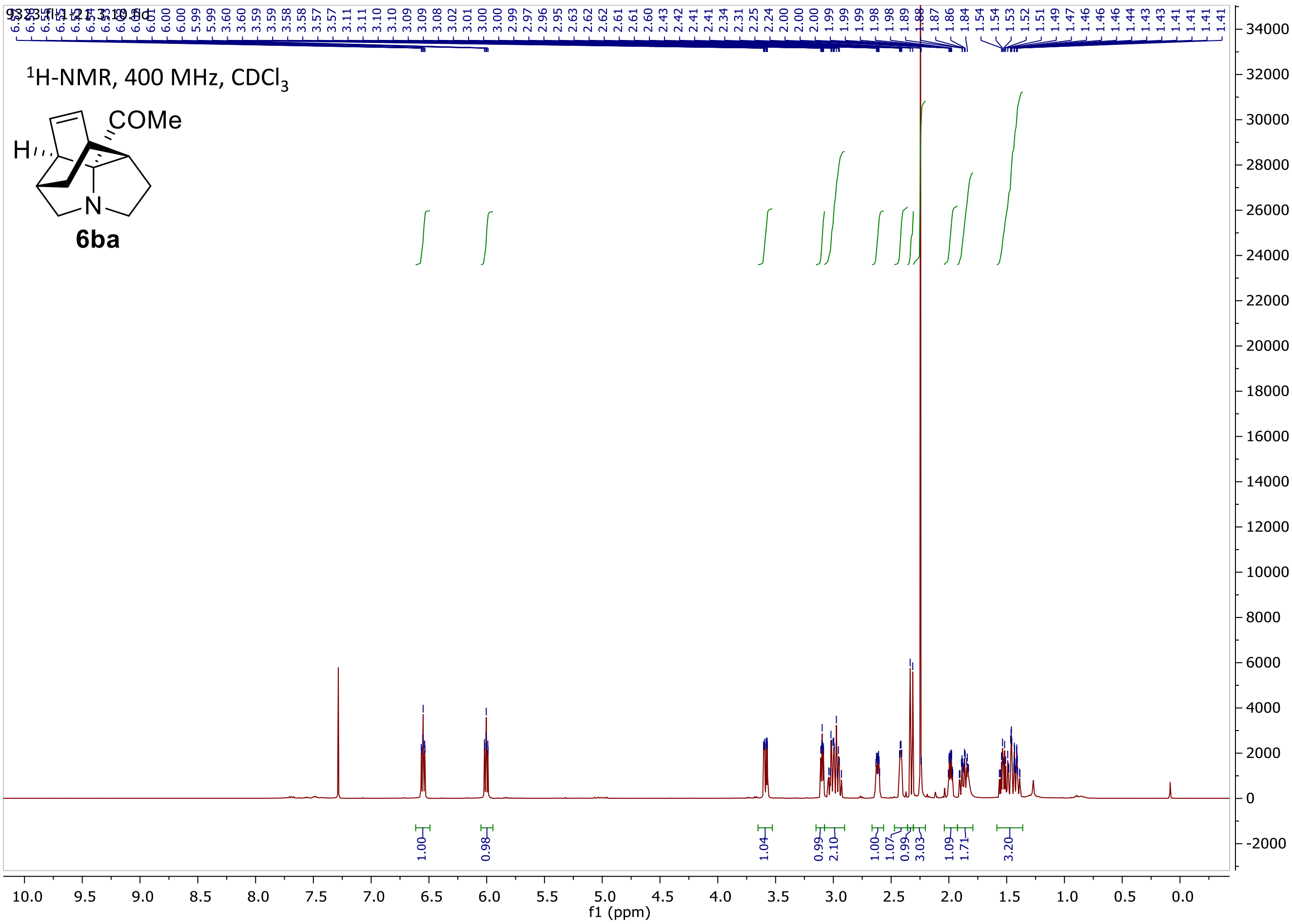
1H-NMR, 400 MHz, CDCl3
COMe
N
6ba
f1 (ppm)

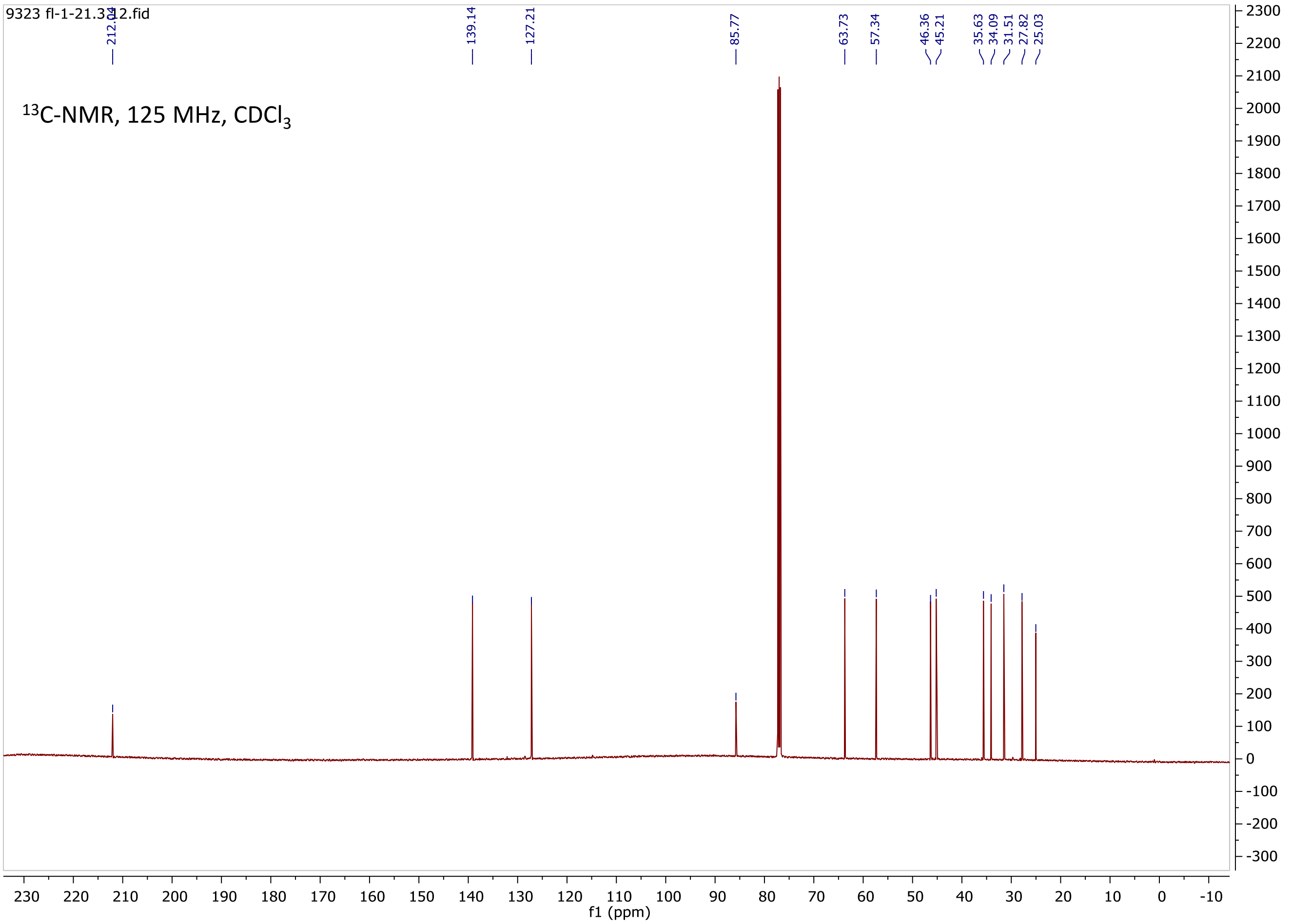
13C-NMR, 125 MHz, CDCl3
212.04
139.14
127.21
85.77
63.73
57.34
46.36
45.21
35.63
34.09
31.51
27.82
25.03
f1 (ppm)

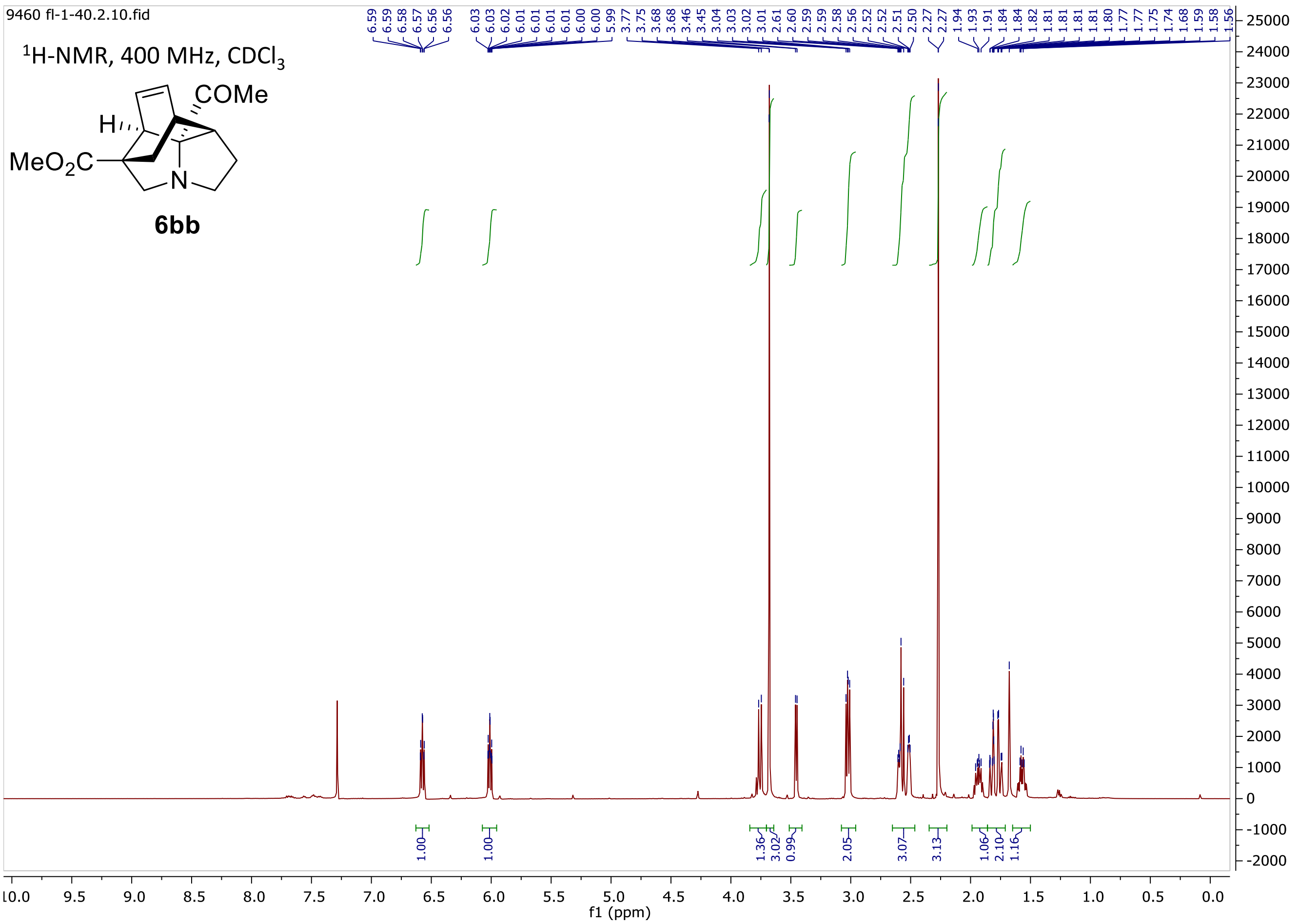

9460 fl-1-40.2.10.fid
^{1}H-NMR, 400 MHz, $CDCl_3$
COMe
H
MeO_2C
N
6bb
6.59 6.59 6.58 6.57 6.56 6.56 6.03 6.03 6.02 6.01 6.01 6.01 6.00 6.00 5.99 3.77 3.75 3.68 3.46 3.45 3.04 3.03 3.02 3.01 2.61 2.60 2.59 2.59 2.58 2.56 2.52 2.52 2.51 2.50 2.27 2.27 1.94 1.93 1.91 1.84 1.84 1.82 1.81 1.81 1.81 1.80 1.77 1.75 1.74 1.68 1.59 1.58 1.56
1.00 1.00 1.36 3.02 0.99 2.05 3.07 3.13 1.06 2.10 1.16
f1 (ppm)

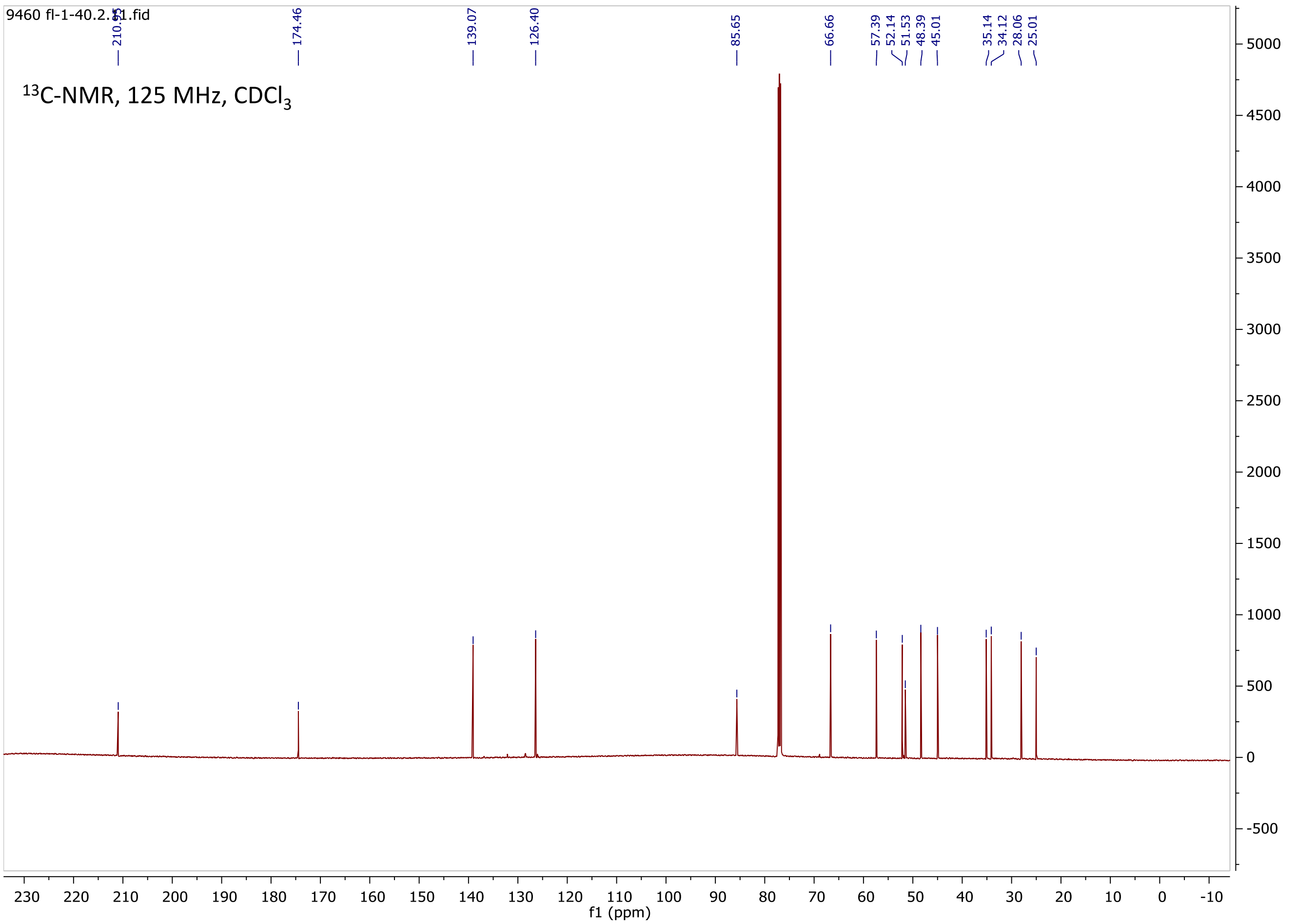

^{13}C-NMR, 125 MHz, $CDCl_3$
174.46 139.07 126.40 85.65 66.66 57.39 52.14 51.53 48.39 45.01 35.14 34.12 28.06 25.01
f1 (ppm)

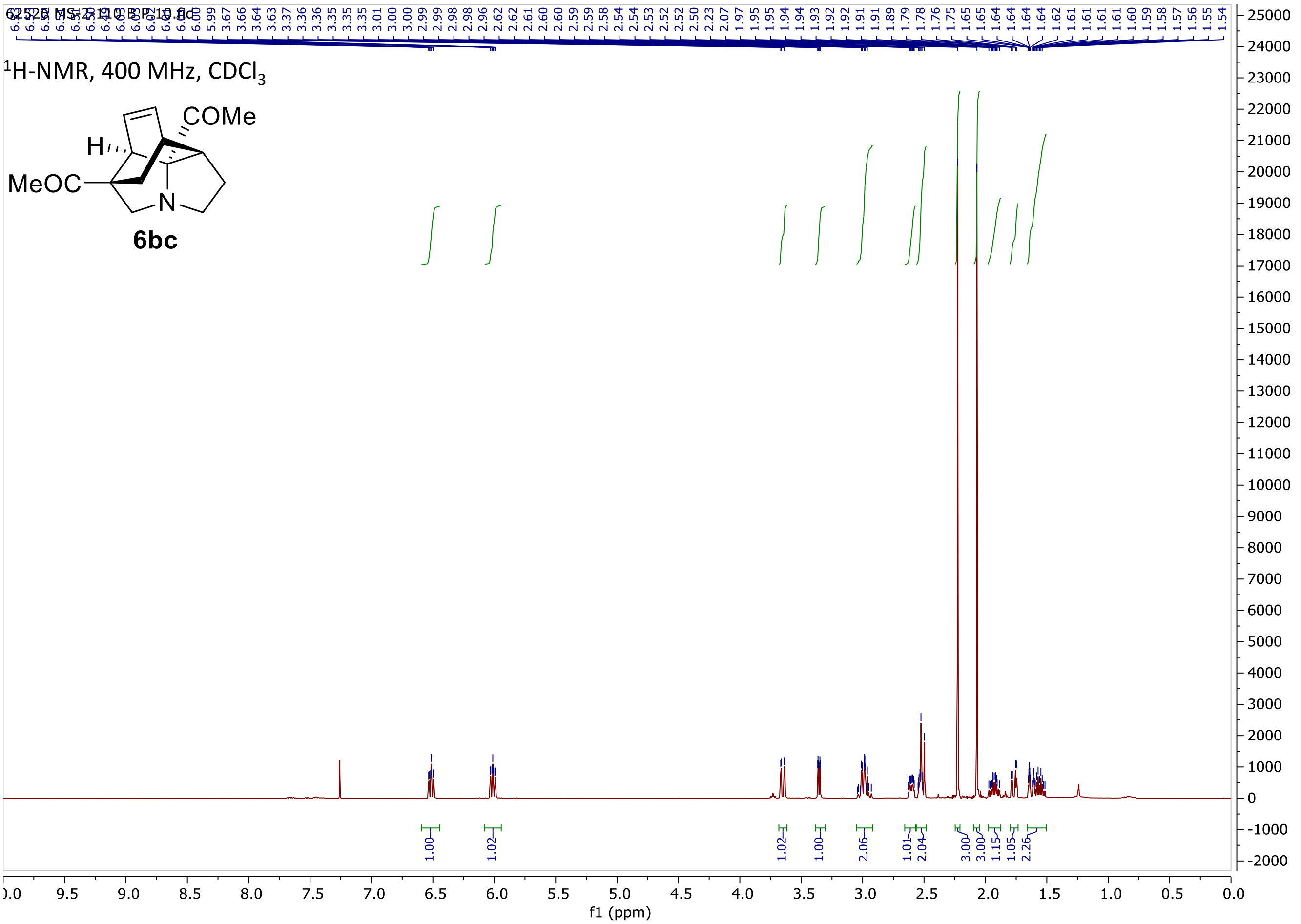

¹H-NMR, 400 MHz, CDCl3
COMe
H
MeOC
N
6bc
1.00
1.02
1.02
1.00
2.06
1.01
2.04
3.00
3.00
1.15
1.05
2.26
f1 (ppm)

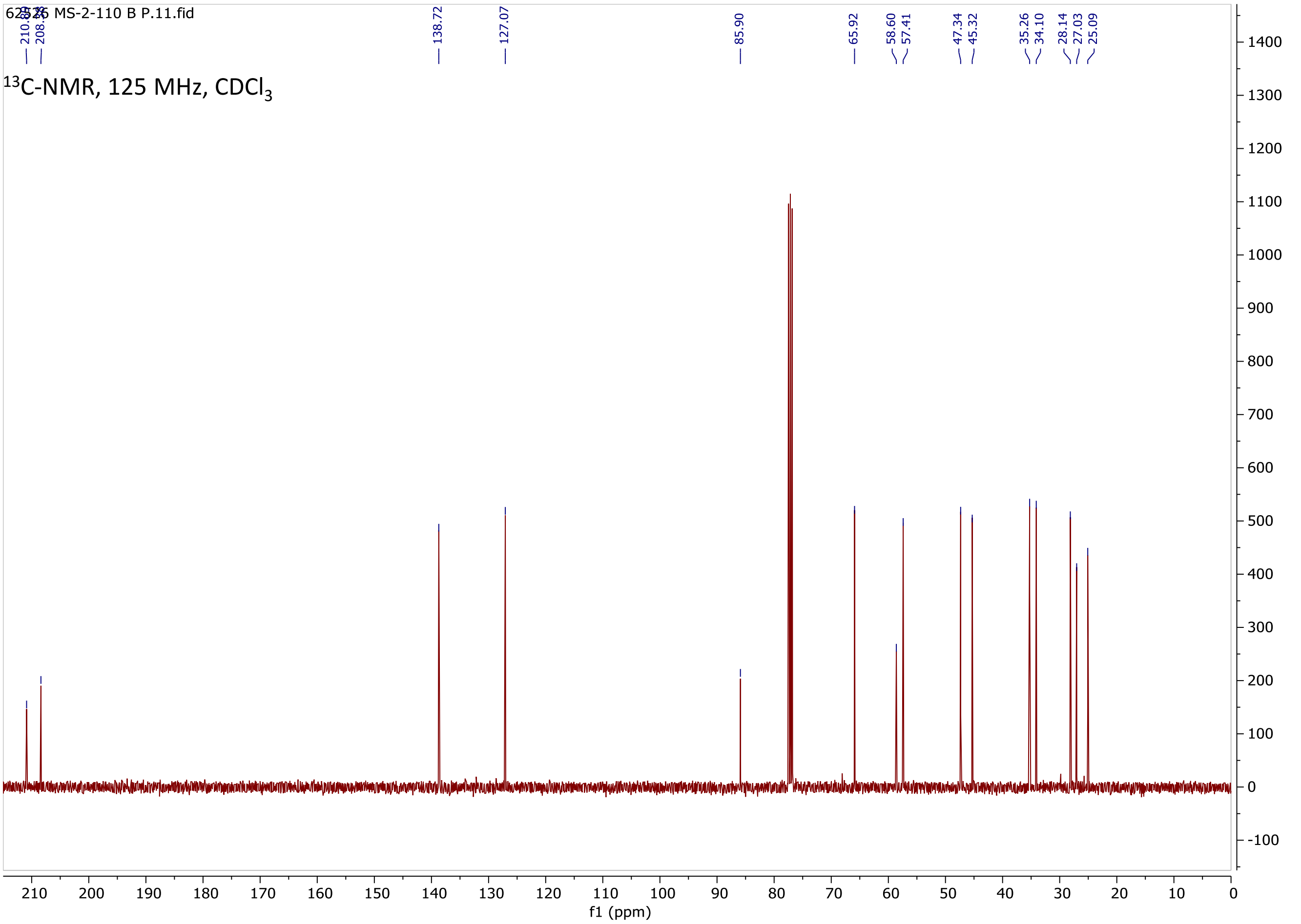

62526 MS-2-110 B P.11.fid
138.72
127.07
85.90
65.92
58.60
57.41
47.34
45.32
35.26
34.10
28.14
27.03
25.09
¹³C-NMR, 125 MHz, CDCl3
f1 (ppm)

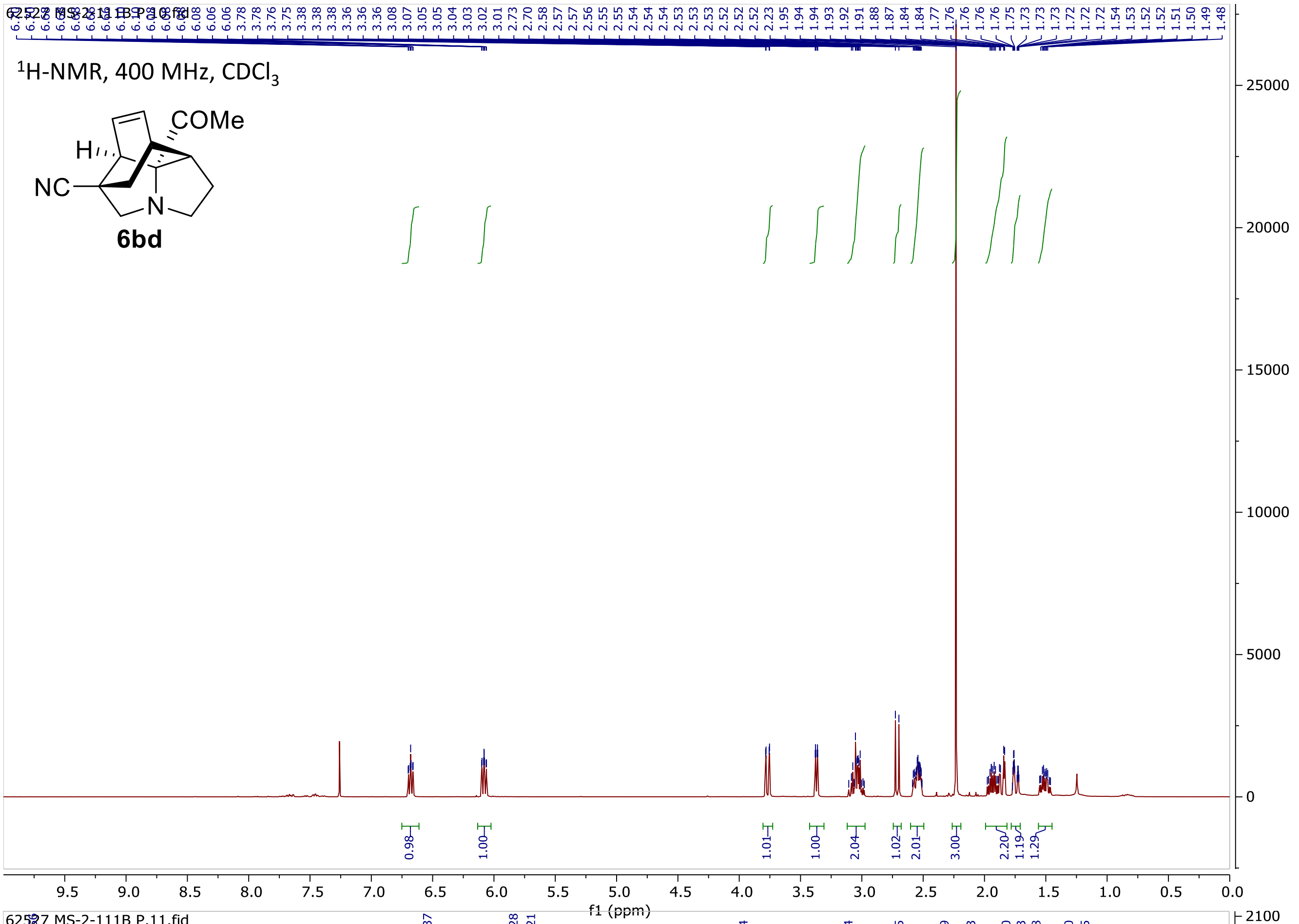

^{1}H-NMR, 400 MHz, $CDCl_3$
COMe
H
NC
N
6bd
0.98
1.00
1.01
1.00
2.04
1.02
2.01
3.00
2.20
1.19
1.29
f1 (ppm)

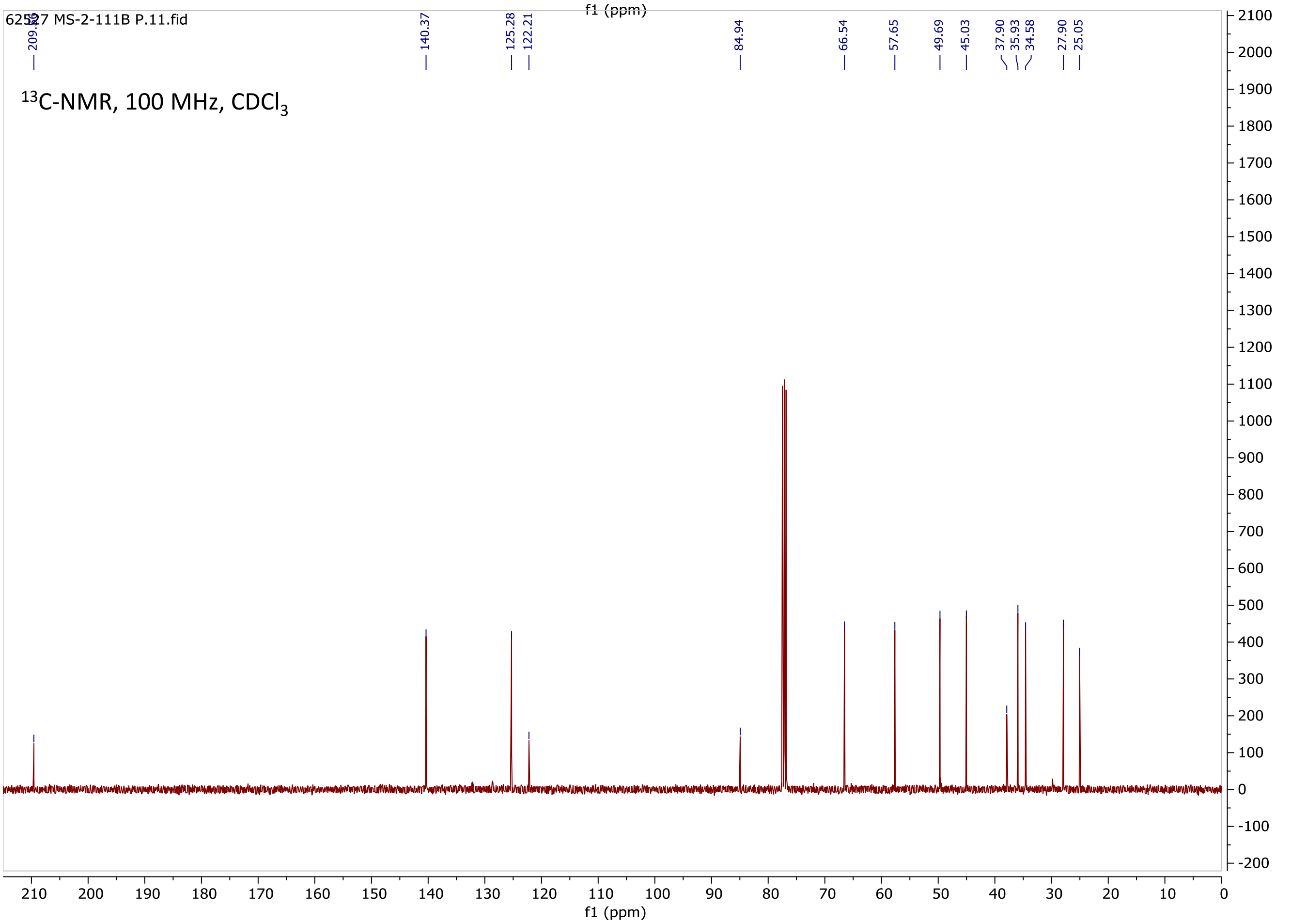

62527 MS-2-111B P.11.fid
^{13}C-NMR, 100 MHz, $CDCl_3$
209.12
140.37
125.28
122.21
84.94
66.54
57.65
49.69
45.03
37.90
35.93
34.58
27.90
25.05
f1 (ppm)

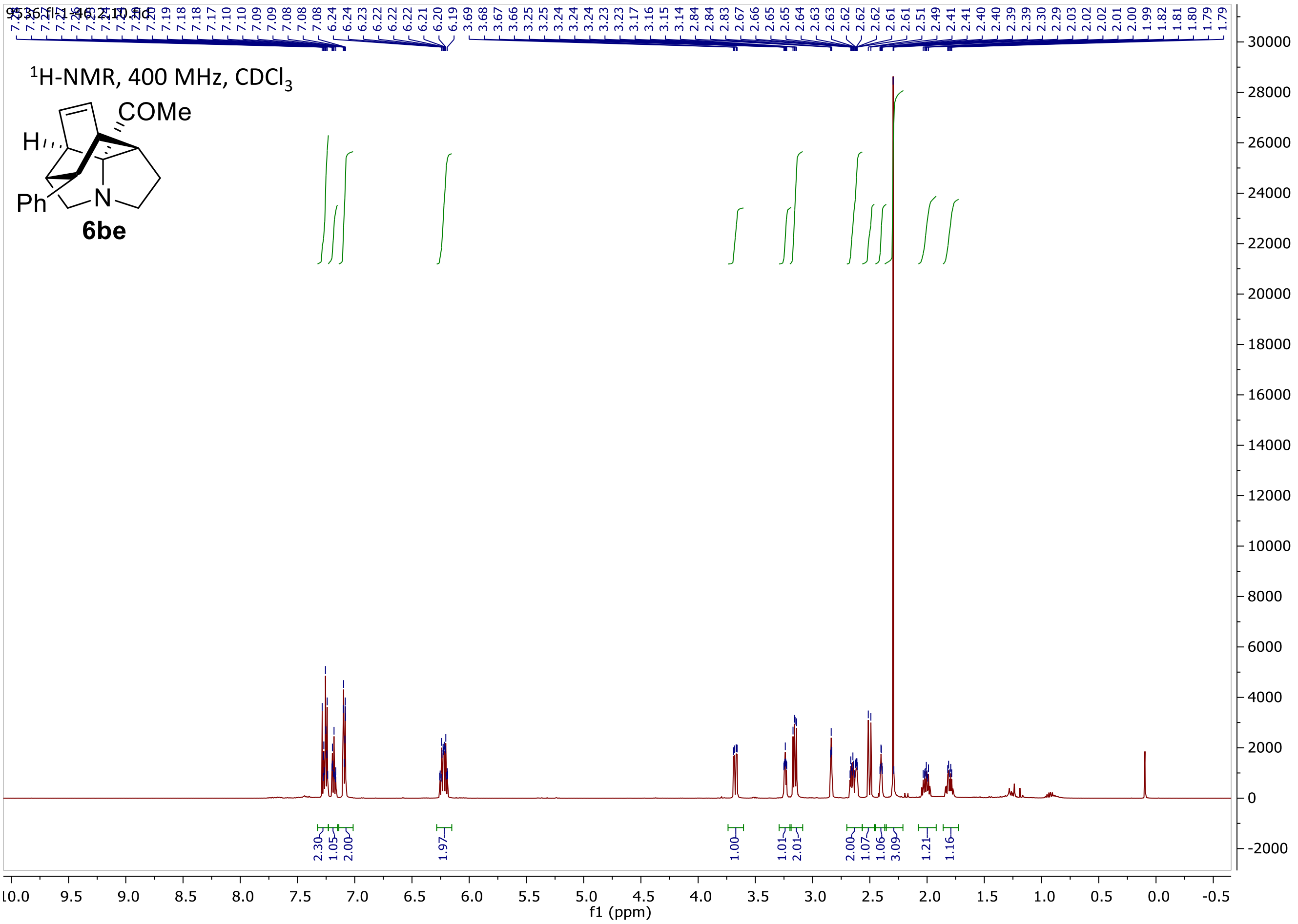
9536 fl-1-46.2.10.fid
^{1}H-NMR, 400 MHz, CDCl$_3$
COMe
H
Ph
N
6be
2.30
1.05
2.00
1.97
1.00
1.01
2.01
2.00
1.07
1.06
3.09
1.21
1.16
f1 (ppm)

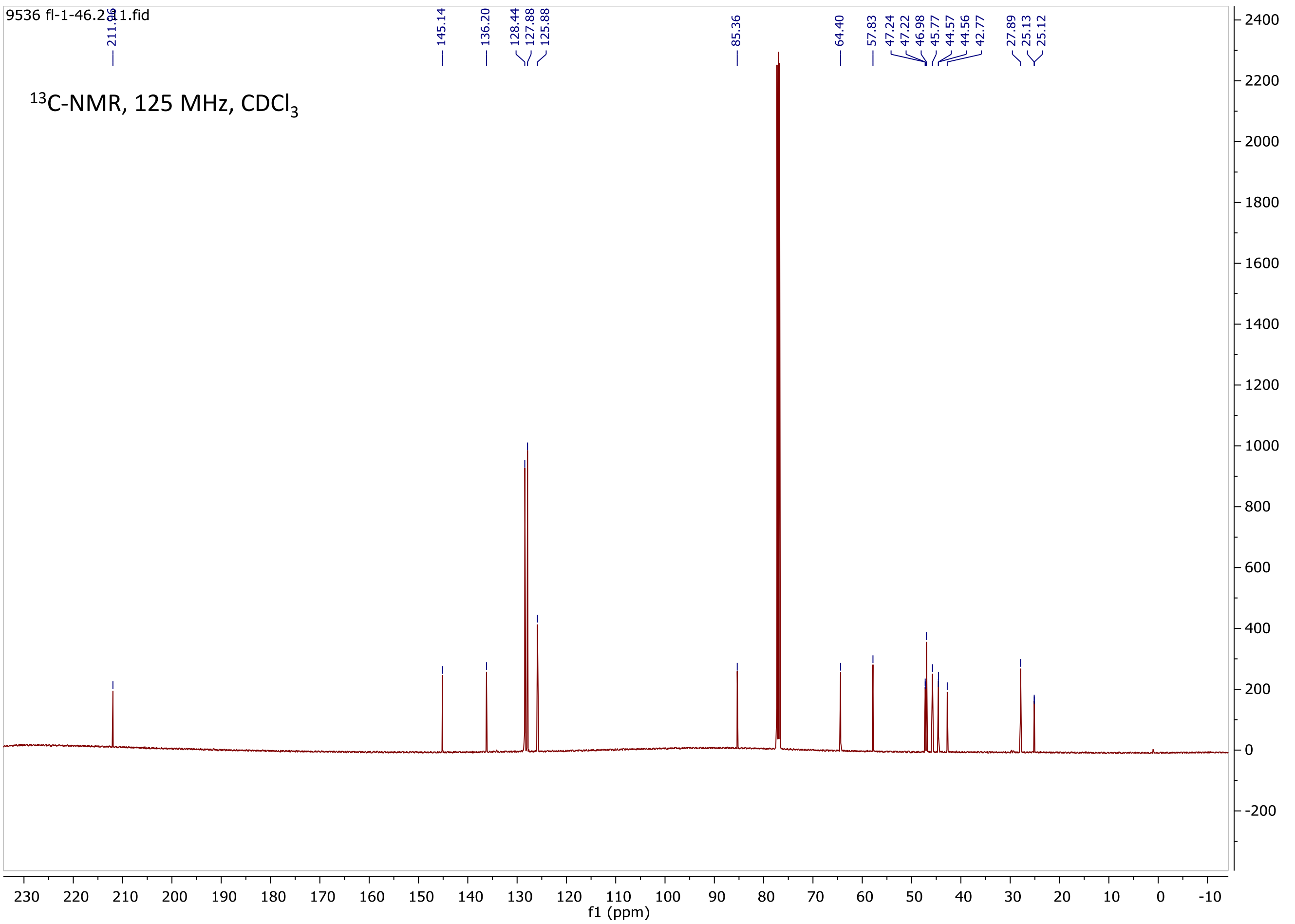
9536 fl-1-46.2.11.fid
^{13}C-NMR, 125 MHz, CDCl$_3$
145.14
136.20
128.44
127.88
125.88
85.36
64.40
57.83
47.24
47.22
46.98
45.77
44.57
44.56
42.77
27.89
25.13
25.12
f1 (ppm)

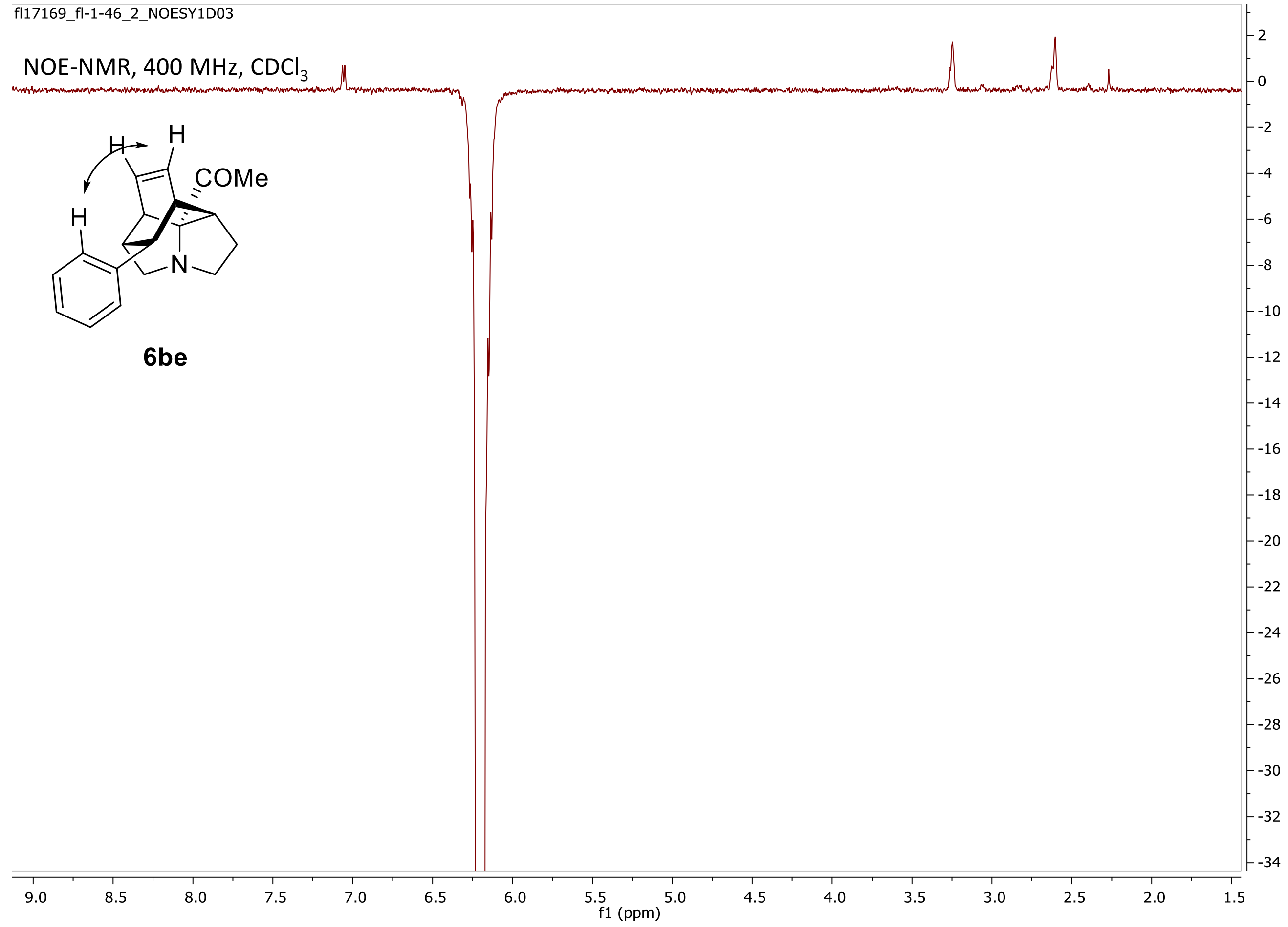
fl17169_fl-1-46_2_NOESY1D03
NOE-NMR, 400 MHz, CDCl3
H
H
COMe
H
N
6be
f1 (ppm)

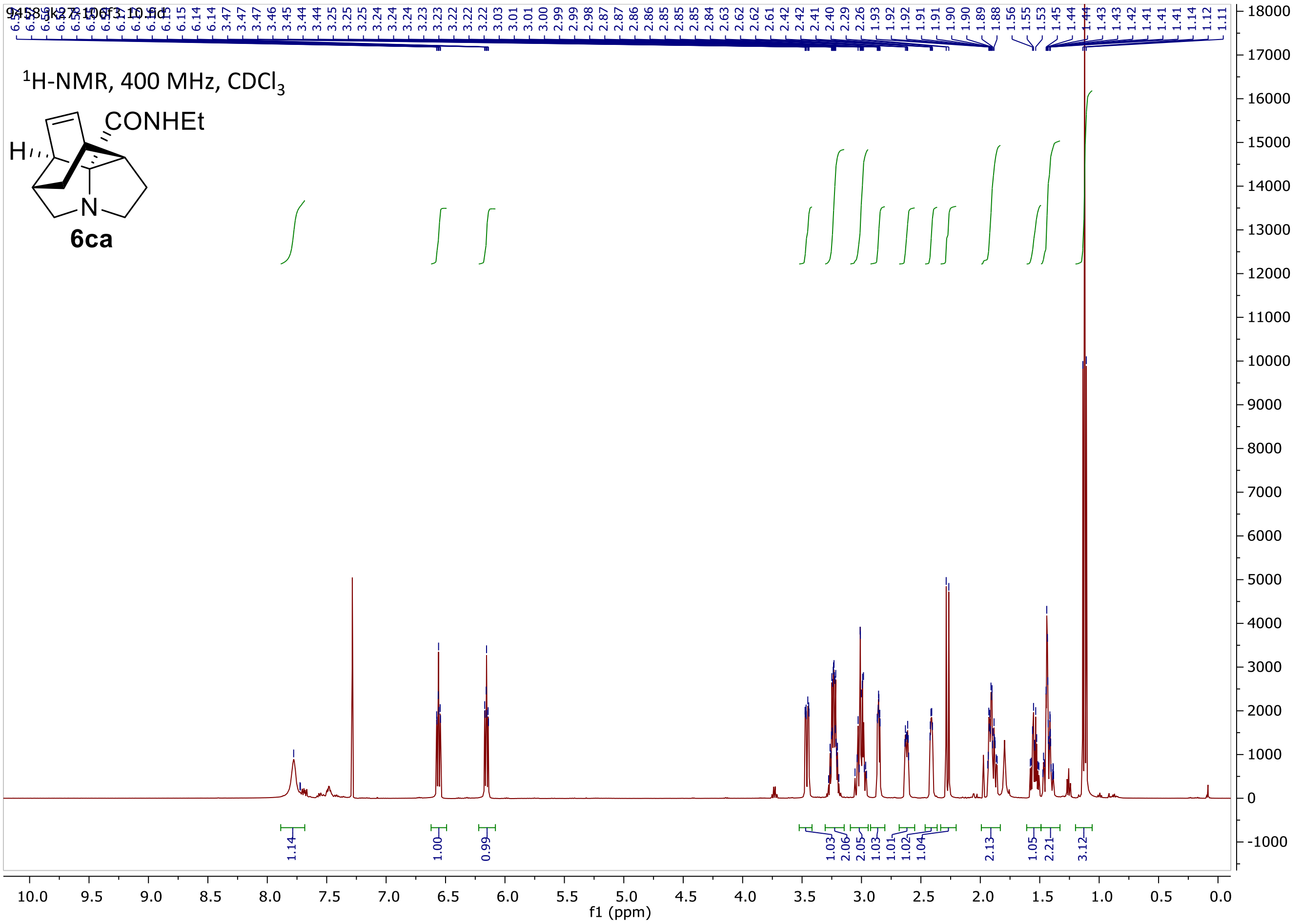
9458 jk27-106f3.10.fid
^{1}H-NMR, 400 MHz, $CDCl_3$
CONHEt
H
N
6ca
1.14
1.00
0.99
1.03
2.06
2.05
1.03
1.01
1.02
1.04
2.13
1.05
2.21
3.12
f1 (ppm)

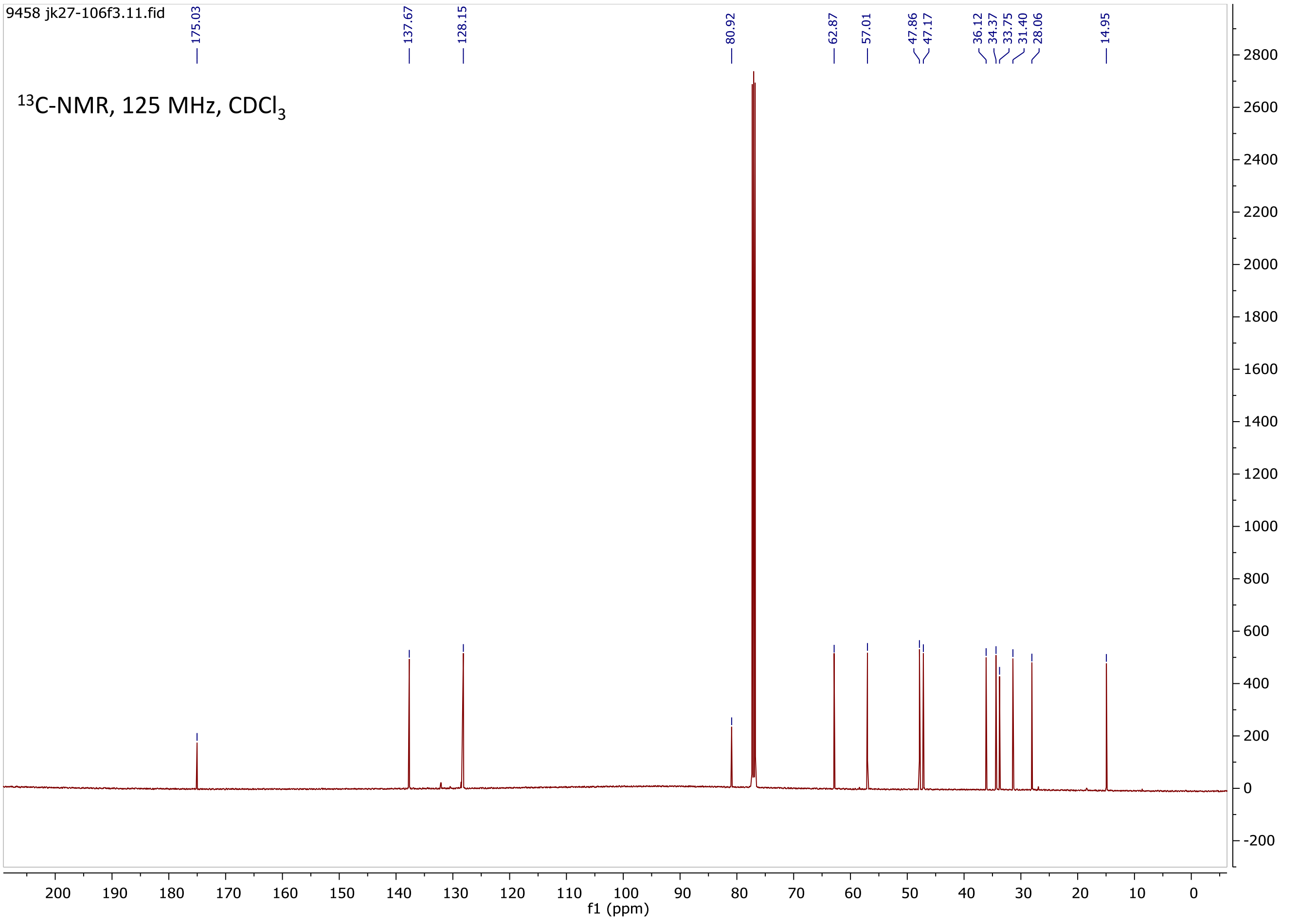
9458 jk27-106f3.11.fid
175.03
137.67
128.15
80.92
62.87
57.01
47.86
47.17
36.12
34.37
33.75
31.40
28.06
14.95
^{13}C-NMR, 125 MHz, $CDCl_3$
f1 (ppm)

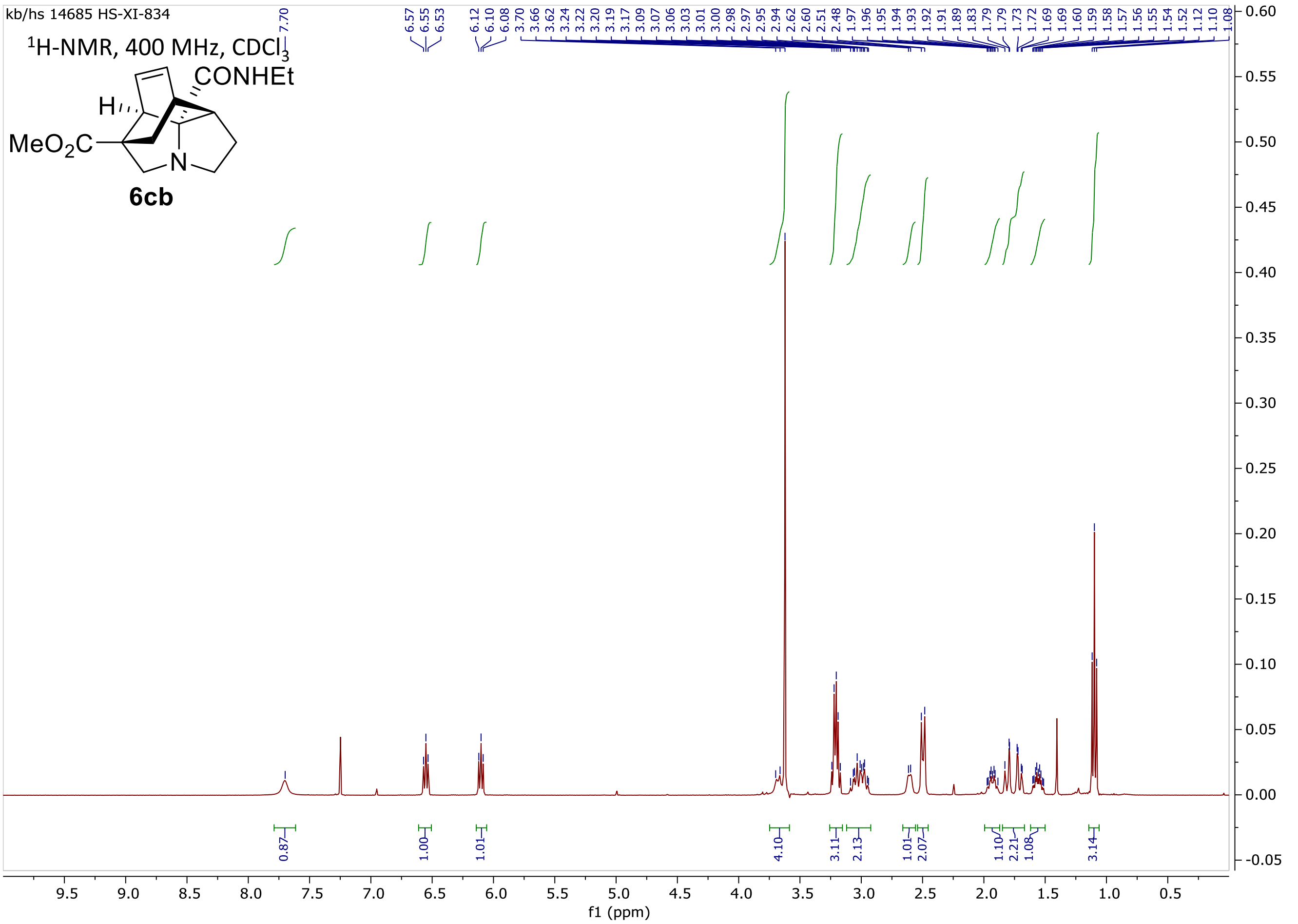
kb/hs 14685 HS-XI-834
¹H-NMR, 400 MHz, CDCl₃
CONHEt
MeO₂C
6cb
f1 (ppm)

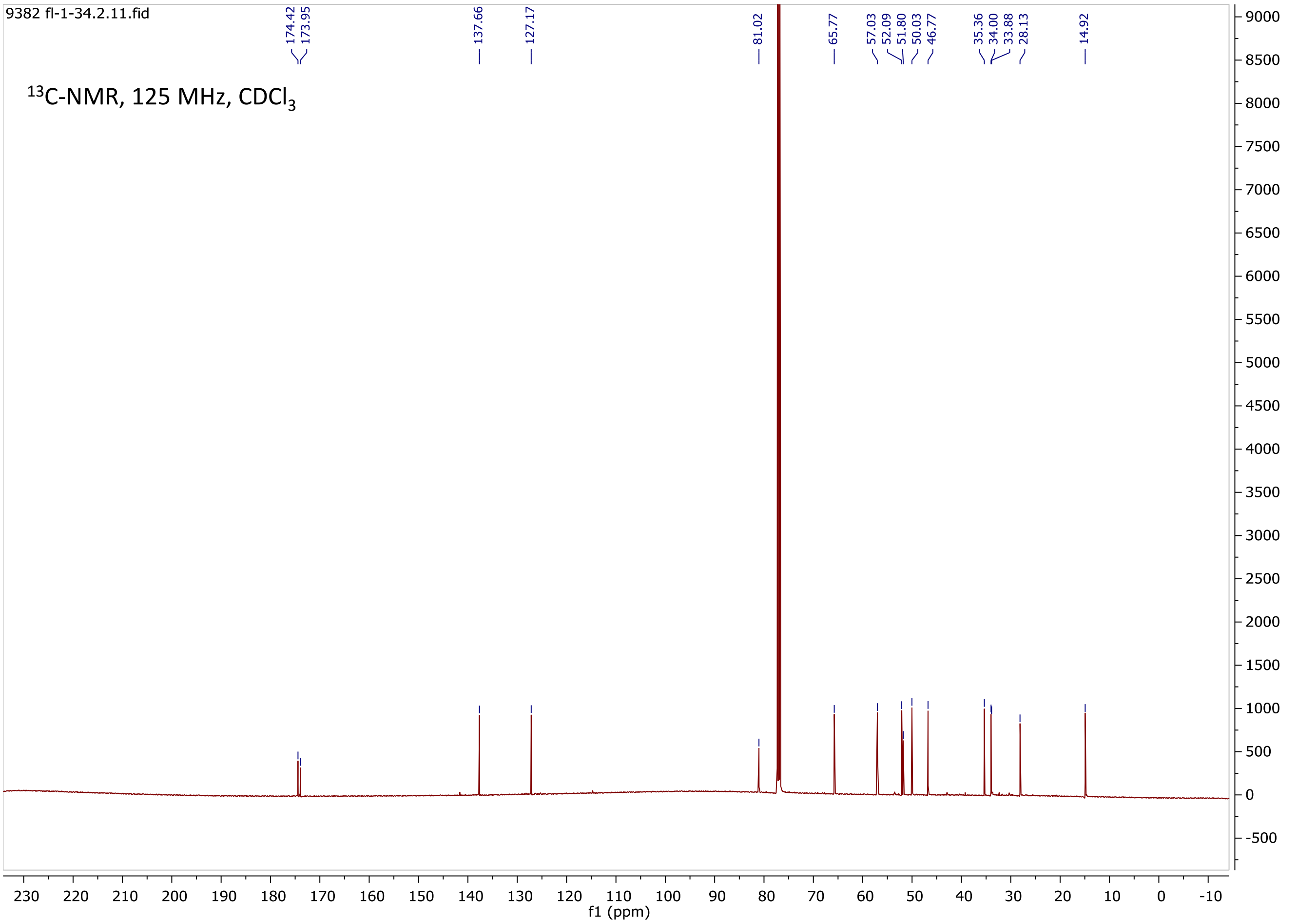
9382 fl-1-34.2.11.fid
¹³C-NMR, 125 MHz, CDCl₃
f1 (ppm)

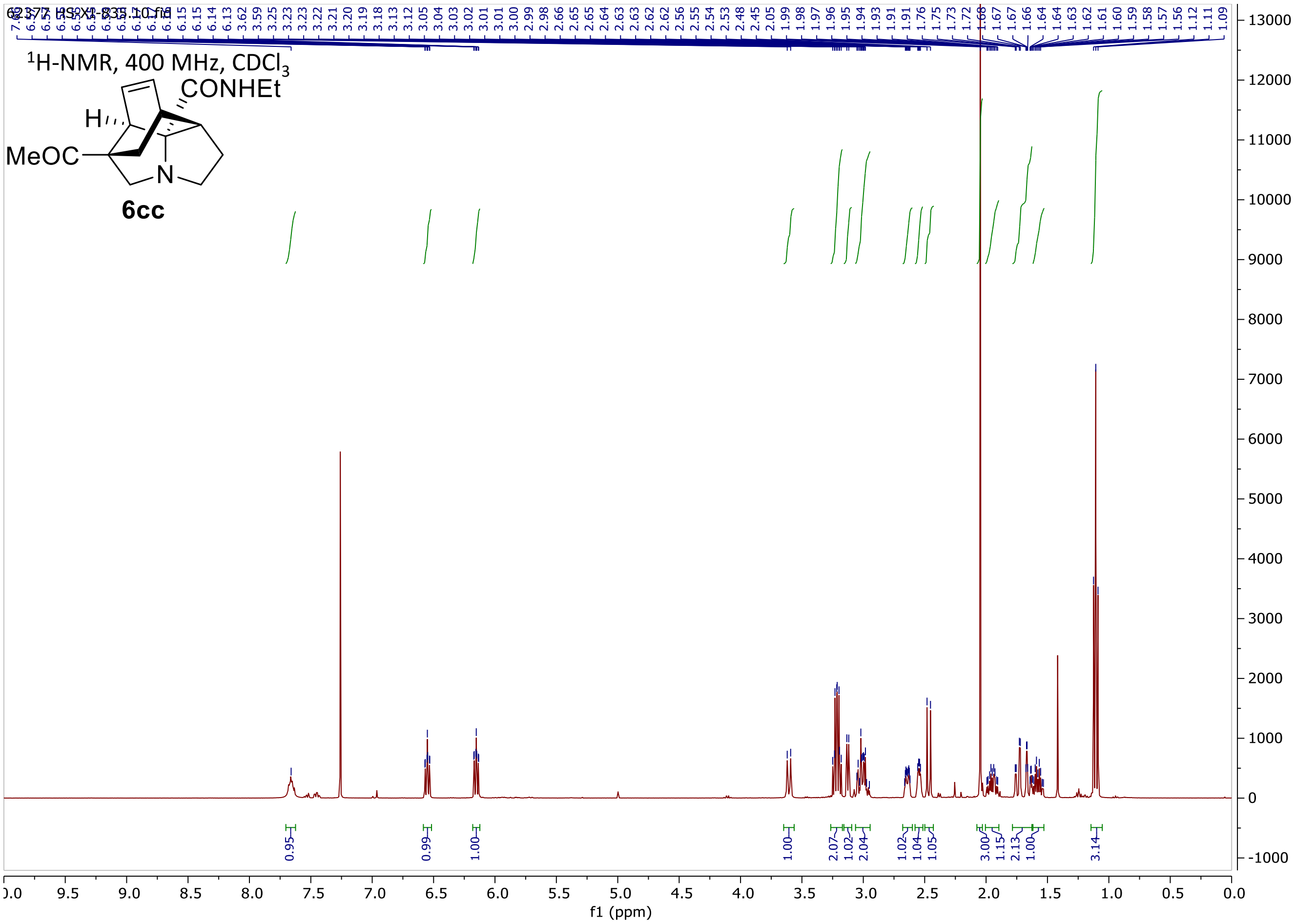
¹H-NMR, 400 MHz, CDCl₃
CONHEt
H
MeOC
N
6cc
0.95
0.99
1.00
1.00
2.07
1.02
2.04
1.02
1.04
1.05
3.00
1.15
2.13
1.00
3.14
f1 (ppm)

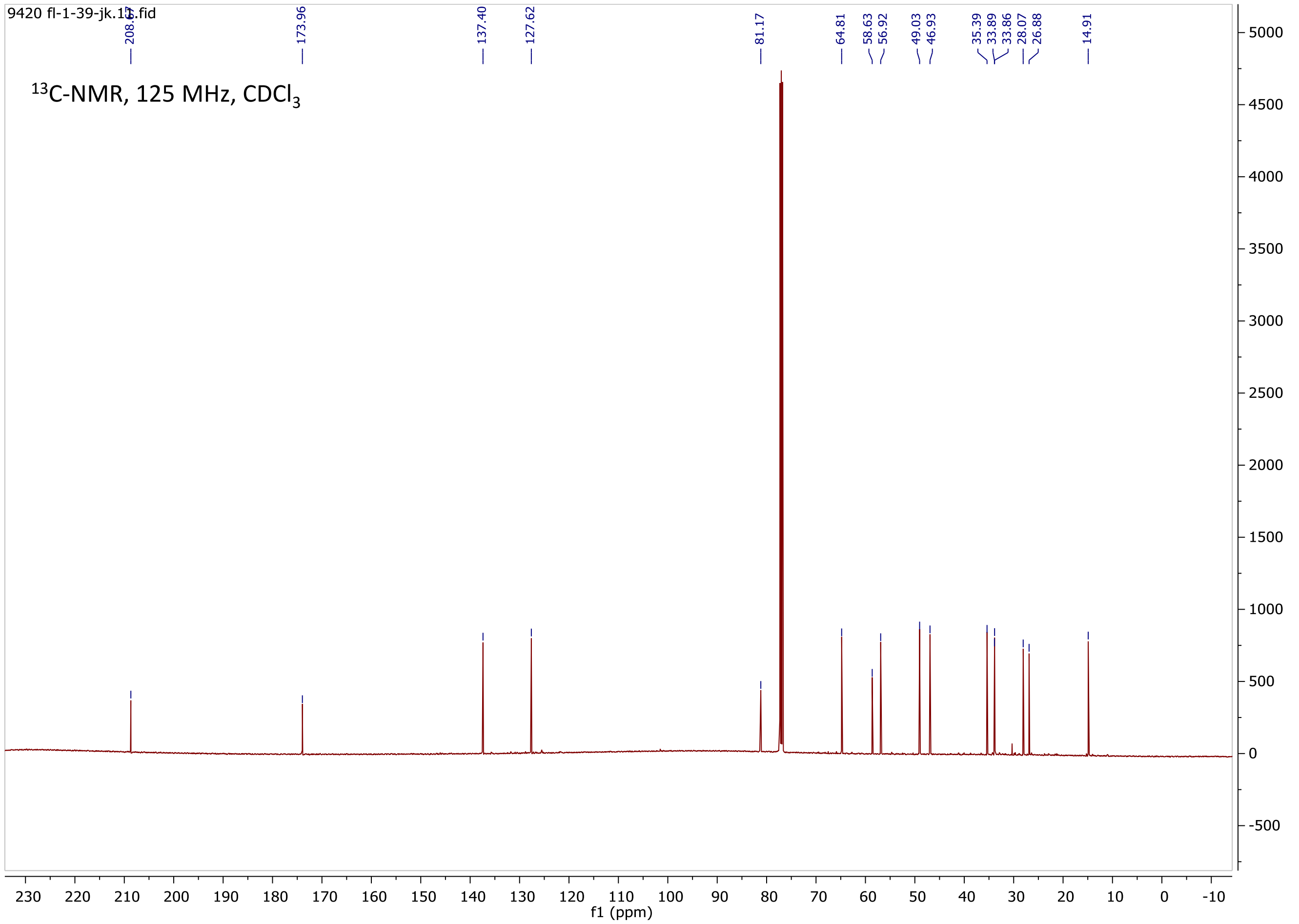
9420 fl-1-39-jk.11.fid
208.67
173.96
137.40
127.62
81.17
64.81
58.63
56.92
49.03
46.93
35.39
33.89
33.86
28.07
26.88
14.91
¹³C-NMR, 125 MHz, CDCl₃
f1 (ppm)

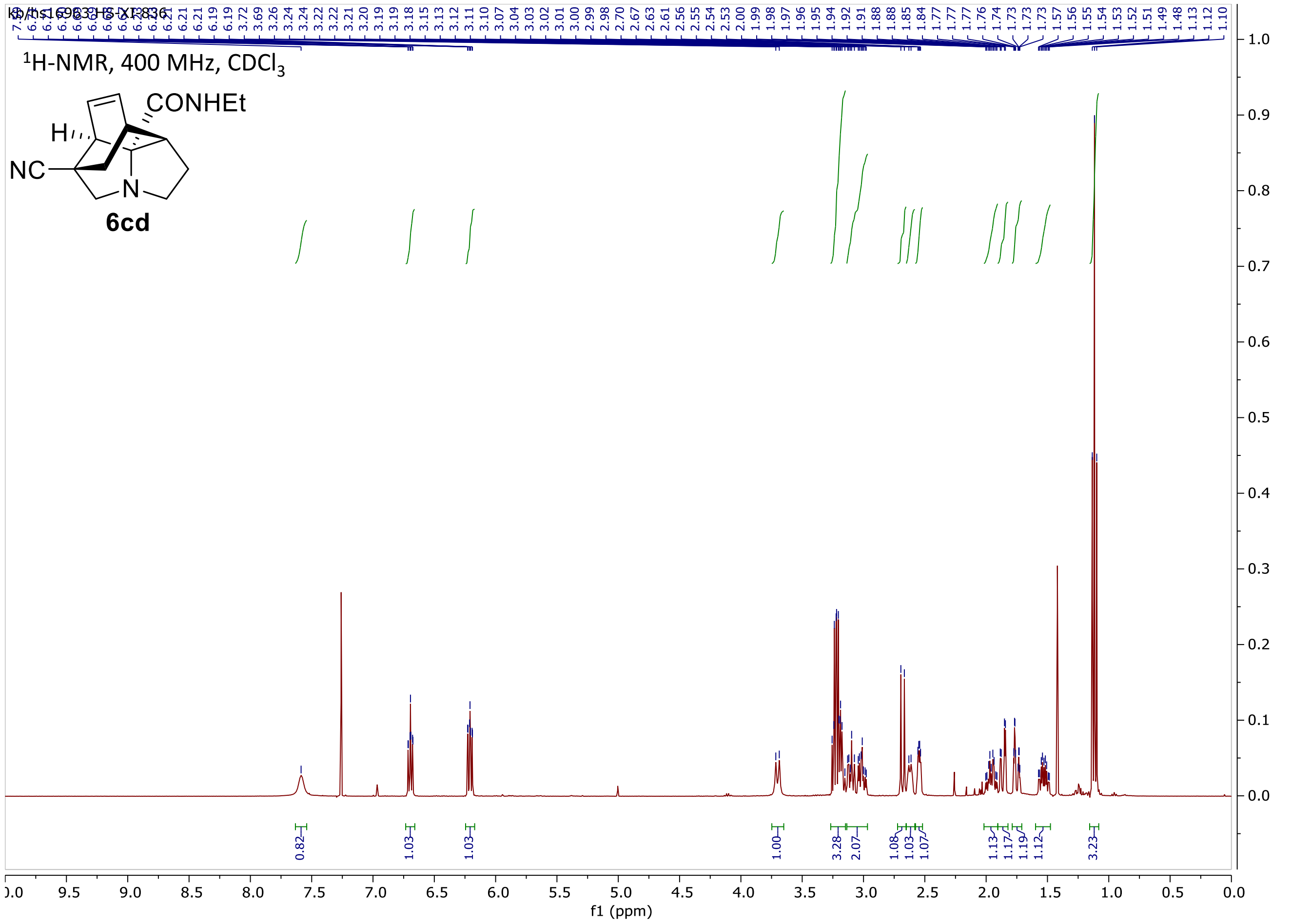
kb/hs16963 HS-XI-836
¹H-NMR, 400 MHz, CDCl₃
CONHEt
H
NC
N
6cd
f1 (ppm)

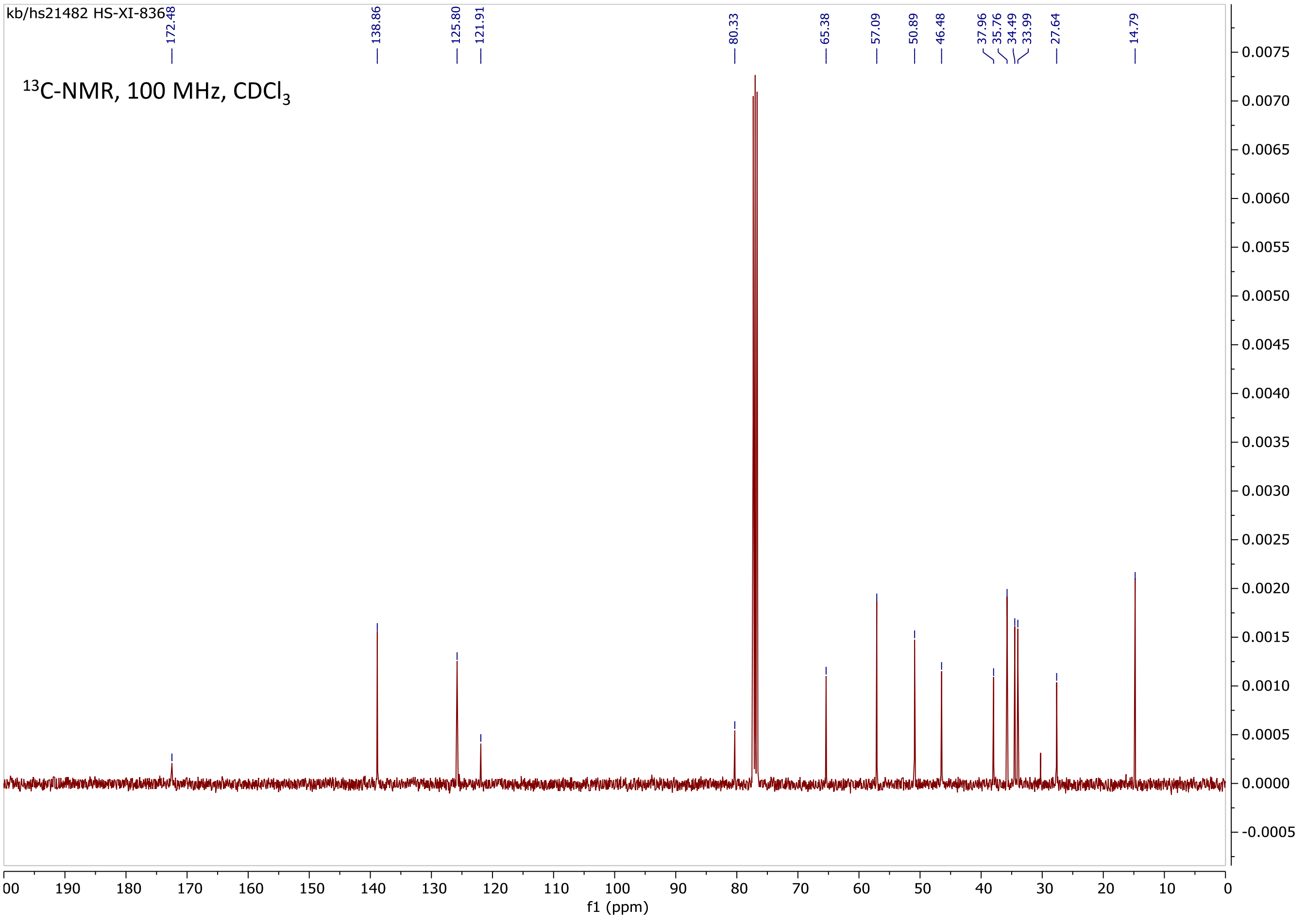
kb/hs21482 HS-XI-836
¹³C-NMR, 100 MHz, CDCl₃
172.48
138.86
125.80
121.91
80.33
65.38
57.09
50.89
46.48
37.96
35.76
34.49
33.99
27.64
14.79
f1 (ppm)

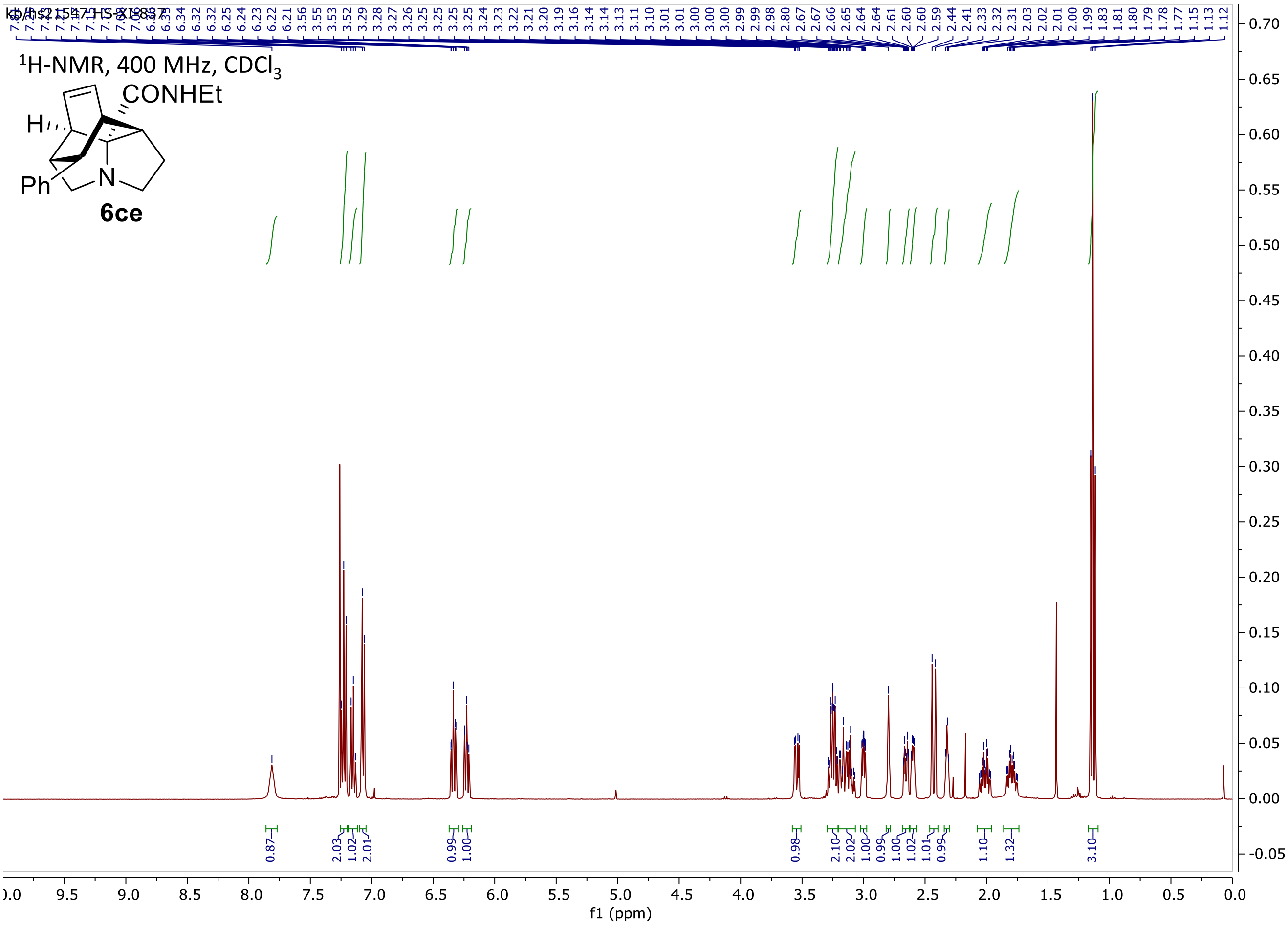

¹H-NMR, 400 MHz, CDCl₃
CONHEt
H
Ph
N
6ce
f1 (ppm)

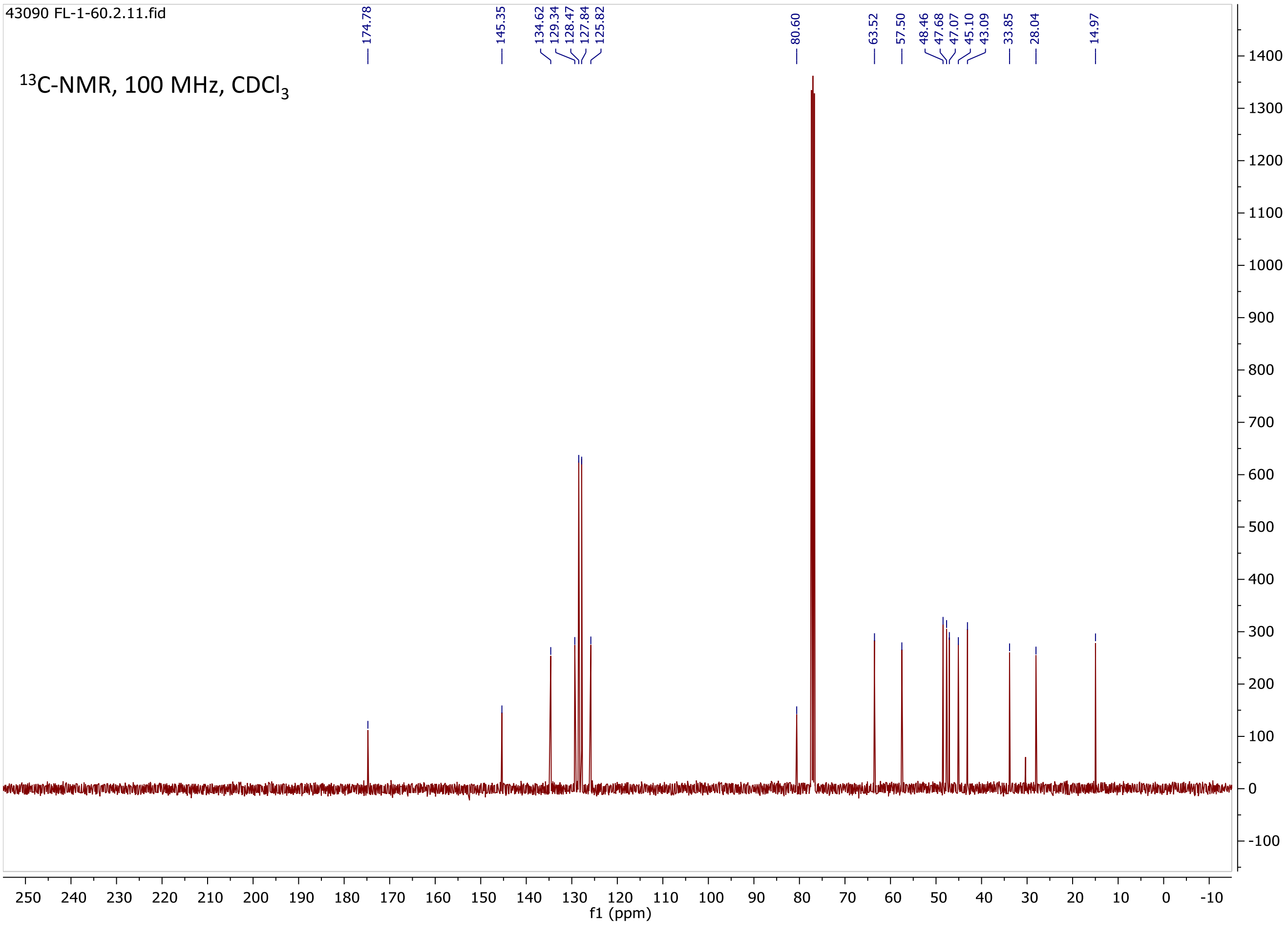

43090 FL-1-60.2.11.fid
¹³C-NMR, 100 MHz, CDCl₃
174.78
145.35
134.62
129.34
128.47
127.84
125.82
80.60
63.52
57.50
48.46
47.68
47.07
45.10
43.09
33.85
28.04
14.97
f1 (ppm)

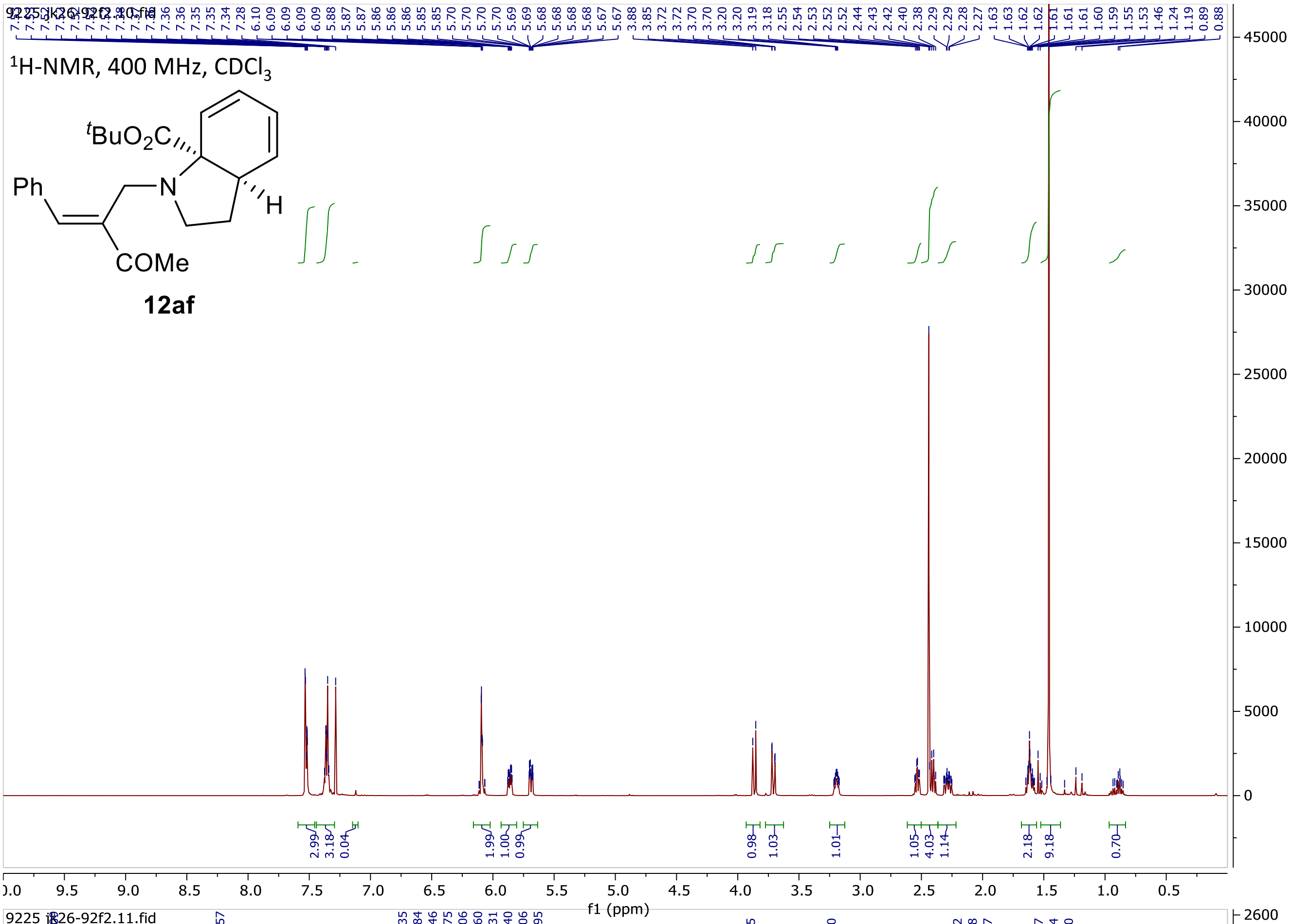
9225 jk26-92f2.10.fid
1H-NMR, 400 MHz, CDCl3
tBuO2C
Ph
N
H
COMe
12af
f1 (ppm)

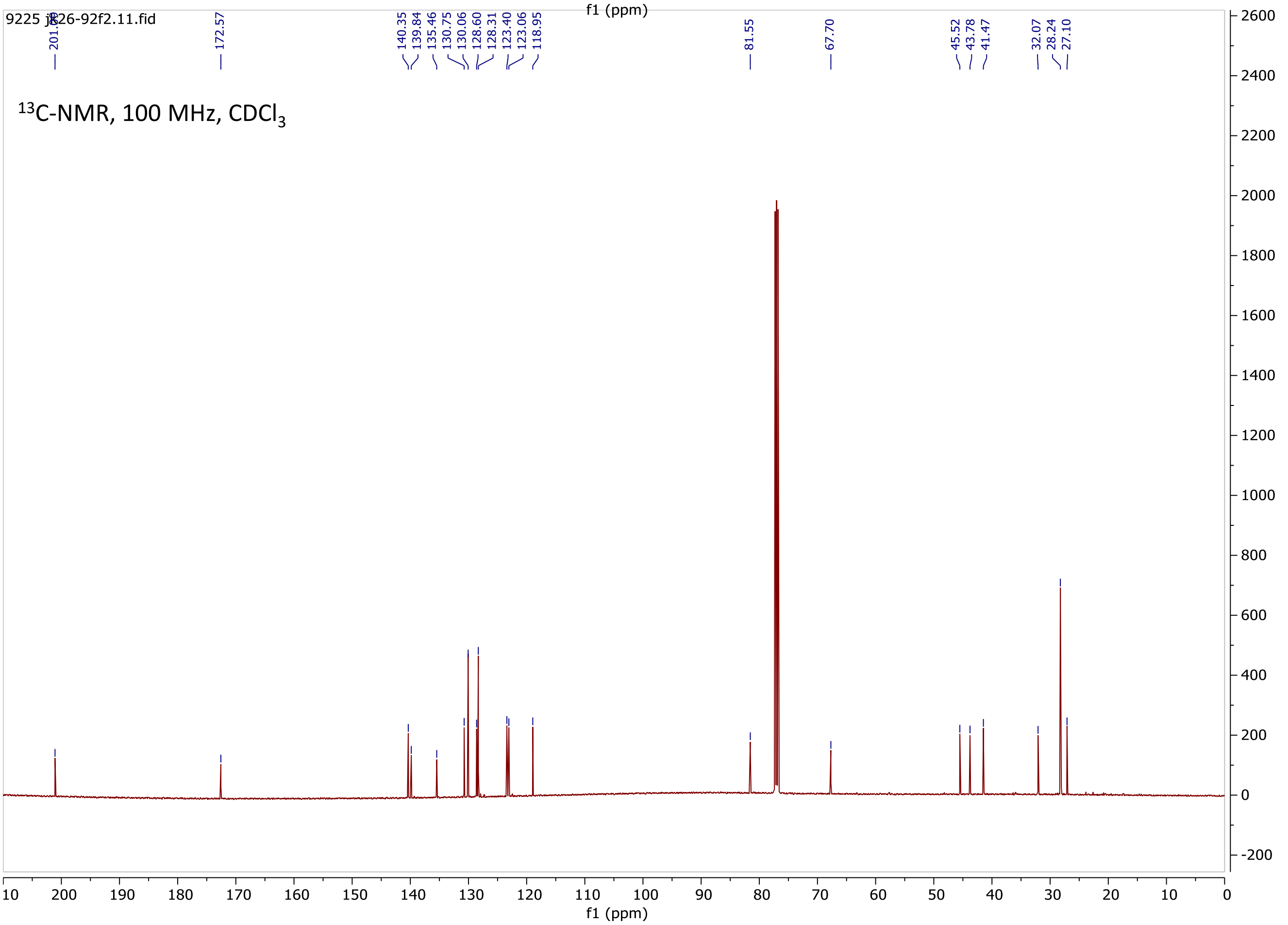
9225 jk26-92f2.11.fid
13C-NMR, 100 MHz, CDCl3
f1 (ppm)

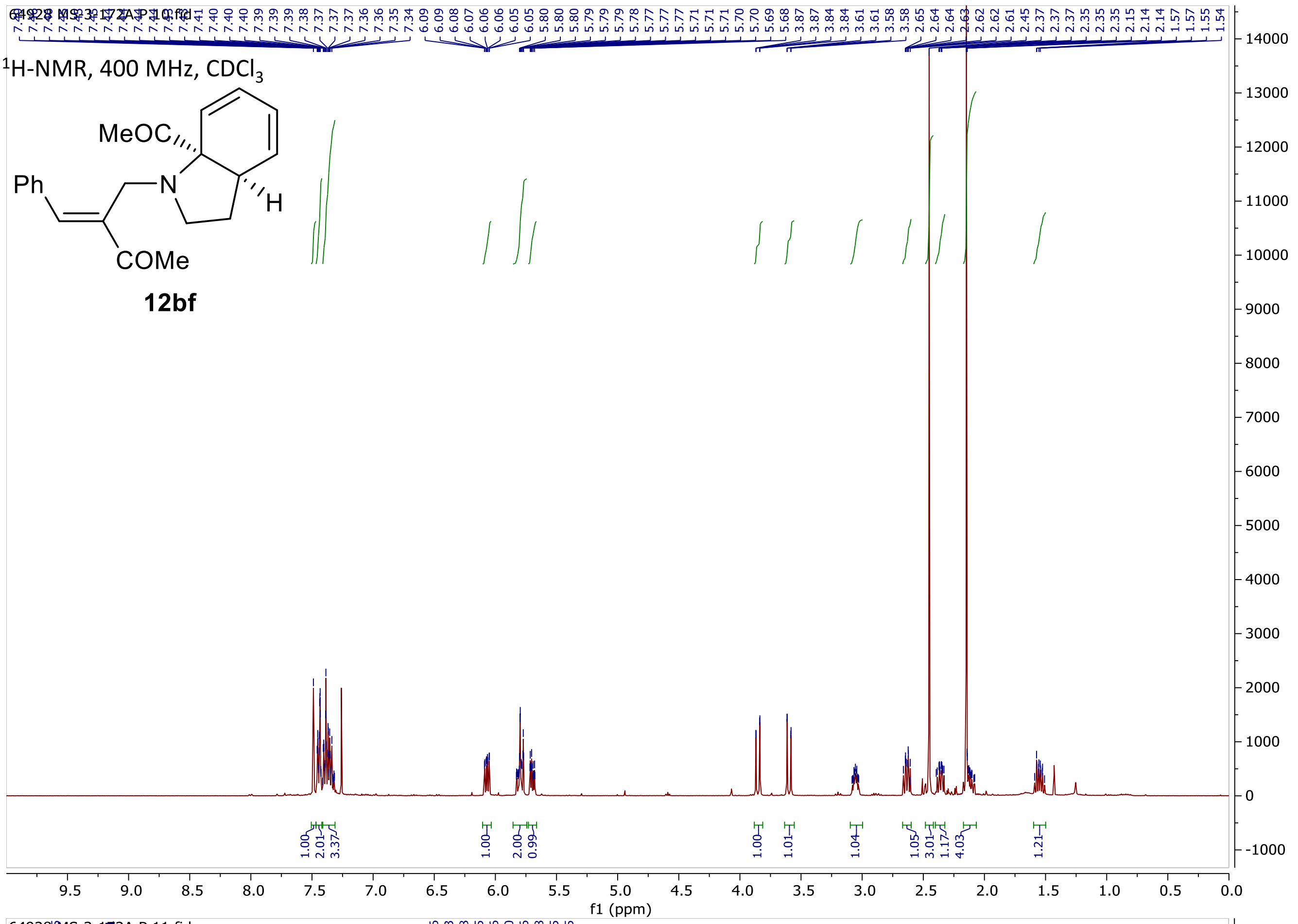

7.41
7.40
7.40
7.40
7.39
7.39
7.39
7.38
7.37
7.37
7.37
7.36
7.35
7.34
6.09
6.09
6.08
6.07
6.06
6.06
6.05
6.05
5.80
5.80
5.80
5.79
5.79
5.79
5.78
5.77
5.77
5.77
5.71
5.71
5.71
5.70
5.70
5.69
5.68
3.87
3.87
3.84
3.84
3.61
3.61
3.58
3.58
2.65
2.64
2.64
2.63
2.62
2.62
2.61
2.45
2.37
2.37
2.37
2.35
2.35
2.35
2.15
2.14
2.14
1.57
1.57
1.55
1.54
^{1}H-NMR, 400 MHz, $CDCl_3$
MeOC
Ph
N
H
COMe
12bf
1.00
2.01
3.37
1.00
2.00
0.99
1.00
1.01
1.04
1.05
3.01
1.17
4.03
1.21
f1 (ppm)

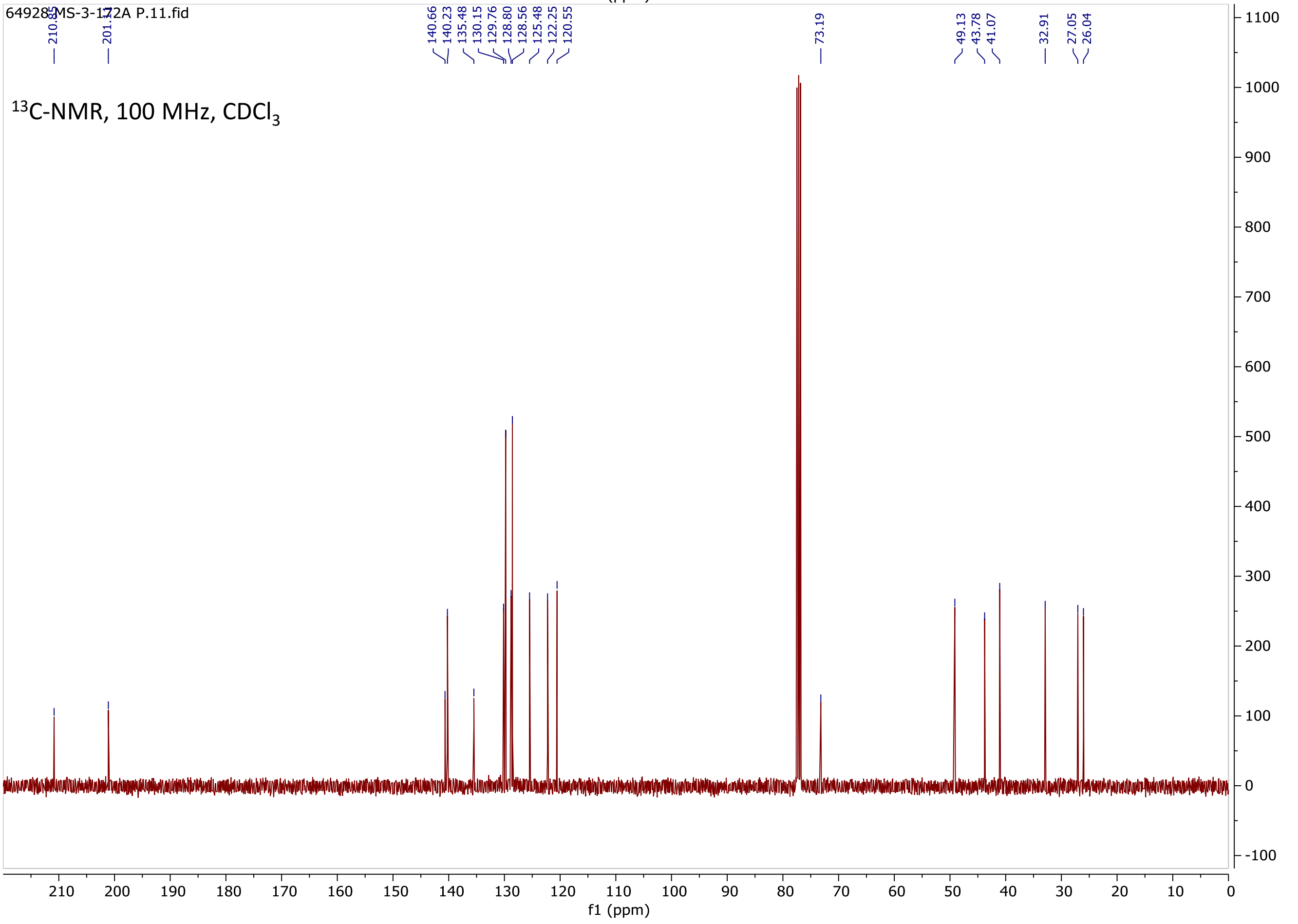

64928 MS-3-172A P.11.fid
140.66
140.23
135.48
130.15
129.76
128.80
128.56
125.48
122.25
120.55
73.19
49.13
43.78
41.07
32.91
27.05
26.04
^{13}C-NMR, 100 MHz, $CDCl_3$
f1 (ppm)

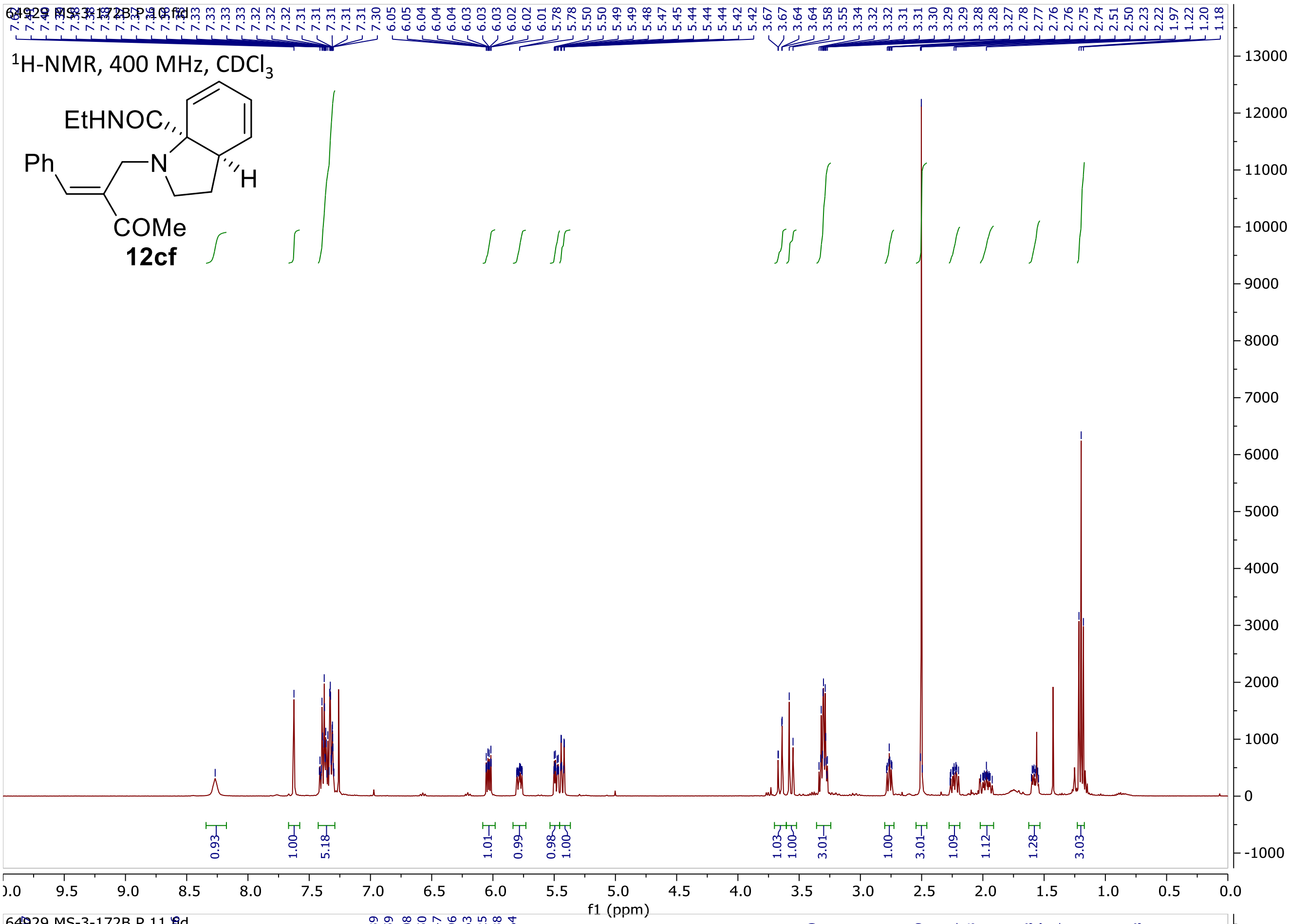
64929 MS-3-172B P.10.fid
¹H-NMR, 400 MHz, CDCl₃
EtHNOC
Ph
N
H
COMe
12cf
0.93
1.00
5.18
1.01
0.99
0.98
1.00
1.03
1.00
3.01
1.00
3.01
1.09
1.12
1.28
3.03
f1 (ppm)

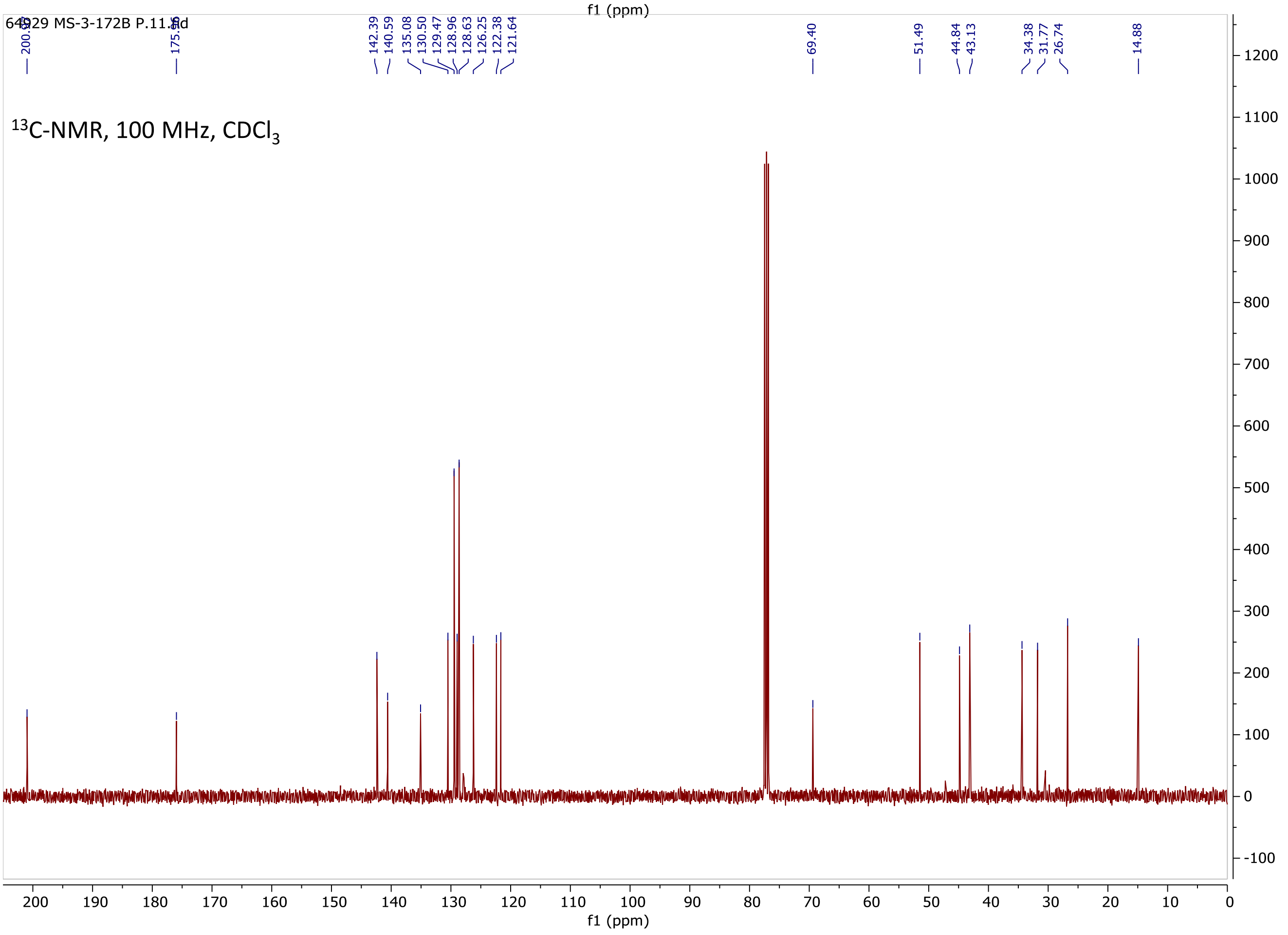
64929 MS-3-172B P.11.fid
¹³C-NMR, 100 MHz, CDCl₃
142.39
140.59
135.08
130.50
129.47
128.96
128.63
126.25
122.38
121.64
69.40
51.49
44.84
43.13
34.38
31.77
26.74
14.88
f1 (ppm)

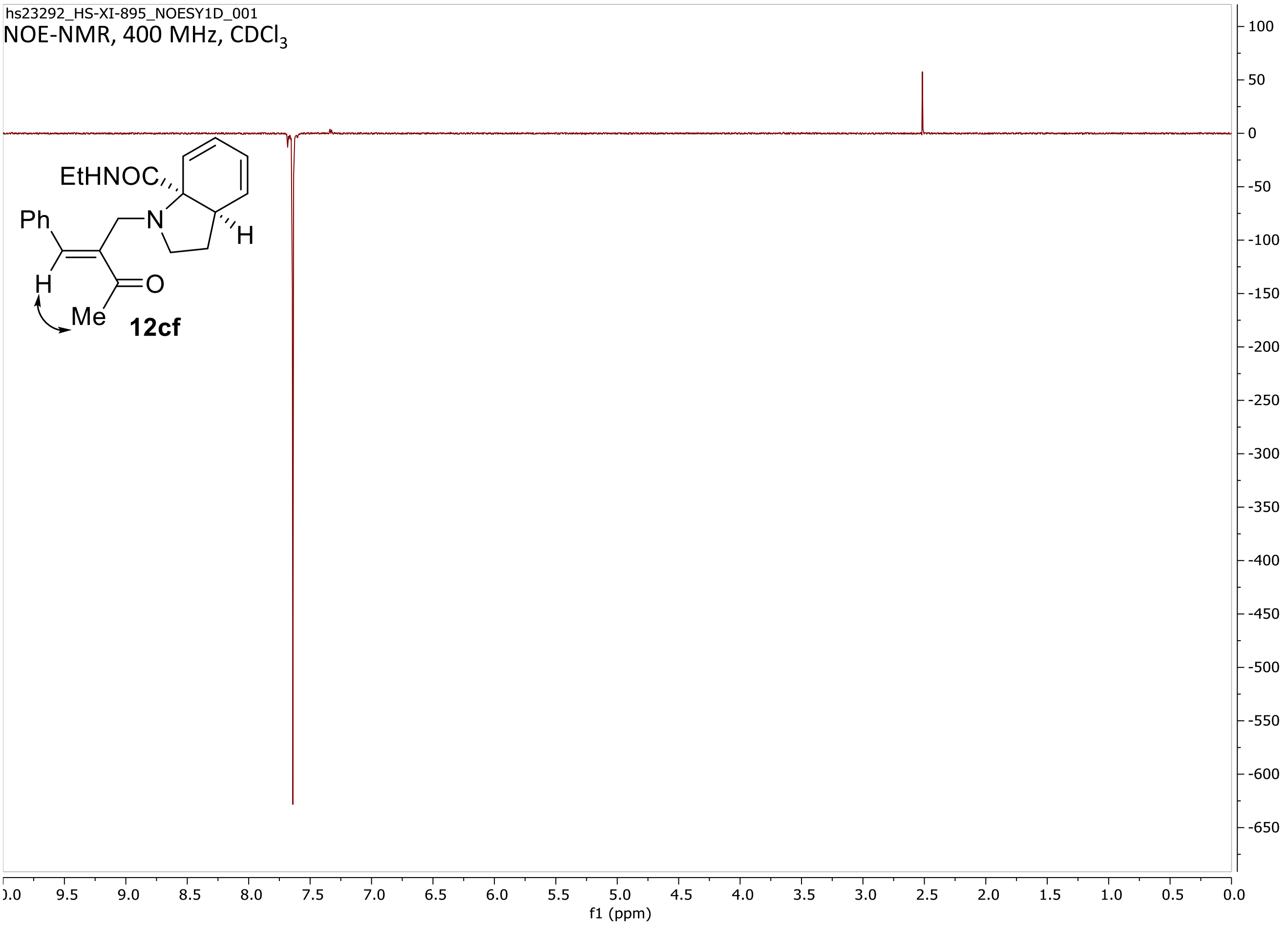

hs23292_HS-XI-895_NOESY1D_001
NOE-NMR, 400 MHz, CDCl3
EtHNOC
Ph
N
H
H
O
Me
12cf
f1 (ppm)

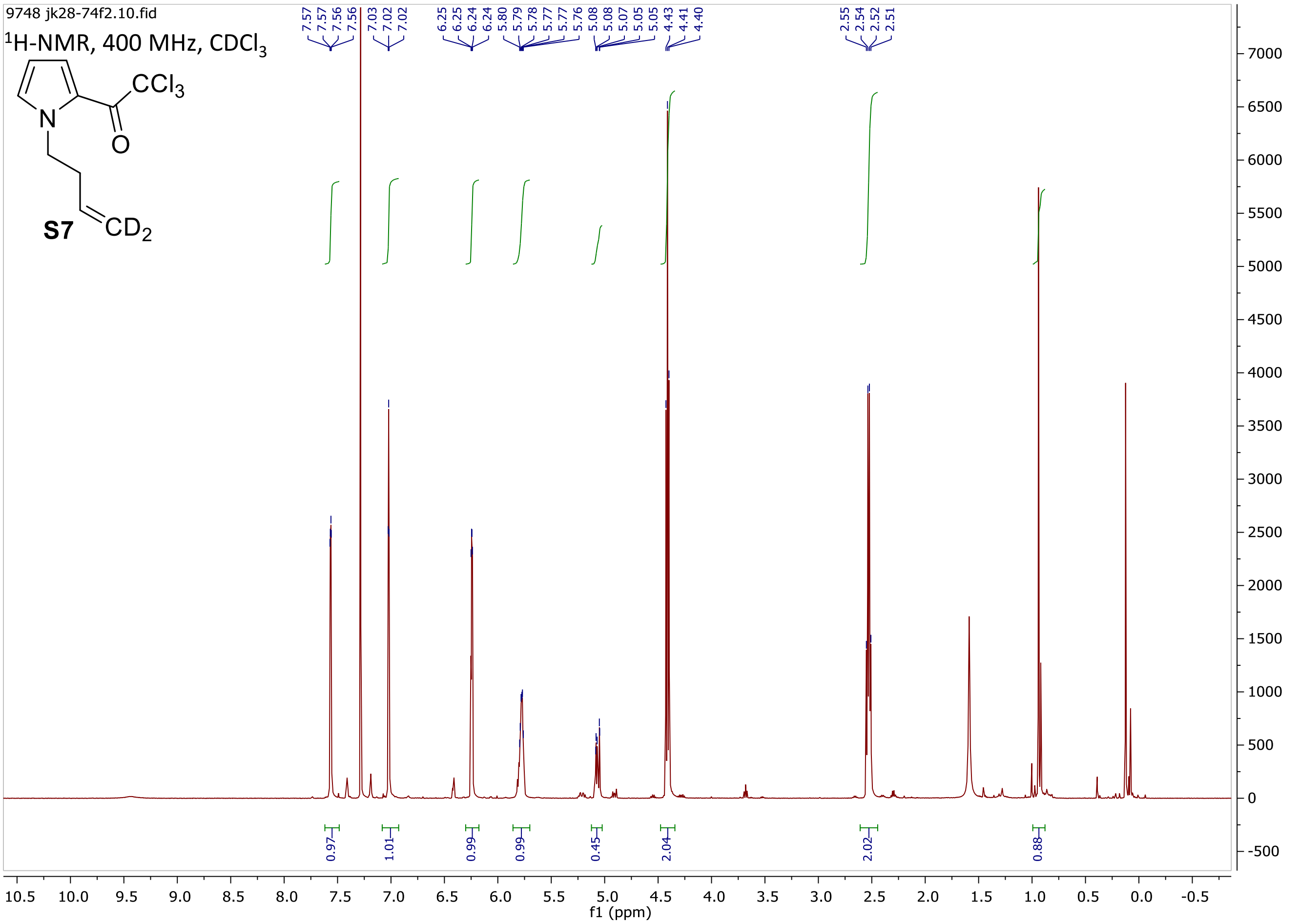
9748 jk28-74f2.10.fid
1H-NMR, 400 MHz, CDCl3
CCl3
N
O
S7
CD2
7.57 7.57 7.56 7.56 7.03 7.02 7.02 6.25 6.25 6.24 6.24 5.80 5.79 5.78 5.77 5.77 5.76 5.08 5.08 5.07 5.05 5.05 4.43 4.41 4.40 2.55 2.54 2.52 2.51
0.97 1.01 0.99 0.99 0.45 2.04 2.02 0.88
f1 (ppm)

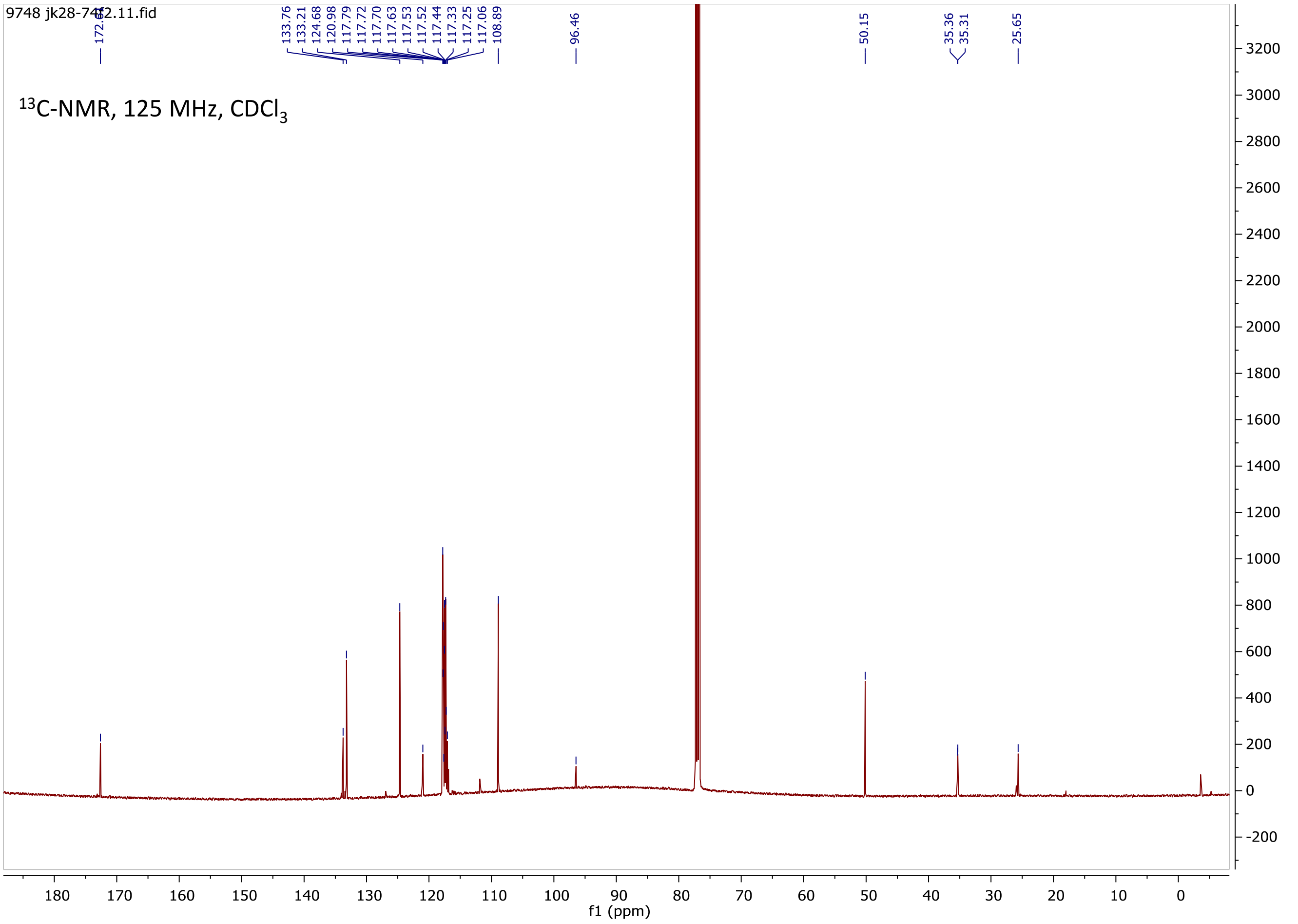
9748 jk28-74f2.11.fid
13C-NMR, 125 MHz, CDCl3
172.6 133.76 133.21 124.68 120.98 117.79 117.72 117.70 117.63 117.53 117.52 117.44 117.33 117.25 117.06 108.89 96.46 50.15 35.36 35.31 25.65
f1 (ppm)

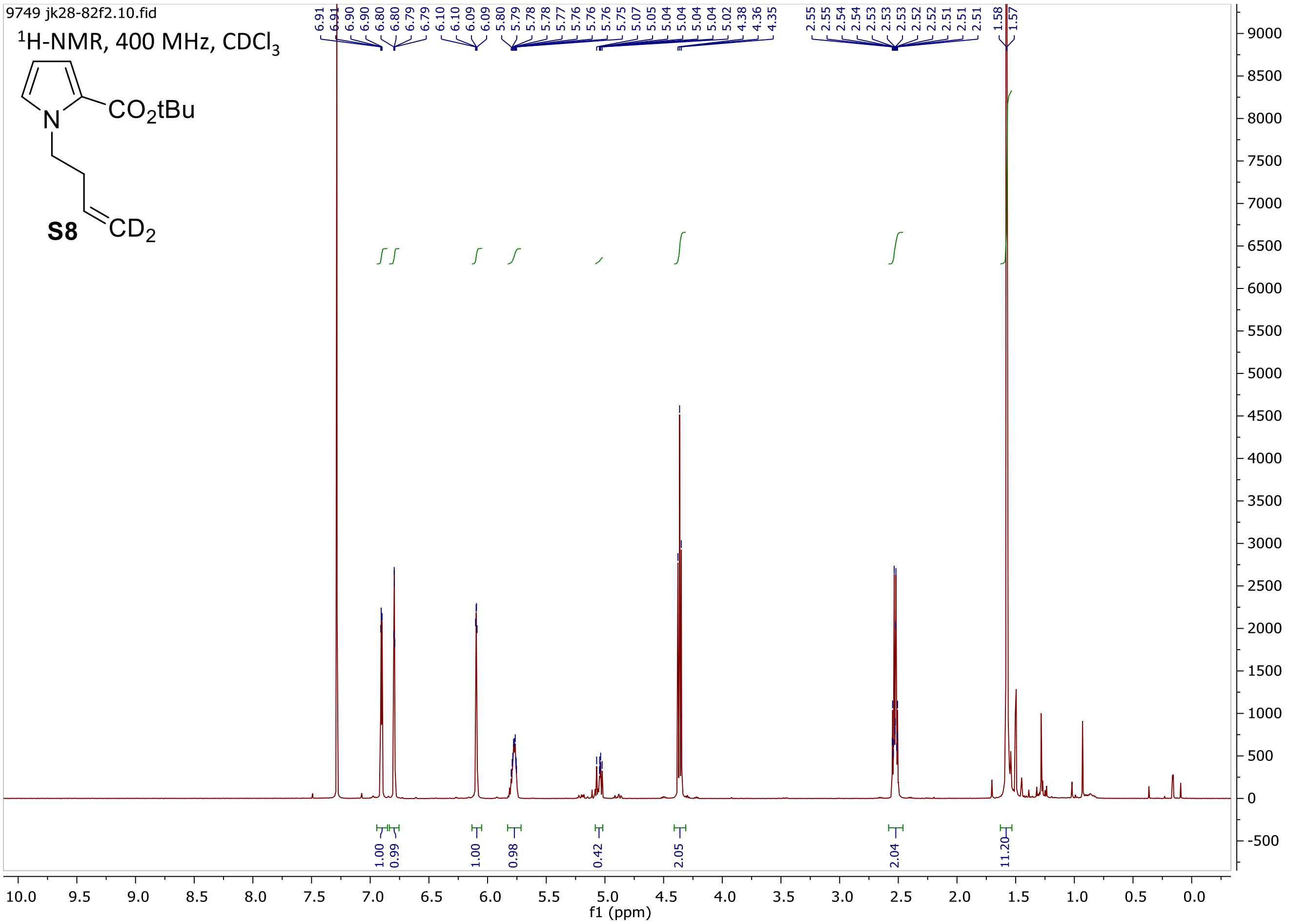
9749 jk28-82f2.10.fid
^{1}H-NMR, 400 MHz, $CDCl_3$
CO_2tBu
N
S8
CD_2
f1 (ppm)

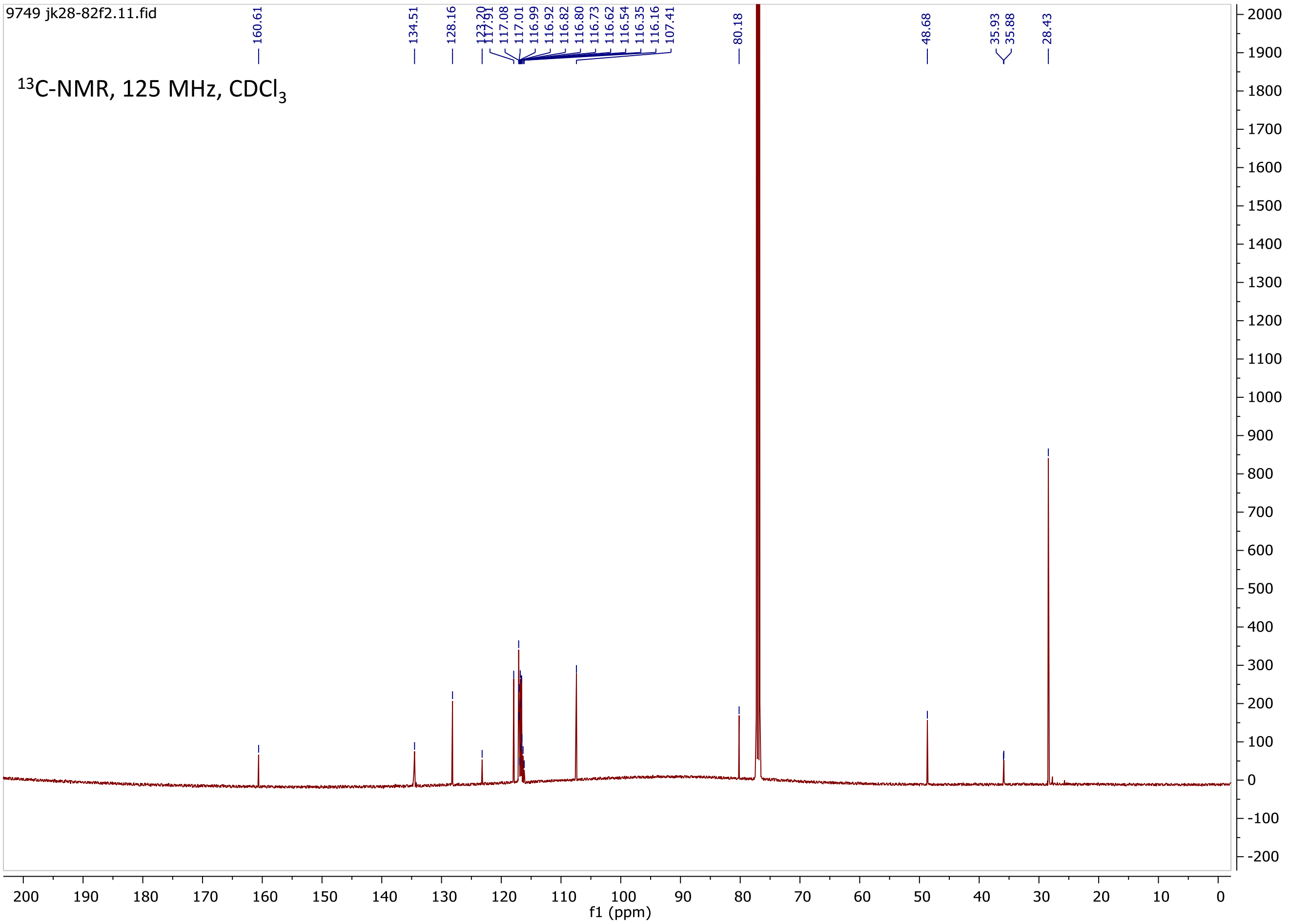
9749 jk28-82f2.11.fid
^{13}C-NMR, 125 MHz, $CDCl_3$
f1 (ppm)

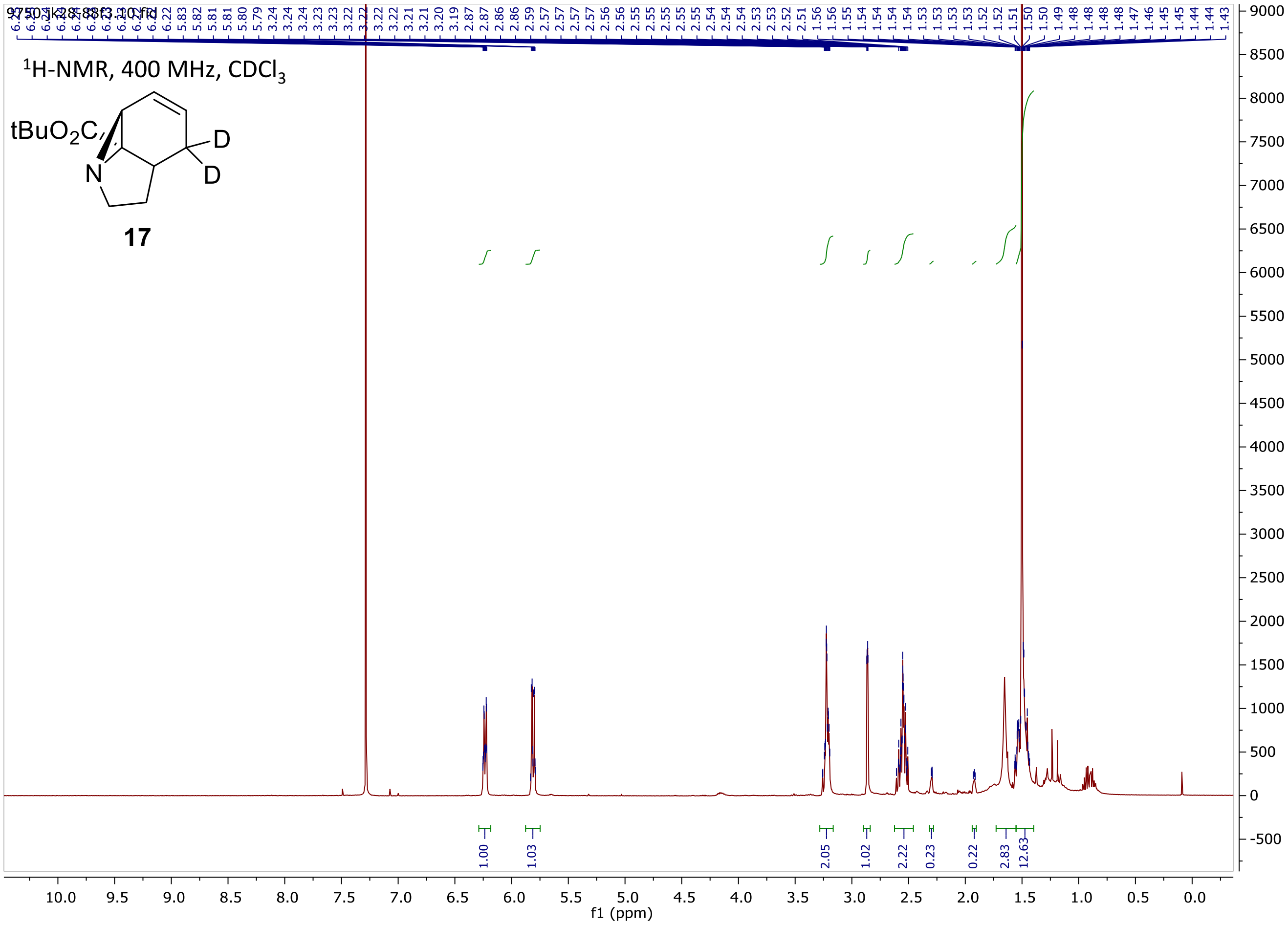
9750 jk28-88f3.10.fid
¹H-NMR, 400 MHz, CDCl3
tBuO2C
N
D
D
17
f1 (ppm)

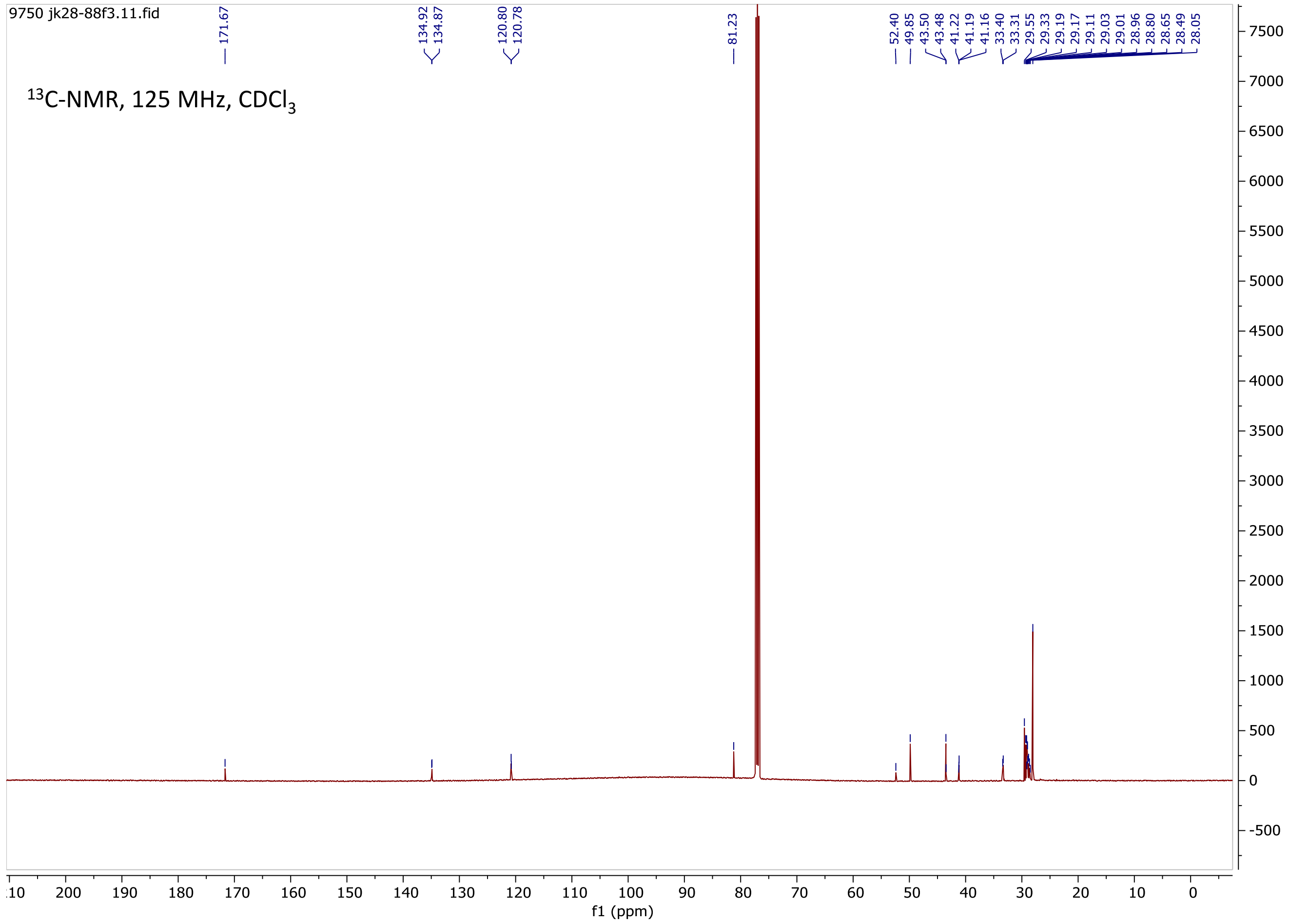
9750 jk28-88f3.11.fid
¹³C-NMR, 125 MHz, CDCl3
171.67
134.92
134.87
120.80
120.78
81.23
52.40
49.85
43.50
43.48
41.22
41.19
41.16
33.40
33.31
f1 (ppm)

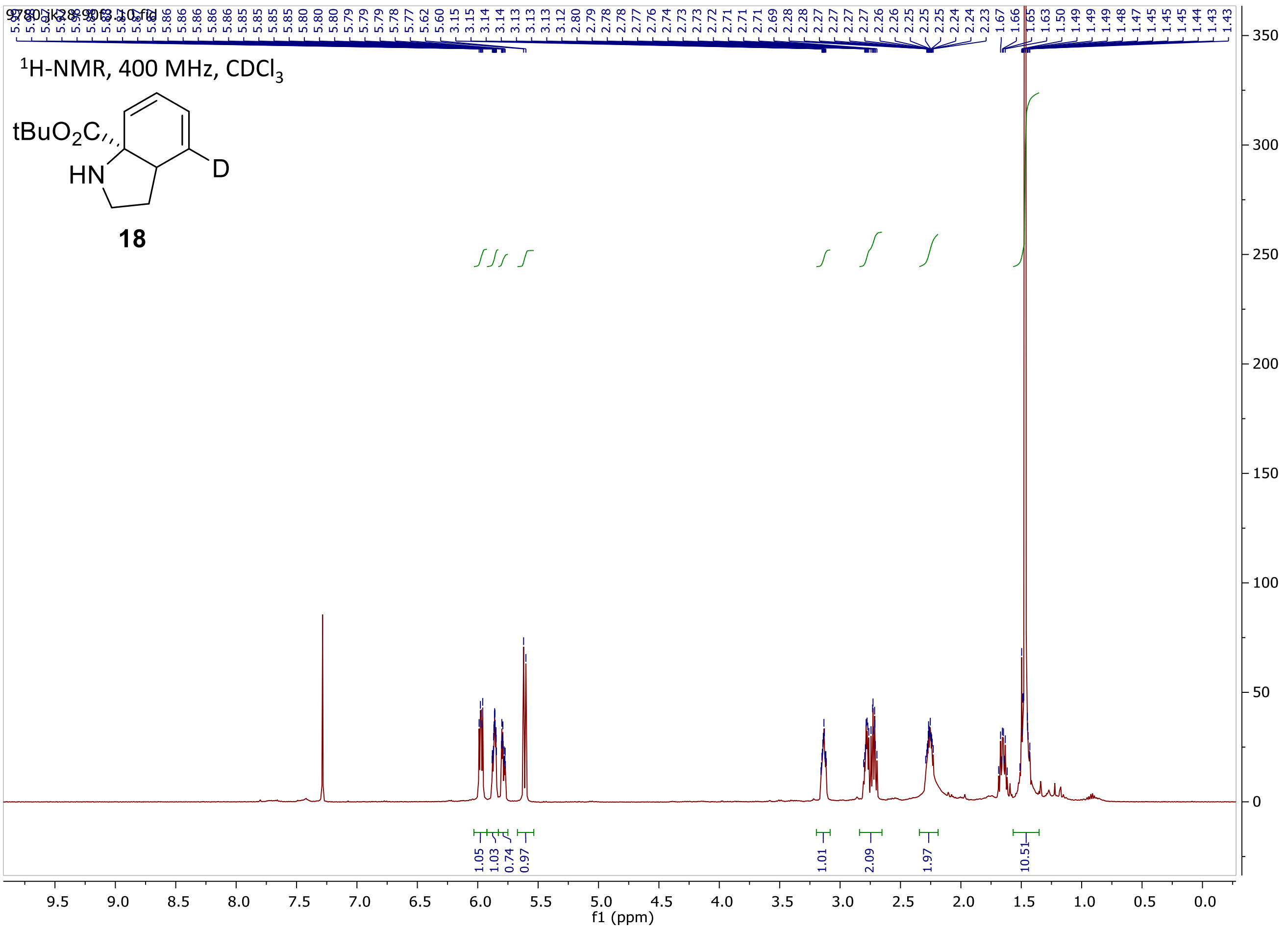

^{1}H-NMR, 400 MHz, $CDCl_3$
18
f1 (ppm)

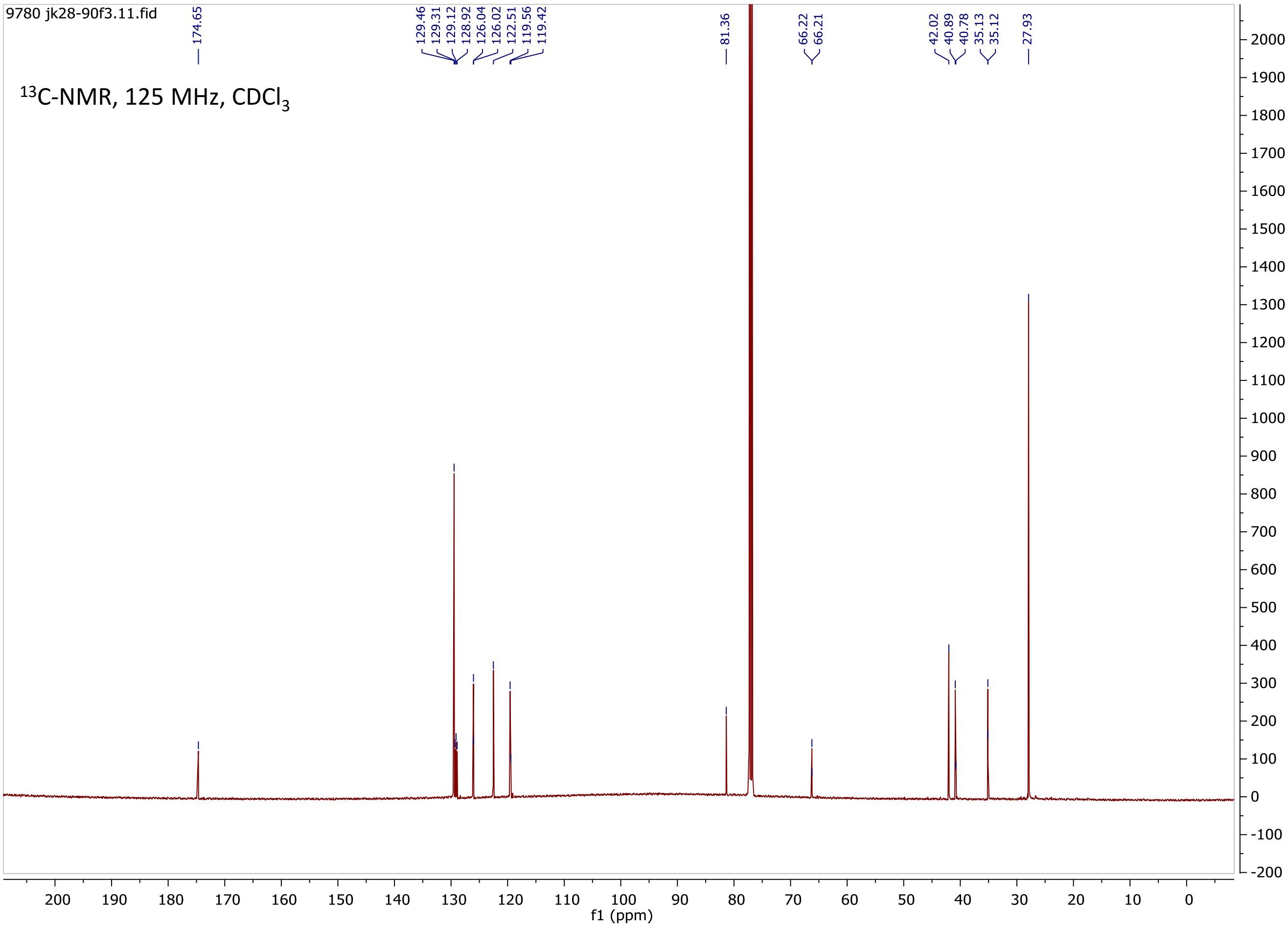

9780 jk28-90f3.11.fid
^{13}C-NMR, 125 MHz, $CDCl_3$
174.65
129.46
129.31
129.12
128.92
126.04
126.02
122.51
119.56
119.42
81.36
66.22
66.21
42.02
40.89
40.78
35.13
35.12
27.93
f1 (ppm)

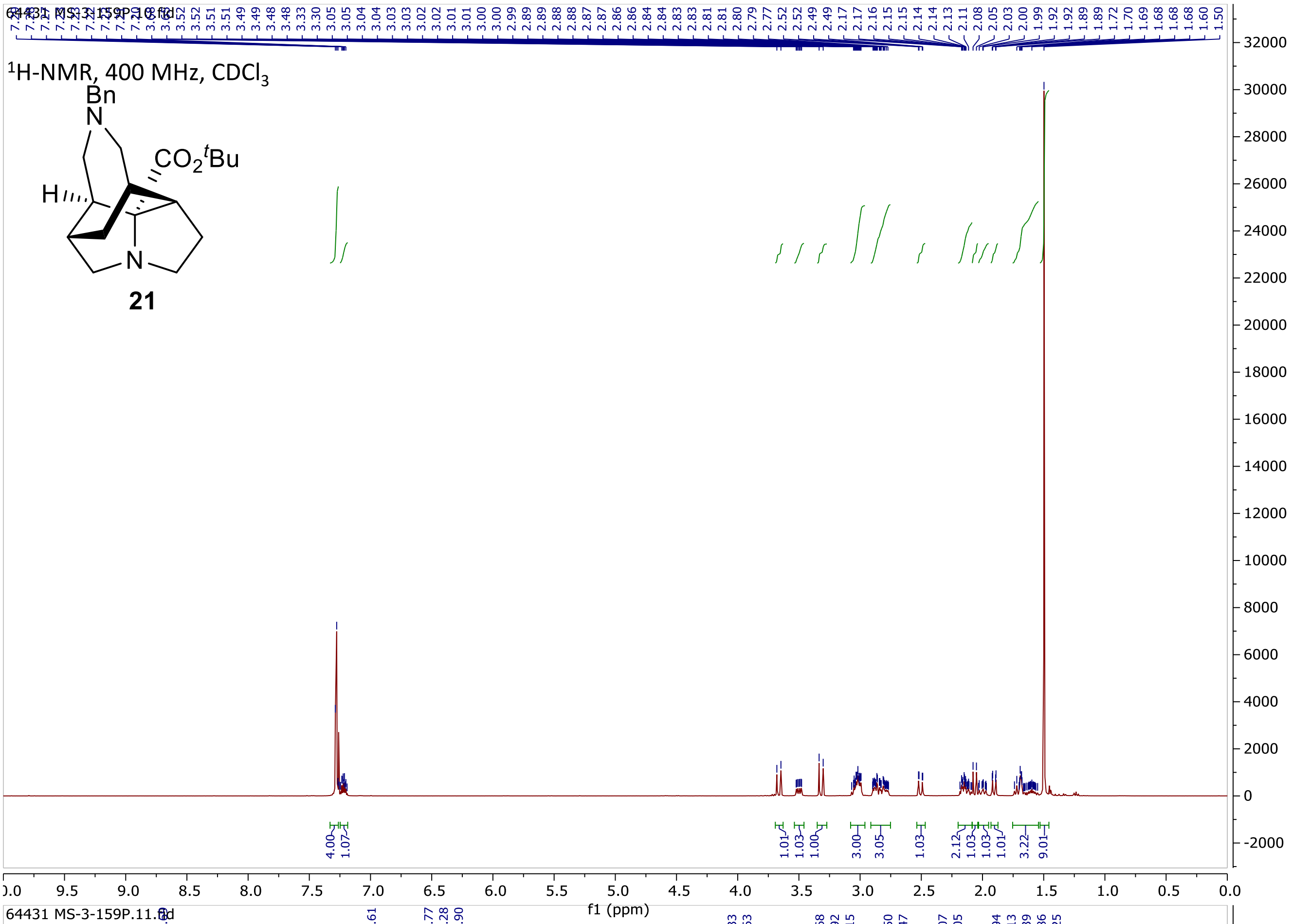

^{1}H-NMR, 400 MHz, $CDCl_3$
21

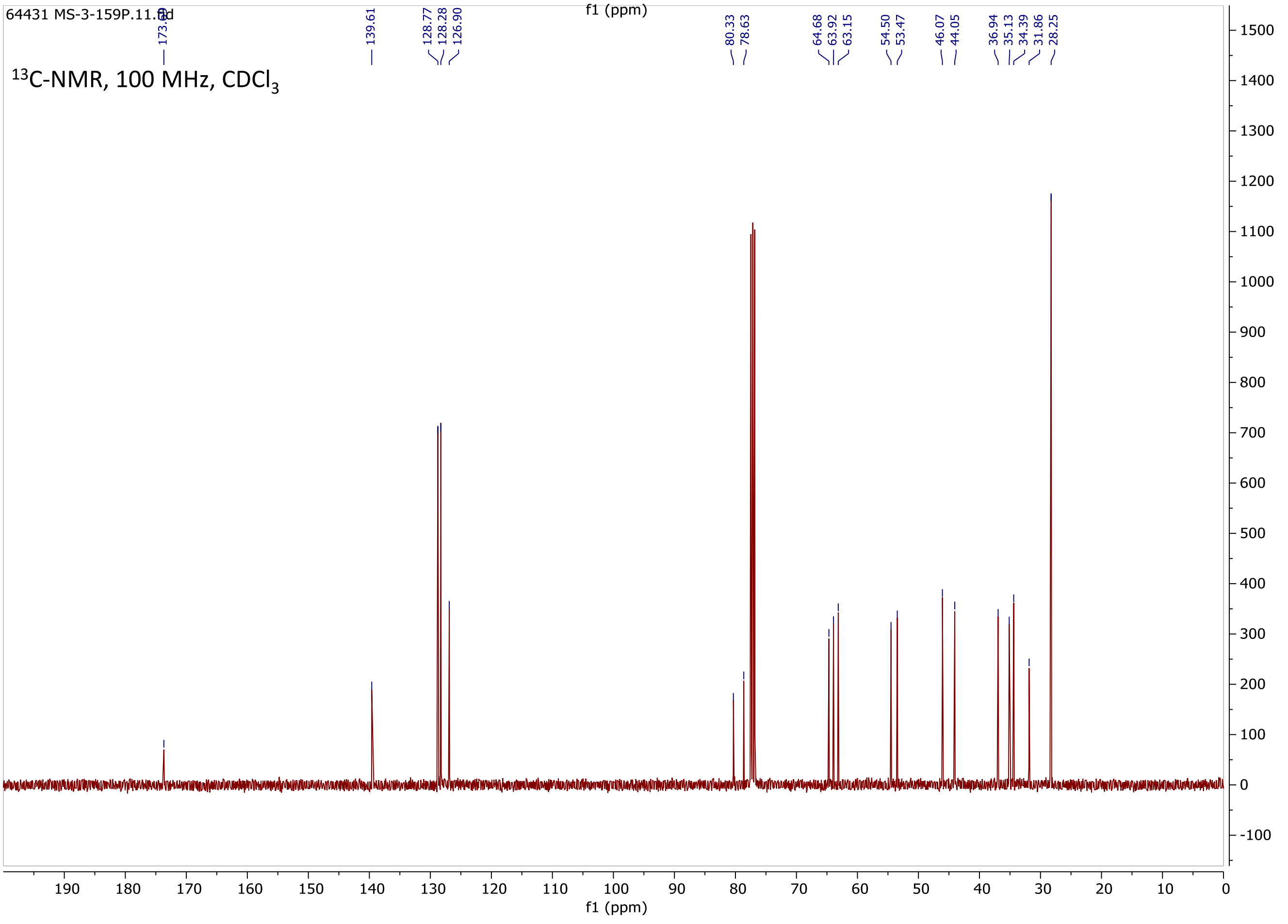

64431 MS-3-159P.11.fid
^{13}C-NMR, 100 MHz, $CDCl_3$
139.61
128.77
128.28
126.90
80.33
78.63
64.68
63.92
63.15
54.50
53.47
46.07
44.05
36.94
35.13
34.39
31.86
28.25
f1 (ppm)

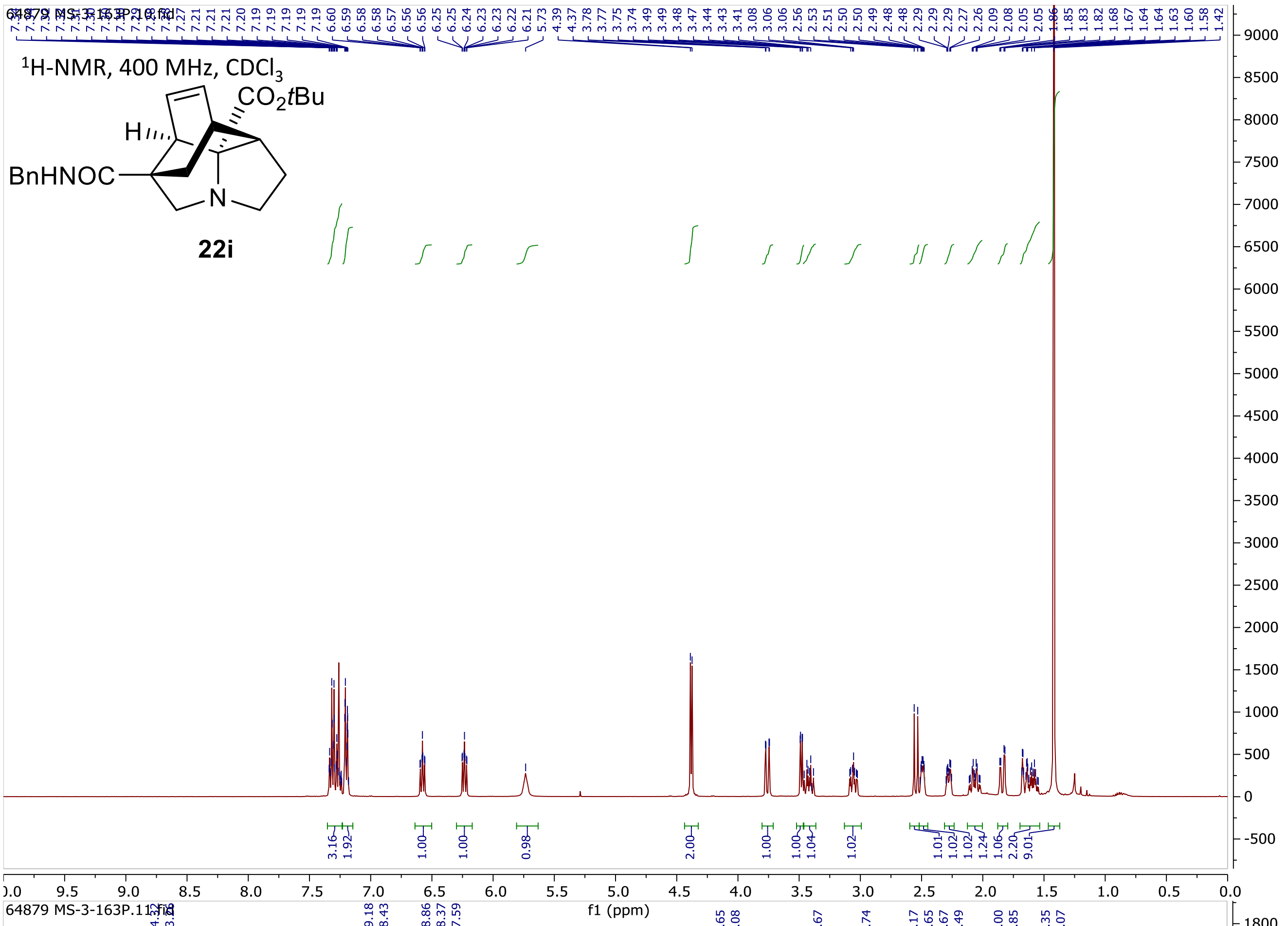
64879 MS-3-163P.10.fid
1H-NMR, 400 MHz, CDCl3
CO2tBu
H
BnHNOC
N
22i
3.16
1.92
1.00
1.00
0.98
2.00
1.00
1.00
1.04
1.02
1.01
1.02
1.02
1.24
1.06
2.20
9.01
f1 (ppm)

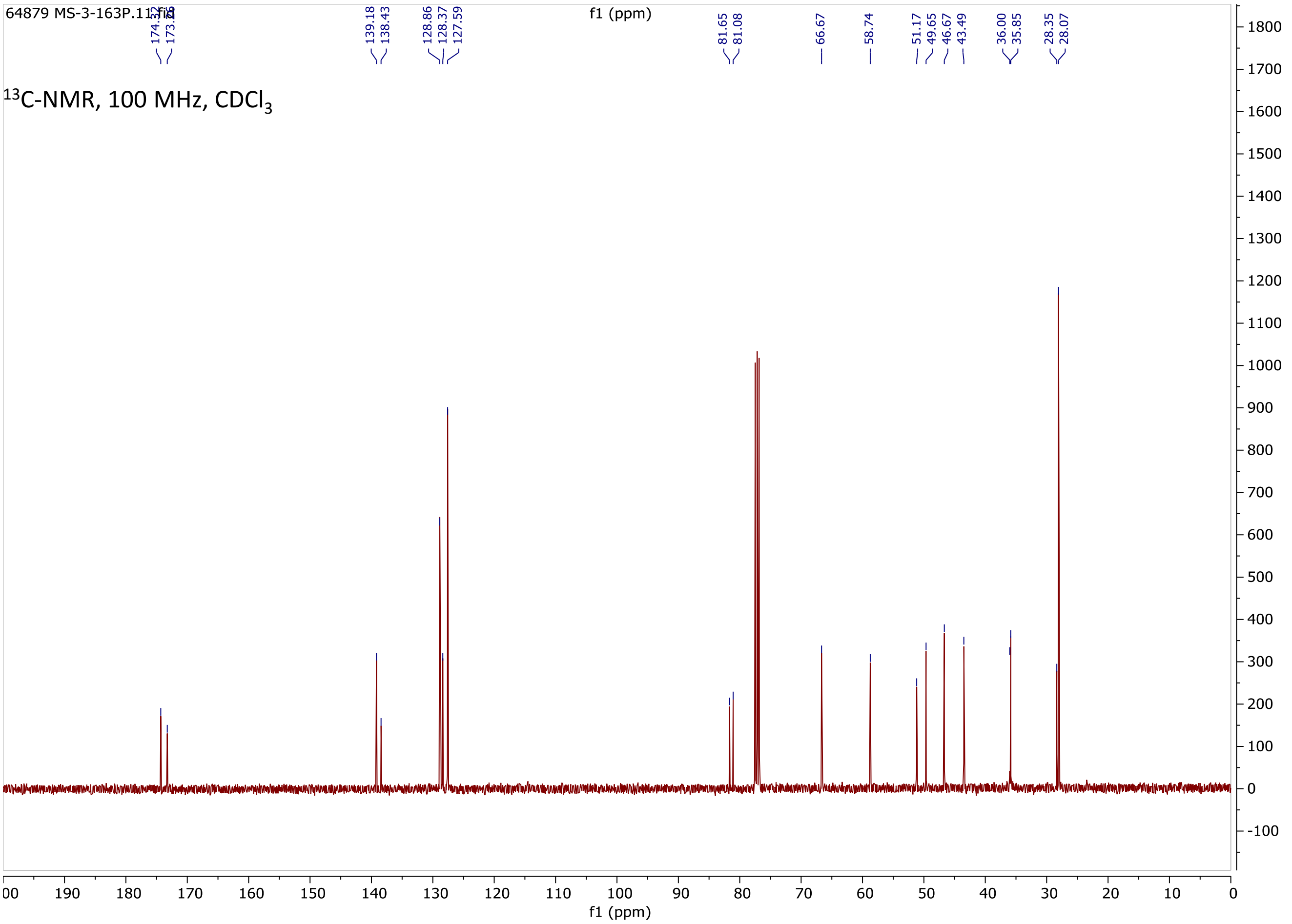
64879 MS-3-163P.11.fid
13C-NMR, 100 MHz, CDCl3
139.18
138.43
128.86
128.37
127.59
81.65
81.08
66.67
58.74
51.17
49.65
46.67
43.49
36.00
35.85
28.35
28.07
f1 (ppm)

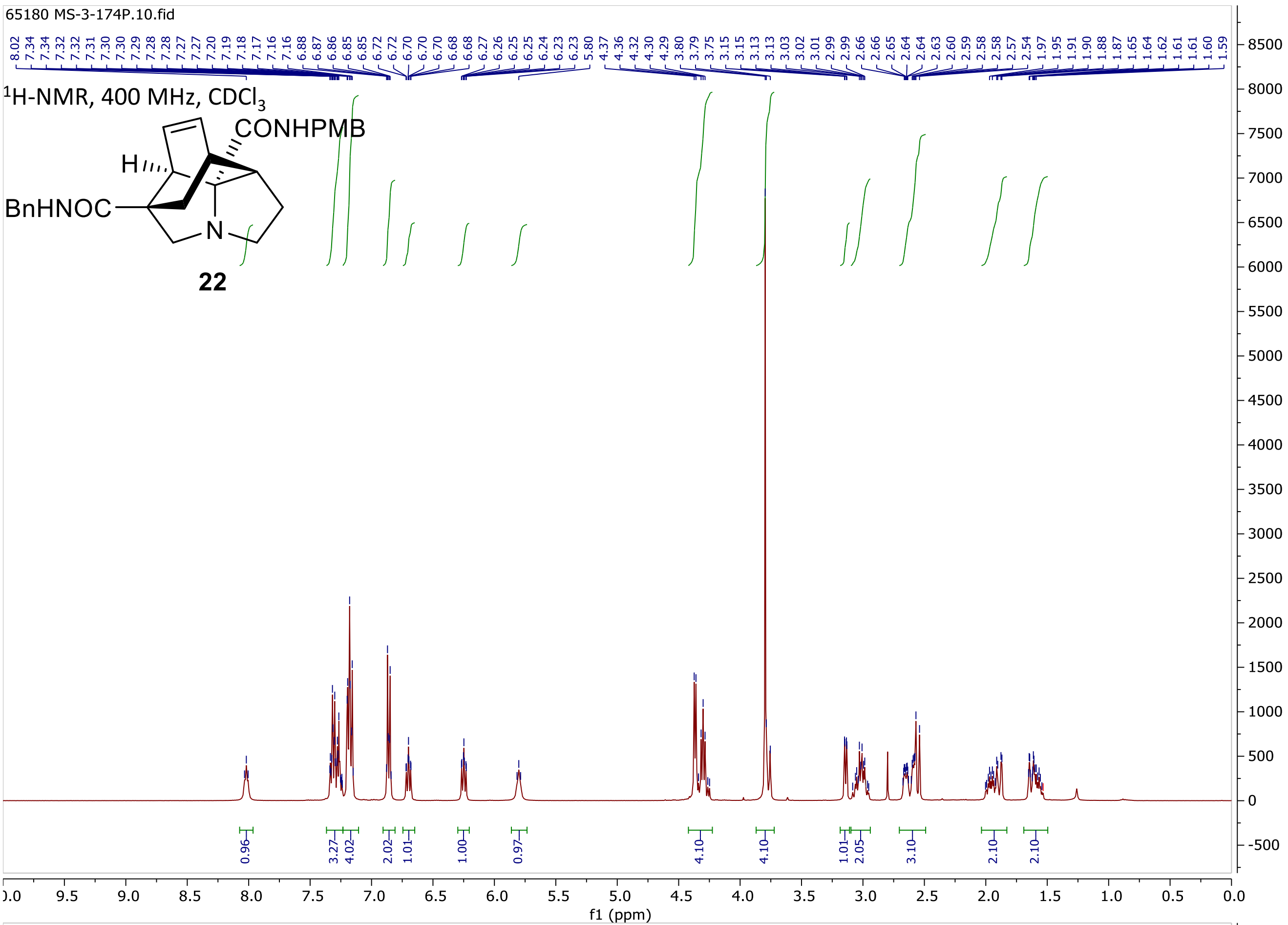
65180 MS-3-174P.10.fid
1H-NMR, 400 MHz, CDCl3
CONHPMB
H
BnHNOC
N
22
8.02 7.34 7.34 7.32 7.32 7.31 7.30 7.30 7.29 7.28 7.28 7.27 7.27 7.20 7.19 7.18 7.17 7.16 7.16 6.88 6.87 6.86 6.85 6.72 6.72 6.70 6.70 6.70 6.68 6.68 6.27 6.26 6.25 6.25 6.24 6.23 6.23 5.80 4.37 4.36 4.32 4.30 4.29 3.80 3.79 3.75 3.15 3.15 3.13 3.13 3.03 3.02 3.01 2.99 2.99 2.66 2.66 2.65 2.64 2.64 2.63 2.60 2.59 2.58 2.58 2.57 2.54 1.97 1.95 1.91 1.90 1.88 1.87 1.65 1.64 1.62 1.61 1.61 1.60 1.59
0.96 3.27 4.02 2.02 1.01 1.00 0.97 4.10 4.10 1.01 2.05 3.10 2.10 2.10
f1 (ppm)

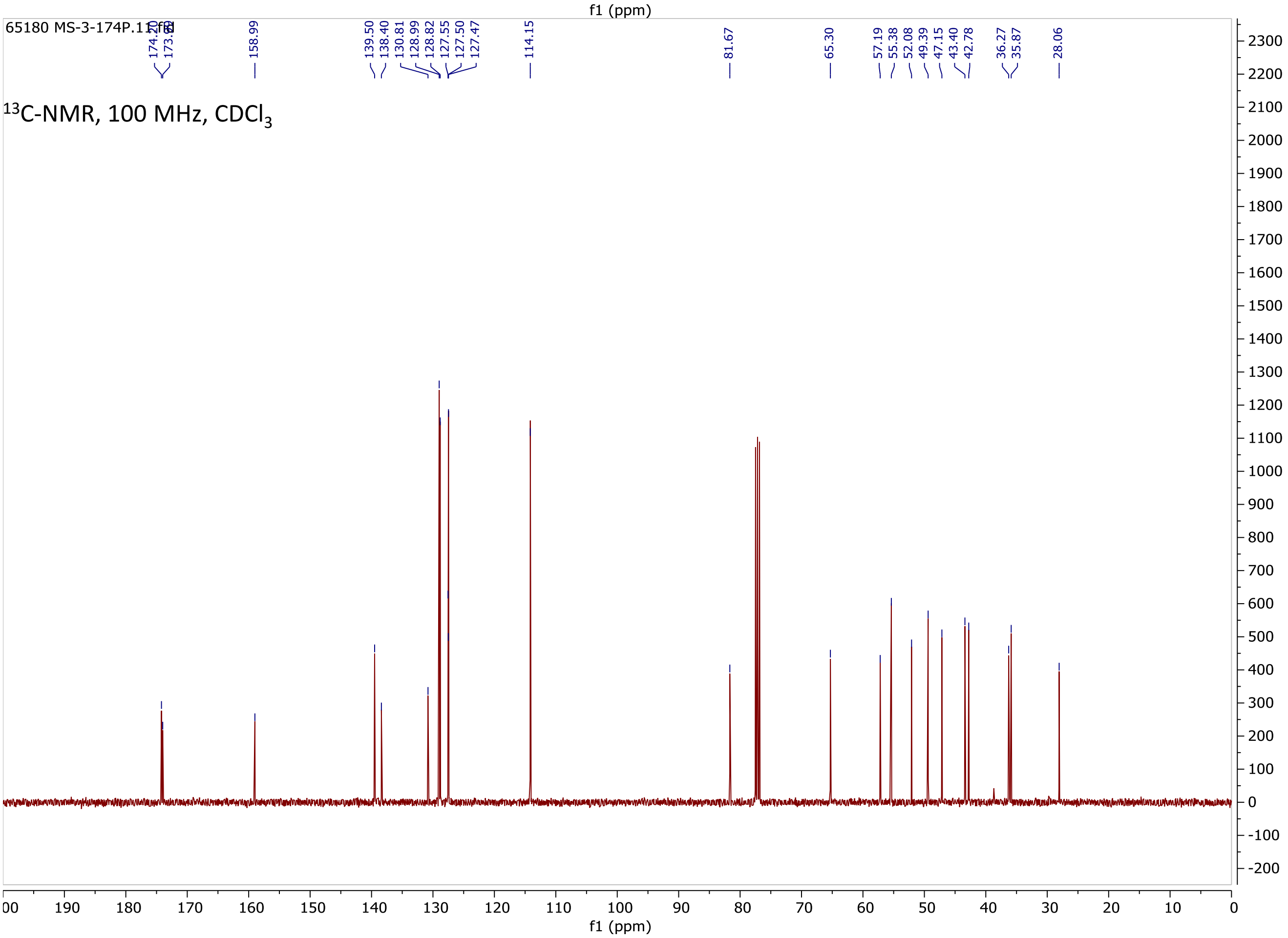
13C-NMR, 100 MHz, CDCl3
158.99 139.50 138.40 130.81 128.99 128.82 127.55 127.50 127.47 114.15 81.67 65.30 57.19 55.38 52.08 49.39 47.15 43.40 42.78 36.27 35.87 28.06
f1 (ppm)
